# Influence of wind direction on the relationship between proximity to pig farms and risk of infection with MRSA CC398 among persons without known contact to livestock: a Danish nationwide population-based study

**DOI:** 10.1007/s15010-025-02629-2

**Published:** 2025-09-08

**Authors:** Martin Rune Hassan Hansen, Jörg Schullehner, Steen Gyldenkærne, Øyvind Omland, Lise Marie Frohn, Torben Sigsgaard, Vivi Schlünssen

**Affiliations:** 1 Research Unit for Environment, Work and Health, Department of Public Health, Aarhus University, Bartholins Allé 2, Building 1260, Aarhus C, DK-8000 Denmark; 2 Department of Infectious Diseases, Aarhus University Hospital, Palle Juul-Jensens Boulevard 99, Aarhus N, DK-8200 Denmark; 3 Department of Environmental Science, Aarhus University, Frederiksborgvej 399, Roskilde, DK-4000 Denmark; 4 Department of Occupational and Environmental Medicine, Danish Ramazzini Centre, Aalborg University Hospital, Havrevangen 1, Aalborg, DK-9000 Denmark

**Keywords:** Methicillin-resistant staphylococcus aureus, MRSA CC398, Livestock-MRSA, Airborne transmission, Environmental health, Geographic information systems

## Abstract

**Background:**

Livestock-MRSA (methicillin-resistant Staphylococcus aureus) can cause infections in persons without known contact to livestock, but the route of transmission is unclear. We investigated whether the risk of livestock-MRSA infection among persons with no known contact to livestock is associated with the number of pig farms near the home, and whether this association is affected by the upwind/downwind location of the farms.

**Methods:**

Register-based case-control study of 518 persons from Denmark with clinical infections with livestock-MRSA in 2016–2021 and no known exposure to livestock, and 4,944 matched controls. Distances and angles from home addresses to all pig farms within a distance of 25 km were calculated, and compared with the mean wind direction in the area.

**Results:**

The mean number of pig farms within 13,127 m of the home address was 3.3 [0.3; 6.3] higher for cases (60.5) than controls (57.1), with a larger difference for farms upwind than downwind. The primary analysis showed that the livestock-MRSA exposure from a downwind farm was 59% [40%; 178%] of the exposure from an upwind farm, but the difference disappeared after confounder adjustment. In a post-hoc analysis, cases were surrounded by more pig farms at 50 − 6,250 m from the home address, and in the interval 1,250-6,250 m the difference was only seen in the upwind directions.

**Conclusion:**

The risk of livestock-MRSA infection among persons without known livestock contact was influenced by the number of and distance to pig farms. In an exploratory post-hoc analysis, but not the main analysis, the risk was also influenced by mean wind direction.

**Supplementary Information:**

The online version contains supplementary material available at 10.1007/s15010-025-02629-2.

## Introduction

Infections with methicillin-resistant Staphylococcus aureus (MRSA) is a significant public health problem in Denmark. Livestock-MRSA is defined as MRSA CC398 that is negative for PVL (Panton-Valentine leukocidin). In Denmark, MRSA infection is a notifiable disease, and registers showed increasing incidence of clinical infections from livestock-MRSA in the period 2010–2017, after which it has stabilized at a little above 200 cases per year [1]. Part of the explanation for the stabilized number of cases may be that reinfections are not registered, since cases are only registered the first time they test positive for a specific strain of MRSA [1]. In 2022, 1,514 Danish cases of clinical infections with MRSA were reported [1]. When a case of MRSA is reported in Denmark, the reporting physician must state whether the infected person has been in frequent contact with animals within the last 6 months. This includes “pigs, cattle, horses, porcupines, poultry, mink or other fur animals, other production animals (e.g. sheep and goats)” (author’s translation), but not “pets, e.g. cat, dog, guinea pig, etc.” (author’s translation) [2]. Likewise, it must also be reported if anyone in the household or in their close social circle has had such contact [2]. Out of the 230 (15%) cases who had livestock-MRSA infection in 2022, 138 (60%) had direct (farmworker) or indirect (farmworker in household) contact with livestock before the diagnosis, while 92 (40%) did not have any known contact to livestock [1].

Pigs and other livestock serve as a reservoir for livestock-MRSA. Most conventional pig farms in Denmark are positive for livestock-MRSA, with 95% of breeding farms testing positive in a nationally representative survey from 2019 [3], and 89% of all conventional pig farms positive in 2018 [3]. A lower prevalence was found among horses, cattle, and mink [3] (before the majority of Danish mink were culled during the COVID-19 pandemic [4]). As described above, a considerable proportion of the clinical infections with livestock-MRSA in humans are seen in persons without any known contact to livestock, raising the question of how these persons get infected. Suggested routes of infection include unreported livestock contact [5], contaminated food products [6], direct contact with colonized individuals [5, 6], and environmental spread of livestock-MRSA bacteria around pig farms [5, 6].

Previous studies have demonstrated that livestock-MRSA can be found in air and soil samples around pig farms, and further downwind than upwind from the farms [7], suggesting that the bacteria are carried by the wind to the surrounding area. Livestock-MRSA can persist in settled dust from pig stables for extended periods at indoor ambient conditions, with a survival half-life of 5 days [8]. However, it remains unclear if the spread of bacteria is large enough and their persistence long enough to be the cause of colonization and infection of people living near pig farms. The purpose of this study was therefore to assess the relationship between the number of pig farms near a person’s home address and the odds of having a clinical livestock-MRSA infection, and to investigate whether this association was modified by the general wind direction in the area.

## Methods

This was a register-based case-control study based on combinations of Danish health and administrative register data with information on the geographical location of Danish pig farms, and on wind direction in Denmark. A detailed analytical protocol was deposited online before analyses began [9], and all analyses were pre-planned unless explicitly described as post-hoc. A summary of the analysis methods is provided below. For details, we refer to the protocol [9].

The cases were all persons in Denmark diagnosed for the first time with a clinical infection with a PVL-negative strain of MRSA CC398 between January 1, 2016 and December 31, 2021, with no known contact to livestock – either direct (farmworker) or indirect (through household members who are farmworkers). The controls were a matched sample of the Danish background population. We studied the interaction between wind direction and the proximity to pig farms on the odds of being an MRSA CC398 case. A priori, we hypothesized that odds of being a MRSA CC398 case increases with an increasing number of pig farms in the vicinity of the home address, and that given the same distance and number of farms, odds will be higher if the farms are located upwind from the home address than if they are located downwind.

### Data sources

In Denmark, MRSA infection is a notifiable disease, and Statens Serum Institut (SSI) keeps a database of all cases. SSI provided us with a dataset of all relevant cases of clinical infections with MRSA CC398 (see above). The dataset included the reason for testing (“infection”, “screening”, “other” or “unknown”), the date of diagnosis and a unique personal identifier (CPR number) that allowed us to merge this information with administrative information from the CPR register (see below). In the main analysis (and in the post-hoc analyses), we only included persons where the listed reason for testing was “infection”.

All cases in the database were identified in the Danish Civil Registration System [10, 11] using their unique personal ID (CPR number). Using sampling with replacement, we randomly selected up to 10 controls for each case from the Danish background population. Controls were matched to cases by gender, age (date of birth within ± 180 days of the case) and municipality of their home addresses. The civil registration system provided information on official gender, date of birth, educational level, address history (including the coordinates of each address) and income level for each participant. For educational level (less than high school, high school, or higher than high school), we used a participant’s own value if they had an age ≥ 25 years – otherwise we used the highest value among their parents.

We excluded anyone who had lived closer than 50 m to a pig farm in the last 365 days before their diagnosis (for controls, the date of diagnosis of the corresponding case), since we assumed such persons to have animal contact (no matter if such contact was registered in the SSI database).

Owners of pig farms in Denmark are legally obliged to register their farms in The Central Husbandry register (CHR, https://chr.fvst.dk) [12]. CHR provided a dataset of all pig farms in Denmark in the years 2006–2021, along with the number of animals in each farm and their coordinates. In our analyses, we included farms with at least 10 animals.

From the ERA5 database [13], we downloaded wind data for a grid that covered Denmark with a spatial resolution of 0.25 × 0.25 degrees (approximately 16 × 28 km) and temporal resolution of one hour. The dataset consisted of reanalyzed data based on the combination of ECMWF (European Centre for Medium-Range Weather Forecast) Integrated Forecast System model data and observations through data assimilation, and contained information on the speed and direction of the wind 10 m above surface level.

### Statistical analyses

#### Primary and sensitivity analyses

**Fig. 1:**
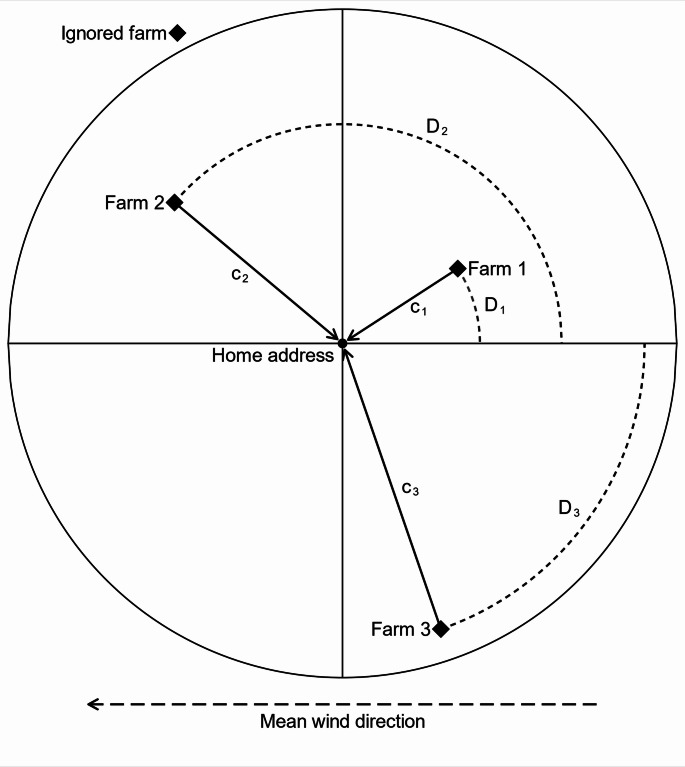
Schematic illustration of parameters in the main exposure model

For each participant in the study, we determined the straight-line distance $\:c$ to all pig farms within a 25-km radius from the home address and calculated the angle (relative to North) between the farm and the address. We then calculated the angle $\:D$ between the farm-address vector and a vector representing the mean wind direction in the area. The time window for calculating $\:D$ was determined in a data-driven approach as described below. $\:D$ lies in the interval ]-180°; 180°], and as our measure for wind direction we used $\:abs\left(D\right)$, lying in the interval [0°; 180°]. These parameters are illustrated schematically in Fig. 1.

In this example, three farms are located within the search radius around the home address, indicated by the circle. One farm is located outside the search radius and is ignored. Each of the relevant farms *i* has a distance $\:{c}_{i}$ to the home address, and each of the vectors connecting a farm with the home address forms an angle $\:{D}_{i}$ with the vector representing the mean wind direction in the area (in this example from east).

A priori, we hypothesized that a proxy measure for the total exposure from the farms around an address ($\:{e}_{total})\:$ could be calculated using an inverse distance-weighting formula:

$$\:{e}_{total}=\sum_{i}{h}_{i}\times\:{{c}_{i}}^{-p}$$

where $\:{c}_{i}$ is the distance to the *i*^*th*^ farm, p is a constant (to be empirically determined) and $\:{h}_{i}$ is a unitless weighting factor (unique to each combination of address and pig farm) that accounts for the wind direction.

We also hypothesized that the weighting factor $\:{h}_{i}$ could be described as a function of $\:abs\left(D\right)$ and of the dimensionless constant $\:{h}_{dw}\:$ that equals the ratio between the measure for exposure from a farm located downwind and an identical farm located upwind at the same distance:

$$\:{h}_{i}={h}_{dw}+\left(1-{h}_{dw}\right)\times\:\frac{\text{cos}\left(abs\left({D}_{i}\right)\right)+1}{2}$$

**Fig. 2:**
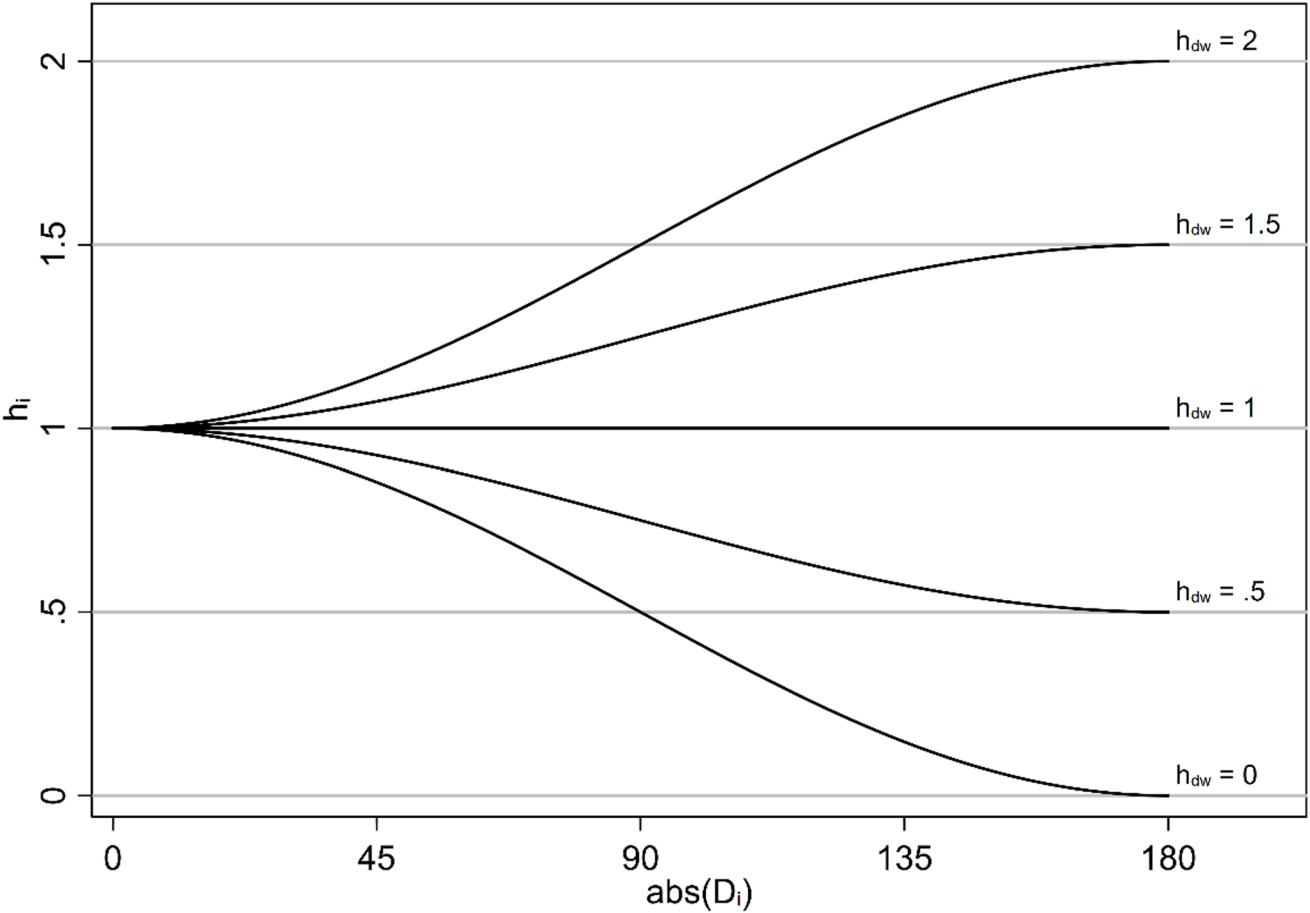
Theoretical relationship between $\:abs\left(D\right)$ and exposure weighting factor $\:{h}_{i}$ for a single pig farm

For an illustration of how the exposure weighting factor $\:{h}_{i}$ changes as a function of $\:{h}_{dw}$ and $\:abs\left(D\right)$, see Fig. 2.

The idea behind the main analysis was to determine whether a model that accounts for wind direction ($\:{h}_{dw}\ne\:1$) is better at predicting MRSA CC398 case status than a model agnostic to wind direction ($\:{h}_{dw}=1$). In addition to $\:{h}_{dw}$, the formulae required us to decide on relevant values of $\:p$, as well as the time interval over which to summarize $\:abs\left(D\right)$, and the search radius for the farms. To do this, we performed a random hyperparameter search [14]. We conducted 1,000 analyses that each had random values of p (from 0 to 3), time window for averaging of wind speed and angle (7, 14, 21, 30, 90, 180, 270 or 365 days) and search radius (from 50 to 25,000 m in steps of 1 m). In each of these analyses, we calculated $\:{e}_{total}$ for all participants based on all possible values of $\:{h}_{dw}$ (from 0 to 2 in steps of 0.01), ran a logistic regression between $\:{e}_{total}$ and odds of being an MRSA CC398 case, and quantified the level of agreement between the model predictions and actual case status using Cohen’s kappa [15]. The model with the highest value of Cohen’s kappa used a search radius of 13,127 m, a time interval of 180 days, and an IDW power ($\:p$) of 1.184. Once these hyperparameters had been determined, we calculated the value of $\:{h}_{dw}$ that had the highest kappa and determined its confidence interval using bias-corrected bootstrapping with 1,000 samples. The optimum $\:{h}_{dw}$ was determined both in crude and adjusted analyses (including age, gender, education and income as covariates). To account for non-linear relationships, age and income was modelled using restricted cubic splines with four knots, the location of which determined by the distribution of the variables [16].

A number of sensitivity analyses were conducted. They assumed a linear relationship between $abs\left(D\right)$ and $\:{h}_{i}$, weighted exposure from each pig farm by the number of animals in the farm or by the prevalence of livestock-MRSA in Danish pig farms in the relevant calendar year, included cases where the reason for testing was listed as “unknown” or “other”, only excluded potential participants if they had lived closer than 10 m from a pig farm in the last year (instead of 50 m), or evaluated model performance using Brier score [17] instead of Cohen’s kappa [15]. For details, refer to the online protocol [9].

#### Post-hoc secondary analysis

As described below, a discrepancy was observed between purely descriptive statistics on the number of farms and pigs in a zone of 13,127 m around participants’ homes, and results from the main analysis that used more complex exposure metrics. To explore the reasons for this discrepancy, a post-hoc secondary analysis was conducted. In this analysis, we calculated the number of pig farms around participants’ homes in ring-shaped zones with varying outer radii (from 1,250 to 25,000 m in steps of 1,250 m), split into segments based on the value of $\:abs\left(D\right)$ (first segment had 0°≤ $\:abs\left(D\right)$≤22.5°, the second 22.5°< $\:abs\left(D\right)$≤45°, etc., the last one 157.5°<$\:abs\left(D\right)$≤180°). The difference in mean number of pig farms between cases and controls was computed in each ring segment using linear regression, 95% bias-corrected confidence intervals were calculated using bootstrapping with 1,000 samples, and the results presented graphically. This analysis was repeated for all the previously described values of time intervals, and in both crude and adjusted versions.

### Software tools

Data were managed using Stata 15 (StataCorp, College Station, Texas, US). Geographical analyses were conducted using Esri ArcGIS Pro (Environmental Systems Research Institute, Redlands, California, US) and Python 3.6 (Python Software Foundation, www.python.org). Stata 15 was used for all statistical analyses.

## Results

**Fig. 3:**
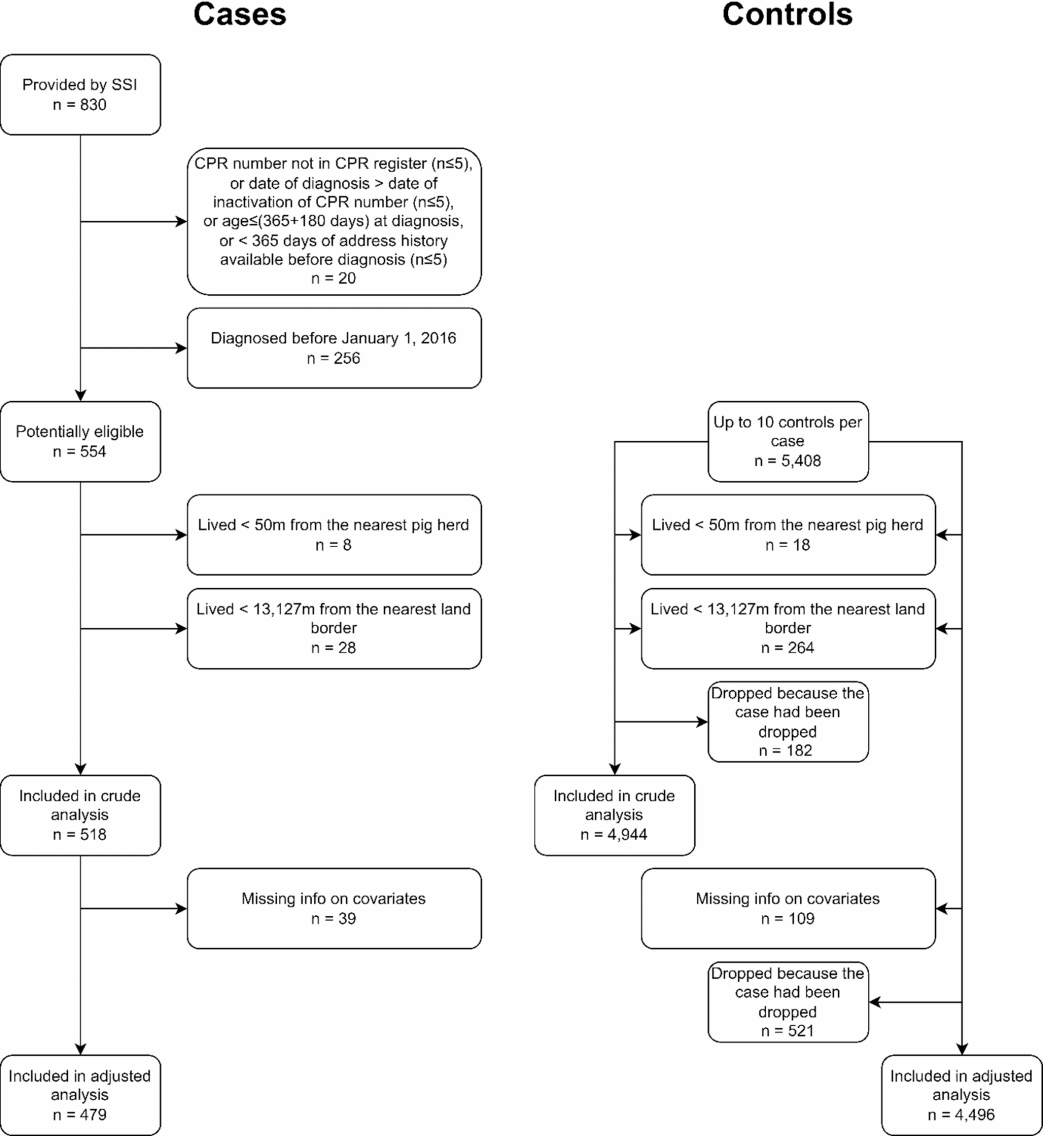
Flowchart of participant in- and exclusion in main analysis

A flowchart on the in- and exclusion of participants is provided in Fig. 3. A priori, we were aiming for up to 10 controls to each case (followed by exclusion of persons who were ineligible because they e.g. had lived too close to an international border). For most MRSA CC398 cases, the number of controls included in the analyses was close to 10. Of the 518 cases in the main crude analysis, 393 cases (75.9%) had 10 controls, 86 cases (16.6%) had 9 controls, 13 cases (2.5%) had 8, and 26 cases (5.0%) had 7 or fewer controls. For the 479 cases in the main adjusted analysis, 300 cases (62.6%) had 10 controls, 131 cases (27.3%) had 9, 22 cases (4.6%) had 8, and 26 cases (5.4%) had 7 or fewer controls.

Descriptive statistics for the participants included in the crude main analysis is provided in Table 1. Controls were matched to cases by gender, age and municipality, resulting in no differences between the groups in terms of gender composition or age distribution. Cases were slightly less wealthy than controls, and slightly (but not statistically significantly) less educated. The mean distance from the home address to the nearest pig farm was significantly lower for cases than for controls. There were significantly more pig farms and individual pigs in the 13,127-meter zones around the homes of cases than in the corresponding zones for controls, with clear trends towards larger differences in means for smaller values of $\:abs\left(D\right)$. In other words, there were larger differences in the number of farms and pigs upwind than downwind of addresses, which is what would be expected if MRSA CC398 bacteria were spread by wind around the farms.

**Table 1:** Descriptive statistics for the study population included in the crude main analysis

Variable		Cases	Controls	Combined	Association	*p*
n*		518 (100.0)	4,944 (100.0)	5,462 (100.0)	-	-
Gender*	Female	253 (48.8)	2,399 (48.5)	2,652 (48.6)	OR = 0.99 [0.82; 1.18]	0.890
	Male	265 (51.2)	2,545 (51.5)	2,810 (51.4)		
Age		58.8 (21.8)	58.5 (21.7)	58.6 (21.7)	Δ(x̄) = 0.3 [-1.7; 2.3]	0.781
Income percentile	Mean (SD)	46.1 (25.5)	48.7 (26.8)	48.5 (26.7)	Δ(x̄) = -2.6 [-5.0; -0.2]	0.032
	n missing*	26 (5.0)	25 (0.5)	51 (0.9)	-	-
Educational level*	Less than high school	360 (69.5)	3,291 (66.6)	3,651 (66.8)	-	0.210
	High school	14 (2.7)	171 (3.5)	185 (3.4)		
	Higher than high school	129 (24.9)	1,397 (28.3)	1,526 (27.9)		
	n missing	15 (2.9)	85 (1.7)	100 (1.8)		
Distance to nearest pig farm	Mean (SD)	2,289.7 (1,857.9)	2,621.3 (1,987.1)	2,589.8 (1,977.5)	Δ(x̄) = -331.6 [-500.9; -162.2]	< 0.001
	n with distance > 25,000 m*	-	-	12 (0.2)	-	-
Number of farms within 13,127 m	Any $\:abs\left(D\right)$	60.5 (33.3)	57.1 (32.2)	57.5 (32.3)	Δ(x̄) = 3.3 [0.3; 6.3]	0.031
	0°≤ $\:abs\left(D\right)$≤45°	15.7 (11.6)	14.5 (10.8)	14.6 (10.9)	Δ(x̄) = 1.2 [0.2; 2.3]	0.023
	45°< $\:abs\left(D\right)$≤90°	15.9 (10.5)	14.8 (9.6)	14.9 (9.7)	Δ(x̄) = 1.1 [0.2; 2.1]	0.018
	90°< $\:abs\left(D\right)$≤135°	15.0 (9.6)	14.5 (9.8)	14.6 (9.8)	Δ(x̄) = 0.5 [-0.4; 1.4]	0.242
	135°< $\:abs\left(D\right)$≤180°	13.8 (10.6)	13.3 (10.9)	13.3 (10.9)	Δ(x̄) = 0.5 [-0.5; 1.4]	0.358
Thousands of pigs within 13,127 m	Any $\:abs\left(D\right)$	68.4 (38.9)	64.5 (37.6)	64.8 (37.8)	Δ(x̄) = 3.9 [0.4; 7.4]	0.028
	0°≤ $\:abs\left(D\right)$≤45°	17.6 (14.1)	16.2 (13.3)	16.3 (13.4)	Δ(x̄) = 1.5 [0.2; 2.7]	0.024
	45°< $\:abs\left(D\right)$≤90°	17.8 (12.6)	16.7 (11.7)	16.8 (11.8)	Δ(x̄) = 1.1 [0.0; 2.3]	0.050
	90°< $\:abs\left(D\right)$≤135°	17.3 (12.4)	16.5 (12.0)	16.6 (12.1)	Δ(x̄) = 0.8 [-0.3; 1.9]	0.155
	135°< $\:abs\left(D\right)$≤180°	15.6 (13.2)	15.1 (13.4)	15.1 (13.3)	Δ(x̄) = 0.5 [-0.7; 1.7]	0.389

Variables marked with an * are categorical and presented as count (percentage), remaining variables are continuous and presented as mean (SD). OR indicates odds ratio, Δ(x̄) indicates difference in means. For gender, the reported OR is for being male. For the number of participants with distance > 25,000 m to nearest pig farm, results are not stratified by case status, as regulations from Statistics Denmark prohibit the export of counts < 5. For educational level, the test for difference between cases and controls excluded persons with missing information on education (i.e., missing values were not coded as a separate category)

**Fig. 4:**
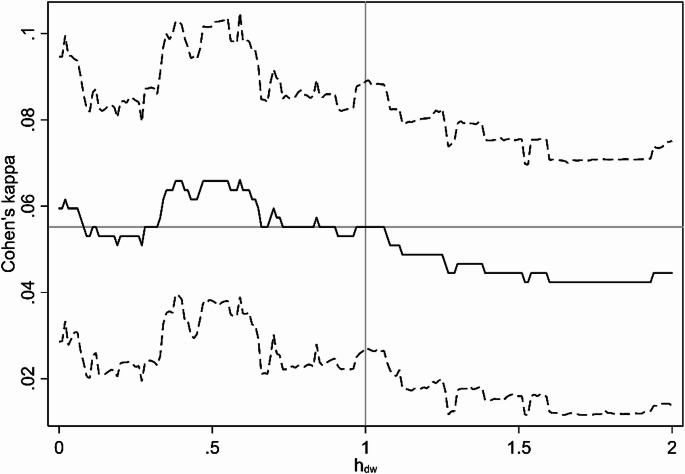
Results from main analysis (crude). Cohen’s kappa as a function of h_dw_

**Fig. 5:**
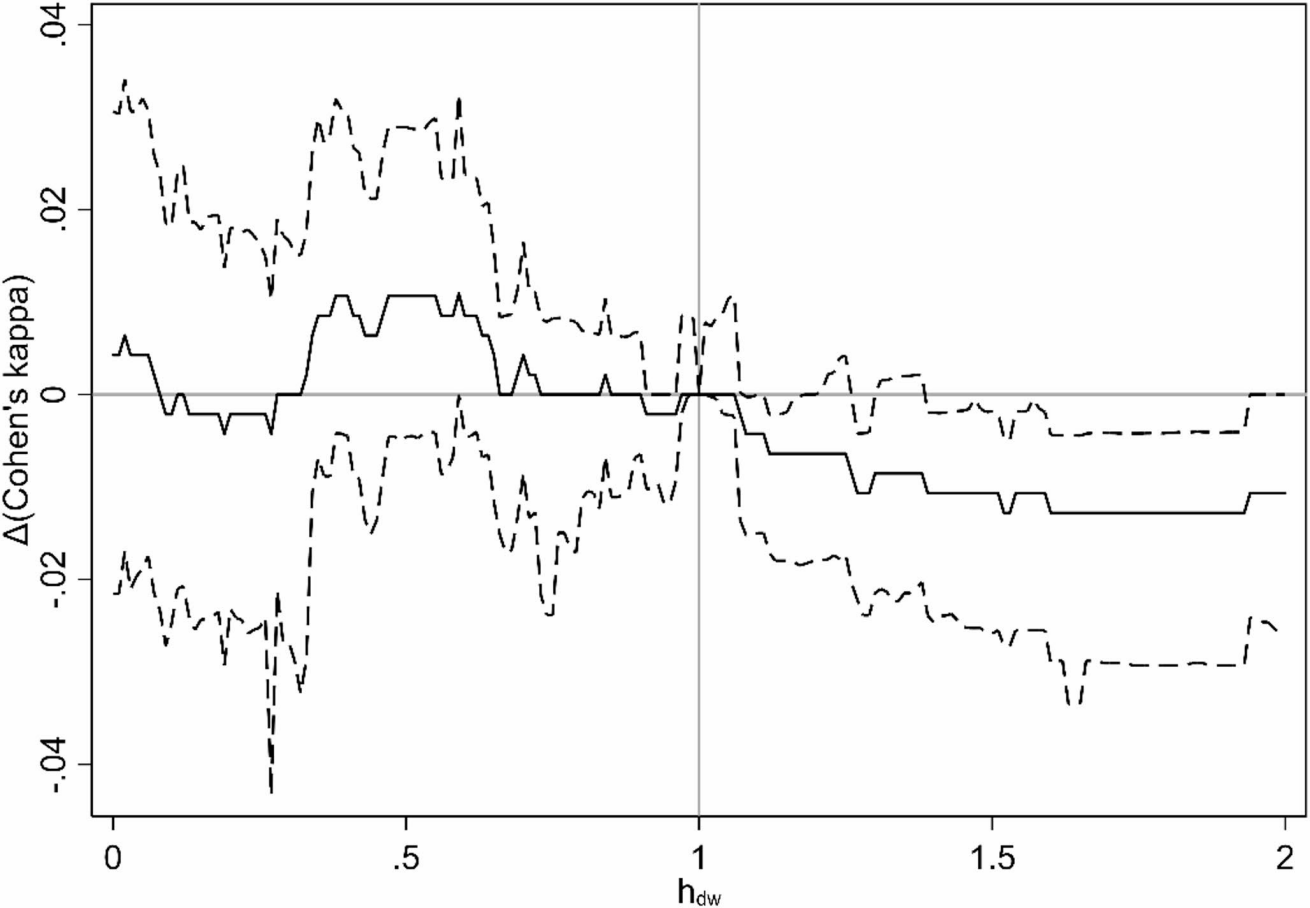
Results from main analysis (crude). Δ(Cohen’s kappa) as a function of h_dw_

**Fig. 6:**
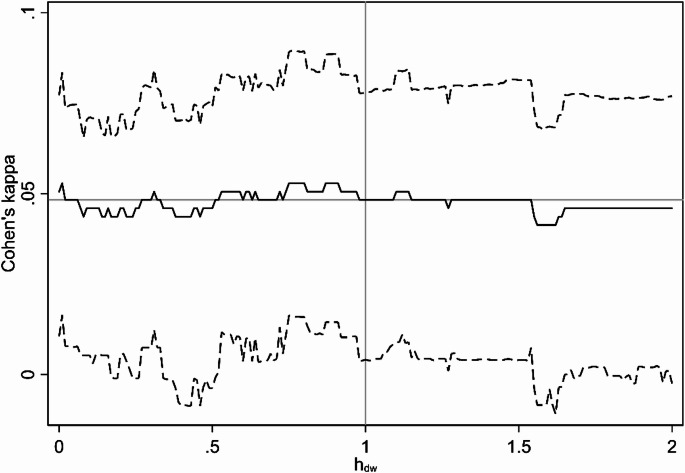
Results from main analysis (adjusted). Cohen’s kappa as a function of h_dw_

**Fig. 7:**
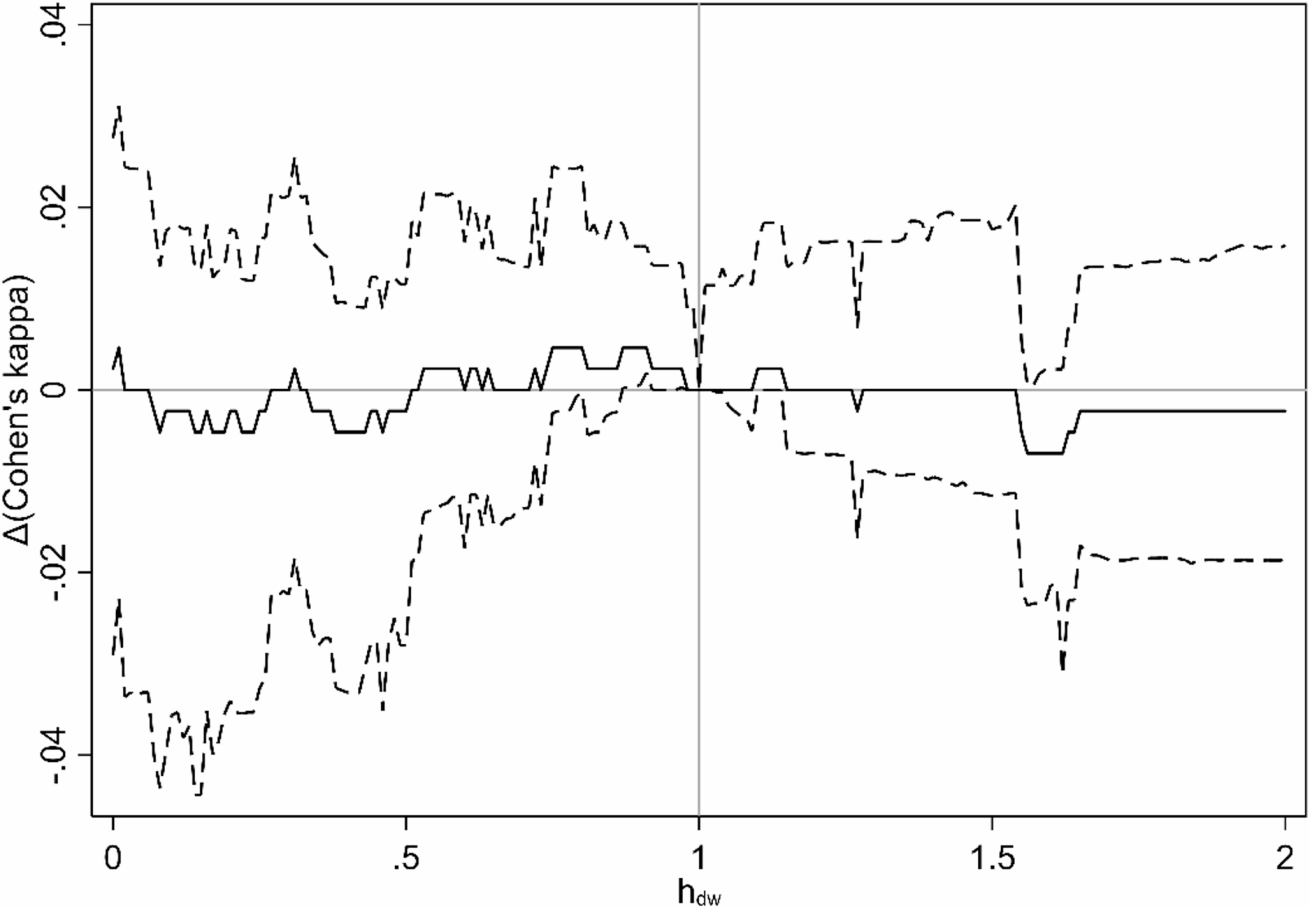
Results from main analysis (adjusted). Δ(Cohen’s kappa) as a function of h_dw_

The main crude analysis had an estimated optimum $\:{h}_{dw}=0.59\:[0.40;1.78]$, meaning that the crude model fit was best under the assumption that exposure from a pig farm downwind of an address was 59% of the exposure from a pig farm located upwind, but the difference was not statistically significant. This finding was confirmed by plots of Cohen’s kappa and Δ(Cohen’s kappa) (defined as the difference in Cohen’s kappa from a wind-agnostic model with $\:{h}_{dw}=1$) as a function of $\:{h}_{dw}$ (Figs. 4 and 5), showing a non-significant tendency for $\:{h}_{dw}<0$ to result in better model fit. These effects disappeared in the main adjusted analysis, that had an estimated optimum $\:{h}_{dw}=0.89\:[0.05;2.00]$, with no clear trends in Cohen’s kappa as a function of $\:{h}_{dw}$ (Figs. 6 and 7).

Results from sensitivity analyses are provided in online supplement S3 and were similar to the results from the main analysis.

Solid line is estimate, dashed lines are 95% confidence interval (based on bias-corrected bootstrapping with 1,000 resamplings).

Solid line is estimate, dashed lines are 95% confidence interval (based on bias-corrected bootstrapping with 1,000 resamplings). Δ(Cohen’s kappa) is defined as the difference in Cohen’s kappa between a model with a given $\:{h}_{dw}$, and a wind-agnostic model with $\:{h}_{dw}=1$.

Solid line is estimate, dashed lines are 95% confidence interval (based on bias-corrected bootstrapping with 1,000 resamplings). Adjusted for age, gender, education and income.

Solid line is estimate, dashed lines are 95% confidence interval (based on bias-corrected bootstrapping with 1,000 resamplings). Δ(Cohen’s kappa) is defined as the difference in Cohen’s kappa between a model with a given $\:{h}_{dw}$, and a wind-agnostic model with $\:{h}_{dw}=1$. Adjusted for age, gender, education and income.

**Fig. 8:**
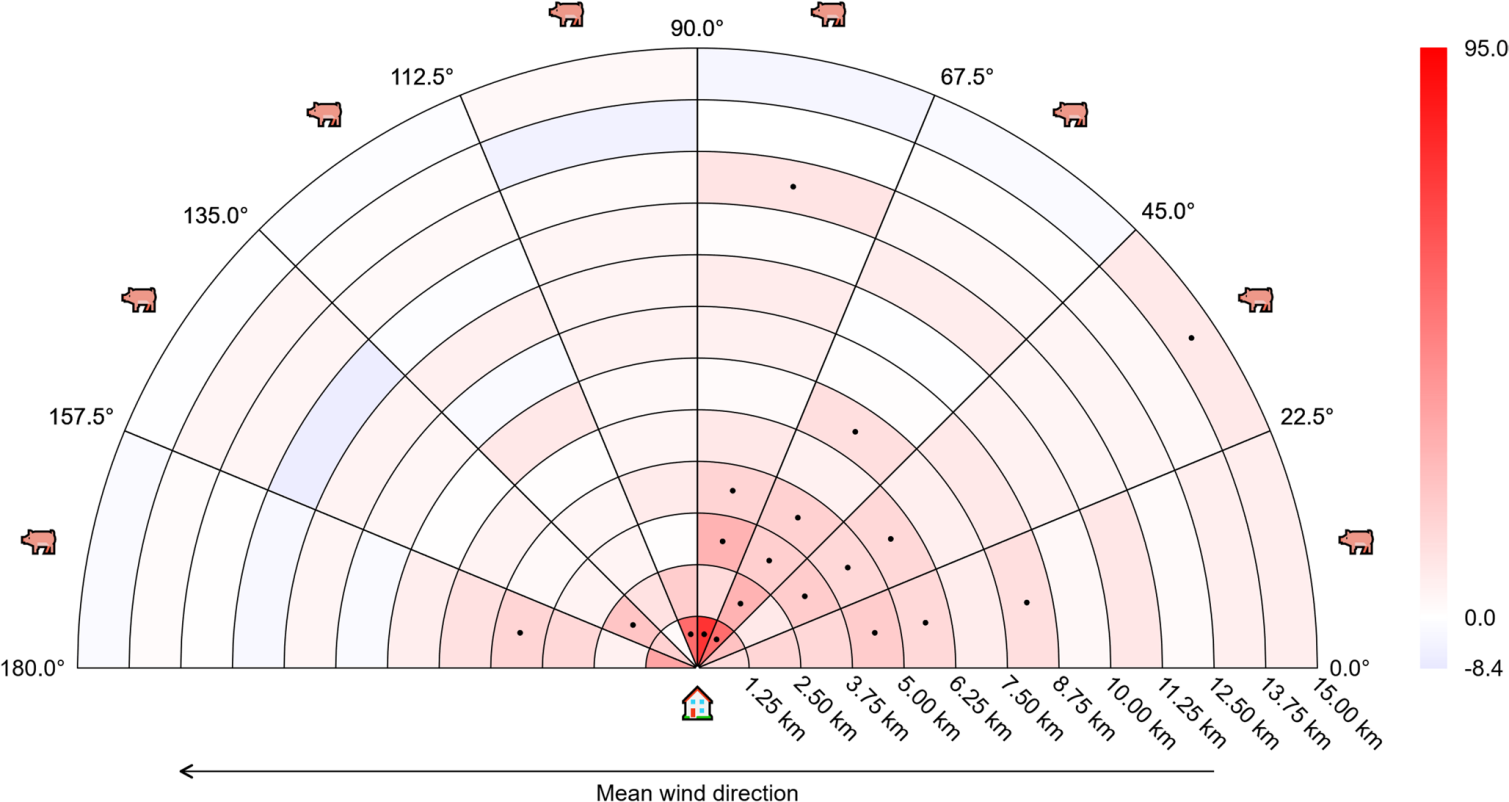
Results from post-hoc secondary analysis with time window 180 days (crude)

**Fig. 9:**
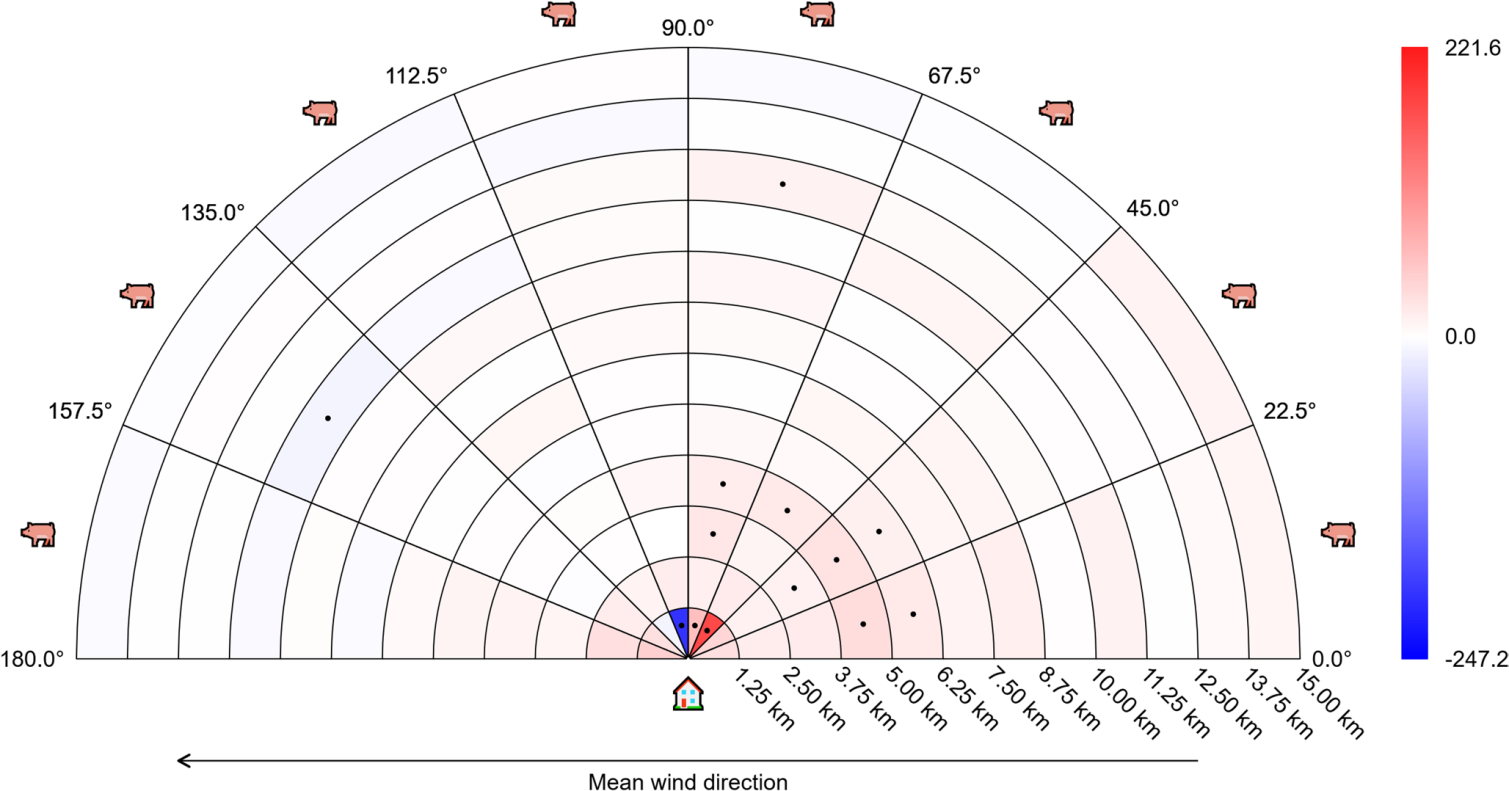
Results from post-hoc secondary analysis with time window 180 days (adjusted)

All results from the secondary post-hoc analyses are provided in online supplement S4. In Figs. 8 and 9, results have been shown for those analyses that used a time window of 180 days for summarizing wind direction, but results for other time windows were very similar. Figures 8 and 9 only include results for the distances 50 − 15,000 m – the results for the distances 15,000–25,000 m did not show any clear patterns, and are available in online appendix S4. Close to the home address (the inner-most zones with a distance of 50 − 1,250 m from the address), MRSA CC398 cases were surrounded by a higher number of pig farms, but there was no clear pattern as to whether the pig farms were located upwind or downwind. However, slightly further away from the address (the zones with distance 1,250-6,250 m), there was a clear pattern for the number of pig farms to be higher for cases than for controls, but mainly in the upwind direction (which would be expected if MRSA CC398 bacteria were spreading by wind) (Fig. 8). This pattern persisted after adjustment for age, gender, income and education (Fig. 9).

Zones are color-coded according to the difference between cases and controls, relative to the mean number for controls. Red is positive (more pig farms for cases), blue is negative (more pig farms for controls). The color bar at the right side of the figure shows the numerical meaning of the colors (numbers are in percent). Zones marked with a dot indicate statistically significant differences between cases and controls. The house and pig symbols indicate the positions of the home address and the pig farms, respectively.

Zones are color-coded according to the difference between cases and controls, relative to the mean number for controls. Red is positive (more pig farms for cases), blue is negative (more pig farms for controls). The color bar at the right side of the figure shows the numerical meaning of the colors (numbers are in percent). Zones marked with a dot indicate statistically significant differences between cases and controls. The house and pig symbols indicate the positions of the home address and the pig farms, respectively. When calculating the relative difference between cases and controls, the difference between groups was divided by the mean value for a control with female gender, age 50 years, income percentile 50, and less than high school education.

## Discussion

In descriptive analysis, we demonstrated that the number of pig farms within 13,127 m was significantly higher for livestock-MRSA cases than controls, and with a stronger effect size for farms located upwind than downwind. The main adjusted analysis did not support a downwind effect compared to farms located upwind. However, post-hoc secondary analyses described in Figs. 8 and 9 provide a possible reason for this discrepancy. Close to the home address (50 − 1,250 m) there was no clear association between mean wind direction and the difference in mean number of farms. Further away from the address (1,250-6,250 m) there were larger differences in the mean number of farms upwind, compared to downwind. In other words, mean wind direction only seems to influence the odds of livestock-MRSA infection at intermediate distances, and not very close (50 − 1,250 m) to the address. Our main analysis assumed that the effect of wind direction would be independent of distance to the farm, which could explain why we did not demonstrate a significant effect of wind in the main analysis.

We have been unable to locate any previous studies on environmental spread of livestock-MRSA that focused on clinical infections in humans accounting for wind. In 2018, Anker et al., analyzed the risk of livestock-MRSA infection in Denmark, using 192 livestock-MRSA cases without known exposure to livestock diagnosed in 2006–2012, and healthy controls [5]. The study did not account for wind direction. Anker et al. [5] demonstrated that cases had lower mean road distance to nearest pig farm than population controls, but this difference became non-significant in a post-hoc analysis limited to three municipalities with a high density of pig farms (out of the 98 Danish municipalities). Anker et al. [5] suggested “that for persons without livestock contact, but living within a pig-farming area, the actual distance to pigs or to nearest LO MRSA CC398 case does not increase the risk of MRSA CC398 infection.” [5] However, the fact that the distance to nearest pig farms was only non-significantly lower for cases than controls in the three municipalities may have been a result of low statistical power. In the present study, cases lived significantly closer to nearest pig farm than controls did.

Previous studies have demonstrated that it is possible for humans to become transiently positive for livestock-MRSA by being exposed to indoor air at pig farms [18], and that livestock-MRSA can be detected in the environment further away downwind than upwind from pig farms [7]. In an experimental study of 34 MRSA-negative persons who visited a pig farm for one hour, 64/70 samples (91%) taken from persons who had not touched anything in the farm tested positive for livestock-MRSA immediately after the participant left the farm (the same participant could be partake in the study on multiple days, and was confirmed MRSA-negative before inclusion on a new test date) [18]. However, repeated sampling showed that only 32/70 (46%) samples were positive after 2 h, and 2/70 (3%) were positive after 2 days. All samples were negative after 7 days [18]. A study in Germany by Schultz et al. tested air and soil samples upwind and downwind from six commercial MRSA-positive pig barns for the presence of livestock-MRSA [7]. 72% of soil samples collected 150 m downwind were positive, compared to 28% of samples collected 100 m upwind [7] (*p* = 0.0082). None of the 24 air samples collected 100 m upwind were positive, while 2/24 air samples collected 150 m and 3/23 air samples collected 50 m downwind were positive [7].

The current study has some weaknesses that must be considered when interpreting results. We had only crude information on the location of the participants, using the location of their home address as a proxy for exposure to any environmentally spreading livestock-MRSA. While we conducted analyses adjusted for age, gender, education and income level, our findings could still be affected by residual confounding, including urban-rural confounding. We did not have information on e.g. the location of participants’ work or school addresses or the amount of time they spent outdoors, and we could therefore not account for these factors. The wind data we used had low spatial resolution of 0.25 × 0.25 degrees, but because we summarized wind direction in time intervals of between 7 and 365 days, and because wind directions in Denmark are relatively uniform over such time intervals (Online Appendix S5), we deem it unlikely that results would have been substantially different had we used wind data with a higher spatial resolution. The analyses only included cases of livestock-MRSA infection where the physician notifying the SSI had not reported that the patient had any livestock contact (either directly or indirectly), but the information on livestock contact was likely incomplete, so that some cases included in the analyses did have livestock contact. In a previous study in Denmark among 223 persons who has tested positive for livestock-MRSA and were registered as having no contact to livestock, 19% did report occupational exposure to pigs in a follow-up interview [19]. However, we would not expect persons with livestock contact to live predominantly in one wind direction relative to the surrounding pig farms. Hence, underreporting of livestock contact would lead to bias towards the null in the interaction between distance to pig farms and wind direction, and underreporting cannot explain why our post-hoc analyses demonstrated that there likely was a link between wind direction and risk of having a clinical infection with livestock-MRSA. Controls were sampled from the general population without consideration of their occupation, so some controls could have had livestock contact. However, we find it unlikely that inclusion of occupationally exposed healthy individuals would lead to considerable bias in the interaction between wind direction and distance on the risk of MRSA infection.

We did not account for the spreading of pig manure on fields around the pig farms, nor for the location of fields relative to participants’ home addresses. However, an environmental study demonstrated that even though livestock-MRSA could be detected in pig manure, the spreading of pig manure on fields did not influence the degree to which field soil samples tested positive for livestock-MRSA [20]. This suggests that other factors than spreading of manure are responsible for dispersion of livestock-MRSA around pig farms, and therefore we do not think that it is a major problem for the validity of our findings that we did not account for spreading of manure and the location of fields.

Our analyses only accounted for the location of pig farms around participants’ homes, and did not account for other kinds of farm animals such as cattle, horses and mink that may be colonized by livestock-MRSA to a lesser degree than conventional pigs [3]. While we do not expect this to explain the pattern demonstrated for pig farms, in future studies it should also be considered to account for other kinds of animals.

While this study provides some evidence that wind influences the risk of infection with livestock-MRSA in humans, it does not answer the question of how such transmission takes places, e.g. whether it is through direct spread of dust containing livestock-MRSA, or through affecting the flight patterns of flies that can be colonized with livestock-MRSA [21].

The main strength of the study was that it was population-based, including all cases of a well-defined notifiable disease (clinical infection with livestock-MRSA among persons with no known contact with livestock) in all of Denmark proper. Cases were matched with a high number of controls based on age, gender and municipality of home address, and we conducted both crude and adjusted analyses. Apart from information on livestock contact, all data was extracted from public registers with a high degree of completeness, minimizing the risk of information bias. The decision to match participants based on municipality was made to remove confounding from unmeasured socio-economic factors, including the prevalence of livestock-MRSA colonization in the background population. It might be argued that matching by municipality represents over-adjustment, since some municipalities in Denmark have much higher numbers of pig farms than others [5], meaning that the matching could lead to bias towards the null in analyses of odds of infection as a function the number of pig farms nearby. However, because the present study matched participants by municipality and still clearly demonstrated that livestock-MRSA cases lived closer to pig farms than controls, it is very likely that the absence of a significant difference in distance to nearest pig farm in the three selected municipalities in Anker et al. [5] was due to a low statistical power, and not evidence of a lack of correlation between living close to pig farms and the risk of becoming infected with livestock-MRSA.

## Conclusion

While the main adjusted analysis did not show effect modification by wind direction on the relationship between proximity to pig farms and odds of clinical infection with livestock-MRSA, exploratory post-hoc analyses suggested that this may be because there is no effect modification at low distances (50 − 1,250 m), while there may be significant effect modification at intermediate distances (1,250-6,250 m). This seems to suggest that livestock-MRSA bacteria may be spreading by wind, but the results need to be confirmed in new studies.

## Supplementary Information

Below is the link to the electronic supplementary material.

Supplementary Material 1: Copy of pre-published analytical protocol [9]

Supplementary Material 2: List of deviations between analytical protocol and analyses conducted

Supplementary Material 3: Results from all pre-planned analyses

Supplementary Material 4: Results from all post-hoc secondary analyses

Supplementary Material 5: Graphical presentation of wind direction in Denmark proper in various time intervals

Supplementary Material 6: Numerical data corresponding to online supplement S4

## Data Availability

Wind data are openly available from the ERA5 database ([10.24381/cds.38b394e6](10.24381/cds.38b394e6)). Information on the location of Danish pig farms can be requested from the Central Husbandry Register ( [https://chr.fvst.dk](https://chr.fvst.dk)). Data on MRSA CC398 infection can be requested from Anders Koch ([ako@ssi.dk](mailto: ako@ssi.dk)) at Statens Serum Institut. To access data from the Central Personal Register, researchers must contact Statistics Denmark ( [https://www.dst.dk/en/TilSalg/Forskningsservice](https://www.dst.dk/en/TilSalg/Forskningsservice)).

## Abbreviations

MRSA: Methicillin-resistant Staphylococcus aureus
Livestock-MRSA: PVL-negative MRSA CC398
SSI: Statens Serum Institut
