# Supplementary material for: Influence of wind direction on the relationship between proximity to pig farms and risk of infection with MRSA CC398 among persons without known contact to livestock: a Danish nationwide population-based study
Source: Infection. 2025 Sep 8;53(6):2795–808. doi: 10.1007/s15010-025-02629-2 (PMC12675557; doi:10.1007/s15010-025-02629-2)
Supplement: Supplementary file 1 — Supplementary Material 1: Copy of pre-published analytical protocol [9] [file 15010_2025_2629_MOESM1_ESM.pdf]

Analysis protocol for the project "Distance to pig herds as related to livestock-associated MRSA CC398 infection in persons without known contact to livestock – A reanalysis accounting for wind"

Contents

1 Definitions and abbreviations ... 2
2 Introduction ... 2
3 Hypothesis ... 3
4 Design ... 3
5 PECO statement ... 3
6 Registers, variables and preprocessing ... 5
6.1 Pig herd data from the Central Husbandry Register (CHR) ... 5
6.2 Wind data from ERA5 ... 5
6.3 Demographic data from the Central Personal Register (CPR) ... 7
6.4 MRSA data from the Statens Serum Institut ... 8
7 Software tools ... 8
8 Analysis plan ... 8
8.1 Primary analysis (analysis 1) ... 8
8.1.1 Purpose ... 8
8.1.2 Relevant cases ... 8
8.1.3 Exposure model with hyperparameters ... 8
8.1.4 Analysis algorithm ... 12
8.1.5 Co-variates ... 13
8.1.6 Handling of addresses close to international borders ... 14
8.1.7 Pairwise dropping of observations ... 14
8.2 Sensitivity analyses ... 14
8.3 Overview of primary and sensitivity analyses ... 16
8.4 Secondary analyses ... 16
8.5 Descriptive statistics ... 16
9 Publication of results ... 17
10 Perspectives ... 18
11 Project group ... 18
12 Appendix 1: Prevalence of MRSA-positive pig herds ... 19
13 Appendix 2: Pseudo-code describing the algorithm for the data-driven analyses ... 22
14 References ... 24

1 Definitions and abbreviations

- AU = Aarhus University
- MRSA = methicillin-resistant Staphylococcus aureus
- Livestock-MRSA = PVL-negative MRSA CC398
- LO = Livestock-onset (MRSA) = clinical case of livestock-MRSA infection where the patient is known to have direct or indirect contact with livestock.
- MUO = Livestock-MRSA of unknown origin = clinical case of livestock-MRSA infection where the patient does not have any known contact with livestock.
- SSI = Statens Serum Institut

2 Introduction

Methicillin-resistant Staphylococcus aureus (MRSA) is a significant public health problem in Denmark, with an increasing incidence of clinical infection.[1] Livestock-MRSA is defined as MRSA CC398 that is PVL negative. The registered incidence of clinical infections with livestock-MRSA increased from 2010 to 2017, after which it has stabilized, but this may be because patients are only registered the first time they are tested positive for a specific strain of MRSA[1] (i.e., reinfections are not registered). In 2019, 1,599 Danish cases of clinical infections with MRSA were reported to Statens Serum Institut.[1] Out of the 253 (16%) cases who had livestock MRSA infection, 167 (63%) had direct or indirect contact with livestock before the diagnosis (LO), while 86 (34%) did not have any known contact to livestock (MUO).[2]

Pigs and other livestock serve as a reservoir for livestock-MRSA. In 2019, 95% of all conventional breeding pig herds were MRSA-positive, while a lower prevalence was found among horses, cattle and mink.[1] However, as described above, a considerable proportion of clinical infections with livestock-MRSA is seen among people without known contact to livestock. In 2018, Anker *et al*[3] published a paper analyzing the spatial distribution of clinical infections with livestock-MRSA in Denmark, in an attempt to determine whether the livestock-MRSA bacteria were transmitted environmentally or through person-to-person contact. The study covered all 192 cases of clinical livestock-MRSA infection without known livestock contact from 1 January 2006 to 11 February 2015, and compared the following parameters for the cases with random population controls:

- Road distance from the home address to nearest pig herd
- Road distance from the home address to nearest person with LO MRSA

An analysis of all 192 cases clearly showed that the median road distance to nearest herd was shorter for the MUO cases than for the controls. The median distance to nearest LO MRSA case was also shorter for MUO cases than for the controls. However, when the analysis was repeated based on only 15 MUO cases in a subjectively selected area (Horsens, Hedensted and Vejle municipalities), the difference appeared smaller and

was no longer statistically significant. In the words of Anker *et al*, this subgroup analysis suggested *"that for persons without livestock contact, but living within a pig-farming area, the actual distance to pigs or to nearest LO MRSA CC398 case does not increase the risk of MRSA CC398 infection."*[3]

While Anker *et al* deserve merit for attempting to determine the route of transmission of MUO MRSA, methodological shortcomings of the study mean that caution is needed when interpreting results, and we believe that a new study is warranted. By definition, LO MRSA cases must have had contact with livestock, so a high degree of covariance must be expected between distance to nearest herd and distance to nearest LO MRSA case. Because of this covariance, the two distances must have confounded each other's associations with the risk of MUO MRSA in the study by Anker *et al*. We have briefly considered if a new study could account for this using an adjusted regression model, but we expect the covariance to be so high that disentangling the effects of the two distances in this manner is infeasible with a dataset containing only a few hundred cases. Second, the lack of a statistically significant association in the subgroup analysis of 15 persons in the study by Anker *et al* did not add information compared to the main analysis, since the statistical power was low. To answer the questions *"Does livestock-MRSA – to a clinically relevant extent – spread environmentally (as opposed to only by person-to-person contact)?*", we plan to conduct a new study of the risk of MUO MRSA infection as a function of Euclidean distance to pig herds and wind direction. If the mean wind direction around the home address of a person influences the risk of becoming a MUO MRSA case, it would be a very strong indication that environmental spread by wind is taking place.

3 Hypothesis

We hypothesize that

1) The risk of becoming an MRSA CC398 MUO case increases when the number of pig herds in the vicinity increases.
2) Given the same distances to a pig herd, the risk of becoming an MRSA CC398 MUO case is higher for persons who live downwind compared to persons who live upwind from the herd.

4 Design

Register-based cross-sectional study.

5 PECO statement

5.1.1.1 Population

The cases are all persons diagnosed with a clinical infection caused by a PVL-negative strain of MRSA CC398 between 29 June 2009 and 31 December 2021, with no known direct (pig farmer) or indirect (through household members) livestock contact.

Cases are only eligible to be included in the study if the geographic location of their residential address is available in the Central Personal Register from 365 days before the date of diagnosis until (and including) the date of diagnosis. Because the same address history exclusion criterion applies to controls, who are matched to cases based on (among other things) a date of birth ±180 the date of birth of the case, we will exclude any cases who have an age below 545 days (365 + 180) at the date of diagnosis. If we did not filter out cases younger than 545 days, controls for these young cases would be systematically older than the cases, which could lead to bias in our analyses.

SSI has registered MRSA CC398 MUO cases since 1 January 2006. Data on prevalence of MRSA CC398 in Danish pig herds are only available from 2008 onwards (see page 19), and we must limit our analyses to persons where this information is available. Therefore, the starting date of the project is 545 days after January 1 2008, i.e. 29 June 2009.

5.1.1.2 Exposure

The exposure of interest is MRSA CC398 PVL-negative bacteria. Proxies for this exposure are

1) Euclidian distance (= in a straight line / as the crow flies) from home address to all pig herds with at least 10 livestock within a 25-km radius from the address.
2) Wind direction (relative to the angles between the home address and each pig herd)

Analyses in the previous article were based on road distance from home address to nearest herd,[3] but we will instead use straight-line distances, as we expect this to be a better expression of any airborne spread of MRSA CC398 from the herd. We will not consider the distance to nearest CC398 LO case in our analyses, as we expect there to be a high degree of covariance between distance to herds and distance to nearest MRSA CC398 LO case, and we will not have the statistical power to disentangle effects of the two.

5.1.1.3 Comparator

Controls will be a random sample of the Danish population, matched to controls by gender, age and municipality (*"kommune"*). Matching to age will happen by selecting persons with a date of birth within ±180 days of the date of birth of the corresponding case. Individuals who at some point in the study period become cases can only be selected as controls up to and including the date 366 days before their own diagnosis. The same individuals may be selected to serve as controls for multiple cases.

We might increase the time window for age-based matching from ±180 to e.g. ±365 days if we are otherwise unable to find appropriate controls for a considerable proportion of the cases.

We will not match based on regions ("region") because we deem that the within-region variation in demographic variables is probably so large that region-based matching is unlikely to remove much confounding. We will not match based on parishes ("sogn") because the parishes are so small that it will likely be difficult to find appropriate matches.

As for cases, potential controls will only be eligible for inclusion if the geographic location of their residential address is available in the Central Personal Register from 365 days before the date of diagnosis of the case until (and including) the date of diagnosis of the case.

To maximize statistical strength, we will sample up to 10 controls for each case. We will use sampling with replacement.

5.1.1.4 Outcome

The outcome of interest is clinical infection with a PVL-negative strain of MRSA CC398 with no "direct (pig farmer) or indirect (through household members) livestock contact."[3]

6 Registers, variables and preprocessing

6.1 Pig herd data from the Central Husbandry Register (CHR)

The Department of Environmental Science at Aarhus University will provide a database containing information on all Danish pig herds in the period 2006-2021. The data was originally provided by the Central Husbandry Register (CHR, https://chr.fvst.dk). The dataset includes number and type of pigs, time interval in which the herd existed, and coordinates of the herd.

6.2 Wind data from ERA5

Wind data have been downloaded from the ERA5 database.[4] For each hour of the day, the database provides information on the **u** and **v** component vectors that together represent the strength and direction of the wind 10 meters above the surface (see Figure 1). The spatial resolution of the database is 0.25×0.25 degrees. To save disk space and processing time, we have calculated mean u and v components in daily resolution using the following formulae:

Equation 1

$$u_{day} = ½ \times u_{h,0} + \frac{\sum_{i=1}^{23} u_{h,i}}{23} + ½ \times u_{h,24}$$

Equation 2

$$v_{day} = ½ \times v_{h,0} + \frac{\sum_{i=1}^{23} v_{h,i}}{23} + ½ \times v_{h,24}$$

Where $v_{h,0}$ is the v component of wind (in m/s) at 0 AM, and i is the hour of the day.

Figure 1: Vector representation of wind

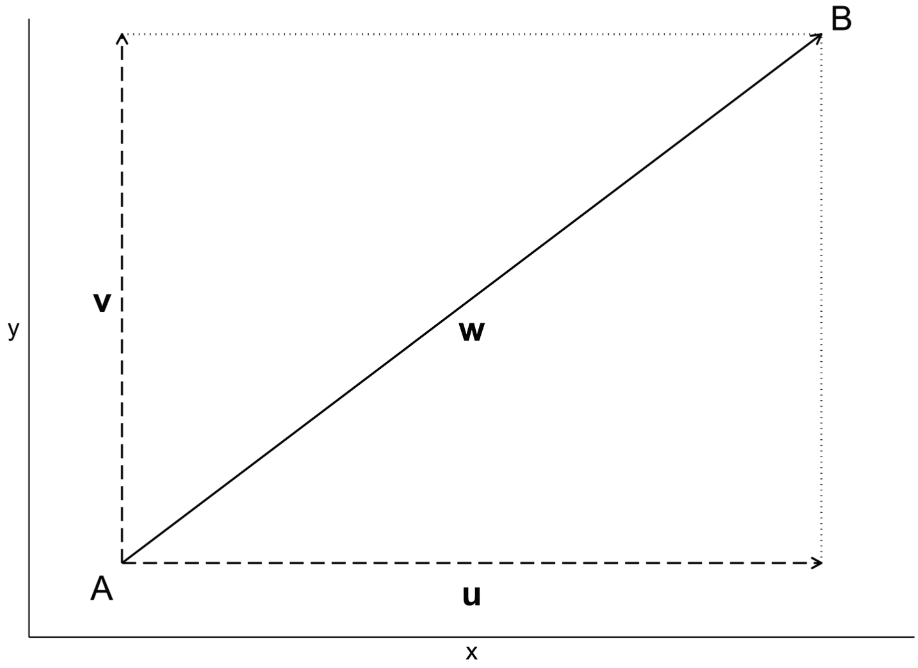

w is a vector representing the strength and direction of the wind, and **u** and **v** are component vectors along the x and y axes, respectively.

6.3 Demographic data from the Central Personal Register (CPR)

Through the National Centre for Register-based Research we have received the following data on cases and controls:

**Variable content**	**Variable name**	**Comment**
Date of birth	fdato	
Gender	kqn[5]	Defined from CPR-number (unique personal identification number used in Danish registers)
Highest attained educational level	hfaudd[6]	Categorized as less than high school, high school, higher than high school.<br>For persons with an age $\geq 25\times 365.24$ days, we use their own value. For persons with an age $<25\times 365.24$ days, we will use the highest value among their parents. In the register, the variable hfaudd is listed by year (as the highest attained educational level changes for a person as they progress through the educational system). As there is no easy way of averaging a categorical variable, we will use the value for the year that the case was diagnosed.
Unique geographic ID of home address	bopindex	1 observation per residential address per person
Date of moving in	tflytd	
Date of moving out	fflytd	
Municipality	komkod[7]	
Income level	aekvivadisp_13[8,9]	aekvivadisp_13 is the equivalised disposable income. We will calculate the percentile rank of the aekvivadisp_13 across all persons in the CPR register by year. The percentile rank is our metric for income level, as it allows comparing participants diagnosed in different years. In the many cases, the time interval over which we summarize wind data (up to 365 days before diagnosis) will span two calendar years. For each person, we will calculate a weighted average of their percentile rank (with number of days in each calendar year as the weighting factor).

6.4 MRSA data from the Statens Serum Institut

SSI has provided a dataset of MUO MRSA CC398 cases from 2007 to 2021. For each case, we have received the following information:

- CPR number (for merging with sociodemographic data from the CPR register)
- Date of diagnosis
- Reason for test ("infection", "screening", "other" or "unknown")

The primary analysis will be restricted to cases diagnosed with "infection" on or after January 1 2016. In sensitivity analyses we will also include cases diagnosed earlier, and with test reason "other" or "unknown" (see details below).

7 Software tools

Data clean-up and management will be performed using Stata 15 (StataCorp, College Station, Texas, US). Address-based geographic analyses will be carried out using Esri ArcGIS Pro (Environmental Systems Research Institute, Redlands, California, US) and Python 3.6 (Python Software Foundation, www.python.org). Statistical analyses will be performed using Stata 15.

8 Analysis plan

8.1 Primary analysis (analysis 1)

8.1.1 Purpose

To describe the relationship between the number of pig herds near an address and the risk of MUO. To investigate whether this association is modified by wind direction, and if whether such effect modification is statistically significant.

8.1.2 Relevant cases

MUO clinical cases diagnosed on or after January 1 2016. At this point in time, we expect most pig herds to be positive for MRSA CC398 (see page 19).

8.1.3 Exposure model with hyperparameters

For each case and control, we determine the straight-line distances to all pig herds within a distance of 25 kilometers from the residential address of the participant (this limit selected to make it possible to conduct the geographic analyses in finite time, as calculations may take extremely long if we include distances to all herds in the entire country). We also calculate the angle (relative to North) between the address (A) and the herd (F). The vector from each herd to the address is called FA, while the vector representing the mean wind direction in the area between the home and the herd is called W. We calculate the angle D between FA and W, see Figure 2. The time window for calculating D will be determined in a data-driven approach as described

below. D lies in the interval from -180 to 180 degrees. Our measure for wind direction is abs(D), i.e. the absolute value of D that lies in the interval from 0 to 180 degrees.

Figure 2: Definition of the angle D

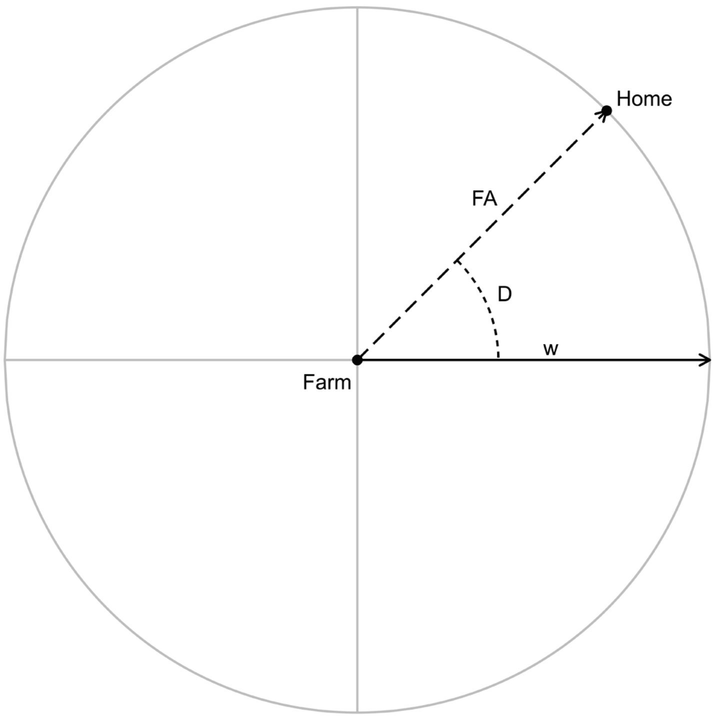

The solid black vector w represents the wind direction. The dashed vector FA goes from the herd to the home address.

As described above, mean wind will be summarized in a grid of 0.25×0.25 degree cells covering Denmark. Some home addresses may be located in a different grid cell than the nearby herds. If FA passes through more than one grid cell, we will calculate a value of D in each grid cell, and use the mean value (calculated using *y* and *x* vectors, weighted by the length of the FA vector located in each grid cell). This is illustrated in Figure 3, where the black dots represent two geographic locations in Denmark, and the white dots represent each place the vector will be split because it crosses into a new grid cell.

Figure 3: How to handle herd-address vectors crossing more than one cell in the wind grid

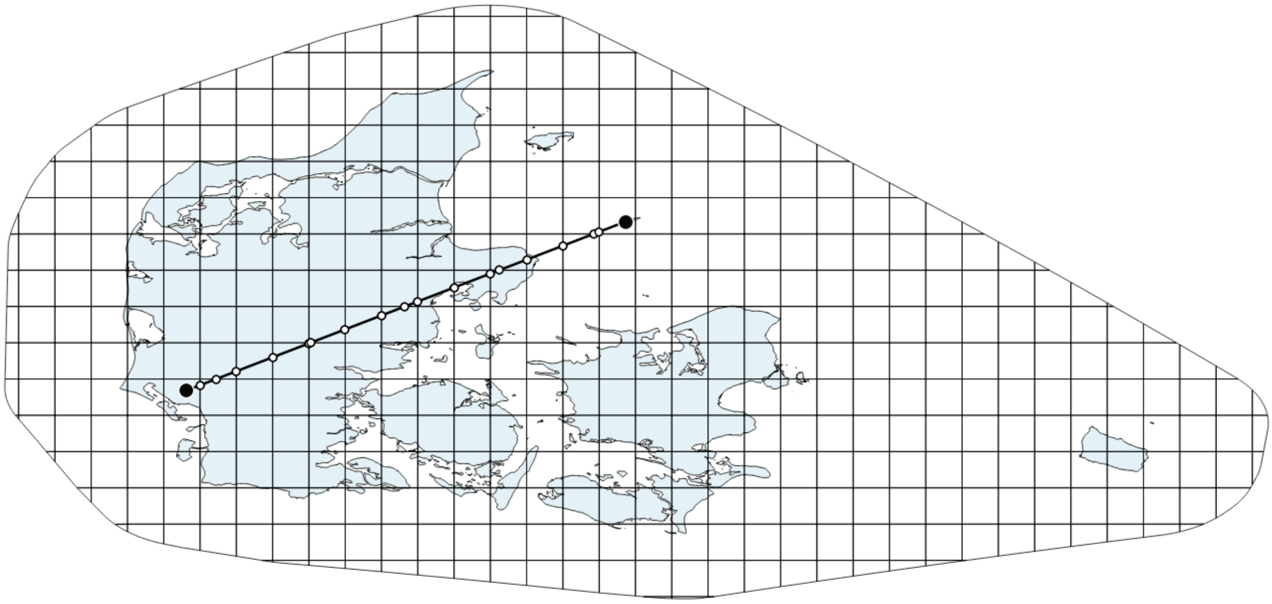

For illustration purposes, a very long vector has been shown. However, we will only perform analyses for herd-address vectors with a maximum length of 25 km.

A priori, we expect that the odds of MUO MRSA increases when the number of pig herds close to the home address increases, and that odds are higher when the herds are upwind relative to the address (during the days critical for infection) - i.e. odds are higher for abs(D) close to 0 degrees than for abs(D) close to 180 degrees. We need to define an exposure metric that takes this into account. Furthermore, we need to account for all herds close to the home address, not just the nearest herd (because a home address may be located close to multiple herds at approximately the same distance, but in different directions).

In Equation 3, we have defined a simple exposure metric x_1 that accounts for the number of herds within a given radius from an address. c_i is the distance to the ith herd, and p is a constant. p must be ≥ 0, since a value of p < 0 would imply that herds further away are more important than herds closer to an address. Note that x_1 as defined by Equation 3 does not account for the wind.

Equation 3

$$x_1 = \sum_i c_i^{-p}$$

We now introduce a weighting factor called h_i to account for the wind, as shown in Equation 4.

Equation 4

$$x_2 = \sum_i h_i \times c_i^{-p}$$

h_i will be defined as shown in Equation 5, where h_{dw} is the exposure from a pig herd located directly downwind from the home address (i.e., $abs(D_i) = 180°$) relative to the exposure from a pig herd located directly upwind from the home address (i.e., $abs(D_i) = 0°$).

Equation 5

$$h_i = h_{dw} + (1 - h_{dw}) \times \frac{\cos\left(abs(D_i)\right) + 1}{2}$$

For an illustration of how h_i changes as a function of $abs(D_i)$, see Figure 4 where graphs for h_i have been drawn for a number of different values of h_{dw}.

Figure 4

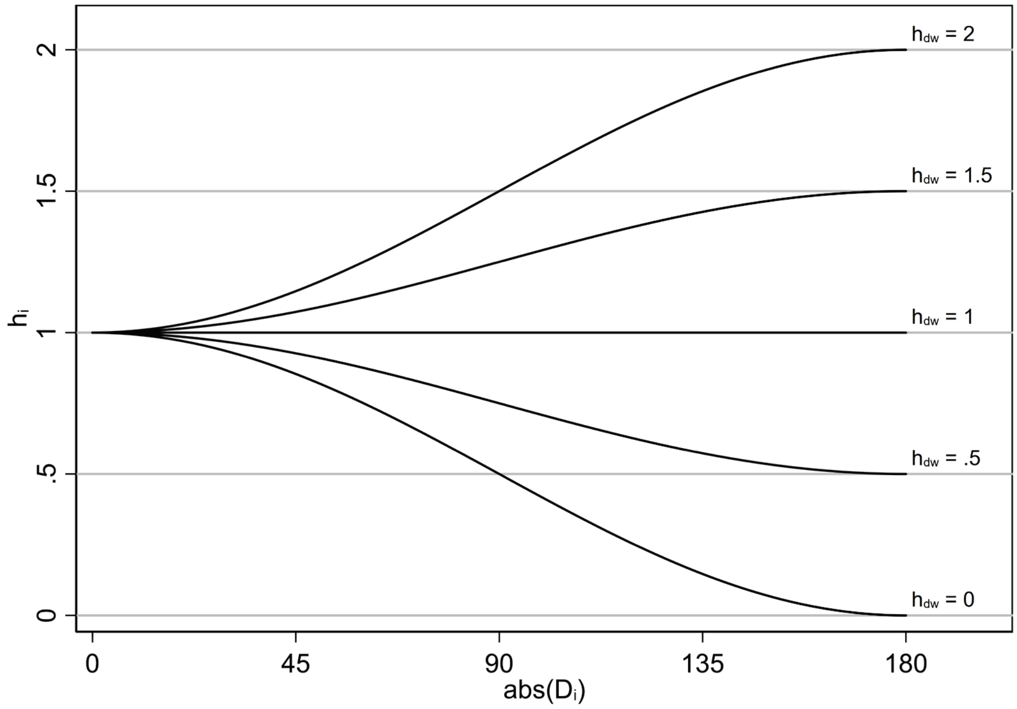

Combining Equation 4 and Equation 5, we get our final exposure metric x_2 as given by Equation 6.

Equation 6

$$x_2 = \sum_i \left(h_{dw} + (1 - h_{dw}) \times \frac{\cos\left(abs(D_i)\right) + 1}{2} \right) \times {c_i}^{-p}$$

Equation 6 explicitly contains the unknown constants h_{dw} and *p*, and we must also decide on the relevant time window over which to summarize abs(D), and the search radius for herds (we have performed calculations up to a distance of 25 km, but we do not know it the relevant distance is e.g. up to 10 km). Table 1 describes the possible ranges of these hyperparameters.

Table 1: Hyperparameters for the calculation of x_2

**Parameter**	**Range of values to be evaluated**	**Comment**
p	[0,3]	No point in evaluating p < 0, as this would imply higher exposure from herds further away.
h_{dw}	[0,2]	No point in evaluating $h_{dw} < 0$, since the exposure from a herd must be non-negative. *A priori*, we expect the optimum h_{dw} to lie in the interval [0,1], but $h_{dw} > 1$ are included in analyses to allow calculation of symmetric confidence intervals, if the optimum h_{dw} is around 1.
Time window before MUO diagnosis over which to summarize abs(D)	7 days, 14 days, 21 days, 30 days, 90 days, 180 days, 270 days, 365 days	
Search radius	[50m; 25000m]	The interval starts at 50 m. MUO cases and controls who have lived closer than 50 meters to a pig herd in the last 365 days before diagnosis of the case will be deleted from the dataset, as persons living so close to a herd are assumed to have animal contact (even if such contact is not registered in the SSI database).

8.1.4 Analysis algorithm

The idea behind our statistical analysis is to determine whether an exposure model that accounts for wind direction on a daily basis (i.e., has $h_{dw} \neq 1$) is better at predicting MUO status (positive/negative) than a model that is agnostic to wind direction.

As described in section "8.1.3 Exposure model with hyperparameters", apart from h_{dw} the exposure model contains a number of other hyperparameters that cannot be estimated using standard regression methods (namely p, time window over which to summarize wind, and search radius). Before we can evaluate how different values of h_{dw} influence model performance, we need to determine the optimum combination of these other hyperparameters. This will be done by performing a random hyperparameter search.[10] We will conduct a large number of analyses with different random combinations of the other hyperparameters, and all possible values of h_{dw}. In each analysis, we will conduct a logistic regression between x_2 and odds of MUO (without any other covariates), and quantify the level of agreement between the model's predictions of case

status and the observed case status using Cohen's kappa.[11] The one combination of hyperparameters that gives the highest kappa will be the one used in the following steps.[*] If two or more combinations of hyperparameters have the same kappa, we will choose one of the combinations at random using a pseudo-random number generator.

Next, we will calculate the h_{dw} that has the highest kappa using a grid-based hyperparameter search[10] – in other words, we will calculate kappa for each of the possible values of h_{dw} and select the one with the highest kappa. Its 95% confidence interval will be calculated using a bootstrap procedure. If the best h_{dw} is statistically significantly lower than 1, it will imply that wind direction modifies the levels of exposure from pig herds. In the bootstrap procedure, we need to account for the possibility that two or more values of h_{dw} could result in the same maximum value of kappa. In such cases, we will chose one of the best h_{dw} values using a pseudo-random number generator.

For each of the possible values of h_{dw}, we will also calculate the difference in kappa between a model with this h_{dw} and a model with $h_{dw} = 1$ (this will be termed λ). We will create line plots of kappa and λ with 95% CI as a function of h_{dw} to examine if the model performance at different values of h_{dw} is statistically significantly better than model performance with $h_{dw} = 1$.

The steps involved in the analysis algorithm are presented in detail as pseudocode in "Appendix 2: Pseudo-code describing the algorithm for the data-driven analyses" on page 22.

8.1.5 Co-variates

We will perform both unadjusted and adjusted versions of the above analysis. The adjusted version will be adjusted for age, gender, and socioeconomic status (education and income). While controls will be matched to cases based on age and municipality of residence, the matching alone is deemed insufficient to avoid confounding. Matching will ensure that age and municipality are marginally independent of disease status, but they may not be conditionally independent of disease status within strata of wind direction and distance. We will have an insufficient number of cases to formally adjust for municipality of residence (other than by matching).

---

[*] In principle, we could also determine the optimum combination of other hyperparameters separately for each value of h_{dw}. However, it could be problematic to use different exposure time intervals and search radii for different values of h_{dw}. As described in section 6.1.6, we will exclude participants who have lived too close to an international border in the time interval summarized. If different h_{dw} are combined with different time intervals and search radii, the number of participants will be different between analyses based on different h_{dw}, and this could be enough to cause differences in kappa. Our results would therefore become difficult to interpret: Are differences in kappa due to different n, or do they indicate an effect of wind?

To account for non-linear relationships with log(odds) of MUO MRSA, we will model age and income using restricted cubic splines with four knots. The location of the knots will be determined by the distribution of the variables.[12]

8.1.6 Handling of addresses close to international borders

The CHR database of pig herds only covers Denmark proper and not neighboring countries. Any potentially windborne MRSA will not respect political borders. Hence, in analyses with a search radius of s and exposure time window of t days, we will exclude any participant who has lived closer than s meters from the nearest point on land in another country within the last t days before the date of diagnosis. Exposure levels for such participants will be systematically underestimated, and failure to exclude such participants could lead to bias in our statistical analyses. We will not exclude participants who live closer than s meters to a sea border, if the closest point on land in another country is still s or more meters away.

8.1.7 Pairwise dropping of observations

If we exclude all controls for a particular case (e.g., because they live close to the border, or in case of adjusted analyses, because they miss information on covariates), we will also exclude the case. If we exclude the case, we will also exclude all the controls.

8.2 Sensitivity analyses

To investigate the robustness of our findings, we will conduct a number of sensitivity analyses.

In analysis 2, h_i is defined using a linear function described in Equation 7 and illustrated in Figure 5. The interpretation of h_{dw} will be the same as in analysis 1, but some readers may find it easier to understand the linear model for h_i.

Equation 7

$$h_i = 1 - (1 - h_{dw}) \times \frac{abs(D_i)}{180°}$$

Figure 5

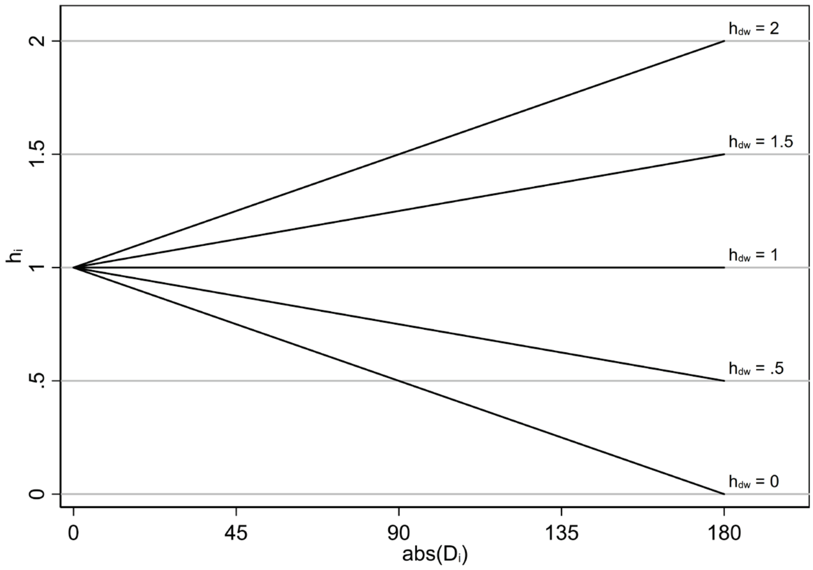

In analysis 3, we will weight the exposure from each pig herd by the number of animals in the herd.

In analysis 4, we will include cases diagnosed in 2009-2021 instead of only 2016-2021, and weight the exposure from each herd by the proportion of pig herds in Denmark that were MRSA CC398 positive in the year of exposure (see "Appendix 1: Prevalence of MRSA-positive pig herds").

In analysis 5, we will combine the approaches from analysis 3 and analysis 4.

In all the previous analyses, we will only include cases MUO cases with a reason for testing listed as "infection" in the SSI registers. In analysis 6, we will also include MUO cases with the reason listed as "other" or "unknown". As we expect considerable biases in who gets screened for MRSA, we will not include cases where the reason is "screening".

In analysis 7, we will not exclude persons living closer than 50 meters from closest pig herd.

In analysis 8, we will conduct hyperparameter optimization and evaluate the model using Brier score[13] instead of Cohen's kappa.

8.3 Overview of primary and sensitivity analyses

An overview of the differences between the primary and sensitivity analyses is provided in Table 2.

Table 2: Overview of analyses

Analysis	*Definition of h_i*	*Time from which cases are included*	*Exposure from each herd weighted by…*		*Reason for testing case*	*Persons excluded if < 50 meters from pig herd*	*Evaluation metric for model*
			Number of animals	*Prevalence of MRSA*			
1	Equation 5	2016-2021	No	No	Infection	Yes	Kappa
2	Equation 7	2016-2021	No	No	Infection	Yes	Kappa
3	Equation 5	2016-2021	Yes	No	Infection	Yes	Kappa
4	Equation 5	2009-2021	No	Yes	Infection	Yes	Kappa
5	Equation 5	2009-2021	Yes	Yes	Infection	Yes	Kappa
6	Equation 5	2016-2021	No	No	Infection, unknown, or "other"	Yes	Kappa
7	Equation 5	2016-2021	No	No	Infection	No	Kappa
1	Equation 5	2016-2021	No	No	Infection	Yes	Brier score

8.4 Secondary analyses

For each of the models in the hyperparameter optimization conducted for each of the analyses (see Table 2 and Appendix 2), we will save the kappa (or Brier score) for the h_{dw} with the highest kappa (or Brier score), along with the corresponding values of p, time interval and search radius. For each analysis, we will then create plot of kappa (or Brier score) as a function of p, time interval, or search radius, respectively.

8.5 Descriptive statistics

For each of the analyses in Table 2, we will create a table of demographics with a structure as shown in Table 3.

Table 3: Example table of descriptive statistics

**Parameter**		**Cases**	**Controls**	**Combined**
Number of participants		n	n	n
Gender	Female	n (%)	n (%)	n (%)
	Male	n (%)	n (%)	n (%)
Age		Mean (SD)	Mean (SD)	Mean (SD)
Income percentile	Summary	Mean (SD)	Mean (SD)	Mean (SD)
	Missing	n (%)	n (%)	n (%)
Educational level	Less than high school	n (%)	n (%)	n (%)
	High school	n (%)	n (%)	n (%)
	Higher than high school	n (%)	n (%)	n (%)
	Missing	n (%)	n (%)	n (%)
Distance to nearest pig herd	Summary (for persons with distance ≤ 25000 m)	Mean (SD)	Mean (SD)	Mean (SD)
	Number of persons with distance > 25000 m	n (%)	n (%)	n (%)
Number of herds within the specified search radius	Any $abs(D_i)$	Mean (SD)	Mean (SD)	Mean (SD)
	$0 \leq abs(D_i) \leq 45°$	Mean (SD)	Mean (SD)	Mean (SD)
	$45 < abs(D_i) \leq 90°$	Mean (SD)	Mean (SD)	Mean (SD)
	$90 < abs(D_i) \leq 135°$	Mean (SD)	Mean (SD)	Mean (SD)
	$135 < abs(D_i) \leq 180°$	Mean (SD)	Mean (SD)	Mean (SD)
Number of pigs within the specified search radius	Any $abs(D_i)$	Mean (SD)	Mean (SD)	Mean (SD)
	$0 \leq abs(D_i) \leq 45°$	Mean (SD)	Mean (SD)	Mean (SD)
	$45 < abs(D_i) \leq 90°$	Mean (SD)	Mean (SD)	Mean (SD)
	$90 < abs(D_i) \leq 135°$	Mean (SD)	Mean (SD)	Mean (SD)
	$135 < abs(D_i) \leq 180°$	Mean (SD)	Mean (SD)	Mean (SD)

9 Publication of results

Results will be submitted for publication in the international peer-reviewed journal "Zoonoses and Public Health", ISSN 1863-2378, https://onlinelibrary.wiley.com/journal/18632378 (or another international peer-reviewed medical journal).

10 Perspectives

If the results show that the risk of MUO is influenced by wind, it opens new perspectives for the prevention of the spread of the bacteria.

11 Project group

Martin Rune Hassan Hansen[1,2,3], Jörg Schullehner[1,4], Torben Sigsgaard[1], Øyvind Omland[5], Vivi Schlünssen[1,2], Anders Rhod Larsen[5], Anders Koch[5], Robert Leo Skov[5], Tinna Ravnholt Urth[6], Camilla Holten Møller[5], Steen Gyldenkærne[6], Lise Marie Frohn[6]

1) Research Unit for Environment, Work and Health, Department of Public Health, Aarhus University
   Address: Bartholins Allé 2, Building 1260, DK-8000 Aarhus C, Denmark
2) The National Research Center for the Working Environment
   Adress: Lersø Parkallé 105, DK-2100 København Ø, Denmark
3) Department of Infectious Diseases, Aarhus University Hospital
   Address: Palle Juul-Jensens Boulevard 99, DK-8200 Aarhus N, Denmark
4) Geological Survey of Denmark and Greenland
   Address: C. F. Møllers Allé 8, DK-8000 Aarhus C, Denmark
5) Statens Serum Institut
   Address: Artillerivej 5, 2300 København, Denmark
6) Department of Environmental Science, Aarhus University
   Address: Frederiksborgvej 399, 4000 Roskilde

12 Appendix 1: Prevalence of MRSA-positive pig herds

From 2008 to 2019, several surveys have been conducted in Danish pig herds to assess the proportion of herds that were positive for MRSA CC398. The surveys are summarized in Table 4.

Table 4: Nation-wide surveys of MRSA CC398 prevalence in Danish pig herds

Year	Type of herd	Number of herds (n)		% MRSA CC398 +	Source
		Investigated	MRSA CC398 +		
2008	Breeding	95	0	0	EFSA[14]
2008	Slaughter	198	Not reported	3.5	
2009	Slaughter	99	Not reported	16	DANMAP[15]
2011	Slaughter	79	13	16	DANMAP[16]
2014	Breeding	70	Not reported	63	DANMAP[17]
2014	Slaughter	205	Not reported	68	
2015	Organic	64	4	6	DANMAP[18]
2016	Slaughter	57	Not reported	88	DANMAP[19]
2018	Slaughter	130	116	89	DANMAP[20]
2018	Breeding	41	34	83	
2018	Organic/free-range	104	21	20	
2019	Breeding	73	69	95	DANMAP[1]

Using the data from Table 4, we have assessed the proportion of herd that were MRSA positive by year, In three categories of pig herds (organic/free-range, conventional breeding, conventional slaughter). Results are shown numerically in Table 6 and graphically in Figure 6, Figure 7 and Figure 8. We have generally used linear interpolation to assess the proportion in years with no surveys. In the years following the last year with a survey for a specific category of pig herd, we have used the proportion from the last survey conducted in that category.

The CHR does not contain information on whether pig herds are organic or conventional. However, as shown in Table 5, the majority of Danish pig herds are conventional. In our analyses weighted by MRSA prevalence we will assume that all herds are conventional.

When coding pig herds as breeding or slaughter herds, we will assume that all herds listed in the CHR with a non-zero number of sows are breeding herds.

Table 5: Percentage of Danish pig herds that are organic

Year	Number of pig herds		Percent organic herds
	Total[21]	Organic[22]	
2010	5,068	136	2.7%
2020	2,921	254	8.7%
2021	2,576	177	6.9%
2022	2,399	165	6.9%

Table 6: Interpolated estimates of proportion of pig herds with MRSA CC398 by year

Herd type	Year	Number of herds (n)		Proportion of herds MRSA CC398 +		
		Investigated	MRSA CC398 +	Estimate	95% confidence interval	
					Lower limit	Upper limit
Organic or free-range	2015	64	4	0.063	0.017	0.152
	2016	-		0.109	-	
	2017			0.155		
	2018	104	21	0.202	0.130	0.292
	2019	-		0.202	-	
	2020			0.202		
	2021			0.202		
Conventional breeding	2008	95	0	0.000	0.000	0.038
	2009	-		0.105	-	
	2010			0.210		
	2011			0.314		
	2012			0.419		
	2013			0.524		
	2014	70	44	0.629	0.505	0.741
	2015	-		0.679	-	
	2016			0.729		
	2017			0.779		
	2018	41	34	0.829	0.679	0.928
	2019	73	69	0.945	0.866	0.985
	2020	-		0.945	-	
	2021			0.945		
Conventional slaughter	2008	198	7	0.035	0.014	0.071
	2009	99	16	0.162	0.095	0.249
	2010	-		0.163	-	
	2011	79	13	0.165	0.091	0.265
	2012	-		0.336	-	
	2013			0.507		
	2014	205	139	0.678	0.609	0.741
	2015	-		0.778	-	
	2016	57	50	0.877	0.763	0.949
	2017	-		0.885	-	
	2018	130	116	0.892	0.826	0.940
	2019	-		0.892	-	
	2020			0.892		
	2021			0.892		

Figure 6: Estimated proportion of organic/free-range pig herds with MRSA CC398

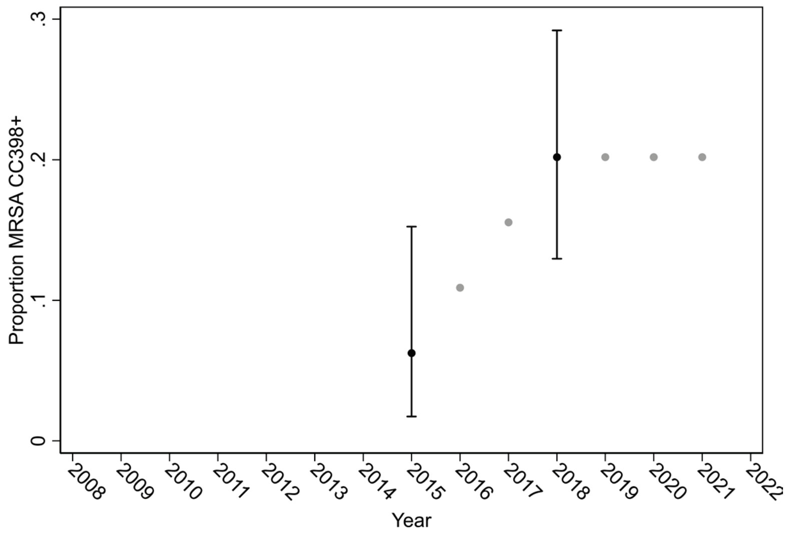

Figure 7: Estimated proportion of conventional breeding pig herds with MRSA CC398

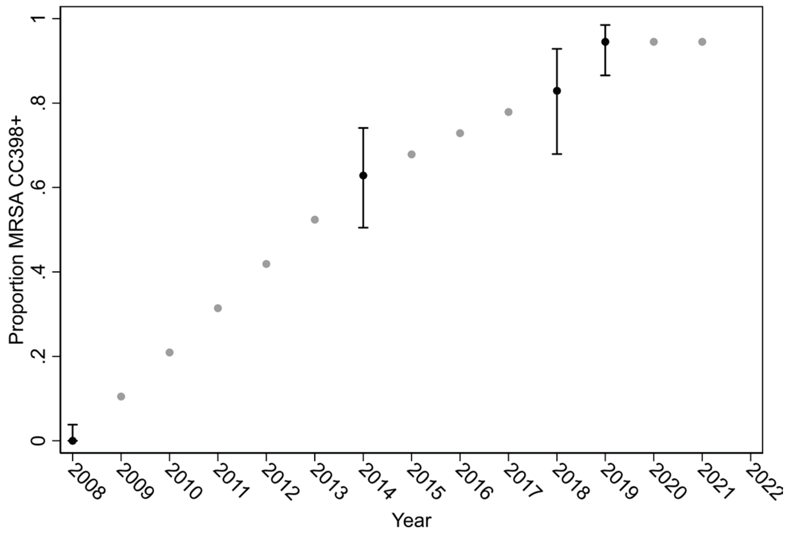

Figure 8: Estimated proportion of conventional slaughter pig herds with MRSA CC398

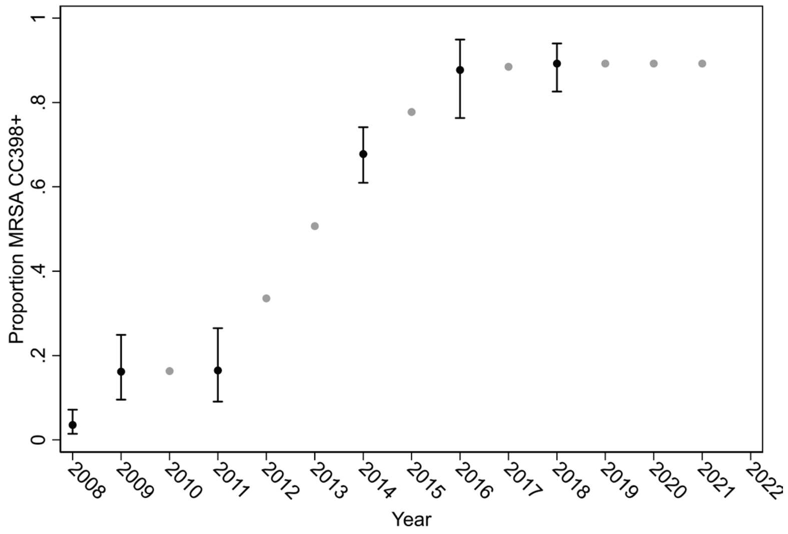

13 Appendix 2: Pseudo-code describing the algorithm for the data-driven analyses

See section "8.1.4 Analysis algorithm" on page 12 for an introduction to the algorithm. In the following, red lines prefixed with # are comments that describe blocks of pseudocode.

Pseudocode 1

	# Save the dataset to analyze		
1	Save dataset PARTICIPANT_DATA with data on cases and controls		
2			
3	# Perform random search to determine the optimum hyperparameters for our exposure metric		
4	Save empty dataset RANDOM_HYPERPARAMETER_RESULTS		
5	Do the following 1,000 times:		
6		Draw a single pseudo-random value each of p, time window and search radius from the possible ranges in Table 1	
		Load dataset PARTICIPANT_DATA	
7		For each possible value of h_{dw} in the interval [0,2] with a step size of 0.01:	
8			Calculate x_2 for each participant given these hyperparameters and current h_{dw}
9			Conduct logistic regression between x_2 and log(odds) of MUO
10			Calculate observed percentage of MUO cases among the observations in the regression (f)
11			For each observation, predict probability of being MUO according to the logistic model (g)
12			Calculate a cutoff c equal to the fth percentile of g
13			Classify each observation as expected case if they have g ≥ c, and expected control if they have g < c.
14			Calculate Cohen's kappa[11] for the degree of agreement between the observed and expected case status
15			Record kappa, h_{dw} and hyperparameters for this model in dataset RANDOM_HYPERPARAMETER_RESULTS
16	Load file RANDOM_HYPERPARAMETER_RESULTS		
17	Extract the value of p, time window and search radius from the row with the highest listed kappa. These values will be used in the remaining steps of the algorithm. Ignore what was the h_{dw} of the model with the highest kappa.		
18			
19	# Run 1,000 bootstrapped analyses, in each of which we determine the kappa for each of the possible values of h_{dw}		
20	Create empty dataset REGRESSION_RESULTS_COMBINED		
21	Do the following 1,001 times:		
22		Create empty dataset REGRESSION_RESULTS_ ONESAMPLE	
23		Load dataset PARTICIPANT_DATA	
24		IF this is not the first iteration of the loop:	
25			Draw bootstrap sample of observations (i.e., take a sample with the same number of observations with replacement)
26		For each possible value of h_{dw} in the interval [0,2] with a step size of 0.01:	
27			Repeat steps 8-14
28			Record kappa, h_{dw} and hyperparameters for this model in dataset REGRESSION_RESULTS_ONESAMPLE
29		Load dataset REGRESSION_RESULTS_ ONESAMPLE	
30		Get the kappa for the model that has $h_{dw} = 1$ and let this be termed k_1	
31		For each possible value of h_{dw} in the interval [0,2] with a step size of 0.01:	
32			Calculate $\lambda = k - k_1$ (where k is the kappa for the model with the current h_{dw})

		Add the information on λ to the file REGRESSION_RESULTS_ONESAMPLE
	Append the dataset REGRESSION_RESULTS_ONESAMPLE to the dataset REGRESSION_RESULTS_COMBINED	
# Determine the best h_{dw} (in terms of highest kappa) with 95% confidence interval (the latter derived from the bootstrapped analyses)		
Load file REGRESSION_RESULTS_COMBINED		
Define $h_{dw,best}$ as the h_{dw} of the model based on non-bootstrapped data, that has the highest kappa		
For each of the 1,000 bootstrap samples:		
	Get the h_{dw} of the model with the highest kappa for this bootstrap sample	
Calculate the bias-corrected[23] 95% CI for $h_{dw,best}$ using the estimates of best h_{dw} from individual bootstrap samples		
Report the $h_{dw,best}$ with its 95% CI (this is our main measure of whether wind influences the risk of MUO MRSA, which will be the case if $h_{dw,best}$ is statistically significantly different from 1)		
# Create plot of kappa with 95% confidence interval (the latter derived from the bootstrapped analyses) as a function of h_{dw}		
Load file REGRESSION_RESULTS_COMBINED		
Only keep the variables h_{dw}, kappa and λ for the non-bootstrapped data		
Save dataset PLOT_DATA		
For each possible value of h_{dw} in the interval [0,2] with a step size of 0.01:		
	Load file OPTIMUM_HYPERPARAMETERS	
	Only keep observations with the currently processed h_{dw}, and based on bootstrapped data	
	Only keep the variables h_{dw}, kappa and λ.	
	Calculate the bias-corrected[23] 95% CI for kappa and λ for the currently processed h_{dw}	
	Add the information on 95% CI for kappa and λ to the file PLOT_DATA	
Load file PLOT_DATA		
Foreach of the two metrics kappa and λ:		
	Create line graph of the metric with 95% CI as a function of h_{dw}	

14 References

1 Korsgaard H, Ellis-Iversen J, Sönksen UW, Skovgaard S. DANMAP 2019 - Use of antimicrobial agents and occurrence of antimicrobial resistance in bacteria from food animals, food and humans in Denmark. 2020. https://www.danmap.org/reports/2019

2 Statens Serum Institut. MRSA - opgørelse over sygdomsforekomst 2018 2019. https://www.ssi.dk/sygdomme-beredskab-og-forskning/sygdomsovervaagning/m/mrsa-2018

3 Anker JCH, Koch A, Ethelberg S, Molbak K, Larsen J, Jepsen MR. Distance to pig farms as risk factor for community-onset livestock-associated MRSA CC398 infection in persons without known contact to pig farms-A nationwide study. *Zoonoses Public Health* 2018;65:352-360. https://dx.doi.org/10.1111/zph.12441

4 Schimanke S, Ridal M, Le Moigne P et al. CERRA sub-daily regional reanalysis data for Europe on model levels from 1984 to present. https://doi.org/10.24381/cds.38b394e6, accessed 2024-01-26.

5 Danmarks Statistik. KOEN. https://www.dst.dk/da/TilSalg/Forskningsservice/Dokumentation/hoejkvalitetsvariable/folketal/koen, accessed 2024-02-01.

6 Danmarks Statistik. HFAUDD. https://www.dst.dk/da/TilSalg/Forskningsservice/Dokumentation/hoejkvalitetsvariable/hoejst-fuldfoerte-uddannelse/hfaudd, accessed 2024-02-01.

7 Danmarks Statistik. KOM. https://www.dst.dk/da/Statistik/dokumentation/Times/moduldata-for-befolkning-og-valg/kom, accessed 2024-02-01.

8 Danmarks Statistik. AEKVIVADISP_13. https://www.dst.dk/da/Statistik/dokumentation/Times/personindkomst/aekvivadisp-13, accessed 2024-02-01.

9 Statistics Denmark. Income Statistics: Statistical presentation. https://www.dst.dk/en/Statistik/dokumentation/documentationofstatistics/income-statistics/statistical-presentation, accessed 2024-02-01.

10 Bergstra J, Bengio Y. Random search for hyper-parameter optimization. *Journal of machine learning research* 2012;13.

11 McHugh ML. Interrater reliability: the kappa statistic. *Biochem Med (Zagreb)* 2012;22:276-82.

12 Harrell FE. *Regression Modeling Strategies : With Applications to Linear Models, Logistic Regression, and Survival Analysis*. New York, UNITED STATES: Springer New York, 2001. http://ebookcentral.proquest.com/lib/asb/detail.action?docID=3085295

13 Brier GW. Verification of forecasts expressed in terms of probability. *Monthly Weather Review* 1950;78:1-3. https://dx.doi.org/https://doi.org/10.1175/1520-0493(1950)078<0001:VOFEIT>2.0.CO;2

14 European Food Safety Authority. Analysis of the baseline survey on the prevalence of methicillin-resistant Staphylococcus aureus (MRSA) in holdings with breeding pigs, in the EU, 2008-Part A: MRSA prevalence estimates. *EFSA Journal* 2009;7:1376. https://dx.doi.org/10.2903/j.efsa.2009.1376

15 Korsgaard H, Agersø Y, Hammerum AM, Skjøt-Rasmussen L. DANMAP 2010 - Use of antimicrobial agents and occurrence of antimicrobial resistance in bacteria from food animals, food and humans in Denmark 2011. https://www.danmap.org/-/media/sites/danmap/downloads/reports/1996-2010/danmap_2010.pdf

16 Korsgaard H, Agersø Y, Hammerum AM, Skjøt-Rasmussen L. DANMAP 2011 - Use of antimicrobial agents and occurrence of antimicrobial resistance in bacteria from food animals, food and humans in Denmark 2012. https://www.danmap.org/-/media/sites/danmap/downloads/reports/2011/danmap_2011.pdf

17 Borck Høg B, Korsgaard H, Sönksen UW, Hammerum AM. DANMAP 2014 - Use of antimicrobial agents and occurrence of antimicrobial resistance in bacteria from food animals, food and humans in Denmark 2015. https://www.danmap.org/-/media/sites/danmap/downloads/reports/2014/danmap_2014.pdf

18 Borck Høg B, Korsgaard H, Sönksen UW. DANMAP 2015 - Use of antimicrobial agents and occurrence of antimicrobial resistance in bacteria from food animals, food and humans in Denmark 2016. https://www.danmap.org/-/media/sites/danmap/downloads/reports/2015/danmap-2015.pdf

19 Borck Høg B, Sönksen UW. DANMAP 2016 - Use of antimicrobial agents and occurrence of antimicrobial resistance in bacteria from food animals, food and humans in Denmark 2017. https://www.danmap.org/-/media/arkiv/projekt-sites/danmap/danmap-reports/danmap-2016/danmap_2016_web.pdf?la=en

20 Borck Høg B, Ellis-Iversen J, Sönksen UW, Korsgaard H, Henius AE, Pedersen KSS. DANMAP 2018 - Use of antimicrobial agents and occurrence of antimicrobial resistance in bacteria from food animals, food and humans in Denmark 2019. https://www.danmap.org/-/media/sites/danmap/downloads/reports/2018/danmap_2018.pdf

21 Danmarks Statistik. HDYR1: Landbrug med dyr efter areal, enhed og art. https://www.statistikbanken.dk/HDYR1, accessed 2024-01-30.

22 Danmarks Statistik. HDYROEKO: Økologiske landbrug med udvalgte dyr efter enhed og art https://www.statistikbanken.dk/HDYROEKO, accessed 2024-01-30.

23 Puth M-T, Neuhäuser M, Ruxton GD. On the variety of methods for calculating confidence intervals by bootstrapping. *Journal of Animal Ecology* 2015;84:892-897. https://dx.doi.org/https://doi.org/10.1111/1365-2656.12382
