# Supplementary material for: Influence of wind direction on the relationship between proximity to pig farms and risk of infection with MRSA CC398 among persons without known contact to livestock: a Danish nationwide population-based study
Source: Infection. 2025 Sep 8;53(6):2795–808. doi: 10.1007/s15010-025-02629-2 (PMC12675557; doi:10.1007/s15010-025-02629-2)
Supplement: Supplementary file 2 — Supplementary Material 2: List of deviations between analytical protocol and analyses conducted [file 15010_2025_2629_MOESM2_ESM.pdf]

Online supplement S2: List of deviations between analysis protocol and analyses conducted

Change to analysis 7

On page 15 of the analysis protocol, we described that "In analysis 7, we will not exclude persons living closer than 50 meters from closest pig herd."

When the analyses were being implemented in Stata, we realized that in all analyses, we had to exclude persons living exactly at a location where a pig herd was also located. Otherwise, such persons would have an infinitely high exposure level, due to the way our exposure metric e_{total} was defined:

$$e_{total} = \sum_i h_i \times c_i^{-p}$$

Where h_i is a weighting factor accounting for the wind direction in the area between the herd and the home address, p is a positive constant, and c_i is the distance between the herd and the address.

To avoid this issue, in the final version of analysis number 7, we excluded persons who had lived closer than 10 meters from the closest pig herd.

Change to algorithm

In the algorithm described on page 22 of the analysis protocol, code line 13 read as follows:

"Classify each observation as expected case if they have $g \geq c$, and expected control if they have $g < c$."

In the implemented algorithm, the pseudocode line would instead be:

"Classify each observation as expected case if they have $g > c$, and expected control if they have $g \leq c$."

This change was made because some models did not have any observations with $g < c$, meaning that all observations were classified as cases, with no controls.
