# Supplementary material for: Influence of wind direction on the relationship between proximity to pig farms and risk of infection with MRSA CC398 among persons without known contact to livestock: a Danish nationwide population-based study
Source: Infection. 2025 Sep 8;53(6):2795–808. doi: 10.1007/s15010-025-02629-2 (PMC12675557; doi:10.1007/s15010-025-02629-2)
Supplement: Supplementary file 3 — Supplementary Material 3: Results from all pre-planned analyses [file 15010_2025_2629_MOESM3_ESM.pdf]

Analysis number 1

Primary results from this analysis

Crude analysis

Cohen's kappa as a function of h_{dw}

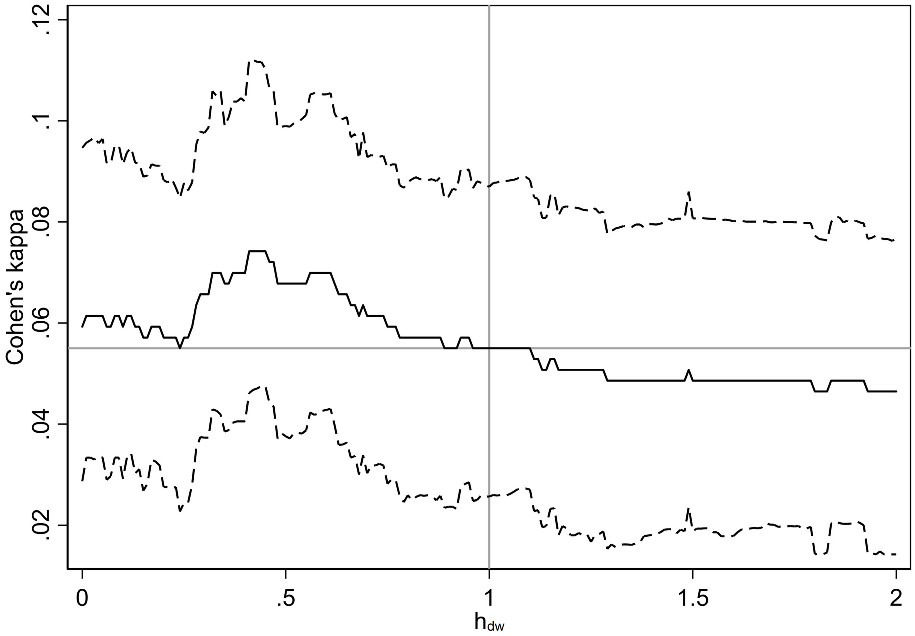

Controls matched to cases by age, gender and municipality of recidence.
Solid line is estimate, dashed lines are 95% confidence interval.

Δ(Cohen's kappa) as a function of h_{dw}

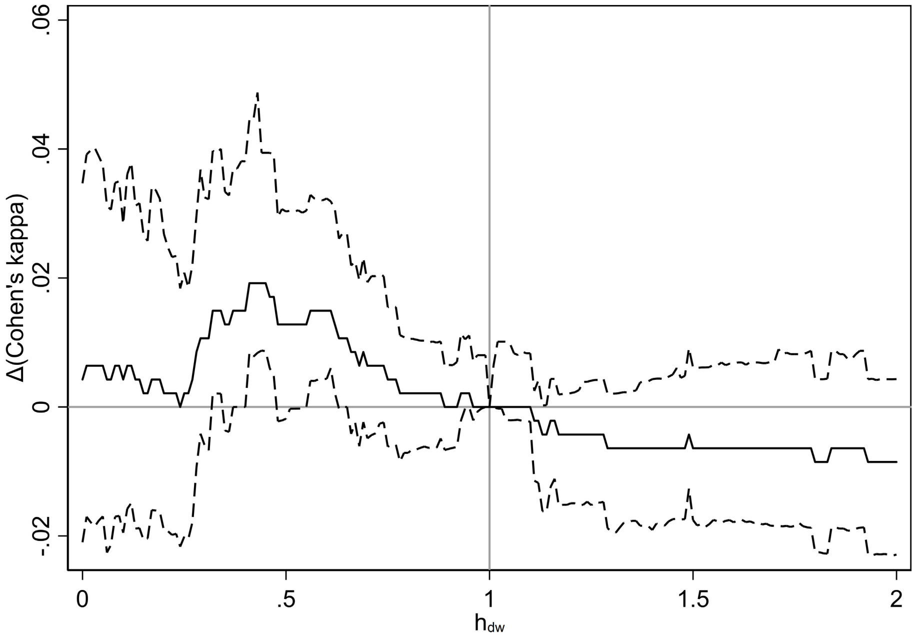

Controls matched to cases by age, gender and municipality of recidence.
Solid line is estimate, dashed lines are 95% confidence interval.

Adjusted analysis

Cohen's kappa as a function of h_{dw}

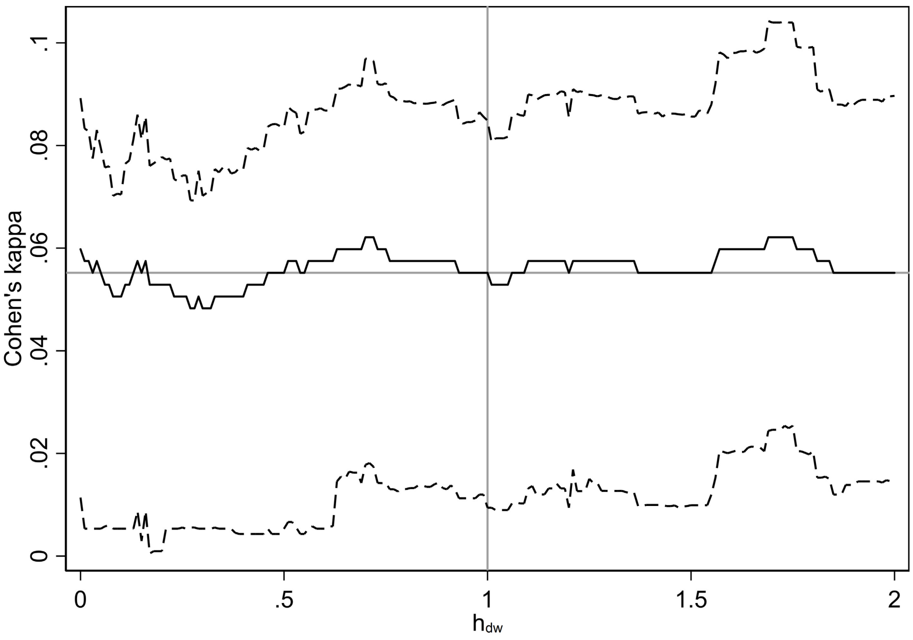

Controls matched to cases by age, gender and municipality of recidence. Analysis adjusted for age, gender, education and income.
Solid line is estimate, dashed lines are 95% confidence interval.

Δ(Cohen's kappa) as a function of h_{dw}

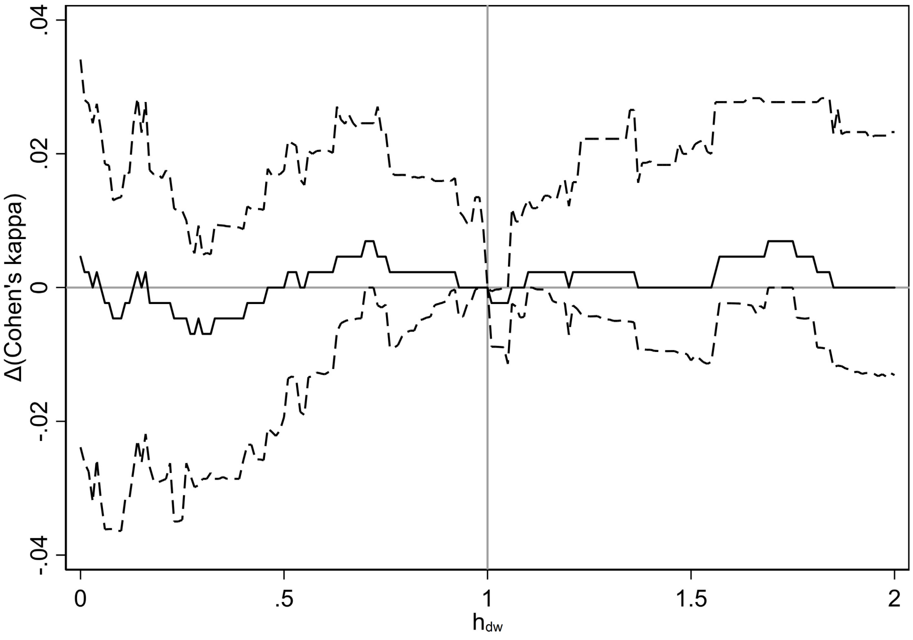

Controls matched to cases by age, gender and municipality of recidence. Analysis adjusted for age, gender, education and income.
Solid line is estimate, dashed lines are 95% confidence interval.

Secondary results for this analysis: Description of hyperparameter optimization

Cohen's kappa as a function of IDW power

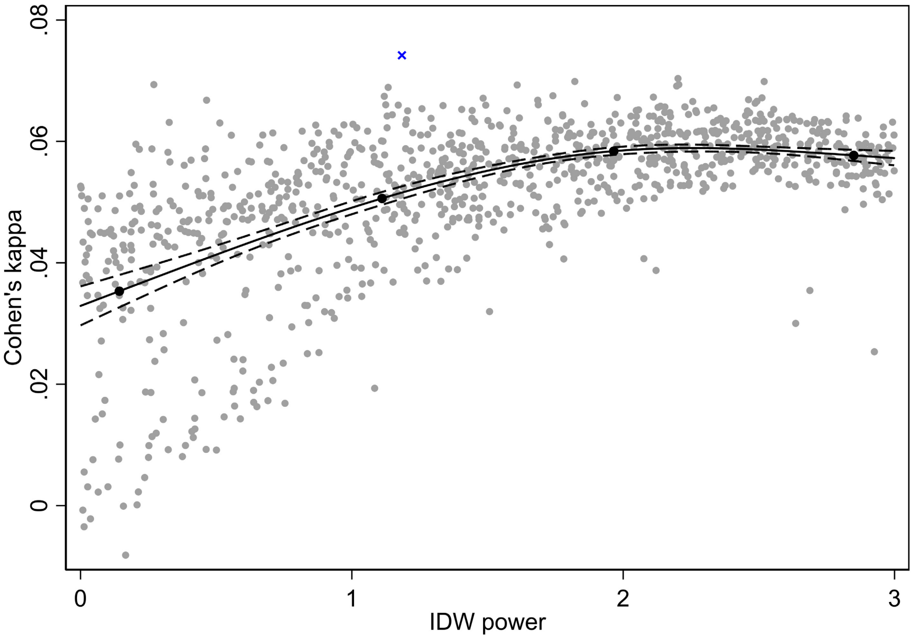

Each gray dot represents one crude analysis. The analyses used random time intervals and search radii. The blue × represents the analysis with the optimum combination of hyperparameters. The solid line represent the trend, modelled with restricted cubic splines with four knots (the location of the knots indicated by black dots). The dashed lines are the 95% confidence interval for the trend.

Cohen's kappa as a function of search radius

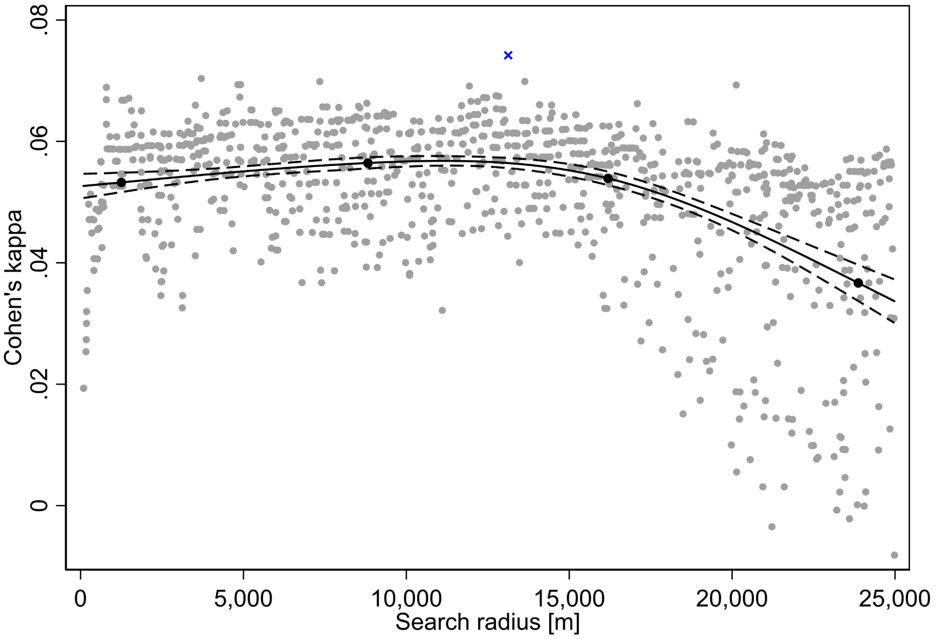

Each gray dot represents one crude analysis. The analyses used random time intervals and values of IDW power. The blue × represents the analysis with the optimum combination of hyperparameters. The solid line represent the trend, modelled with restricted cubic splines with four knots (the location of the knots indicated by black dots). The dashed lines are the 95% confidence interval for the trend.

Cohen's kappa as a function of time interval

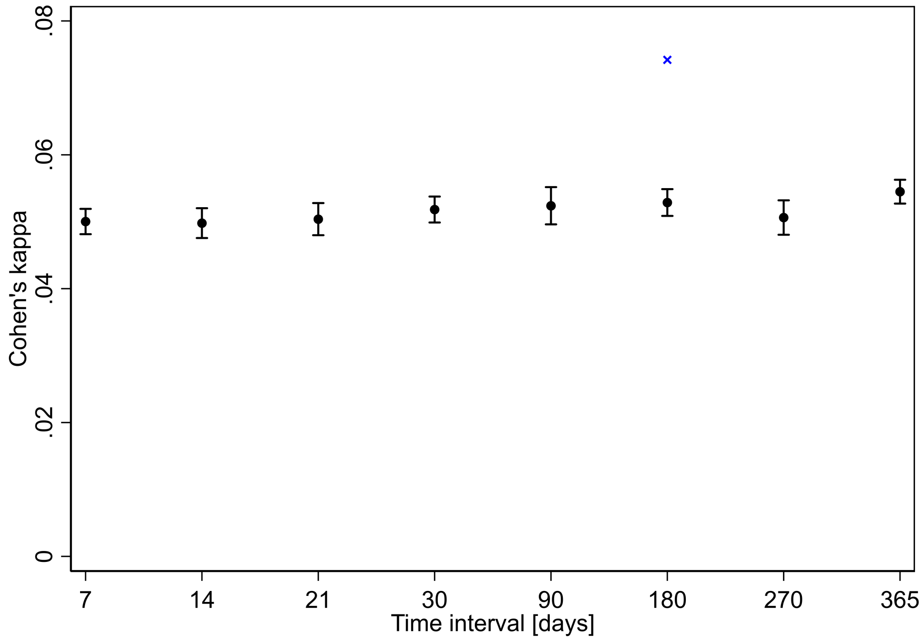

Each black dot represents the mean of the results for crude analyses with the indicated time interval. The analyses used random values of IDW power and search radius. The bars indicate the 95% confidence interval. The blue × represents the analysis with the optimum combination of hyperparameters.

Cohen's kappa as a function of search radius, IDW power and time interval

Time interval: 7 days

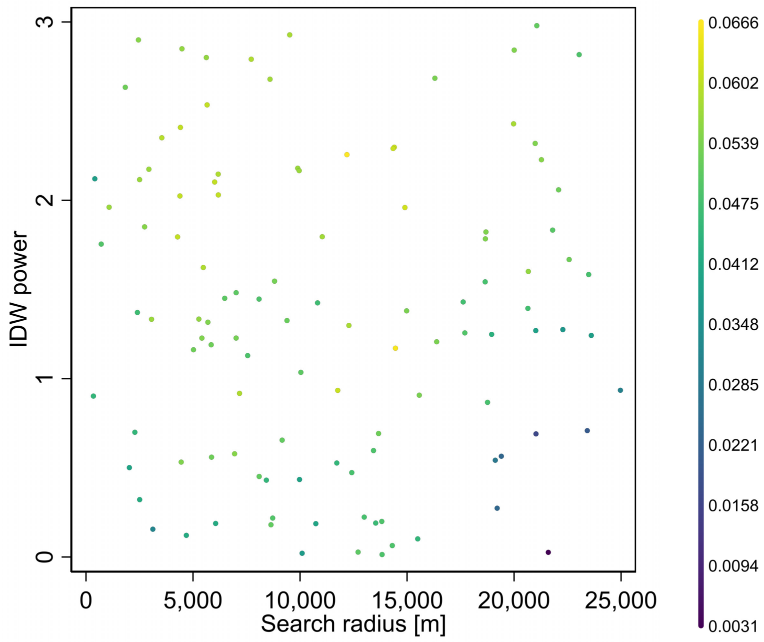

Each dot represents one crude analysis with the specified hyperparameters. Dots are color-coded according to the value of Cohen's kappa.

Time interval: 14 days

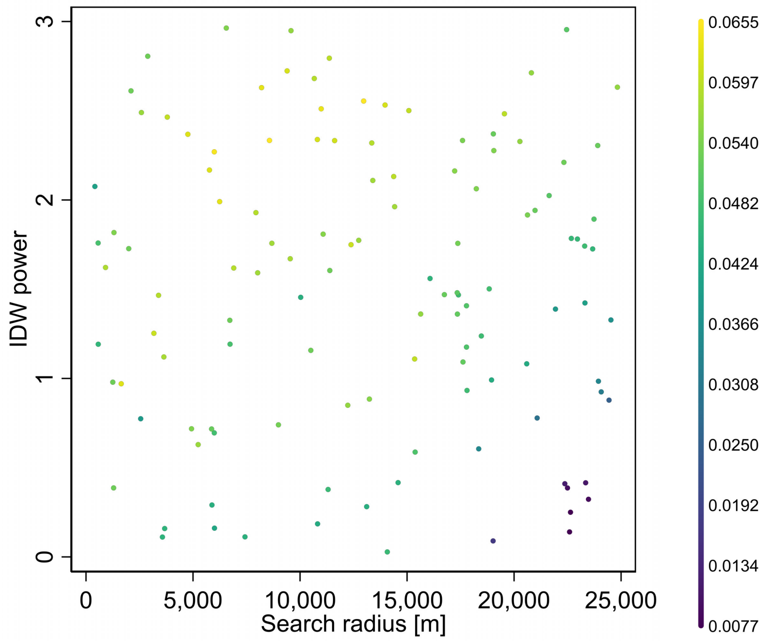

Each dot represents one crude analysis with the specified hyperparameters. Dots are color-coded according to the value of Cohen's kappa.

Time interval: 21 days

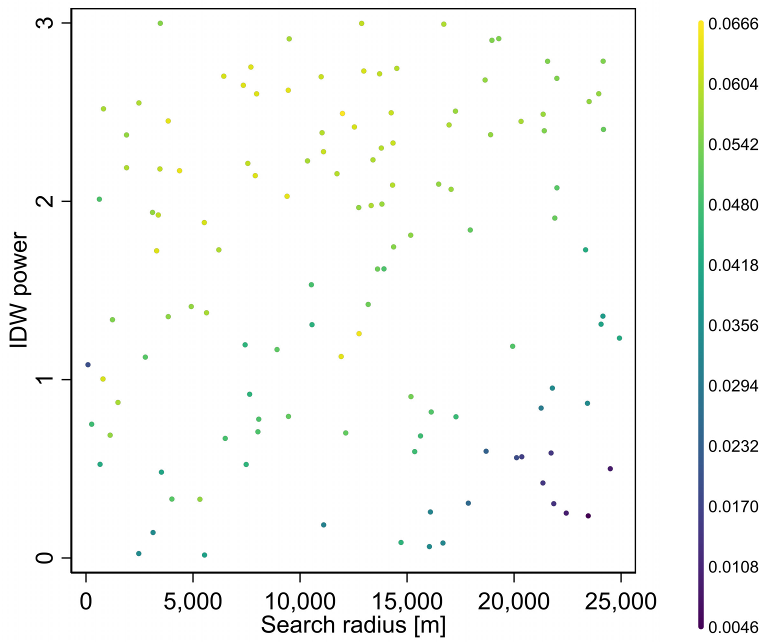

Each dot represents one crude analysis with the specified hyperparameters. Dots are color-coded according to the value of Cohen's kappa.

Time interval: 30 days

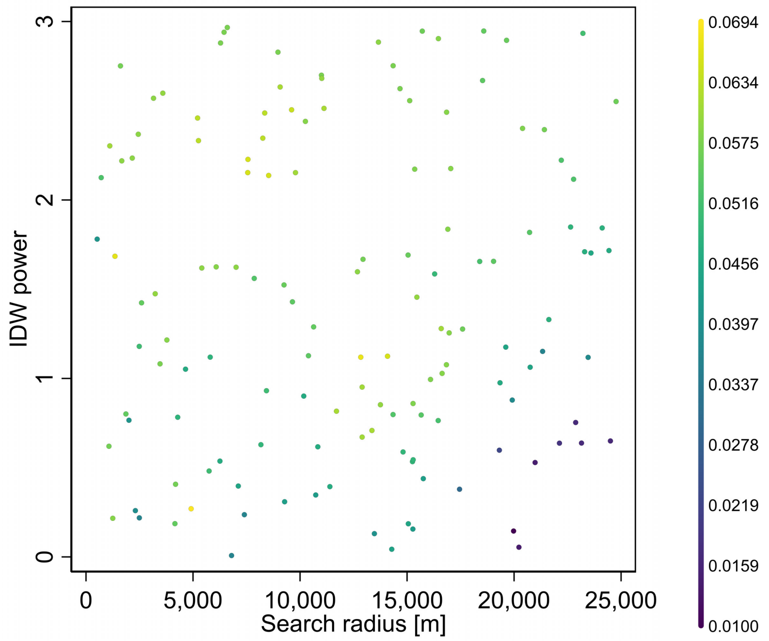

Each dot represents one crude analysis with the specified hyperparameters. Dots are color-coded according to the value of Cohen's kappa.

Time interval: 90 days

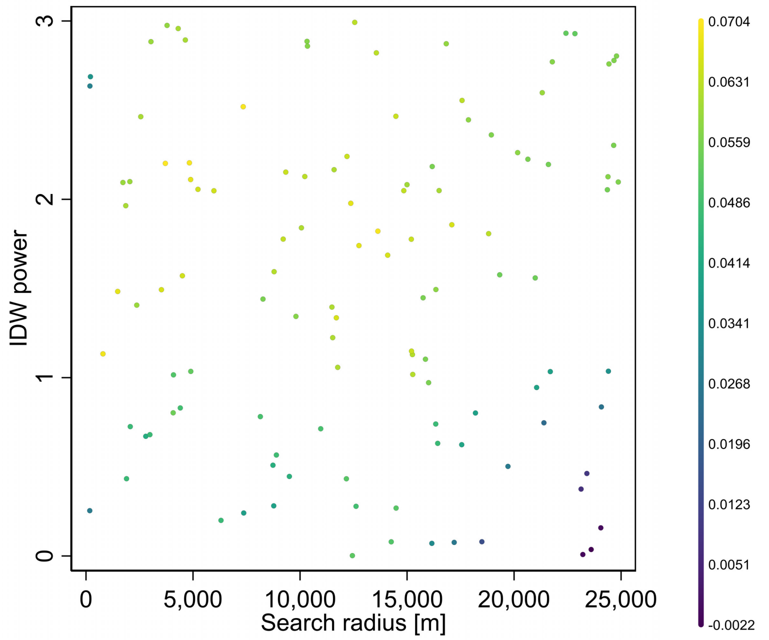

Each dot represents one crude analysis with the specified hyperparameters. Dots are color-coded according to the value of Cohen's kappa.

Time interval: 180 days

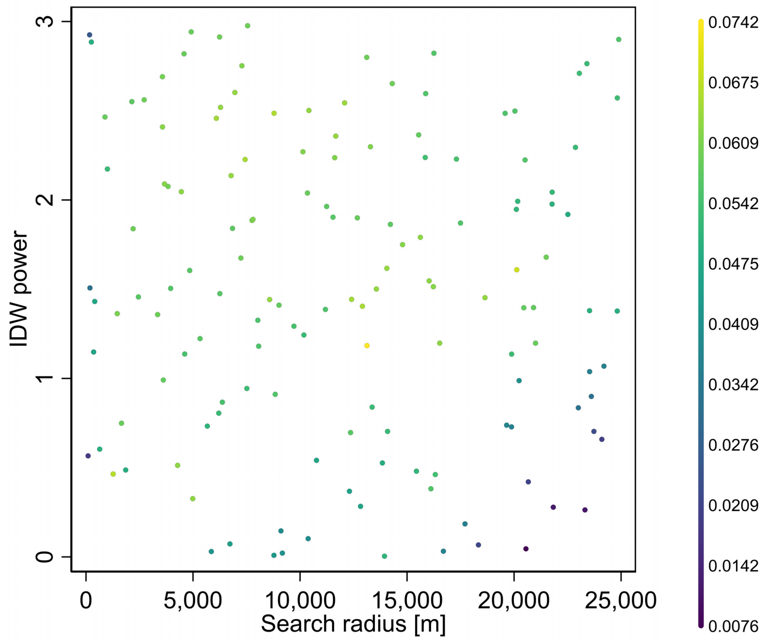

Each dot represents one crude analysis with the specified hyperparameters. Dots are color-coded according to the value of Cohen's kappa.

Time interval: 270 days

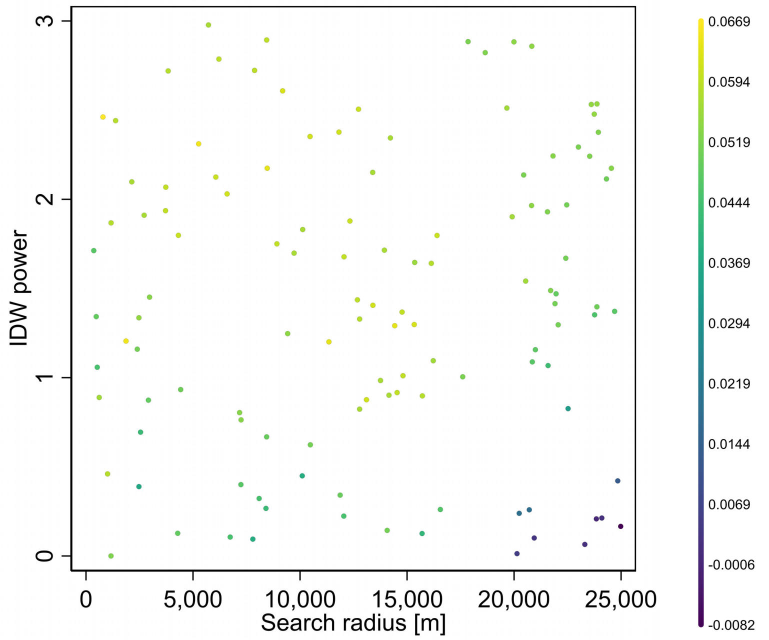

Each dot represents one crude analysis with the specified hyperparameters. Dots are color-coded according to the value of Cohen's kappa.

Time interval: 365 days

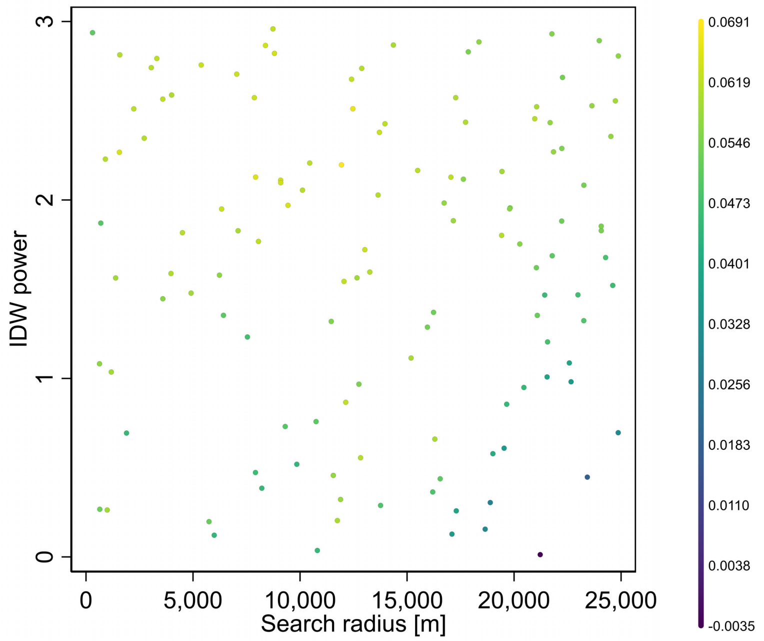

Each dot represents one crude analysis with the specified hyperparameters. Dots are color-coded according to the value of Cohen's kappa.

Time interval: Any

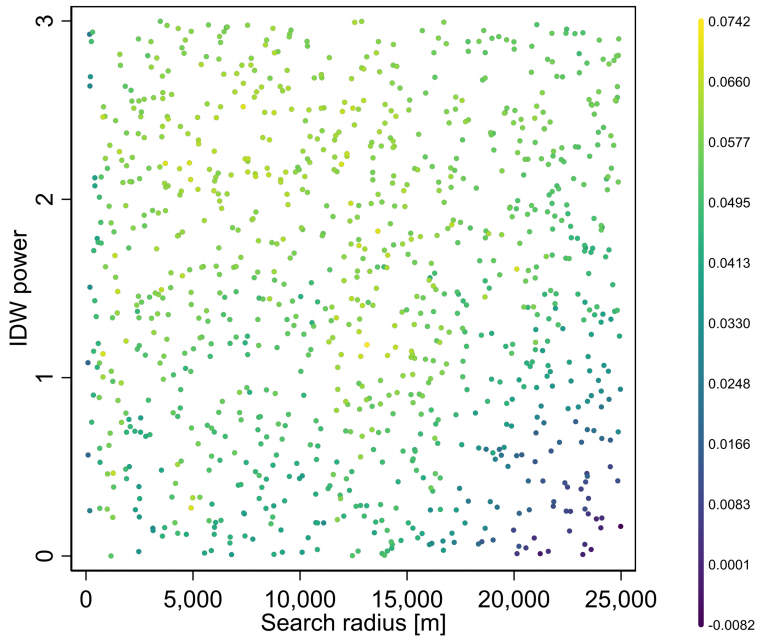

Each dot represents one crude analysis with the specified hyperparameters. Dots are color-coded according to the value of Cohen's kappa.

Analysis number 2

Primary results from this analysis

Crude analysis

Cohen's kappa as a function of h_{dw}

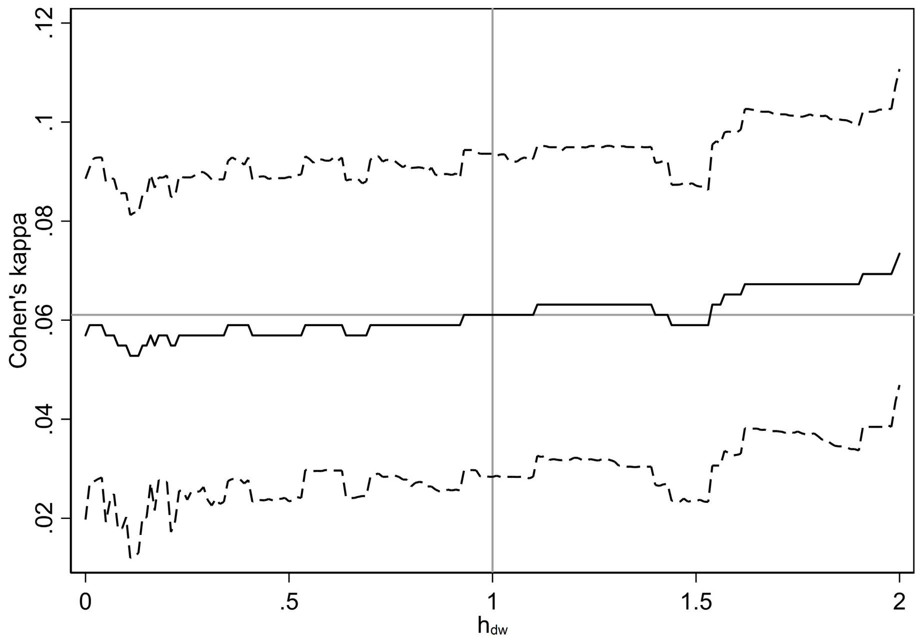

Controls matched to cases by age, gender and municipality of recidence.
Solid line is estimate, dashed lines are 95% confidence interval.

Δ(Cohen's kappa) as a function of h_{dw}

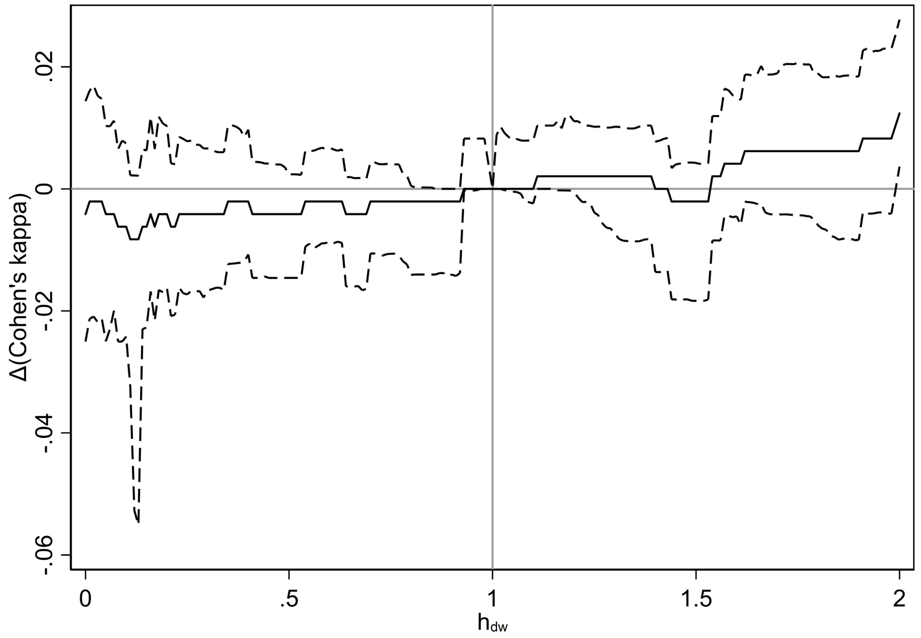

Controls matched to cases by age, gender and municipality of recidence.
Solid line is estimate, dashed lines are 95% confidence interval.

Adjusted analysis

Cohen's kappa as a function of h_{dw}

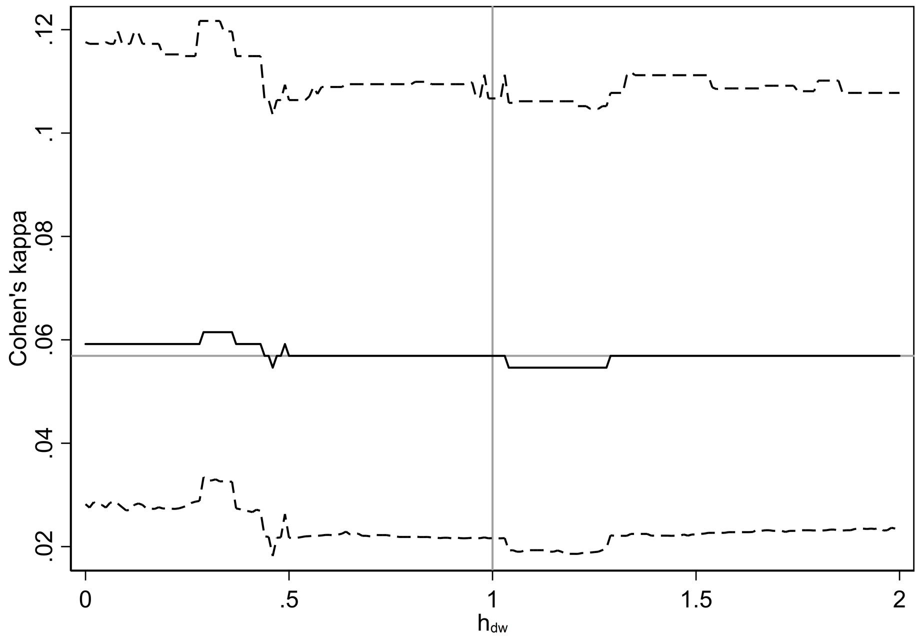

Controls matched to cases by age, gender and municipality of recidence. Analysis adjusted for age, gender, education and income.
Solid line is estimate, dashed lines are 95% confidence interval.

Δ(Cohen's kappa) as a function of h_{dw}

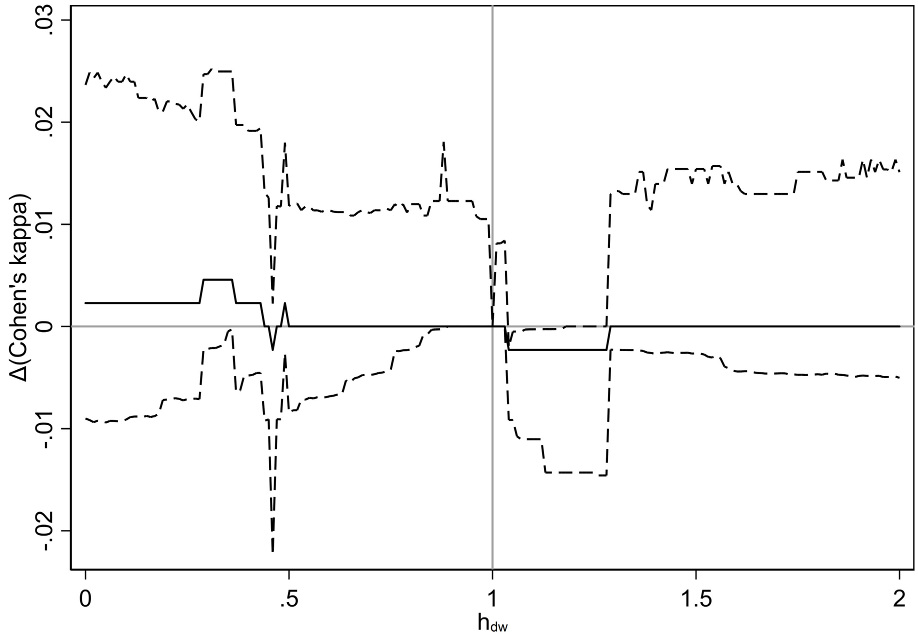

Controls matched to cases by age, gender and municipality of recidence. Analysis adjusted for age, gender, education and income.
Solid line is estimate, dashed lines are 95% confidence interval.

Secondary results for this analysis: Description of hyperparameter optimization

Cohen's kappa as a function of IDW power

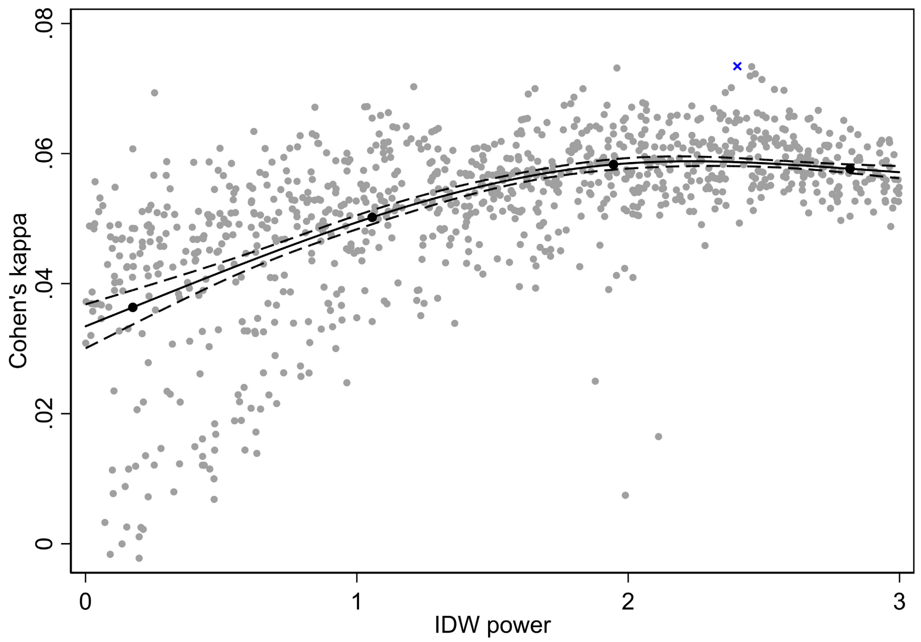

Each gray dot represents one crude analysis. The analyses used random time intervals and search radii. The blue × represents the analysis with the optimum combination of hyperparameters. The solid line represent the trend, modelled with restricted cubic splines with four knots (the location of the knots indicated by black dots). The dashed lines are the 95% confidence interval for the trend.

Cohen's kappa as a function of search radius

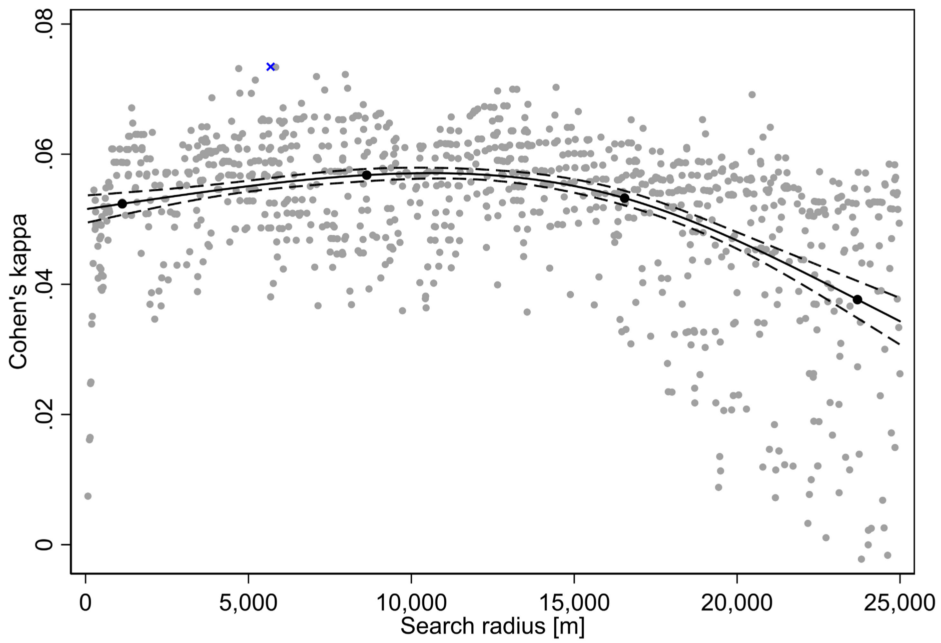

Each gray dot represents one crude analysis. The analyses used random time intervals and values of IDW power. The blue × represents the analysis with the optimum combination of hyperparameters. The solid line represent the trend, modelled with restricted cubic splines with four knots (the location of the knots indicated by black dots). The dashed lines are the 95% confidence interval for the trend.

Cohen's kappa as a function of time interval

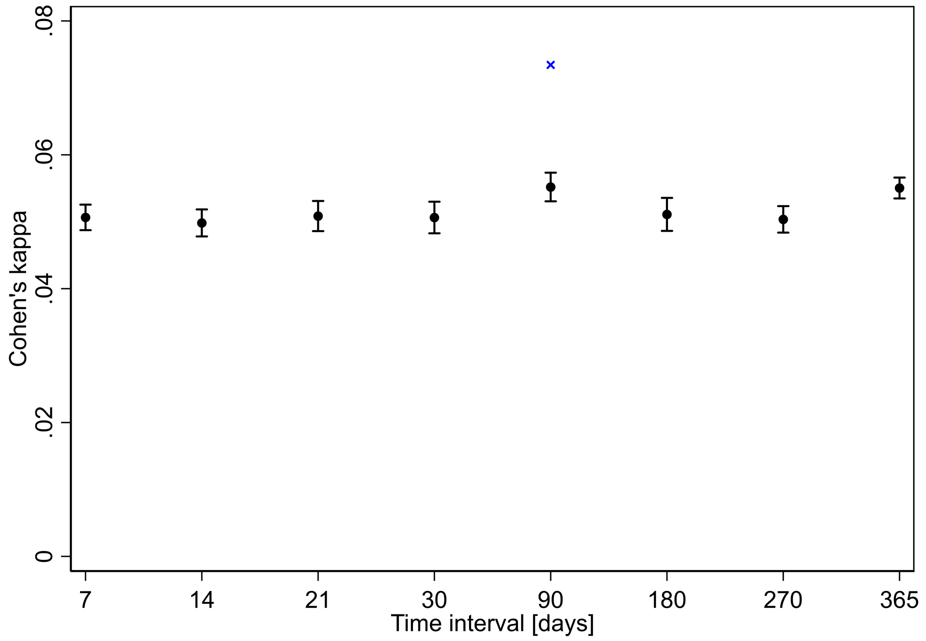

Each black dot represents the mean of the results for crude analyses with the indicated time interval. The analyses used random values of IDW power and search radius. The bars indicate the 95% confidence interval. The blue × represents the analysis with the optimum combination of hyperparameters.

Cohen's kappa as a function of search radius, IDW power and time interval

Time interval: 7 days

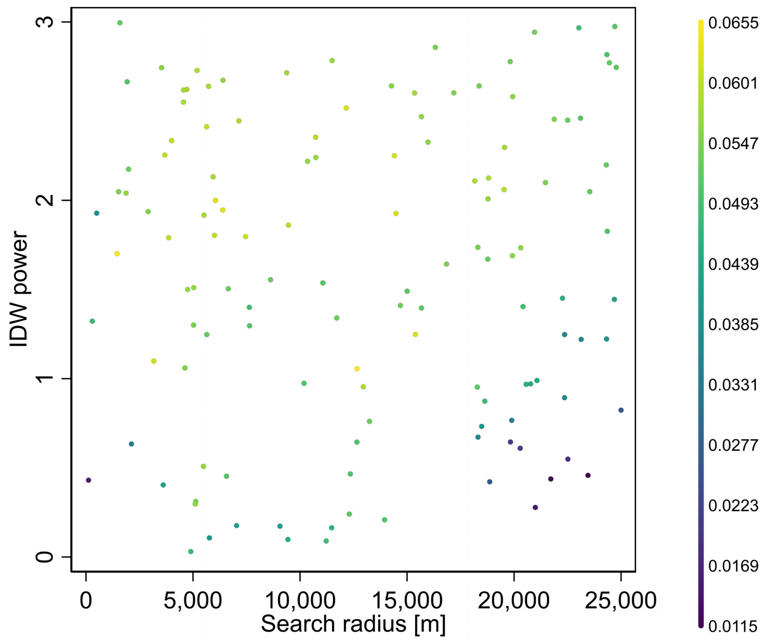

Each dot represents one crude analysis with the specified hyperparameters. Dots are color-coded according to the value of Cohen's kappa.

Time interval: 14 days

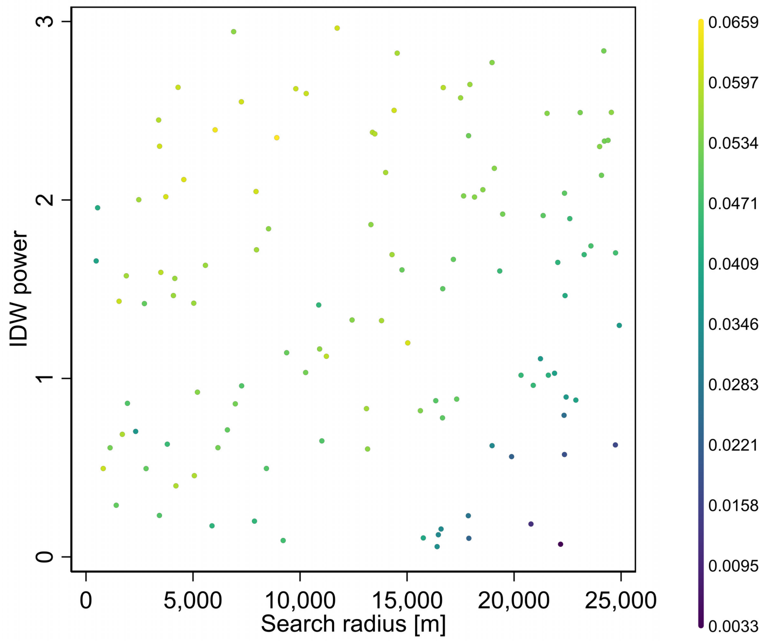

Each dot represents one crude analysis with the specified hyperparameters. Dots are color-coded according to the value of Cohen's kappa.

Time interval: 21 days

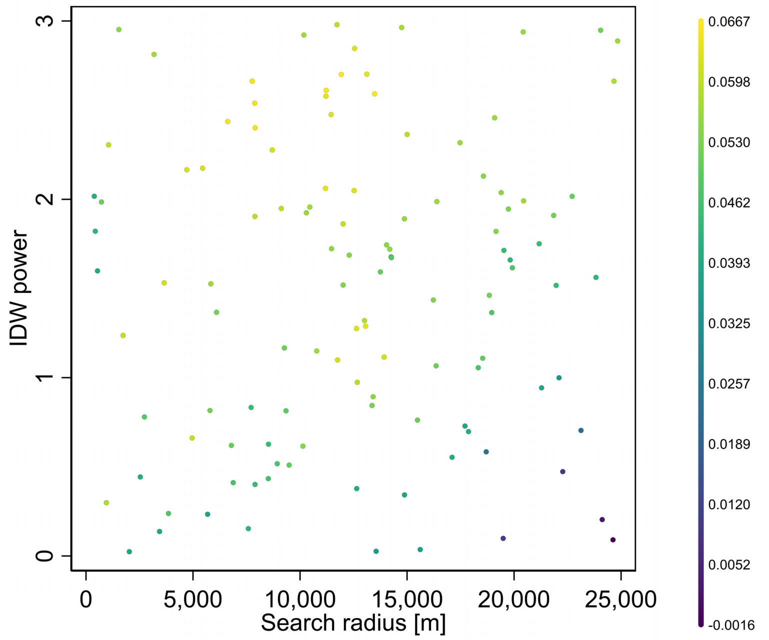

Each dot represents one crude analysis with the specified hyperparameters. Dots are color-coded according to the value of Cohen's kappa.

Time interval: 30 days

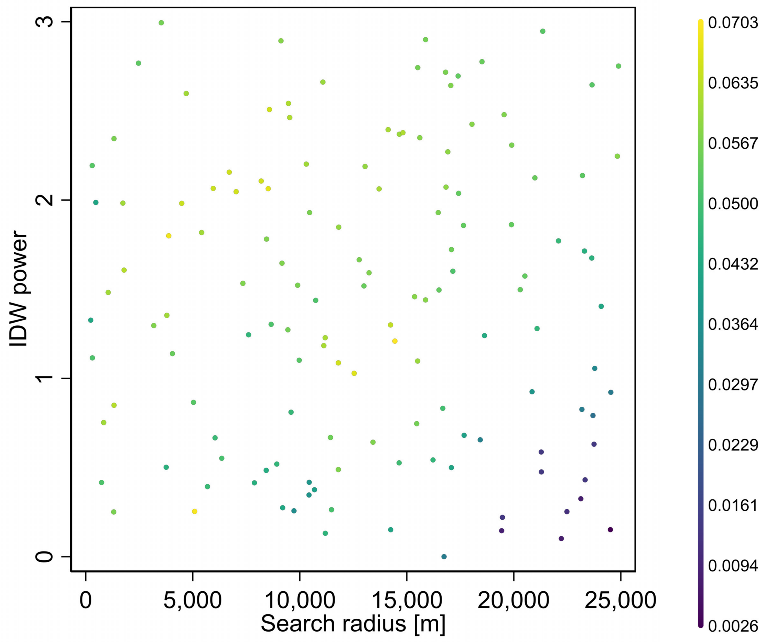

Each dot represents one crude analysis with the specified hyperparameters. Dots are color-coded according to the value of Cohen's kappa.

Time interval: 90 days

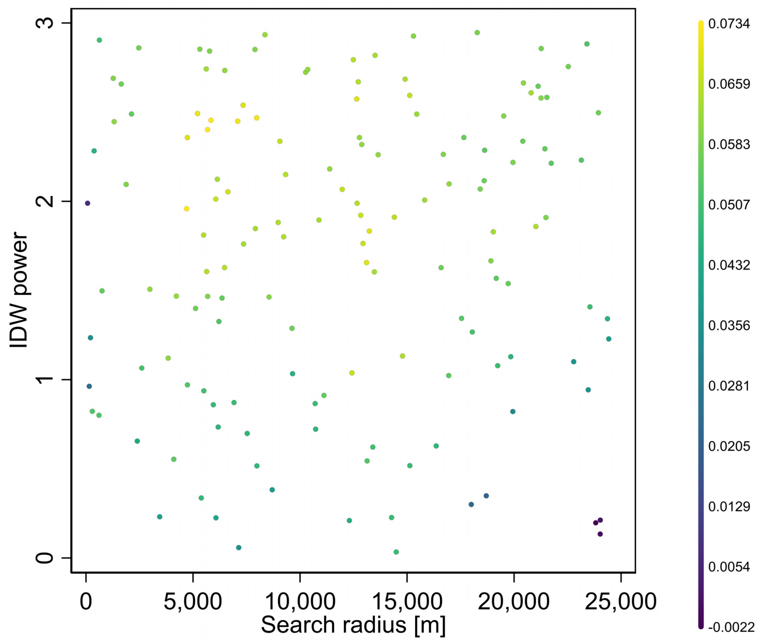

Each dot represents one crude analysis with the specified hyperparameters. Dots are color-coded according to the value of Cohen's kappa.

Time interval: 180 days

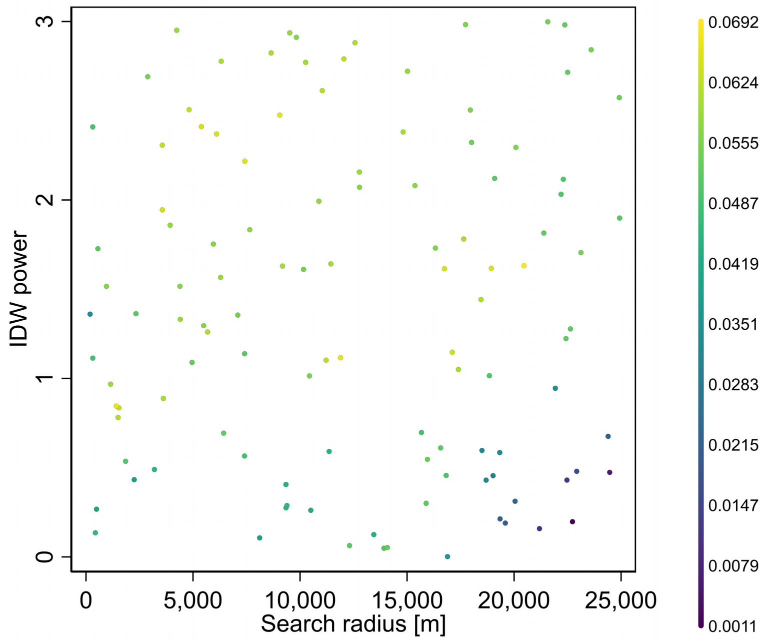

Each dot represents one crude analysis with the specified hyperparameters. Dots are color-coded according to the value of Cohen's kappa.

Time interval: 270 days

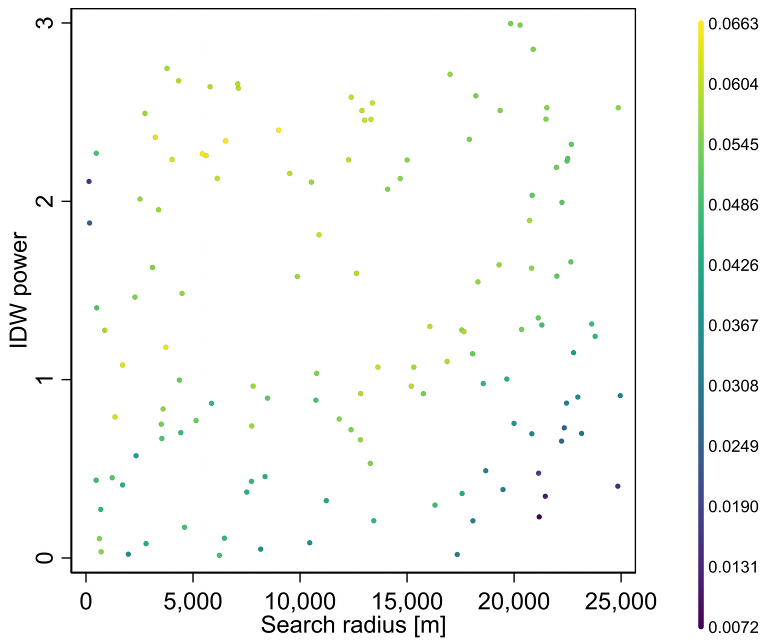

Each dot represents one crude analysis with the specified hyperparameters. Dots are color-coded according to the value of Cohen's kappa.

Time interval: 365 days

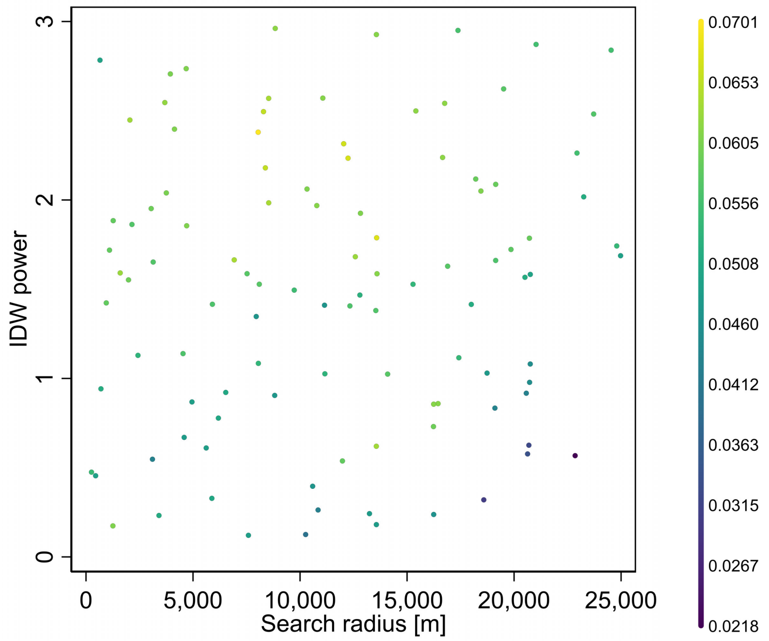

Each dot represents one crude analysis with the specified hyperparameters. Dots are color-coded according to the value of Cohen's kappa.

Time interval: Any

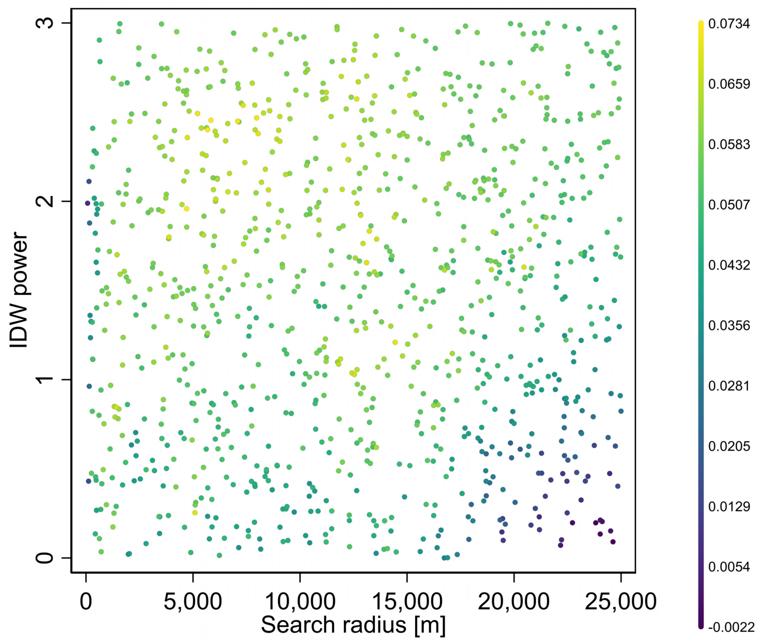

Each dot represents one crude analysis with the specified hyperparameters. Dots are color-coded according to the value of Cohen's kappa.

Analysis number 3

Primary results from this analysis

Crude analysis

Cohen's kappa as a function of h_{dw}

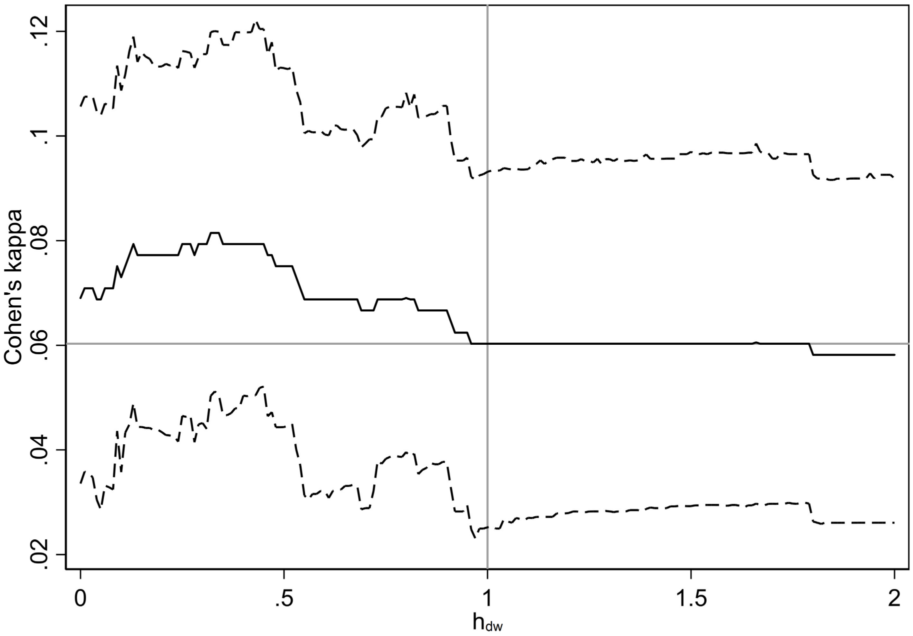

Controls matched to cases by age, gender and municipality of recidence.
Solid line is estimate, dashed lines are 95% confidence interval.

Δ(Cohen's kappa) as a function of h_{dw}

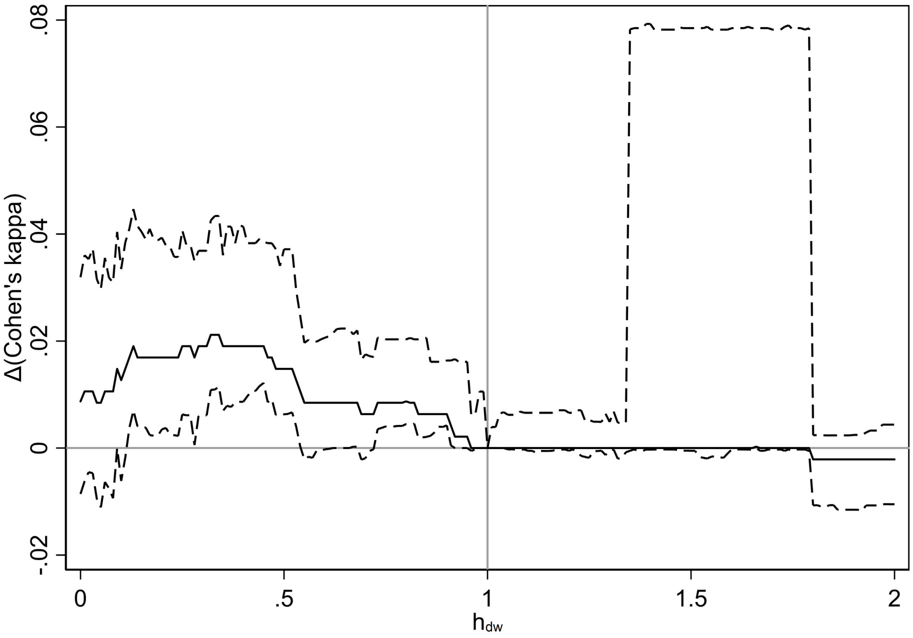

Controls matched to cases by age, gender and municipality of recidence.
Solid line is estimate, dashed lines are 95% confidence interval.

Adjusted analysis

Cohen's kappa as a function of h_{dw}

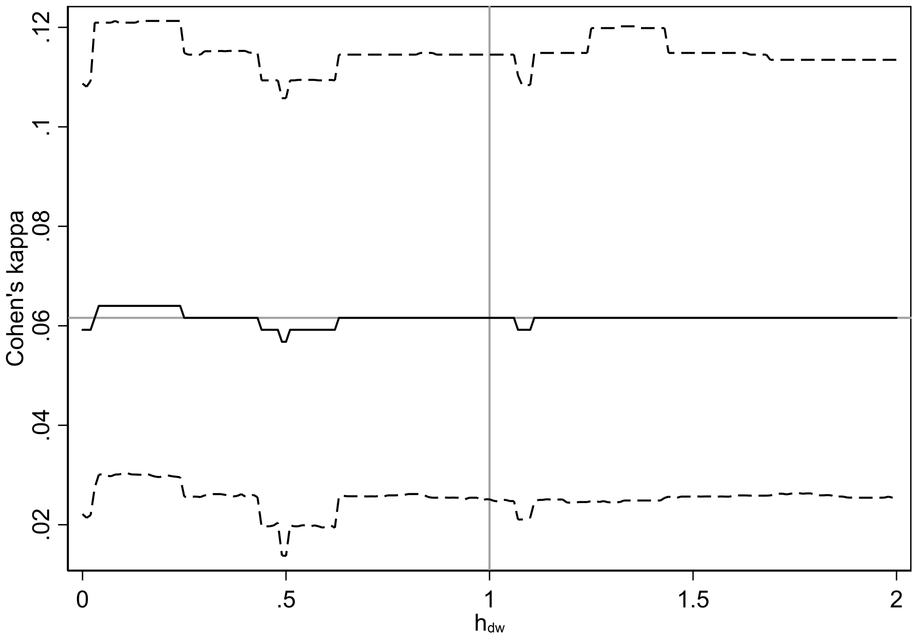

Controls matched to cases by age, gender and municipality of recidence. Analysis adjusted for age, gender, education and income.
Solid line is estimate, dashed lines are 95% confidence interval.

Δ(Cohen's kappa) as a function of h_{dw}

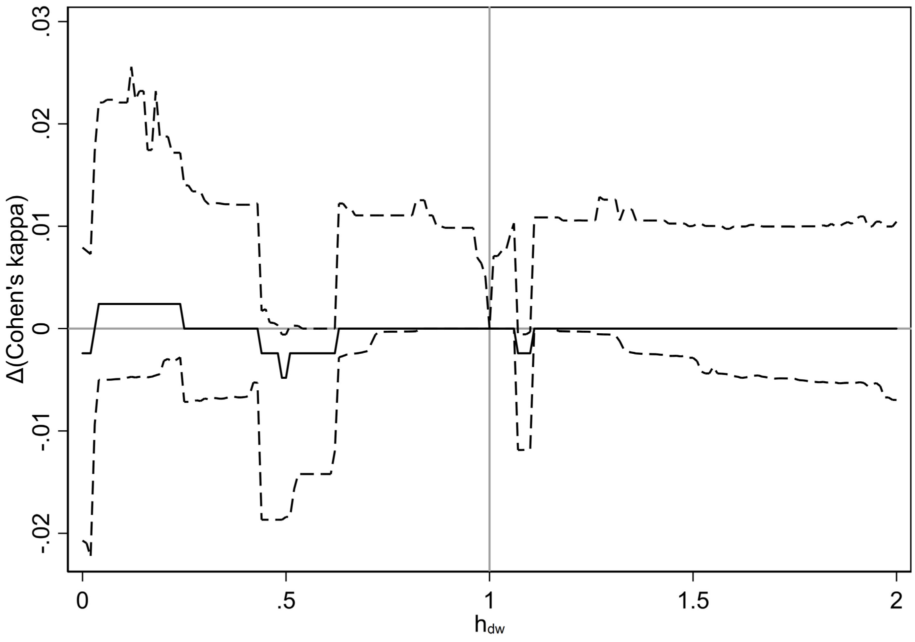

Controls matched to cases by age, gender and municipality of recidence. Analysis adjusted for age, gender, education and income.
Solid line is estimate, dashed lines are 95% confidence interval.

Secondary results for this analysis: Description of hyperparameter optimization

Cohen's kappa as a function of IDW power

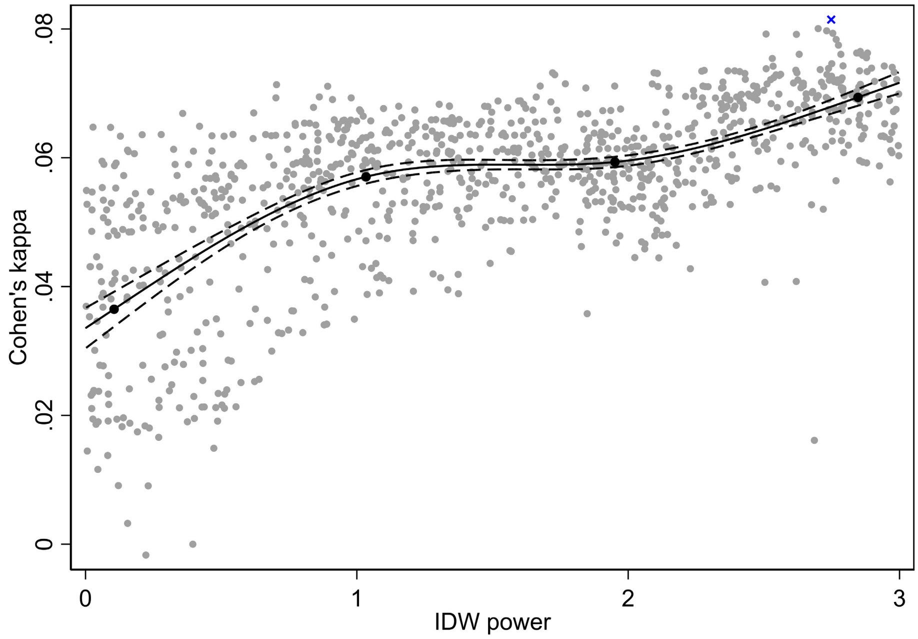

Each gray dot represents one crude analysis. The analyses used random time intervals and search radii. The blue × represents the analysis with the optimum combination of hyperparameters. The solid line represent the trend, modelled with restricted cubic splines with four knots (the location of the knots indicated by black dots). The dashed lines are the 95% confidence interval for the trend.

Cohen's kappa as a function of search radius

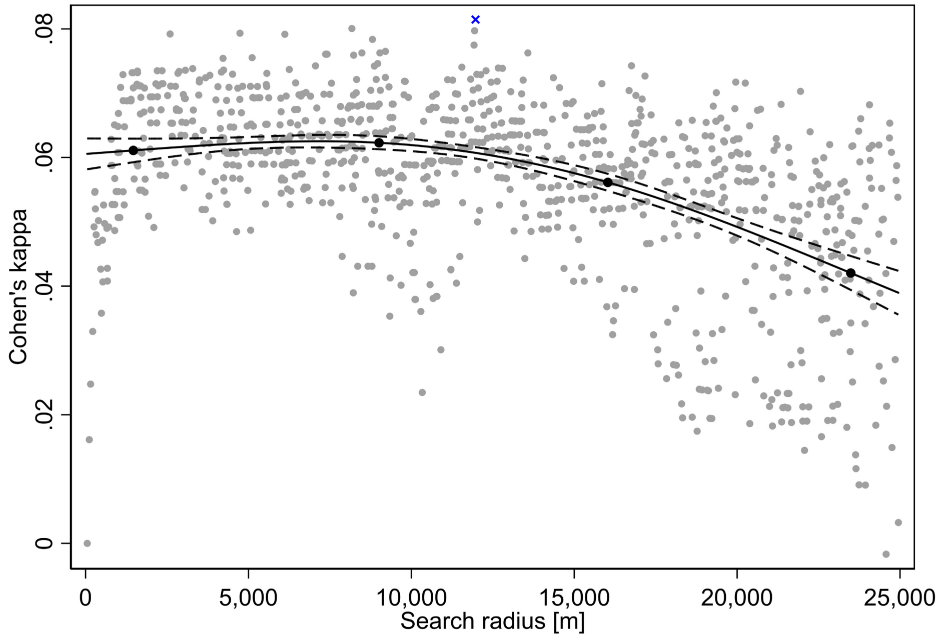

Each gray dot represents one crude analysis. The analyses used random time intervals and values of IDW power. The blue × represents the analysis with the optimum combination of hyperparameters. The solid line represent the trend, modelled with restricted cubic splines with four knots (the location of the knots indicated by black dots). The dashed lines are the 95% confidence interval for the trend.

Cohen's kappa as a function of time interval

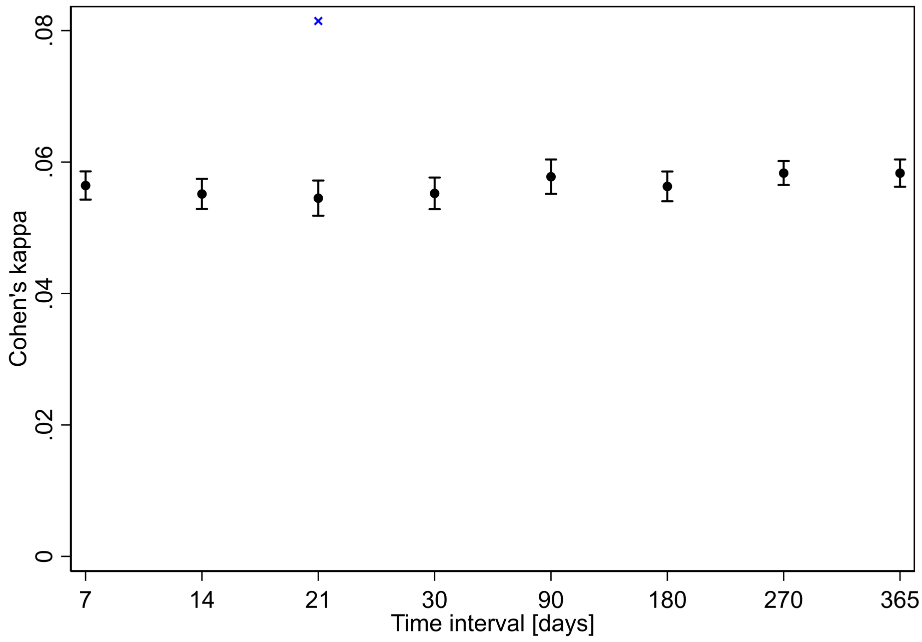

Each black dot represents the mean of the results for crude analyses with the indicated time interval. The analyses used random values of IDW power and search radius. The bars indicate the 95% confidence interval. The blue × represents the analysis with the optimum combination of hyperparameters.

Cohen's kappa as a function of search radius, IDW power and time interval

Time interval: 7 days

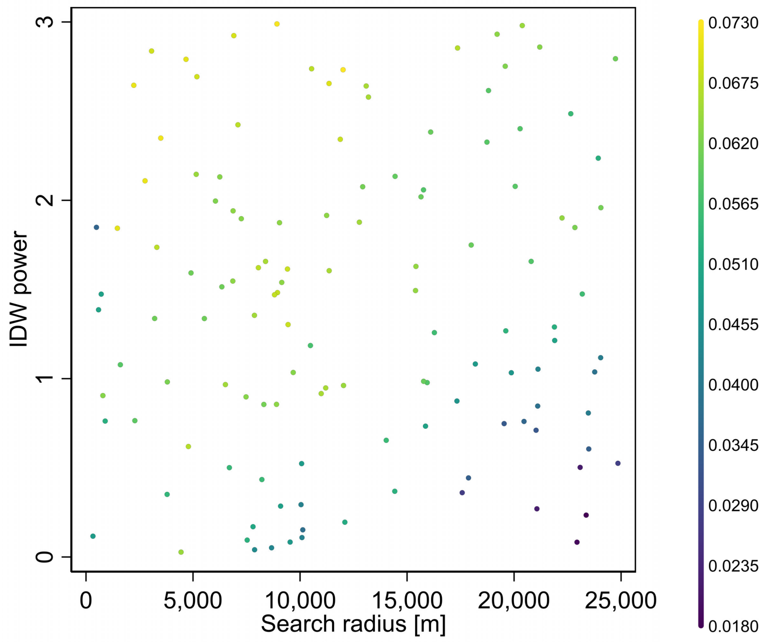

Each dot represents one crude analysis with the specified hyperparameters. Dots are color-coded according to the value of Cohen's kappa.

Time interval: 14 days

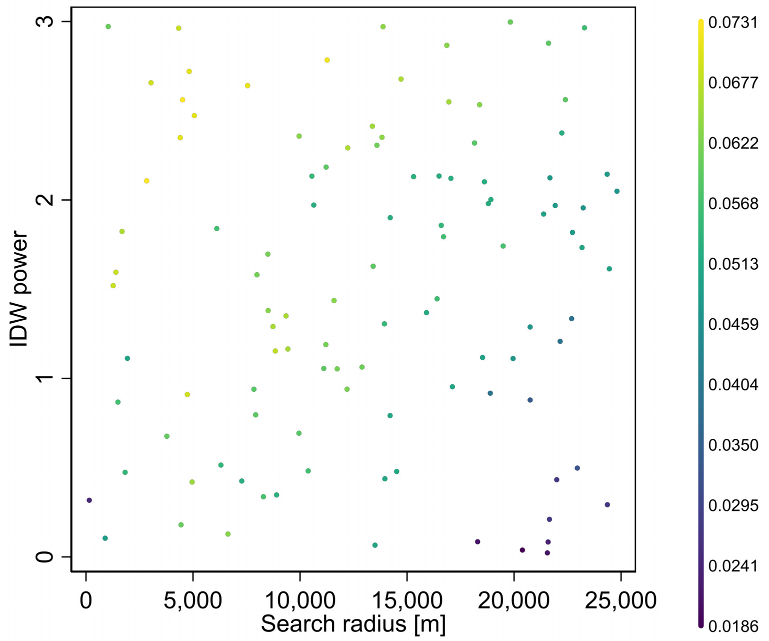

Each dot represents one crude analysis with the specified hyperparameters. Dots are color-coded according to the value of Cohen's kappa.

Time interval: 21 days

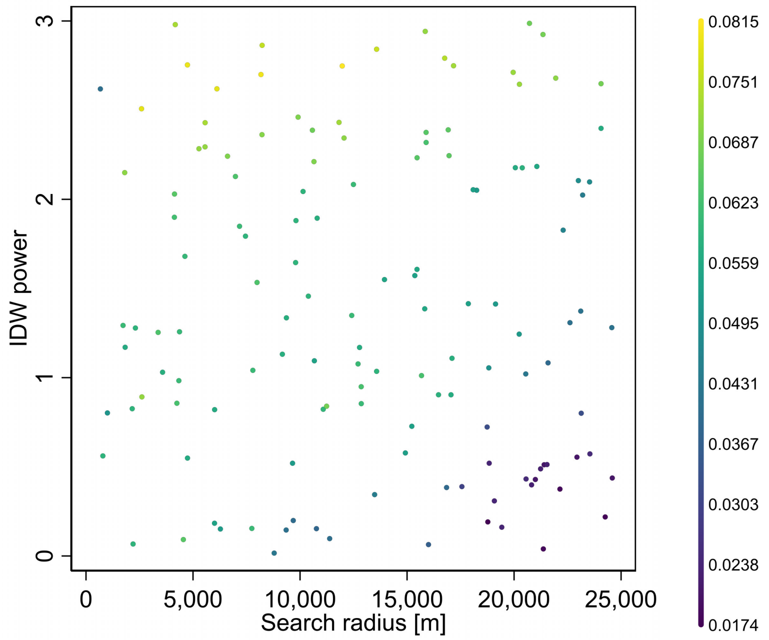

Each dot represents one crude analysis with the specified hyperparameters. Dots are color-coded according to the value of Cohen's kappa.

Time interval: 30 days

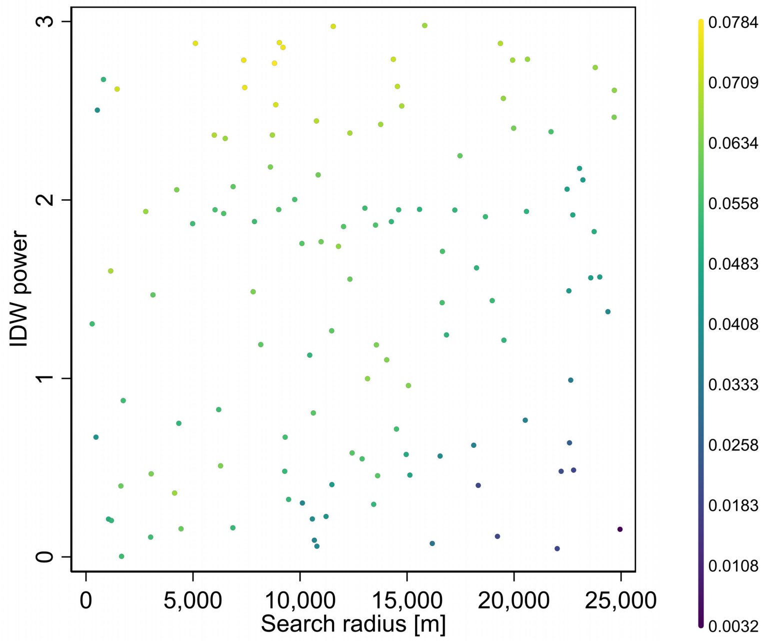

Each dot represents one crude analysis with the specified hyperparameters. Dots are color-coded according to the value of Cohen's kappa.

Time interval: 90 days

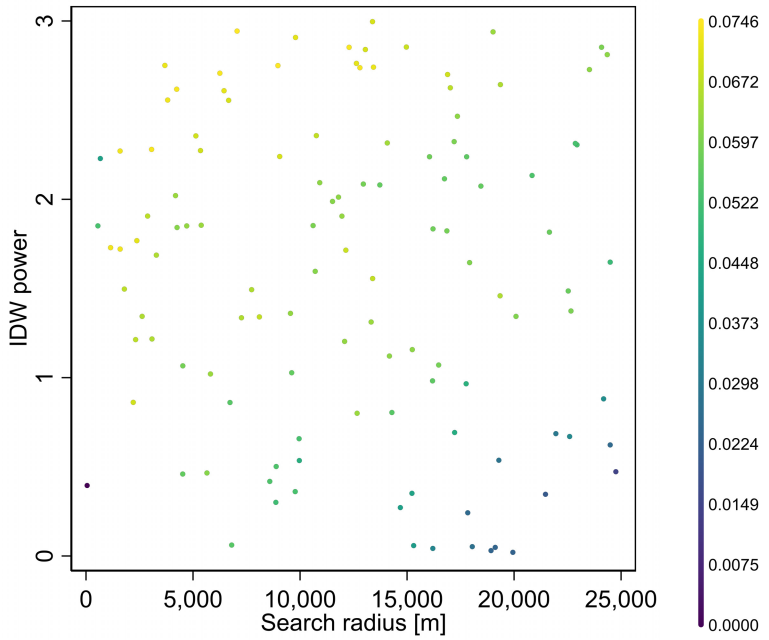

Each dot represents one crude analysis with the specified hyperparameters. Dots are color-coded according to the value of Cohen's kappa.

Time interval: 180 days

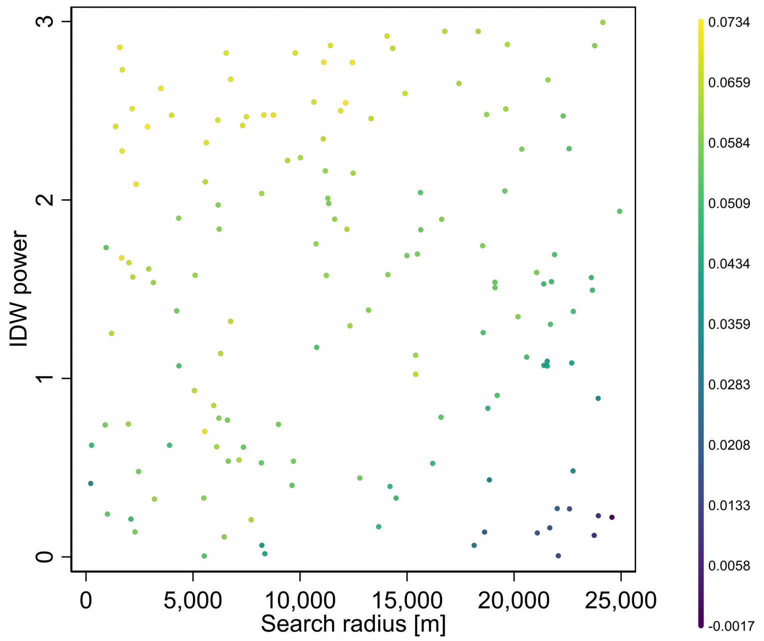

Each dot represents one crude analysis with the specified hyperparameters. Dots are color-coded according to the value of Cohen's kappa.

Time interval: 270 days

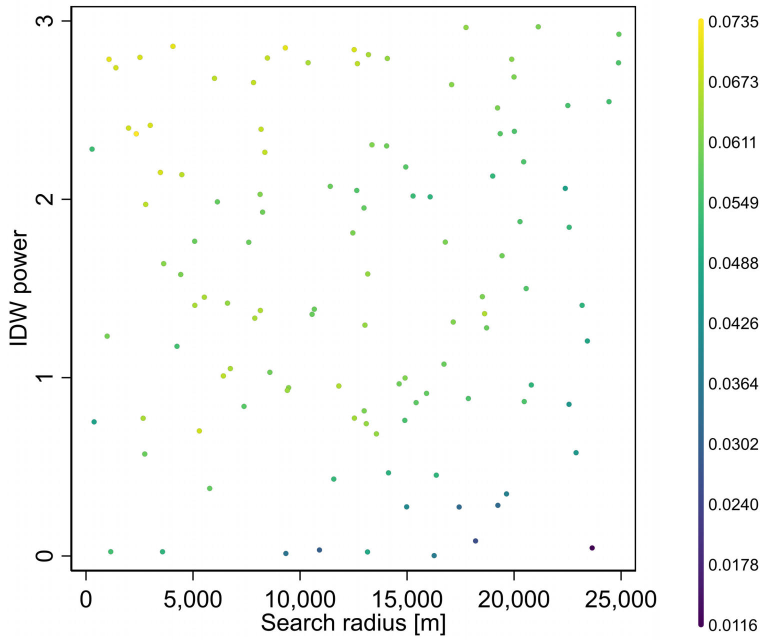

Each dot represents one crude analysis with the specified hyperparameters. Dots are color-coded according to the value of Cohen's kappa.

Time interval: 365 days

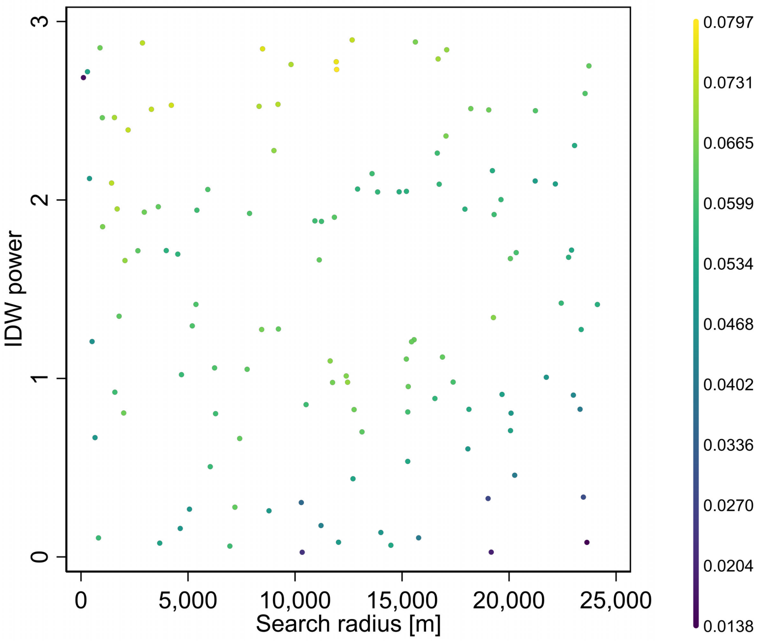

Each dot represents one crude analysis with the specified hyperparameters. Dots are color-coded according to the value of Cohen's kappa.

Time interval: Any

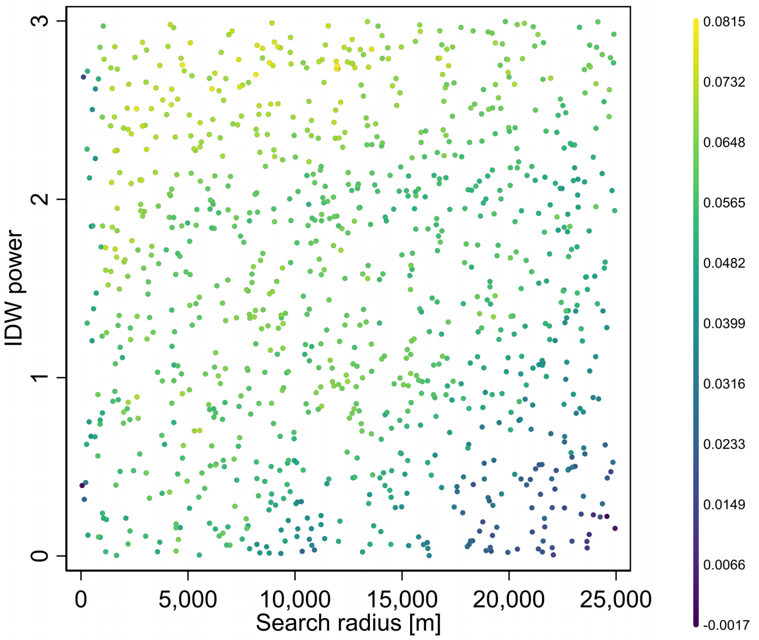

Each dot represents one crude analysis with the specified hyperparameters. Dots are color-coded according to the value of Cohen's kappa.

Analysis number 4

Primary results from this analysis

Crude analysis

Cohen's kappa as a function of h_{dw}

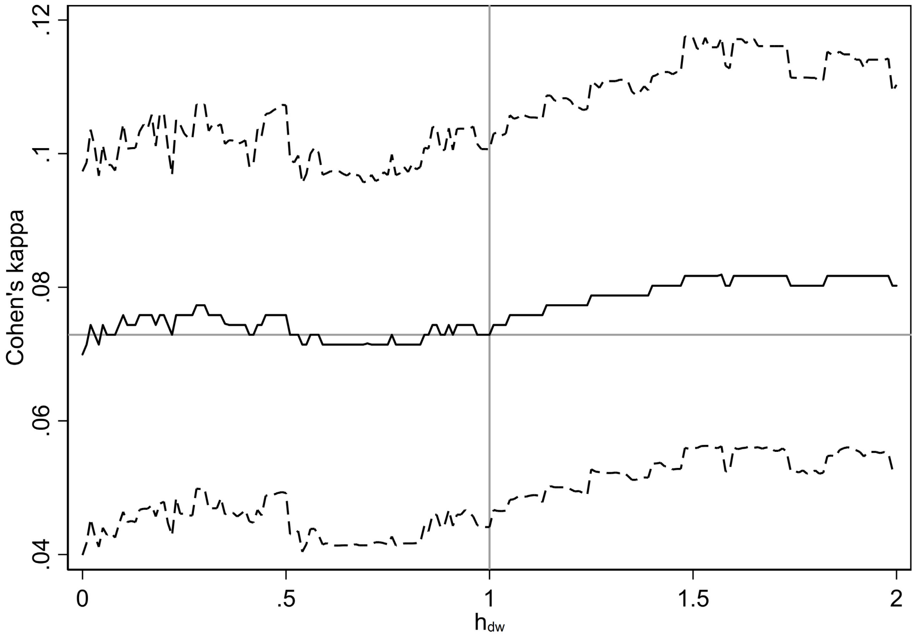

Controls matched to cases by age, gender and municipality of recidence.
Solid line is estimate, dashed lines are 95% confidence interval.

Δ(Cohen's kappa) as a function of h_{dw}

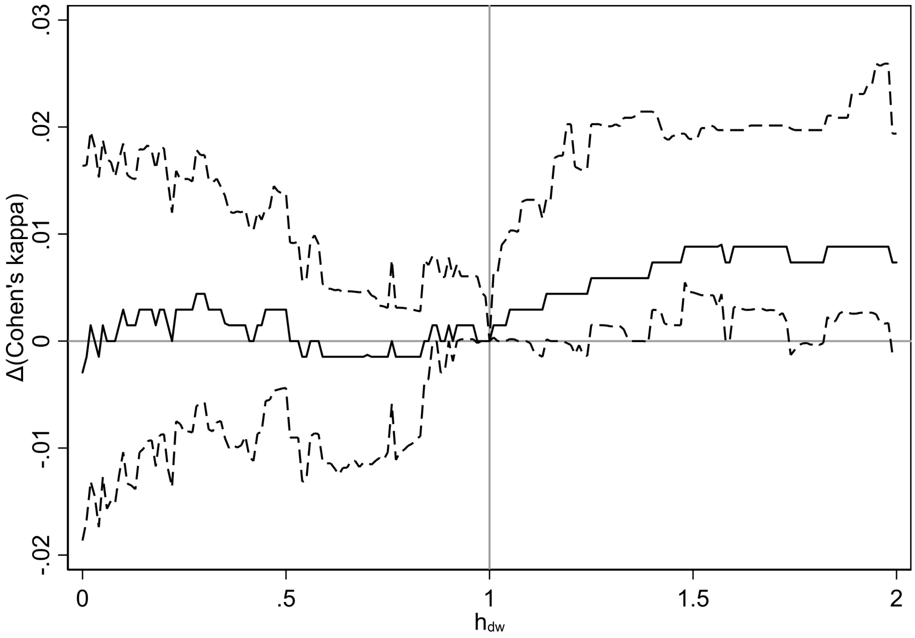

Controls matched to cases by age, gender and municipality of recidence.
Solid line is estimate, dashed lines are 95% confidence interval.

Adjusted analysis

Cohen's kappa as a function of h_{dw}

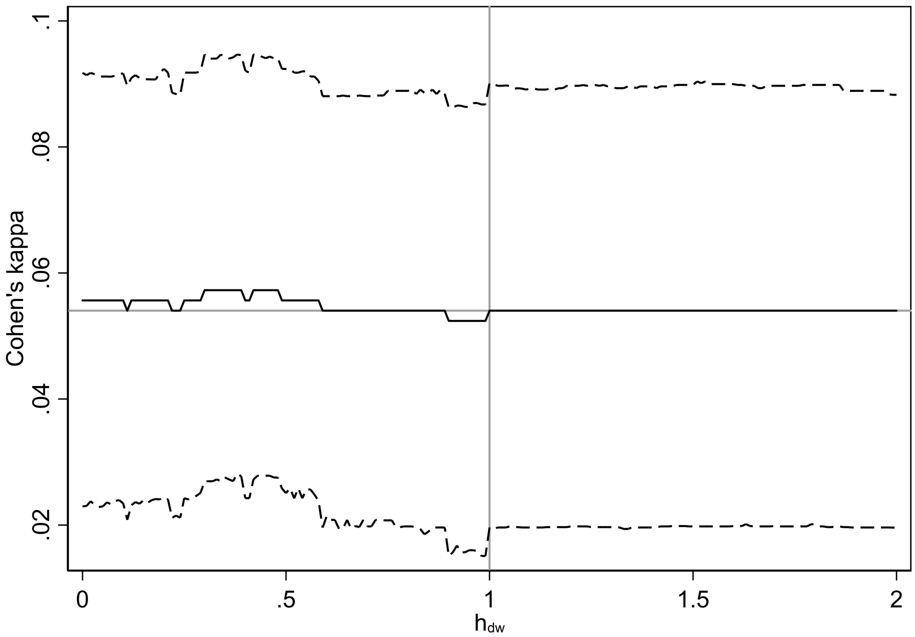

Controls matched to cases by age, gender and municipality of recidence. Analysis adjusted for age, gender, education and income.
Solid line is estimate, dashed lines are 95% confidence interval.

Δ(Cohen's kappa) as a function of h_{dw}

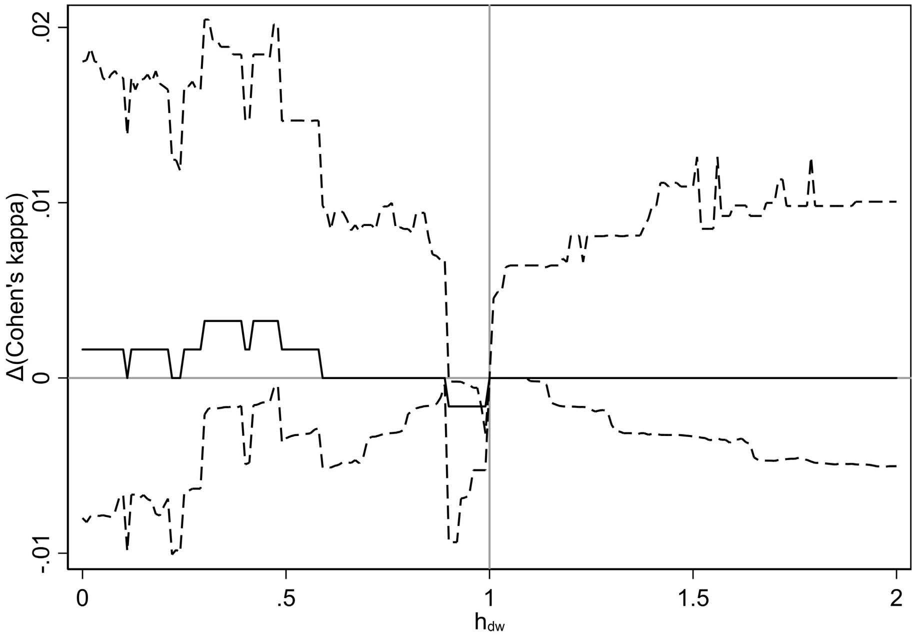

Controls matched to cases by age, gender and municipality of recidence. Analysis adjusted for age, gender, education and income.
Solid line is estimate, dashed lines are 95% confidence interval.

Secondary results for this analysis: Description of hyperparameter optimization

Cohen's kappa as a function of IDW power

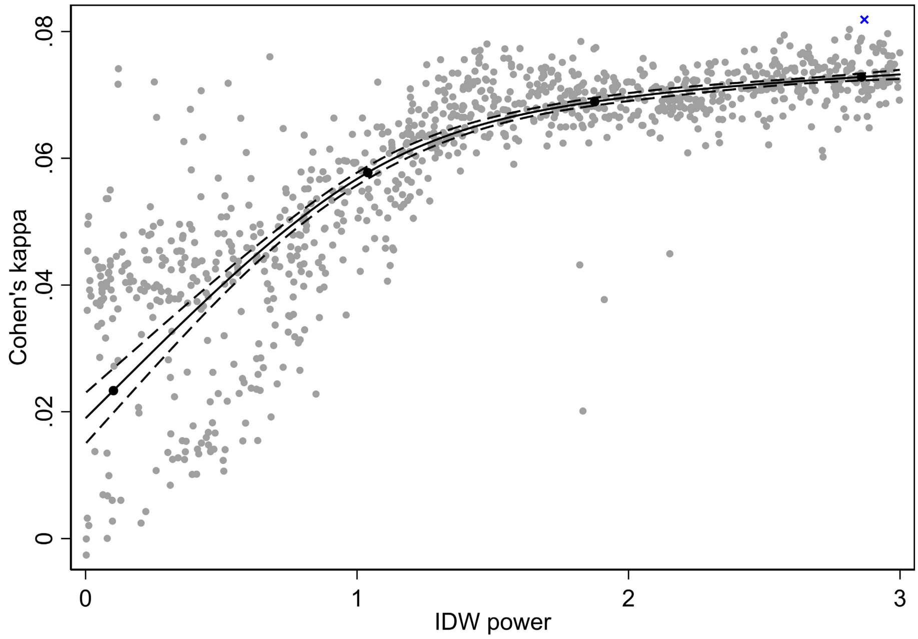

Each gray dot represents one crude analysis. The analyses used random time intervals and search radii. The blue × represents the analysis with the optimum combination of hyperparameters. The solid line represent the trend, modelled with restricted cubic splines with four knots (the location of the knots indicated by black dots). The dashed lines are the 95% confidence interval for the trend.

Cohen's kappa as a function of search radius

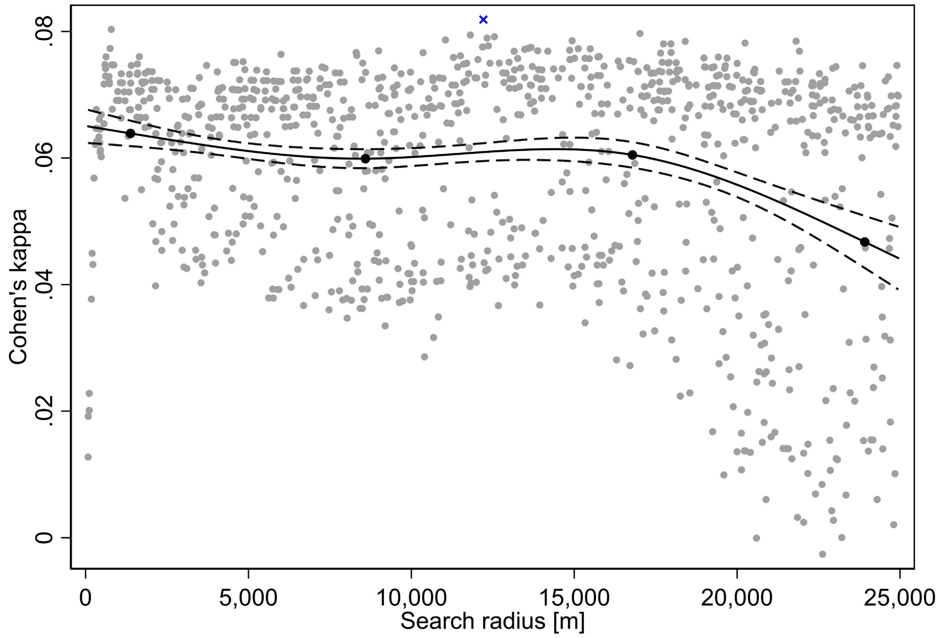

Each gray dot represents one crude analysis. The analyses used random time intervals and values of IDW power. The blue × represents the analysis with the optimum combination of hyperparameters. The solid line represent the trend, modelled with restricted cubic splines with four knots (the location of the knots indicated by black dots). The dashed lines are the 95% confidence interval for the trend.

Cohen's kappa as a function of time interval

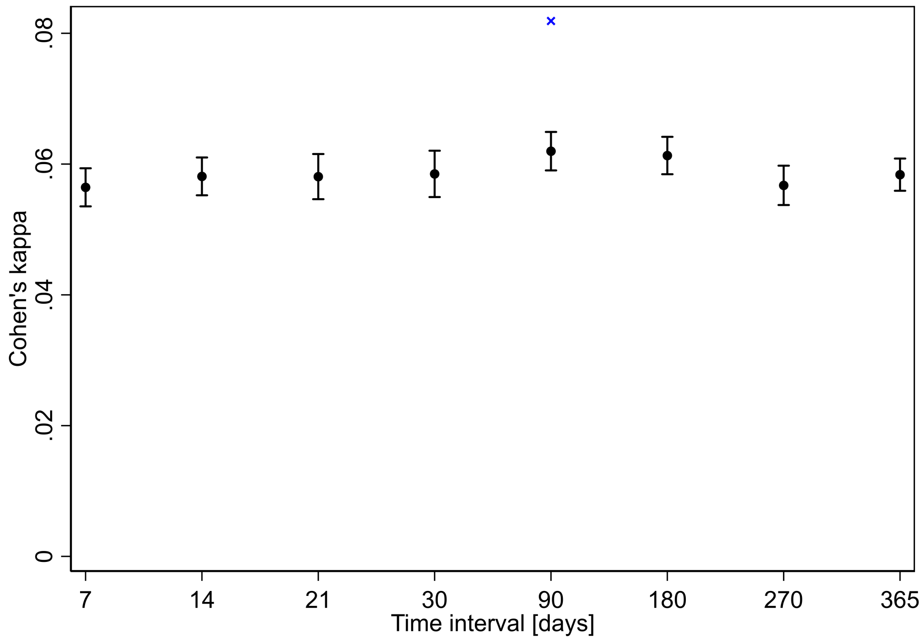

Each black dot represents the mean of the results for crude analyses with the indicated time interval. The analyses used random values of IDW power and search radius. The bars indicate the 95% confidence interval. The blue × represents the analysis with the optimum combination of hyperparameters.

Cohen's kappa as a function of search radius, IDW power and time interval

Time interval: 7 days

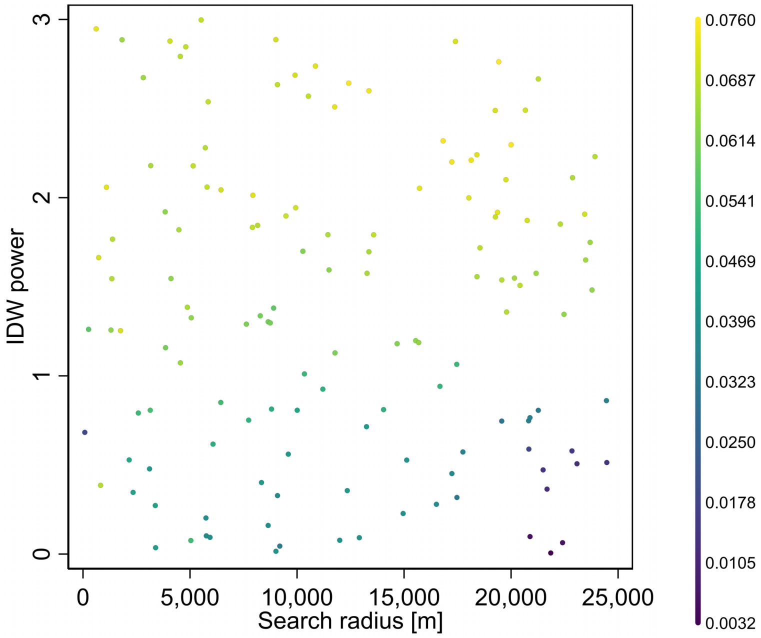

Each dot represents one crude analysis with the specified hyperparameters. Dots are color-coded according to the value of Cohen's kappa.

Time interval: 14 days

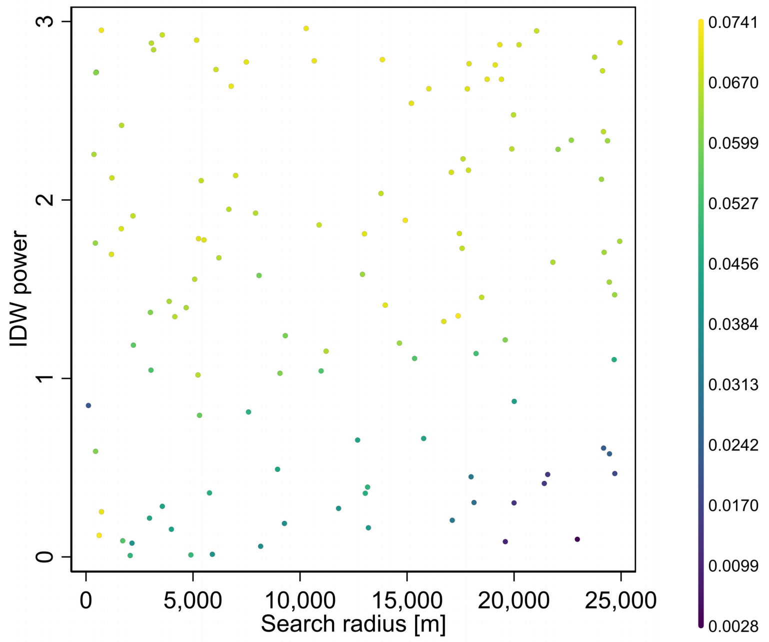

Each dot represents one crude analysis with the specified hyperparameters. Dots are color-coded according to the value of Cohen's kappa.

Time interval: 21 days

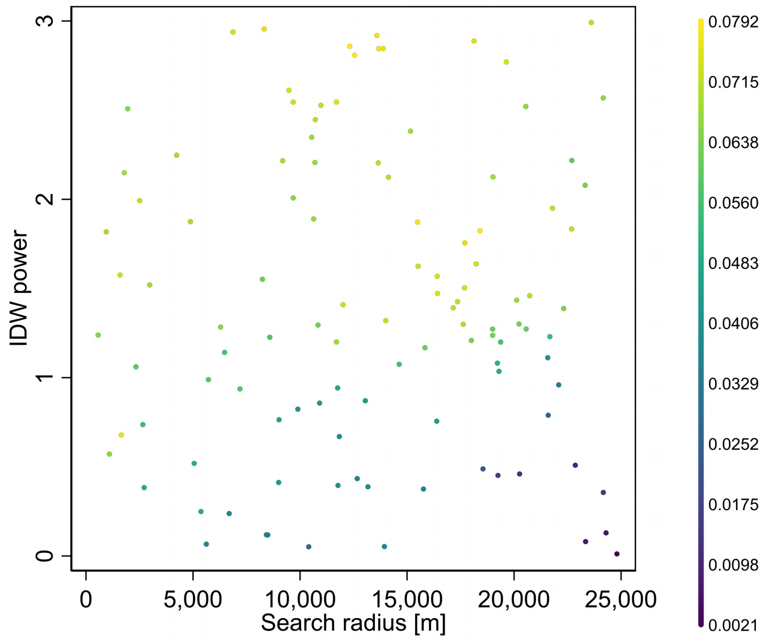

Each dot represents one crude analysis with the specified hyperparameters. Dots are color-coded according to the value of Cohen's kappa.

Time interval: 30 days

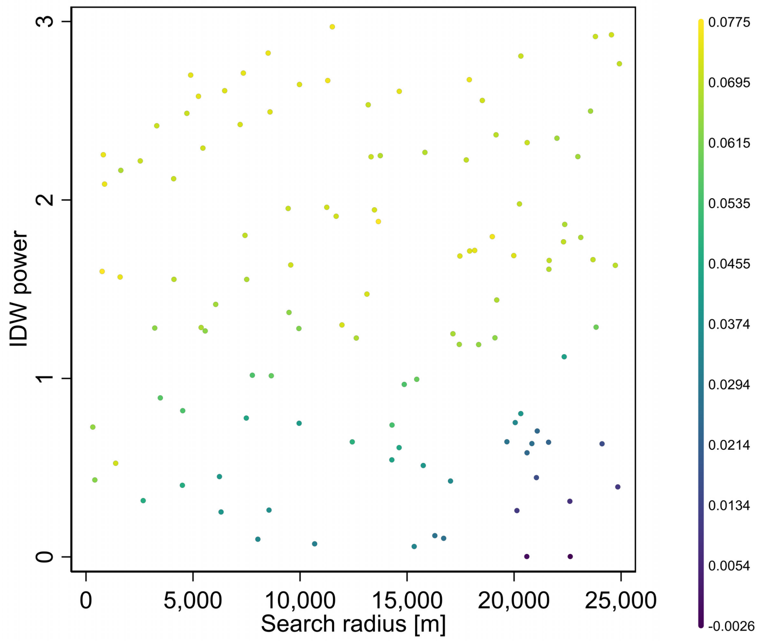

Each dot represents one crude analysis with the specified hyperparameters. Dots are color-coded according to the value of Cohen's kappa.

Time interval: 90 days

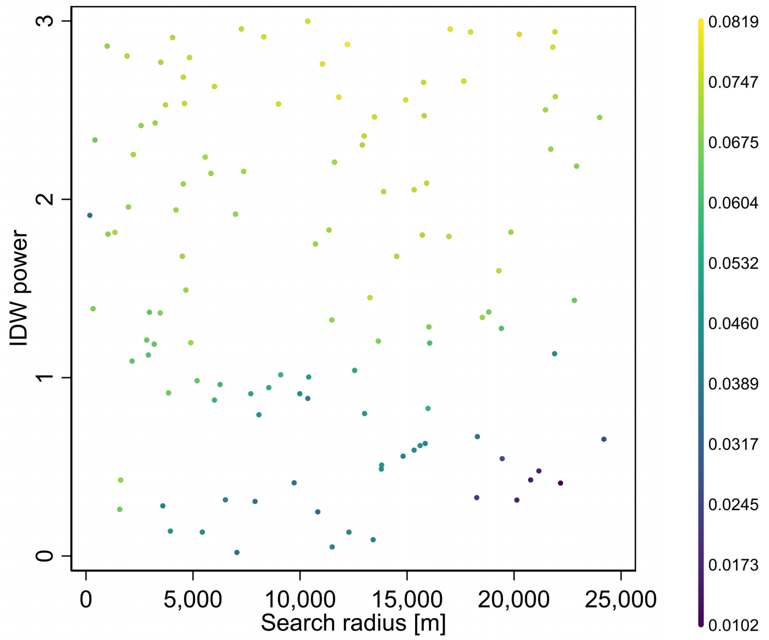

Each dot represents one crude analysis with the specified hyperparameters. Dots are color-coded according to the value of Cohen's kappa.

Time interval: 180 days

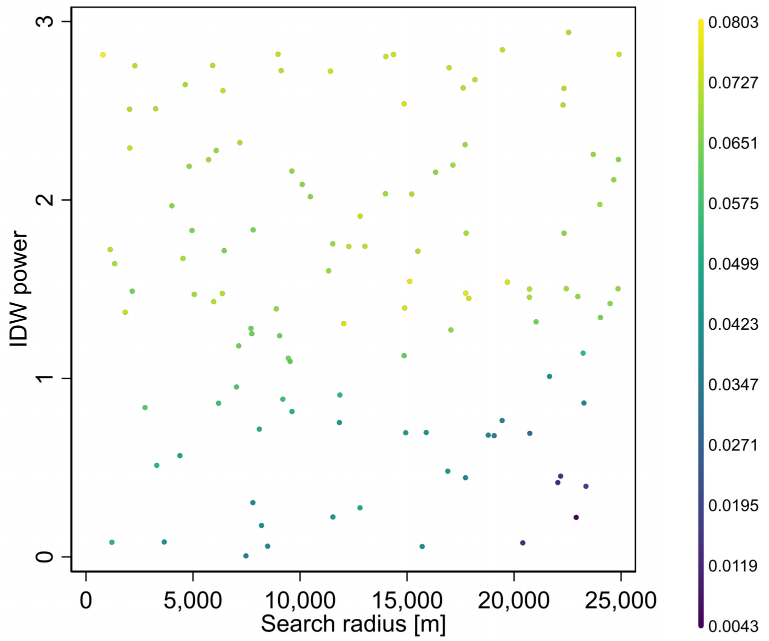

Each dot represents one crude analysis with the specified hyperparameters. Dots are color-coded according to the value of Cohen's kappa.

Time interval: 270 days

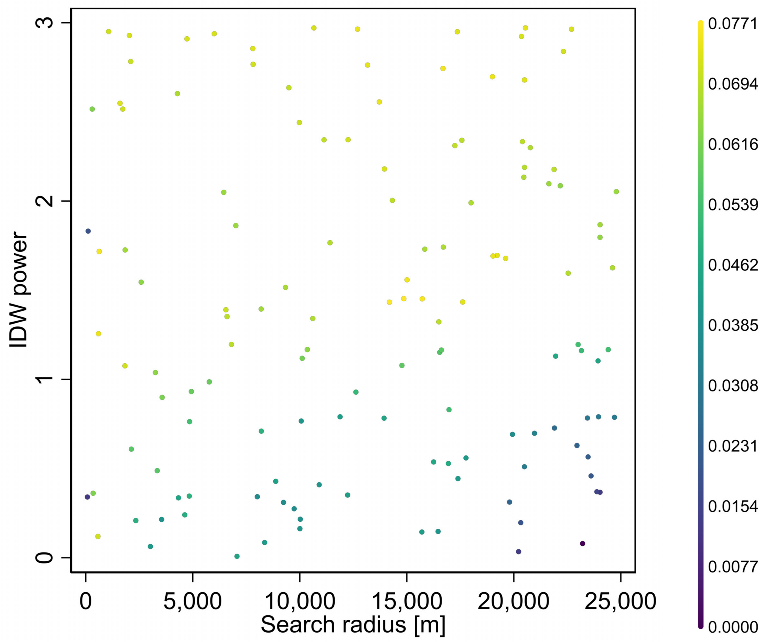

Each dot represents one crude analysis with the specified hyperparameters. Dots are color-coded according to the value of Cohen's kappa.

Time interval: 365 days

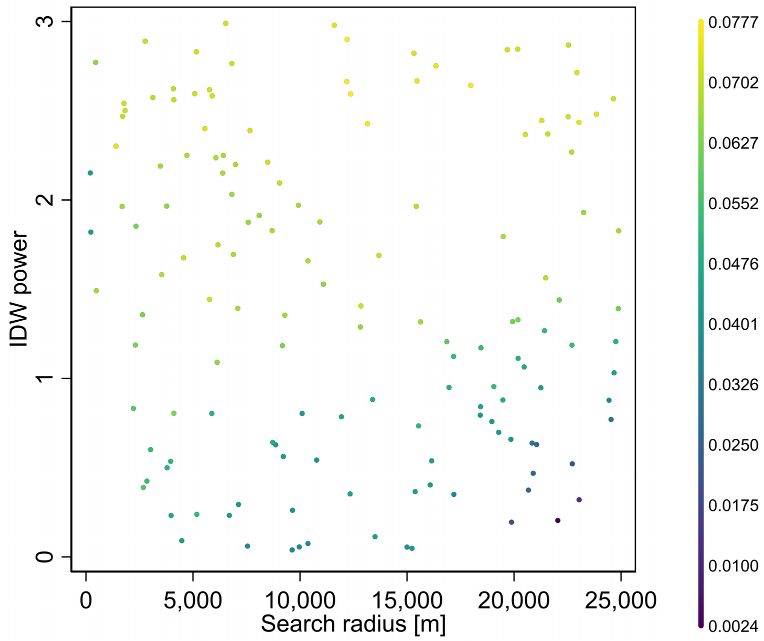

Each dot represents one crude analysis with the specified hyperparameters. Dots are color-coded according to the value of Cohen's kappa.

Time interval: Any

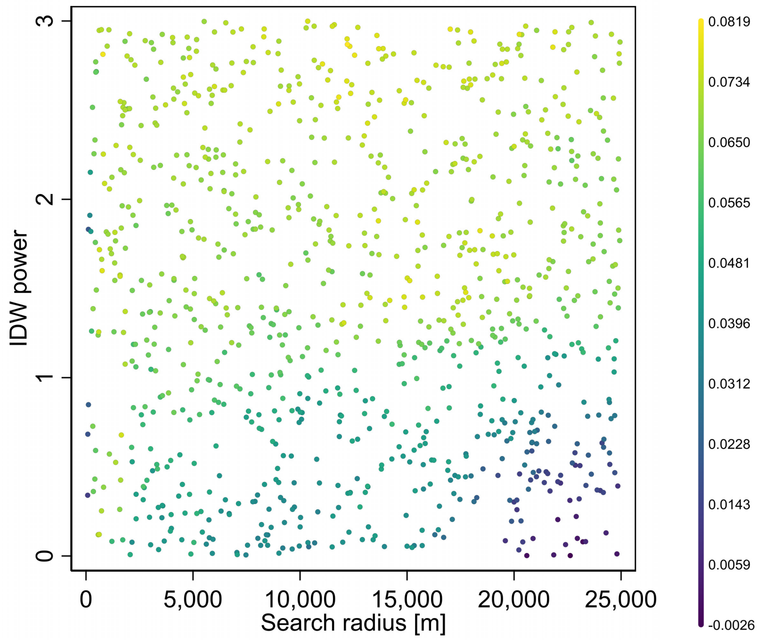

Each dot represents one crude analysis with the specified hyperparameters. Dots are color-coded according to the value of Cohen's kappa.

Analysis number 5

Primary results from this analysis

Crude analysis

Cohen's kappa as a function of h_{dw}

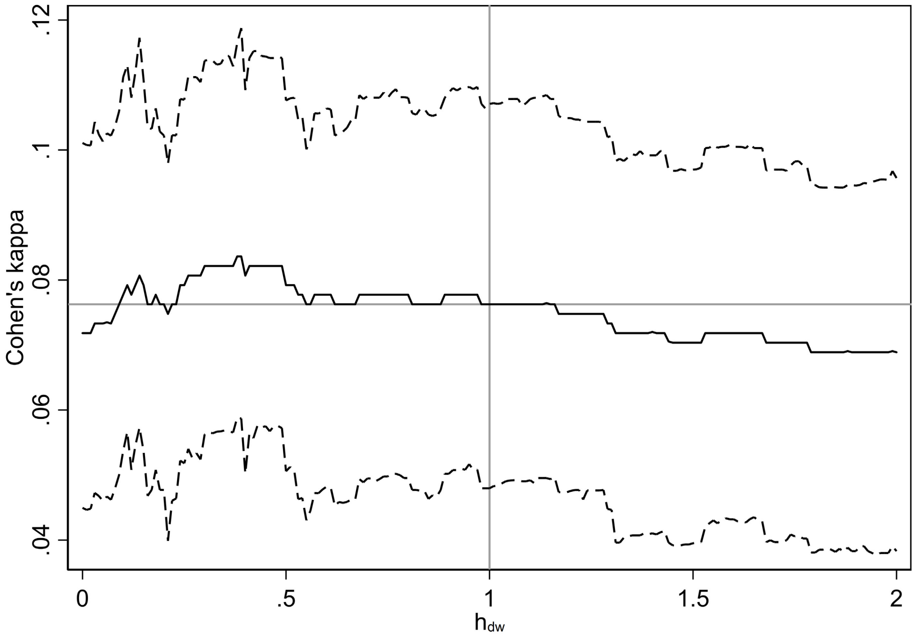

Controls matched to cases by age, gender and municipality of recidence.
Solid line is estimate, dashed lines are 95% confidence interval.

Δ(Cohen's kappa) as a function of h_{dw}

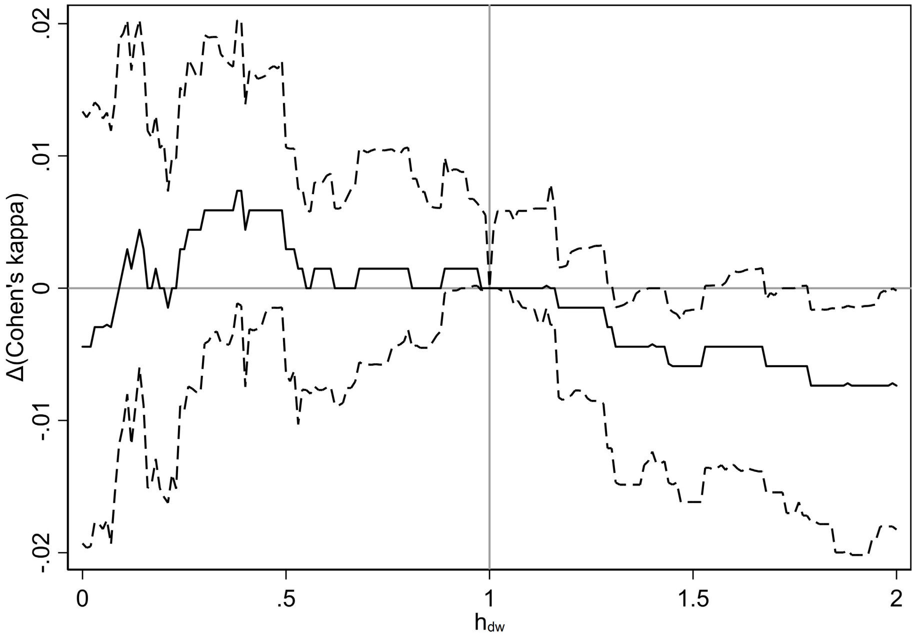

Controls matched to cases by age, gender and municipality of recidence.
Solid line is estimate, dashed lines are 95% confidence interval.

Adjusted analysis

Cohen's kappa as a function of h_{dw}

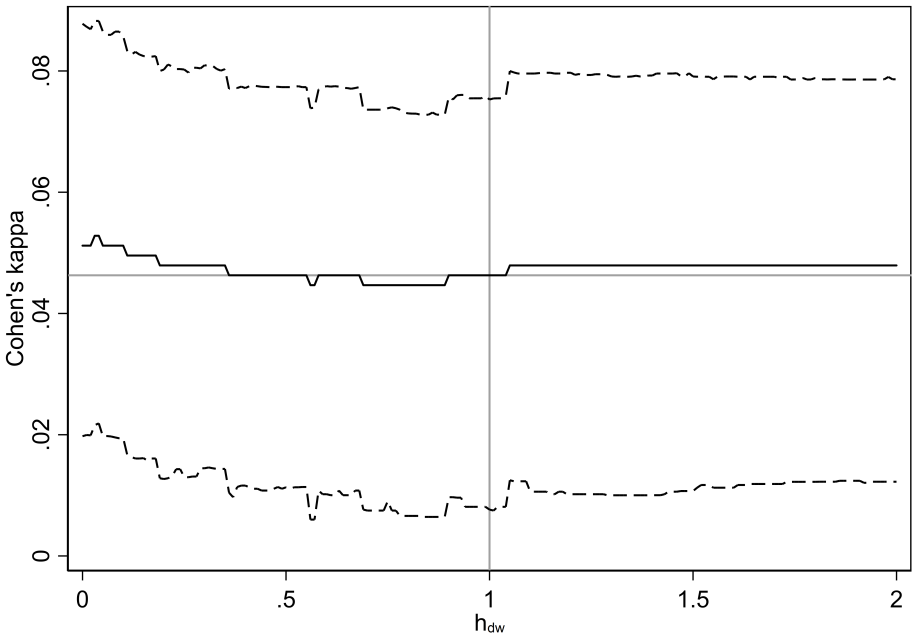

Controls matched to cases by age, gender and municipality of recidence. Analysis adjusted for age, gender, education and income.
Solid line is estimate, dashed lines are 95% confidence interval.

Δ(Cohen's kappa) as a function of h_{dw}

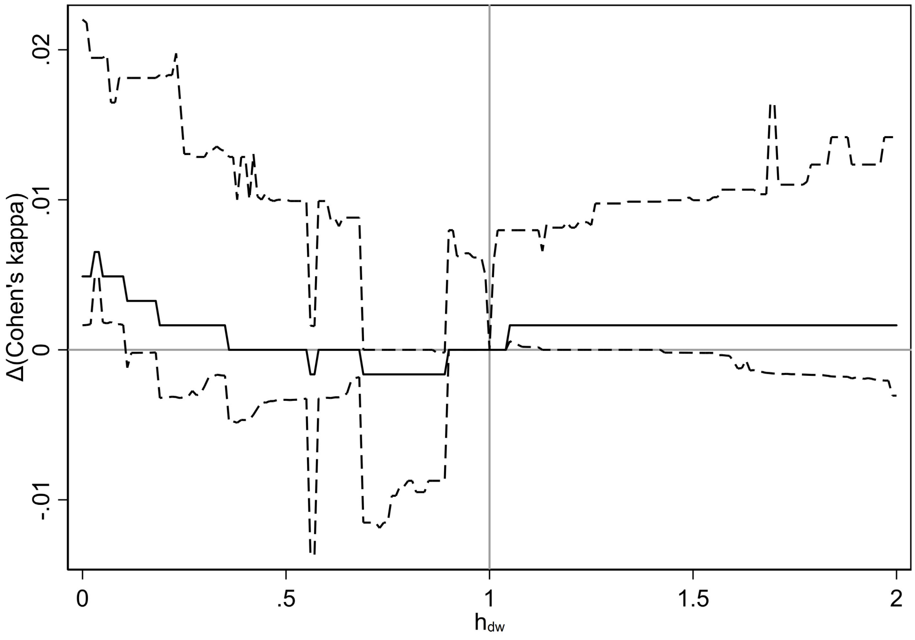

Controls matched to cases by age, gender and municipality of recidence. Analysis adjusted for age, gender, education and income.
Solid line is estimate, dashed lines are 95% confidence interval.

Secondary results for this analysis: Description of hyperparameter optimization

Cohen's kappa as a function of IDW power

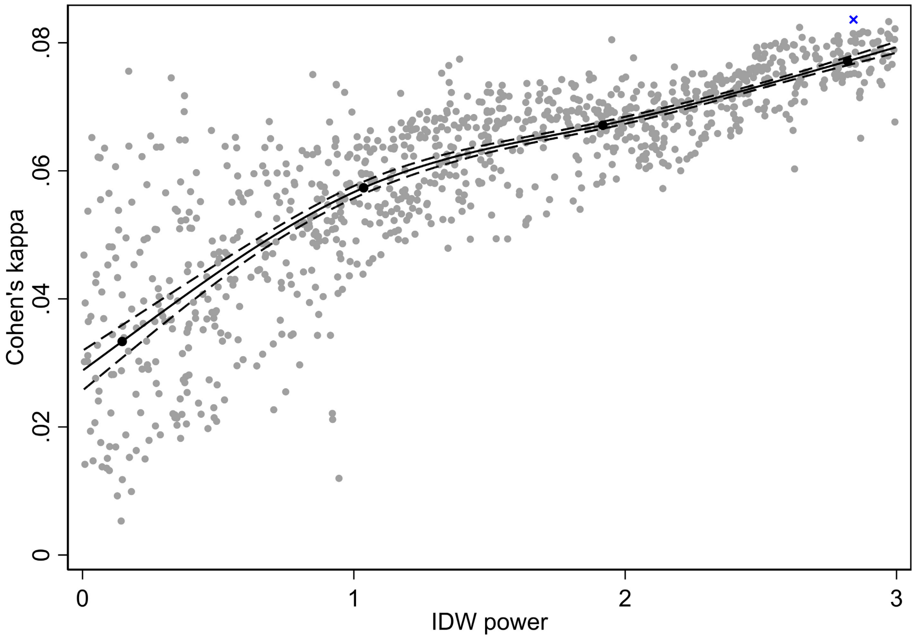

Each gray dot represents one crude analysis. The analyses used random time intervals and search radii. The blue × represents the analysis with the optimum combination of hyperparameters. The solid line represent the trend, modelled with restricted cubic splines with four knots (the location of the knots indicated by black dots). The dashed lines are the 95% confidence interval for the trend.

Cohen's kappa as a function of search radius

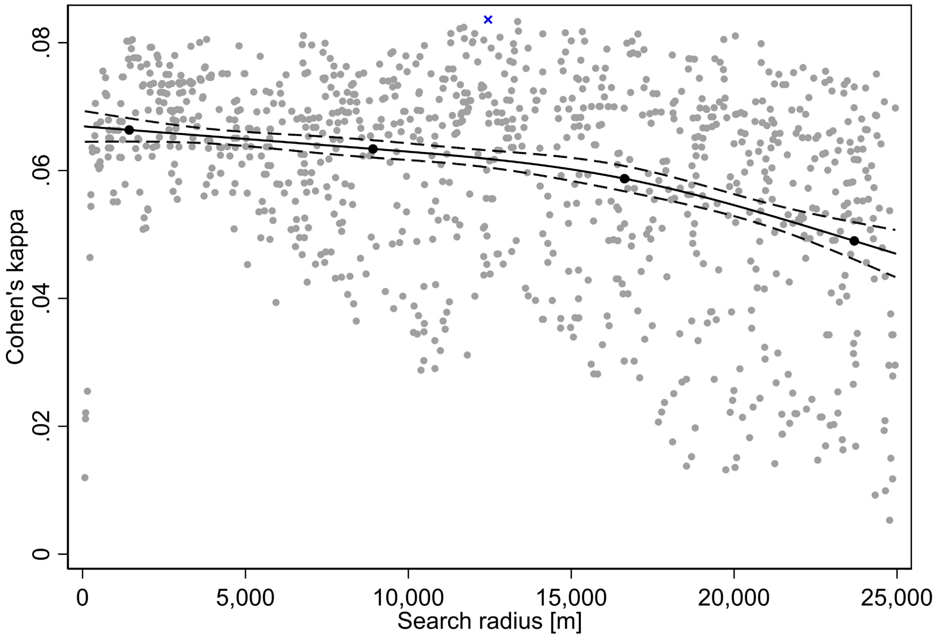

Each gray dot represents one crude analysis. The analyses used random time intervals and values of IDW power. The blue × represents the analysis with the optimum combination of hyperparameters. The solid line represent the trend, modelled with restricted cubic splines with four knots (the location of the knots indicated by black dots). The dashed lines are the 95% confidence interval for the trend.

Cohen's kappa as a function of time interval

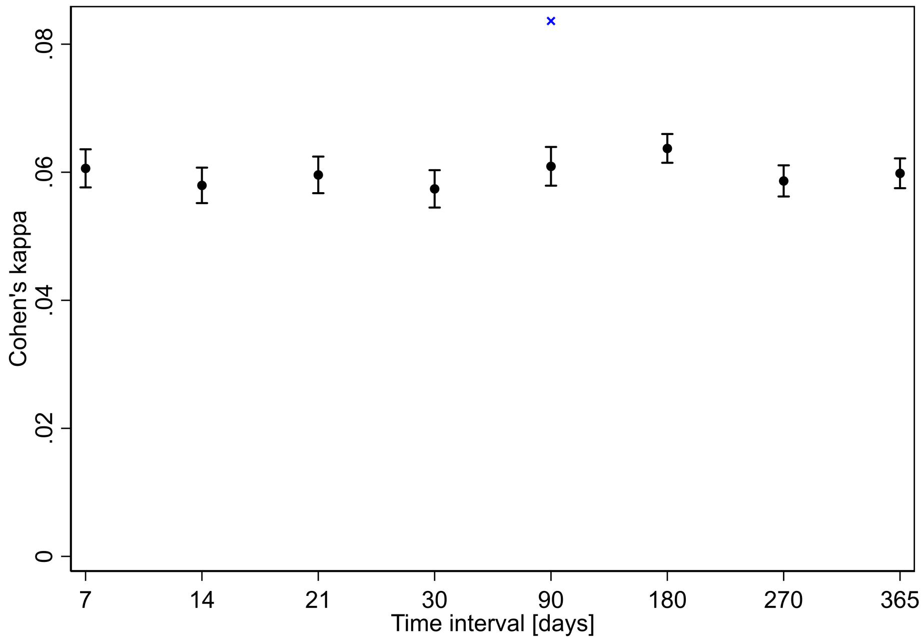

Each black dot represents the mean of the results for crude analyses with the indicated time interval. The analyses used random values of IDW power and search radius. The bars indicate the 95% confidence interval. The blue × represents the analysis with the optimum combination of hyperparameters.

Cohen's kappa as a function of search radius, IDW power and time interval

Time interval: 7 days

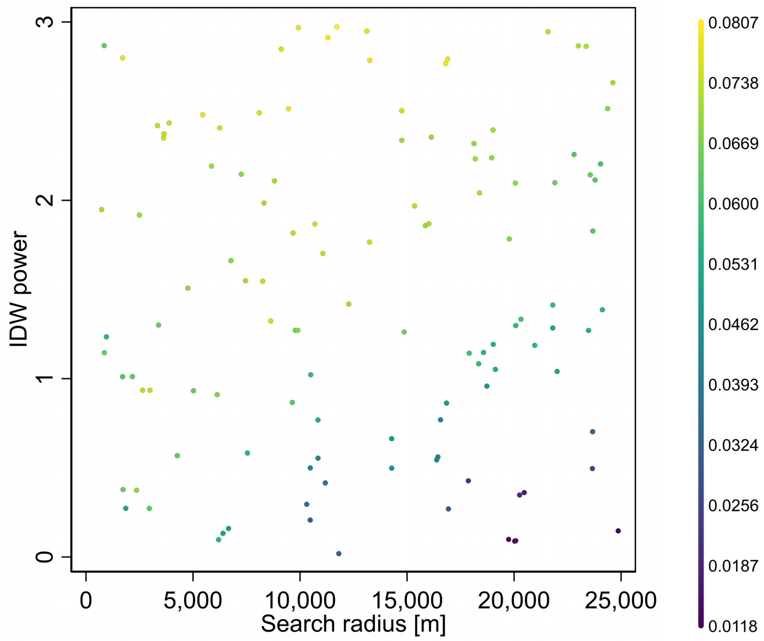

Each dot represents one crude analysis with the specified hyperparameters. Dots are color-coded according to the value of Cohen's kappa.

Time interval: 14 days

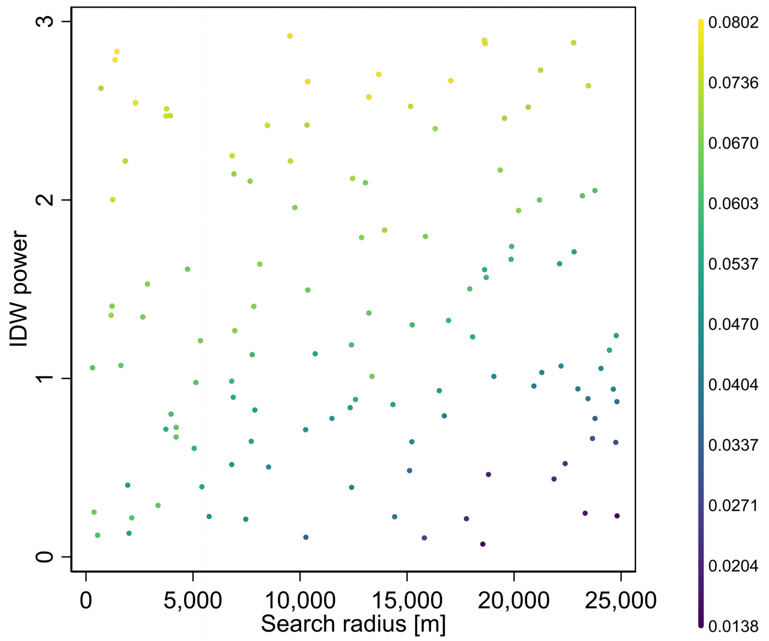

Each dot represents one crude analysis with the specified hyperparameters. Dots are color-coded according to the value of Cohen's kappa.

Time interval: 21 days

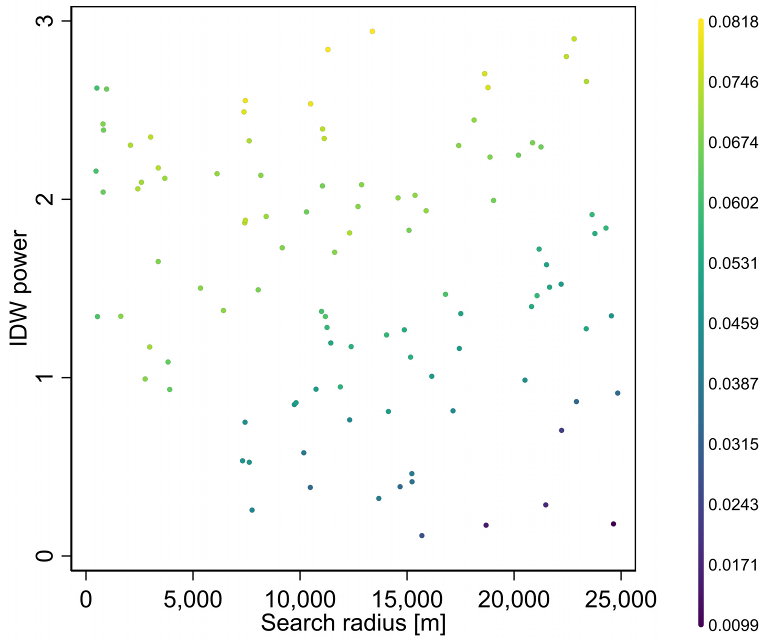

Each dot represents one crude analysis with the specified hyperparameters. Dots are color-coded according to the value of Cohen's kappa.

Time interval: 30 days

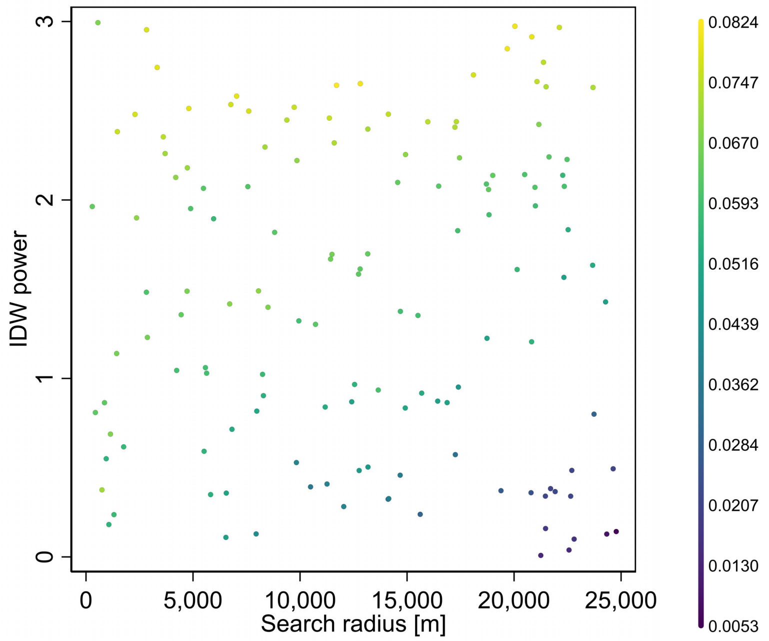

Each dot represents one crude analysis with the specified hyperparameters. Dots are color-coded according to the value of Cohen's kappa.

Time interval: 90 days

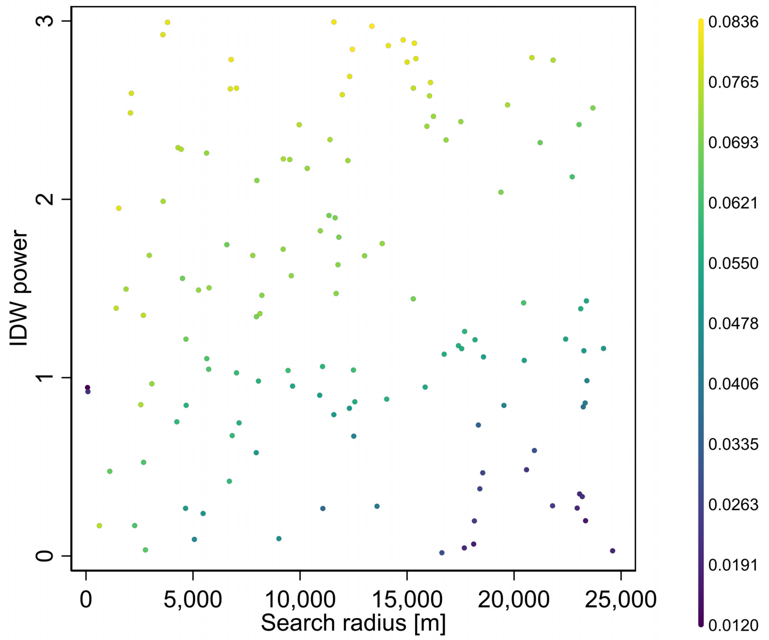

Each dot represents one crude analysis with the specified hyperparameters. Dots are color-coded according to the value of Cohen's kappa.

Time interval: 180 days

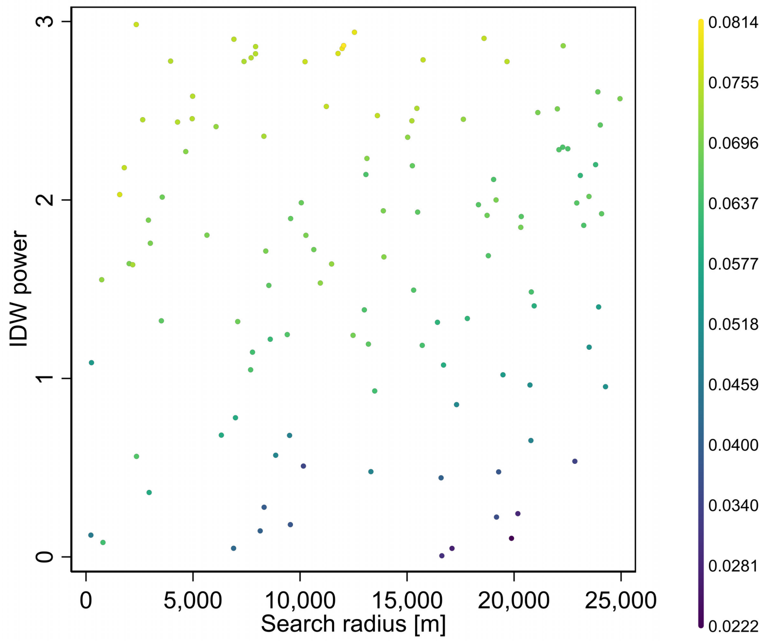

Each dot represents one crude analysis with the specified hyperparameters. Dots are color-coded according to the value of Cohen's kappa.

Time interval: 270 days

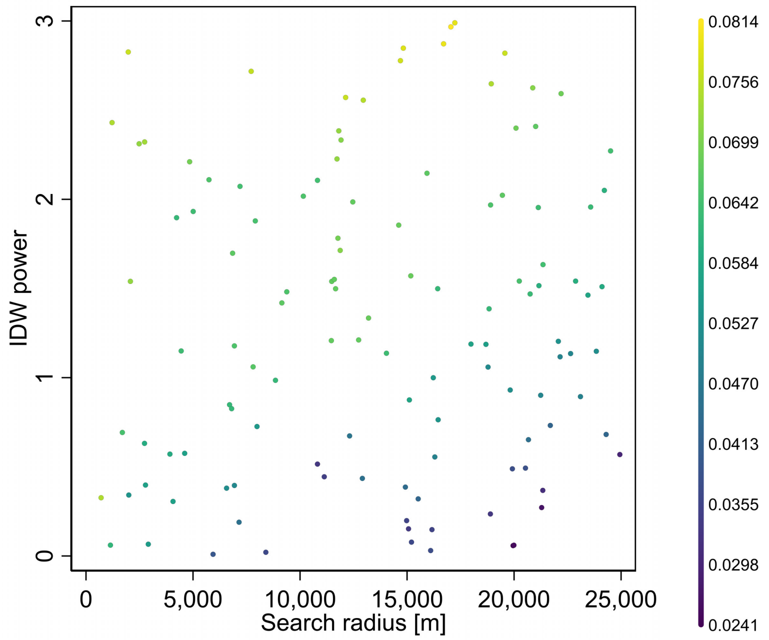

Each dot represents one crude analysis with the specified hyperparameters. Dots are color-coded according to the value of Cohen's kappa.

Time interval: 365 days

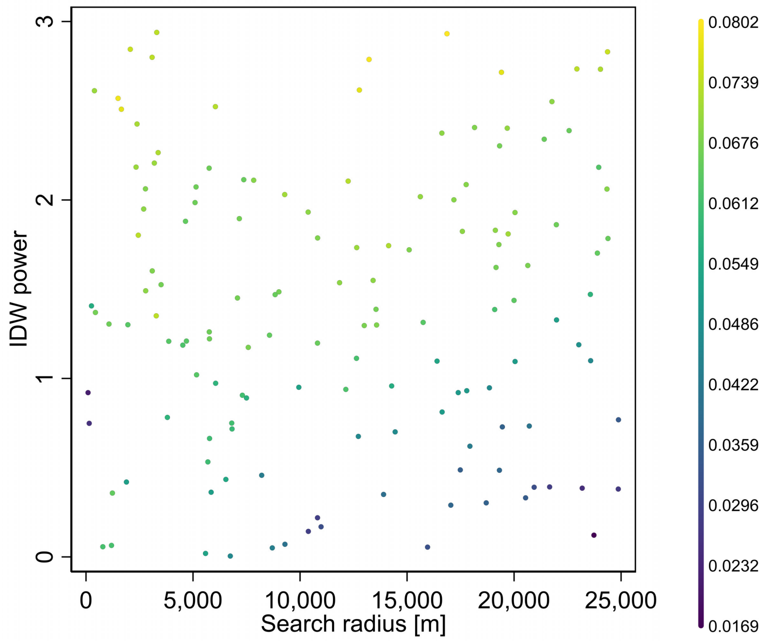

Each dot represents one crude analysis with the specified hyperparameters. Dots are color-coded according to the value of Cohen's kappa.

Time interval: Any

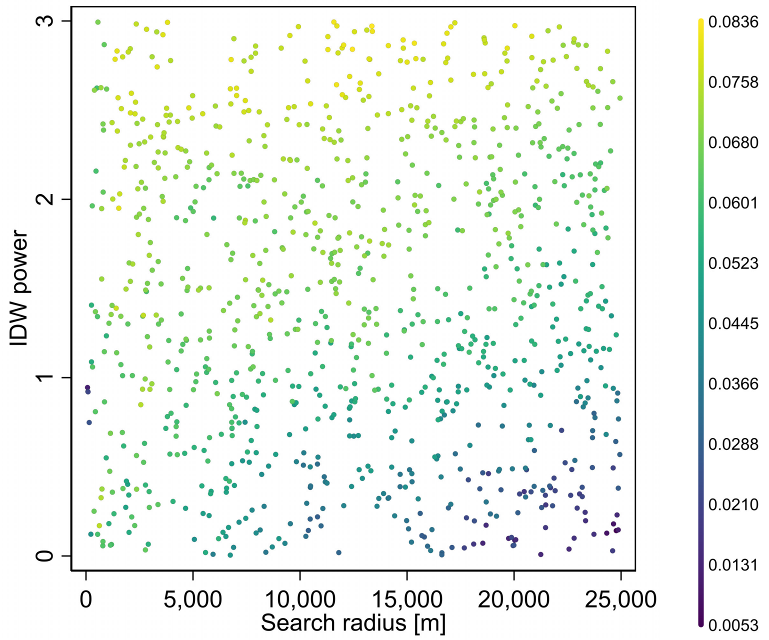

Each dot represents one crude analysis with the specified hyperparameters. Dots are color-coded according to the value of Cohen's kappa.

Analysis number 6

Primary results from this analysis

Crude analysis

Cohen's kappa as a function of h_{dw}

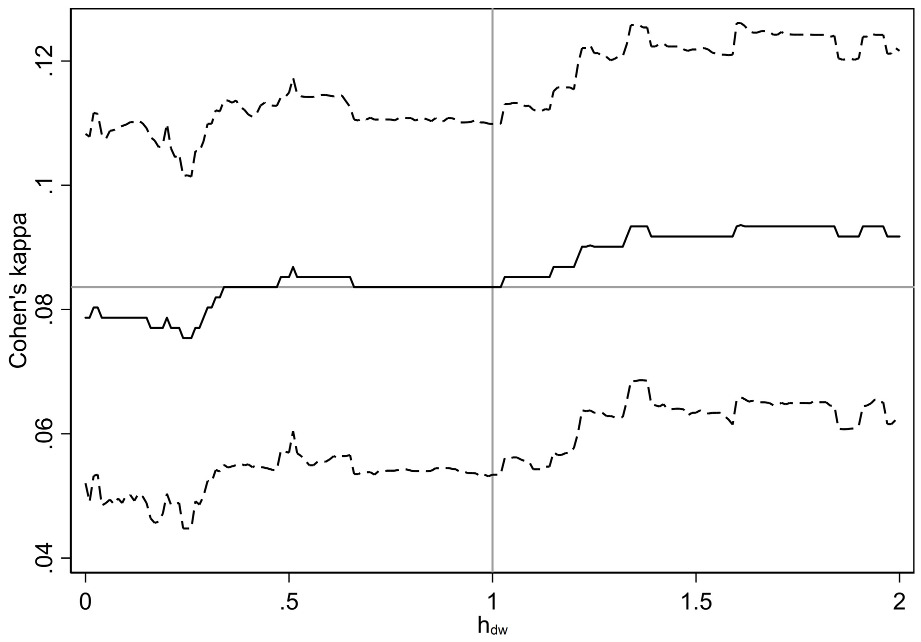

Controls matched to cases by age, gender and municipality of recidence.
Solid line is estimate, dashed lines are 95% confidence interval.

Δ(Cohen's kappa) as a function of h_{dw}

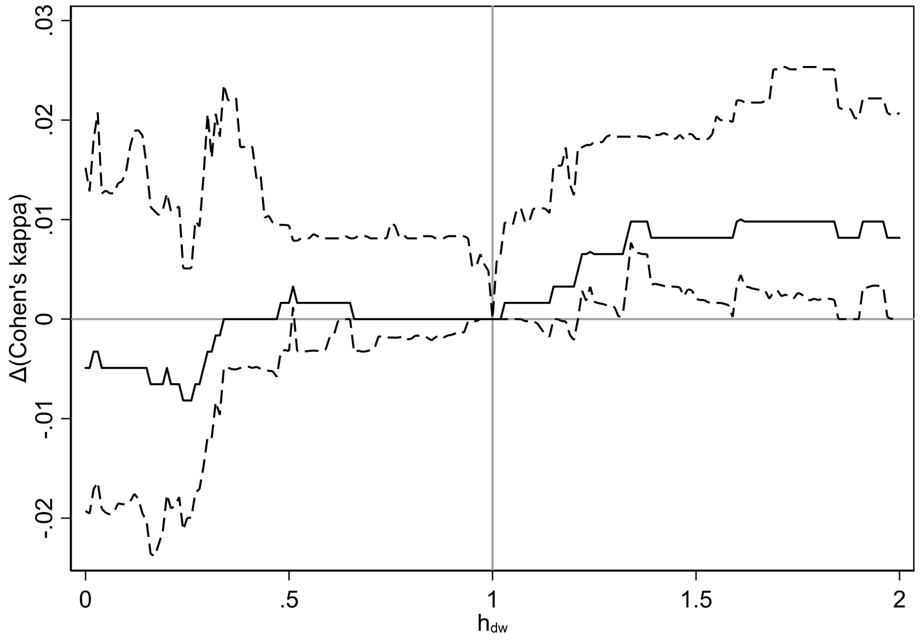

Controls matched to cases by age, gender and municipality of recidence.
Solid line is estimate, dashed lines are 95% confidence interval.

Adjusted analysis

Cohen's kappa as a function of h_{dw}

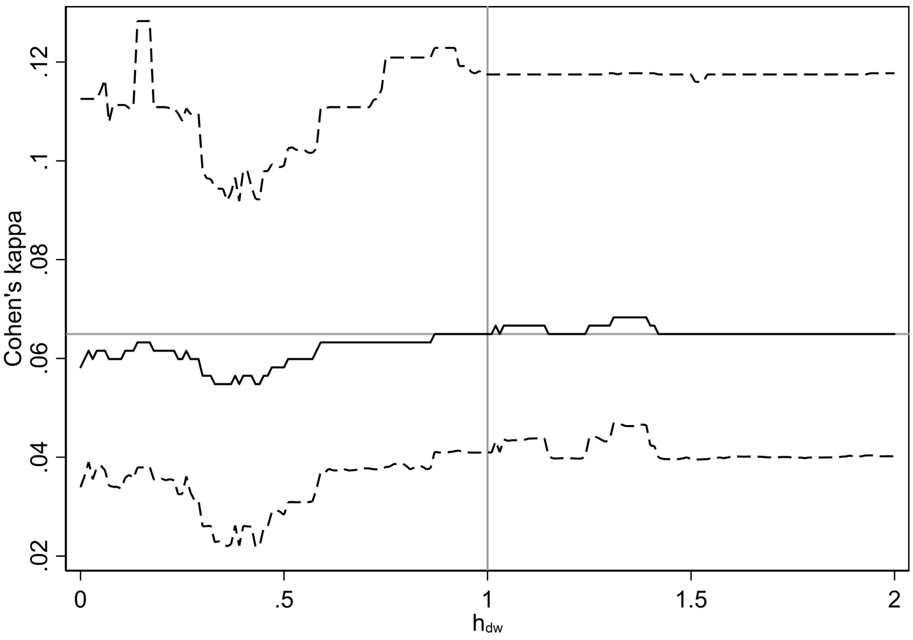

Controls matched to cases by age, gender and municipality of recidence. Analysis adjusted for age, gender, education and income.
Solid line is estimate, dashed lines are 95% confidence interval.

Δ(Cohen's kappa) as a function of h_{dw}

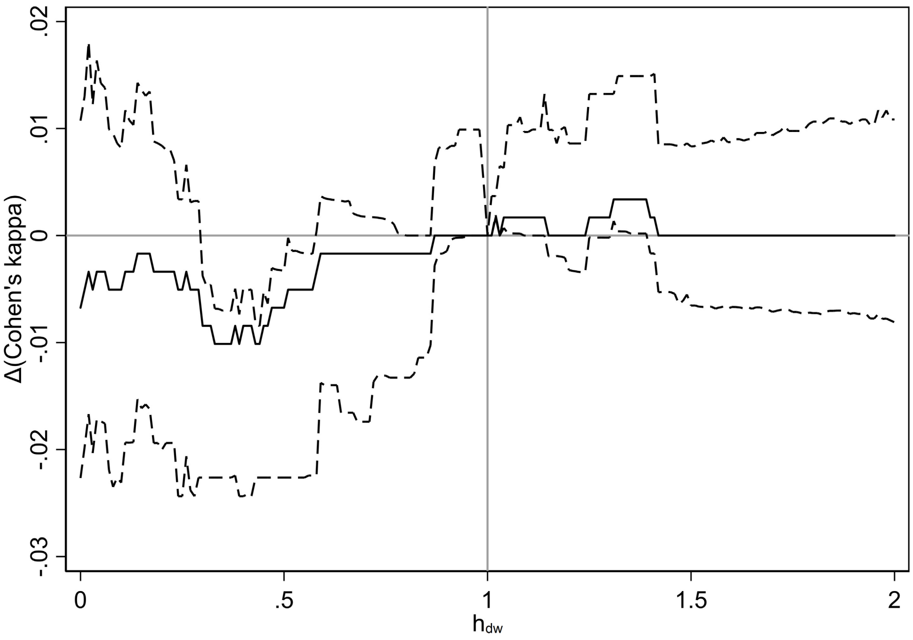

Controls matched to cases by age, gender and municipality of recidence. Analysis adjusted for age, gender, education and income.
Solid line is estimate, dashed lines are 95% confidence interval.

Secondary results for this analysis: Description of hyperparameter optimization

Cohen's kappa as a function of IDW power

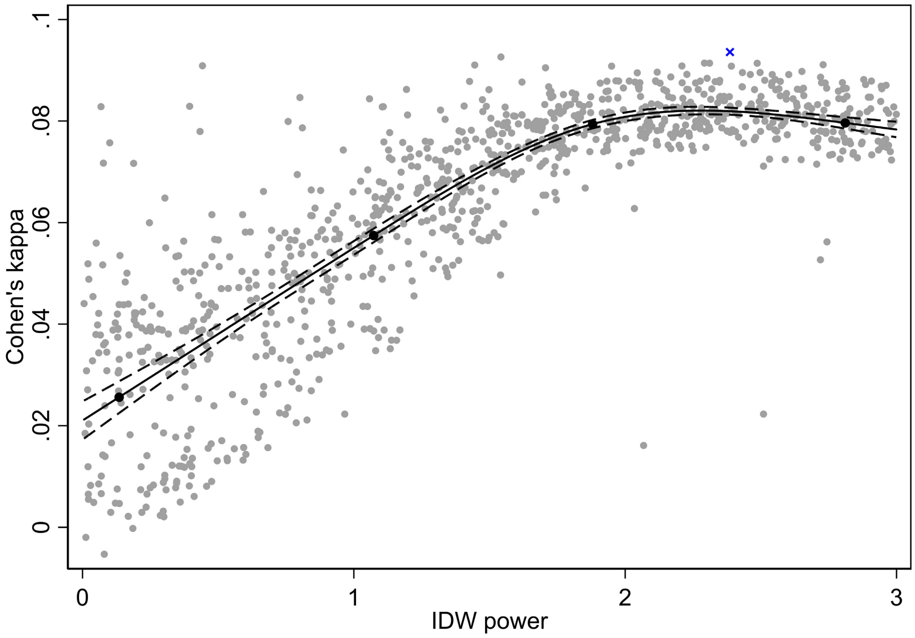

Each gray dot represents one crude analysis. The analyses used random time intervals and search radii. The blue × represents the analysis with the optimum combination of hyperparameters. The solid line represent the trend, modelled with restricted cubic splines with four knots (the location of the knots indicated by black dots). The dashed lines are the 95% confidence interval for the trend.

Cohen's kappa as a function of search radius

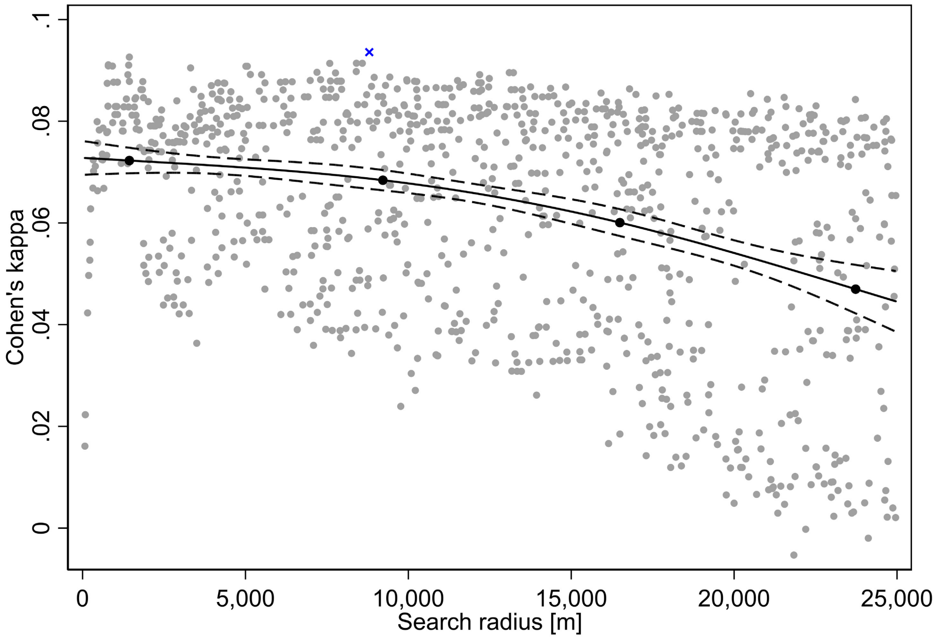

Each gray dot represents one crude analysis. The analyses used random time intervals and values of IDW power. The blue × represents the analysis with the optimum combination of hyperparameters. The solid line represent the trend, modelled with restricted cubic splines with four knots (the location of the knots indicated by black dots). The dashed lines are the 95% confidence interval for the trend.

Cohen's kappa as a function of time interval

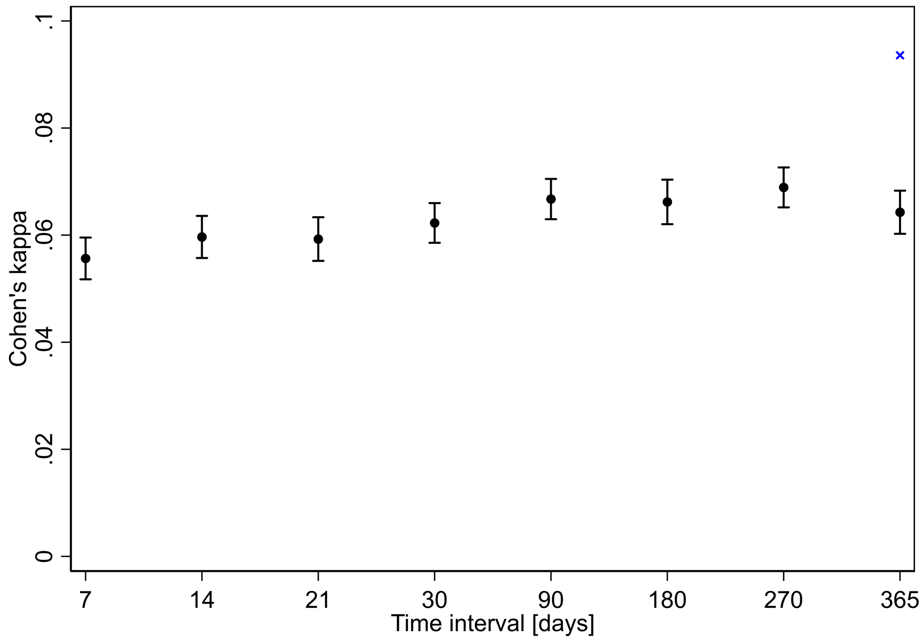

Each black dot represents the mean of the results for crude analyses with the indicated time interval. The analyses used random values of IDW power and search radius. The bars indicate the 95% confidence interval. The blue × represents the analysis with the optimum combination of hyperparameters.

Cohen's kappa as a function of search radius, IDW power and time interval

Time interval: 7 days

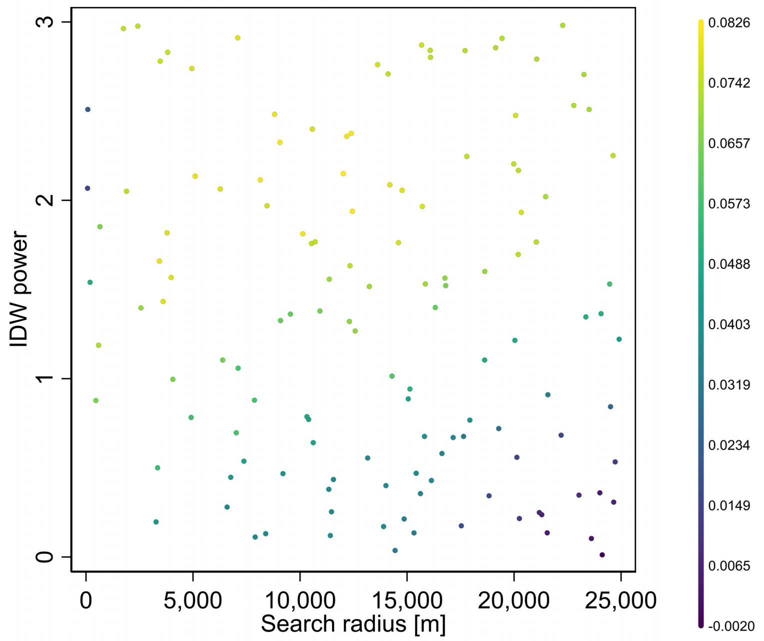

Each dot represents one crude analysis with the specified hyperparameters. Dots are color-coded according to the value of Cohen's kappa.

Time interval: 14 days

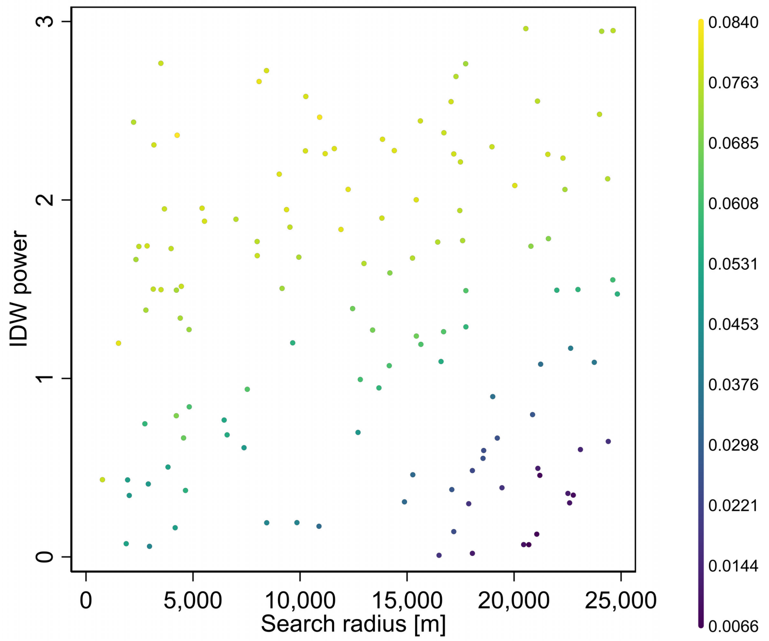

Each dot represents one crude analysis with the specified hyperparameters. Dots are color-coded according to the value of Cohen's kappa.

Time interval: 21 days

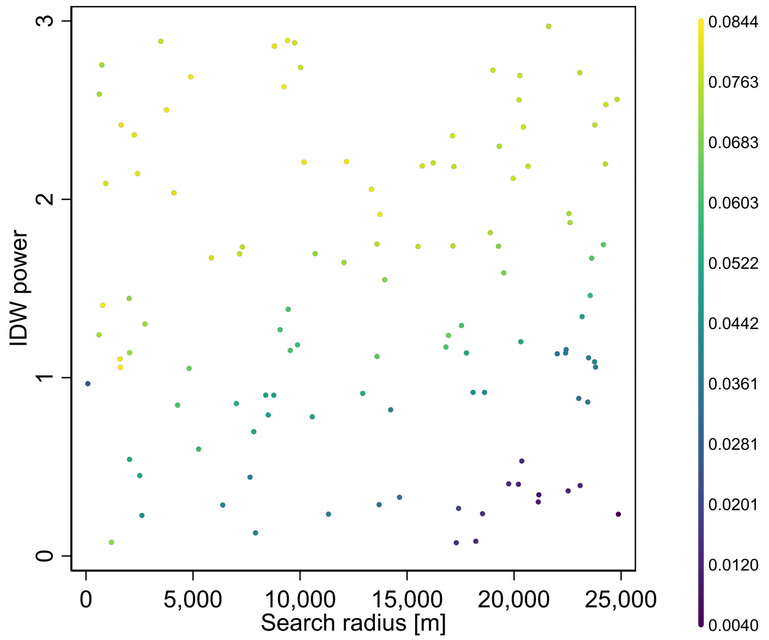

Each dot represents one crude analysis with the specified hyperparameters. Dots are color-coded according to the value of Cohen's kappa.

Time interval: 30 days

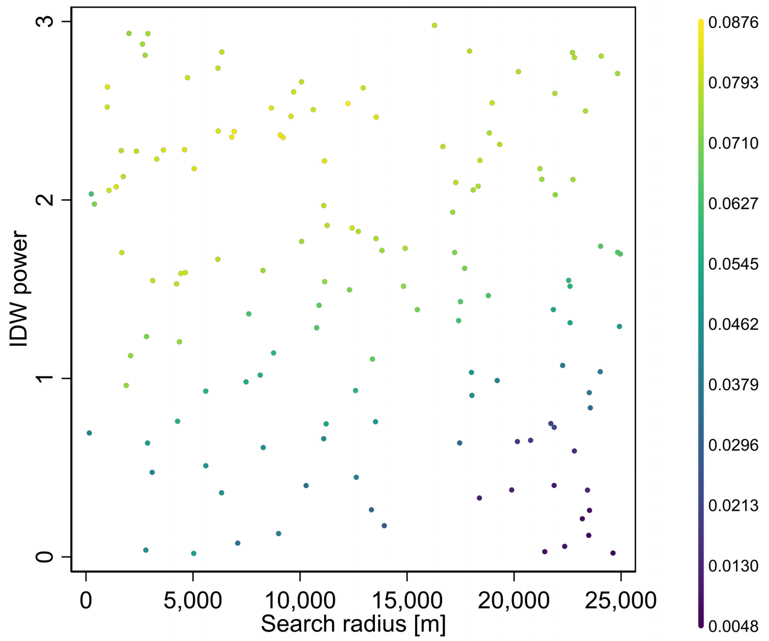

Each dot represents one crude analysis with the specified hyperparameters. Dots are color-coded according to the value of Cohen's kappa.

Time interval: 90 days

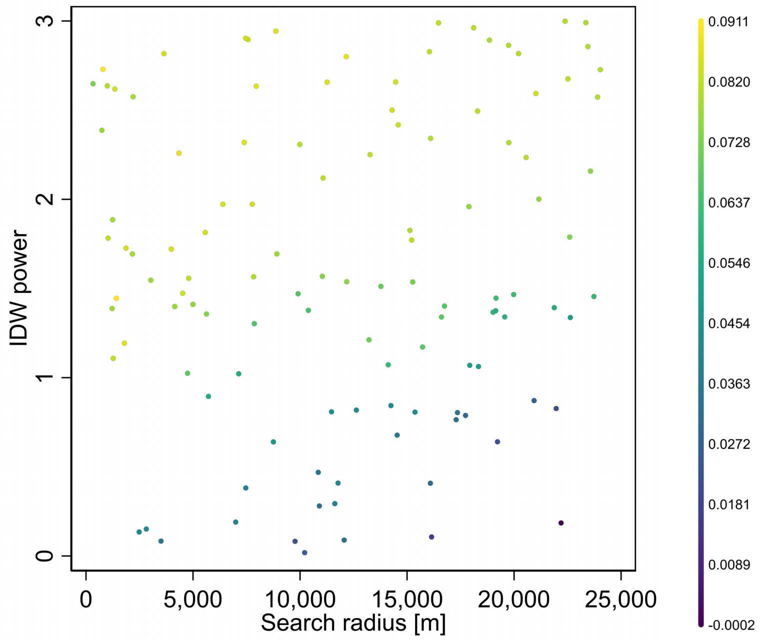

Each dot represents one crude analysis with the specified hyperparameters. Dots are color-coded according to the value of Cohen's kappa.

Time interval: 180 days

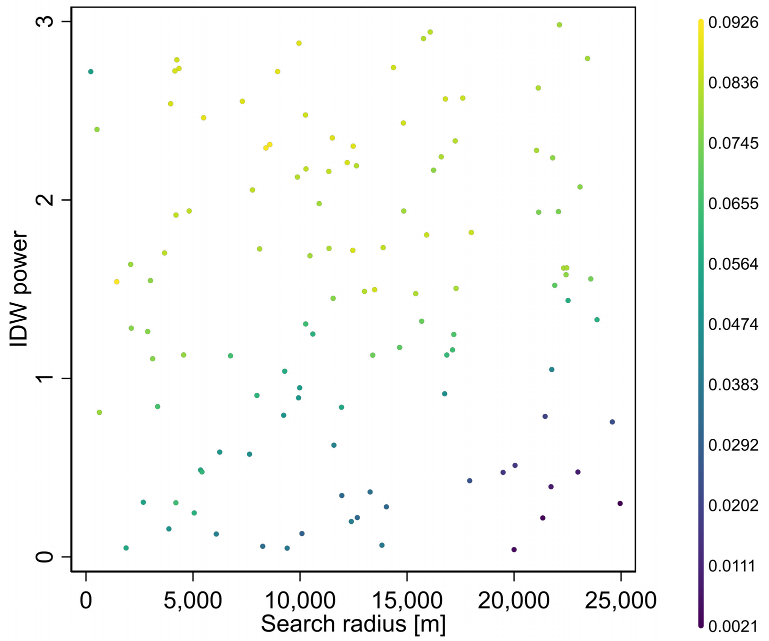

Each dot represents one crude analysis with the specified hyperparameters. Dots are color-coded according to the value of Cohen's kappa.

Time interval: 270 days

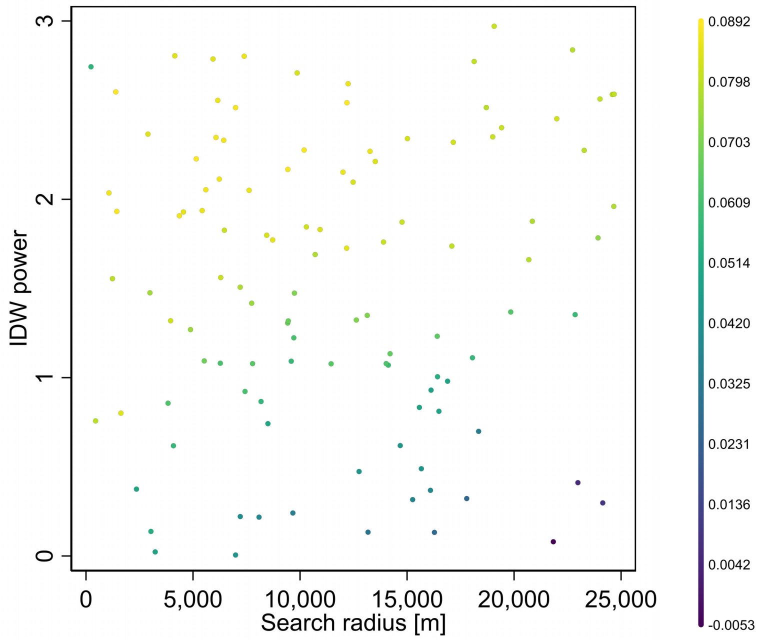

Each dot represents one crude analysis with the specified hyperparameters. Dots are color-coded according to the value of Cohen's kappa.

Time interval: 365 days

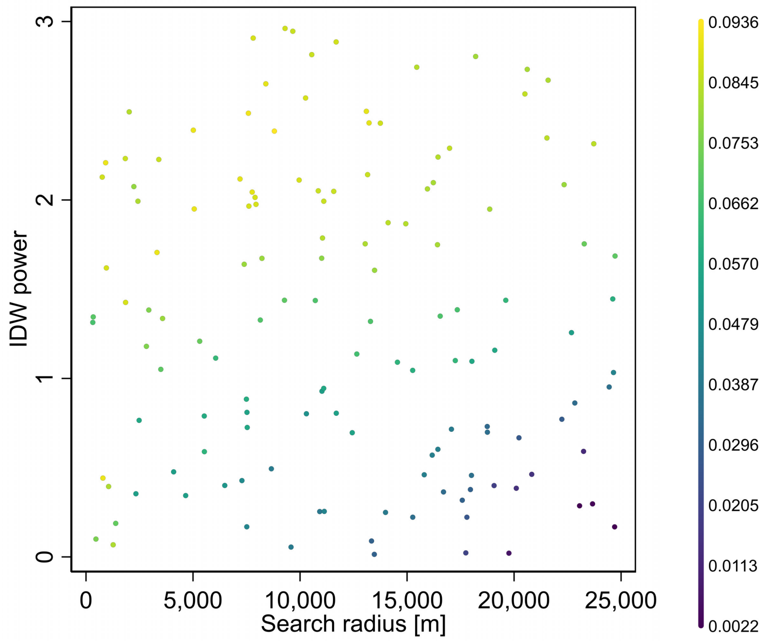

Each dot represents one crude analysis with the specified hyperparameters. Dots are color-coded according to the value of Cohen's kappa.

Time interval: Any

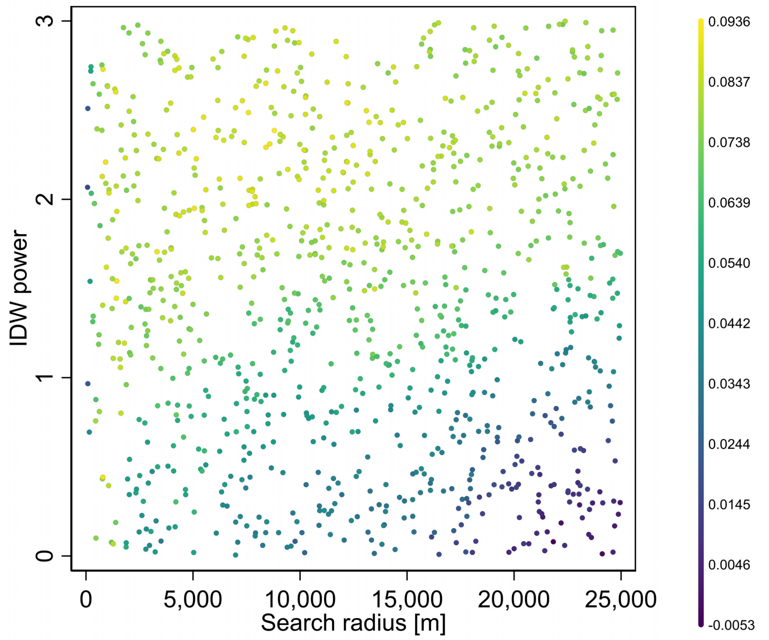

Each dot represents one crude analysis with the specified hyperparameters. Dots are color-coded according to the value of Cohen's kappa.

Analysis number 7

Primary results from this analysis

Crude analysis

Cohen's kappa as a function of h_{dw}

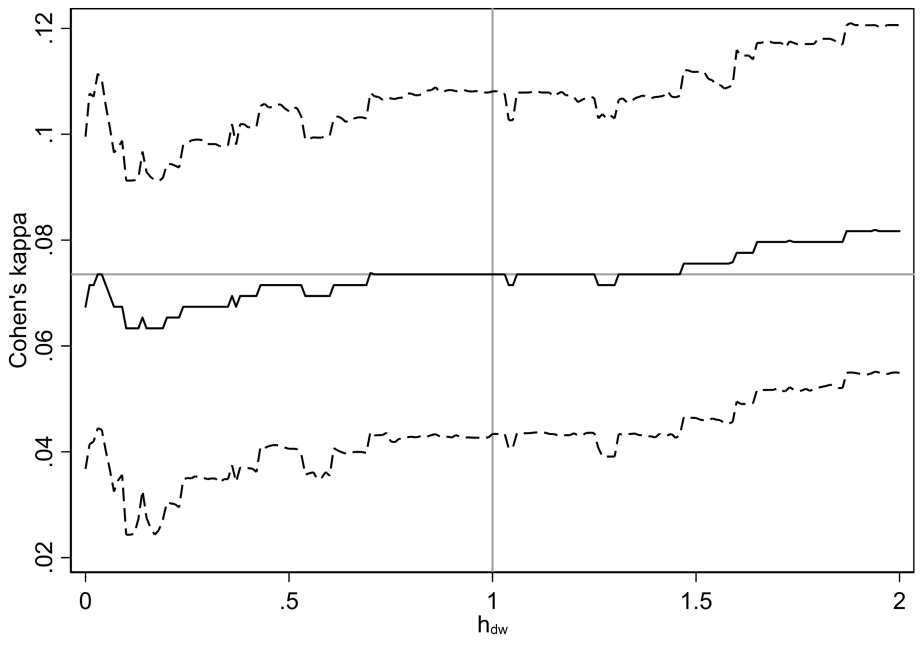

Controls matched to cases by age, gender and municipality of recidence.
Solid line is estimate, dashed lines are 95% confidence interval.

Δ(Cohen's kappa) as a function of h_{dw}

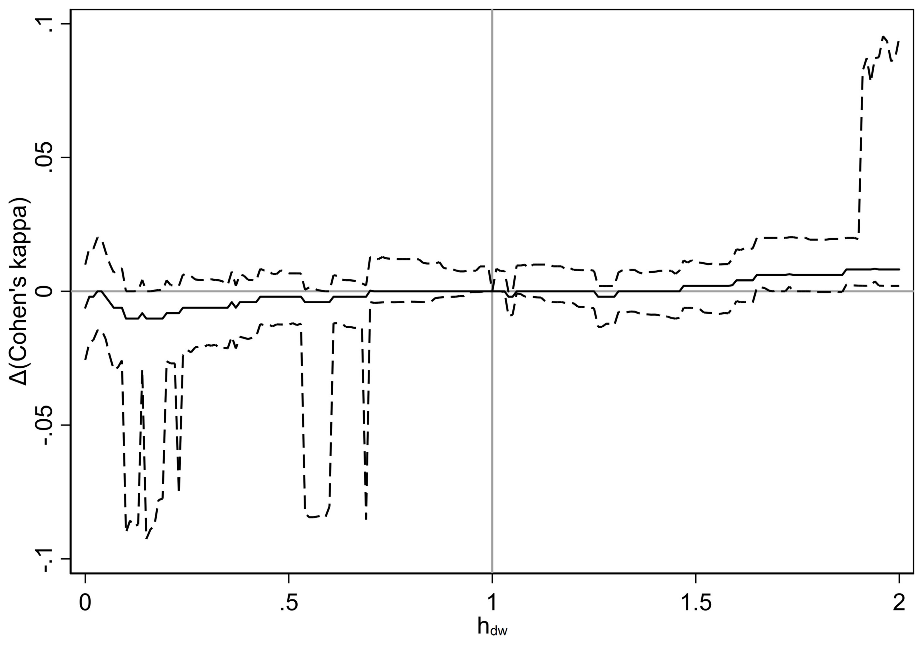

Controls matched to cases by age, gender and municipality of recidence.
Solid line is estimate, dashed lines are 95% confidence interval.

Adjusted analysis

Cohen's kappa as a function of h_{dw}

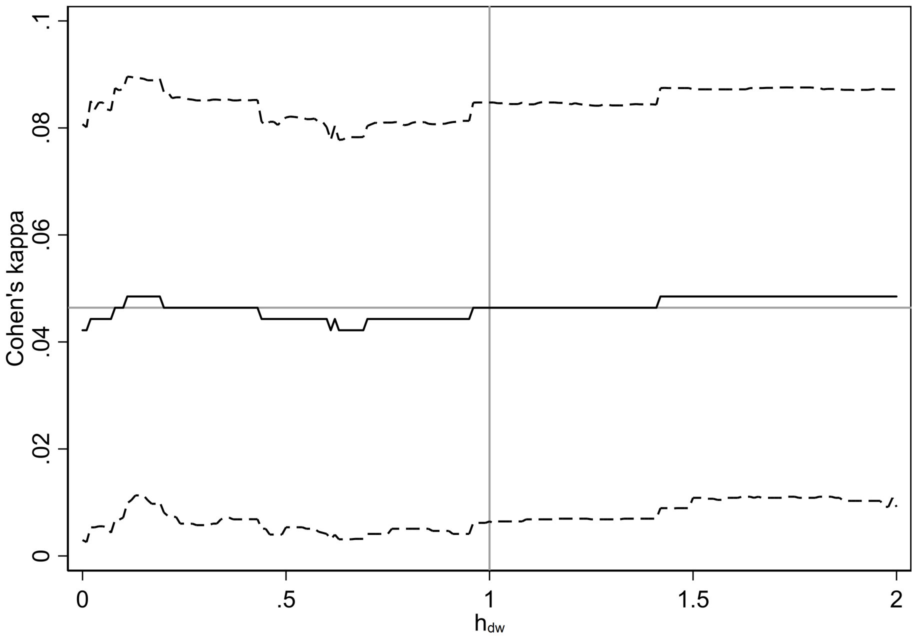

Controls matched to cases by age, gender and municipality of recidence. Analysis adjusted for age, gender, education and income.
Solid line is estimate, dashed lines are 95% confidence interval.

Δ(Cohen's kappa) as a function of h_{dw}

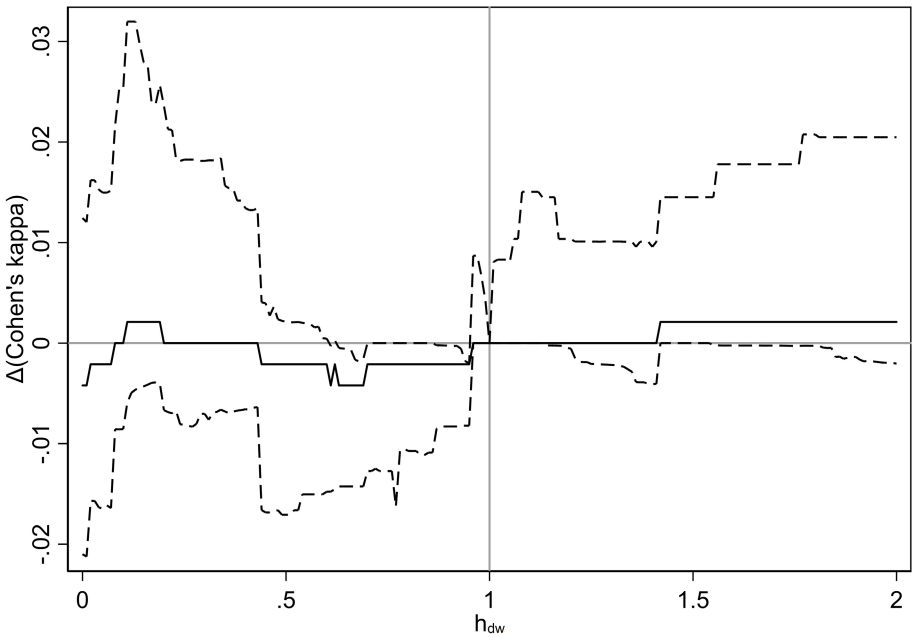

Controls matched to cases by age, gender and municipality of recidence. Analysis adjusted for age, gender, education and income.
Solid line is estimate, dashed lines are 95% confidence interval.

Secondary results for this analysis: Description of hyperparameter optimization

Cohen's kappa as a function of IDW power

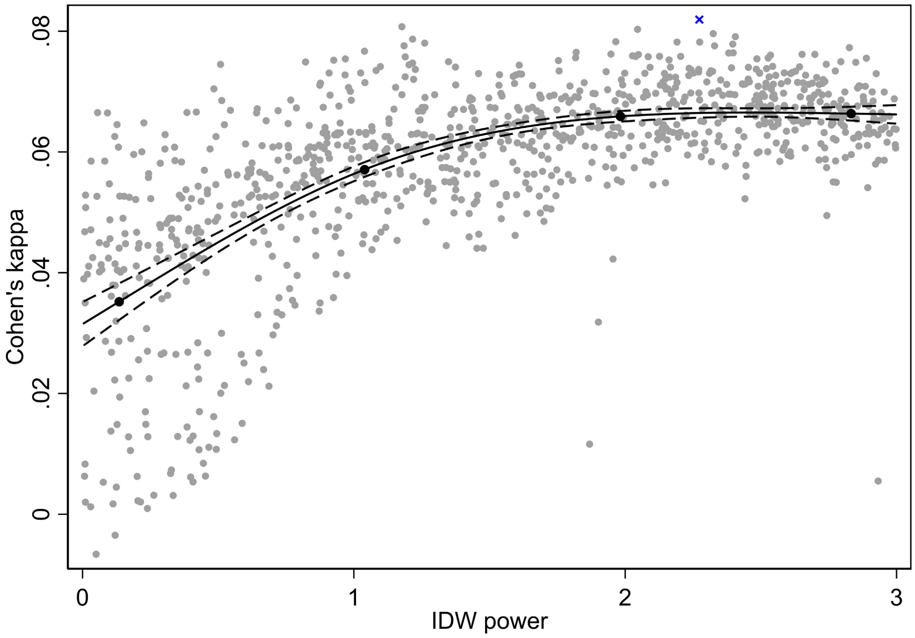

Each gray dot represents one crude analysis. The analyses used random time intervals and search radii. The blue × represents the analysis with the optimum combination of hyperparameters. The solid line represent the trend, modelled with restricted cubic splines with four knots (the location of the knots indicated by black dots). The dashed lines are the 95% confidence interval for the trend.

Cohen's kappa as a function of search radius

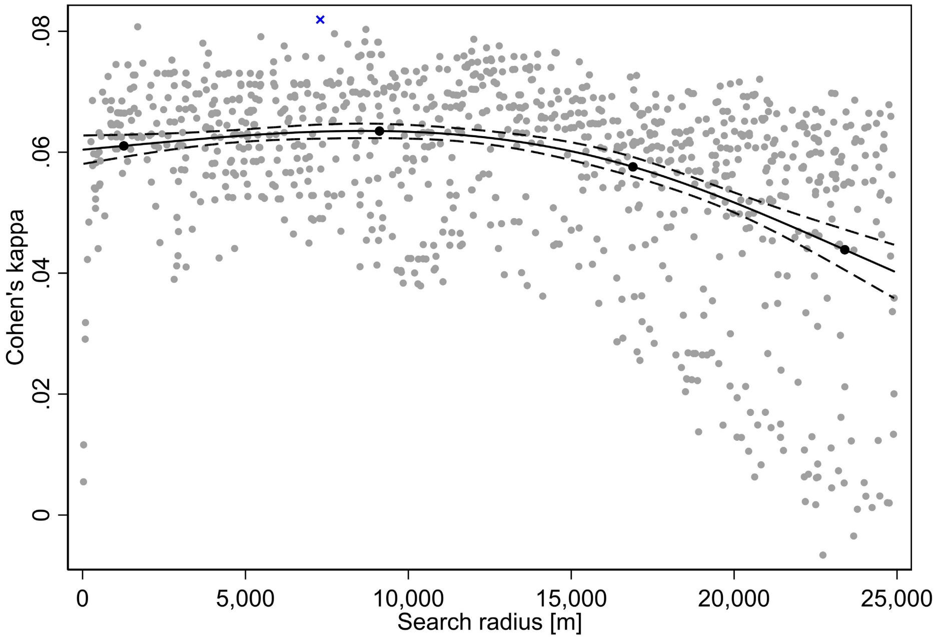

Each gray dot represents one crude analysis. The analyses used random time intervals and values of IDW power. The blue × represents the analysis with the optimum combination of hyperparameters. The solid line represent the trend, modelled with restricted cubic splines with four knots (the location of the knots indicated by black dots). The dashed lines are the 95% confidence interval for the trend.

Cohen's kappa as a function of time interval

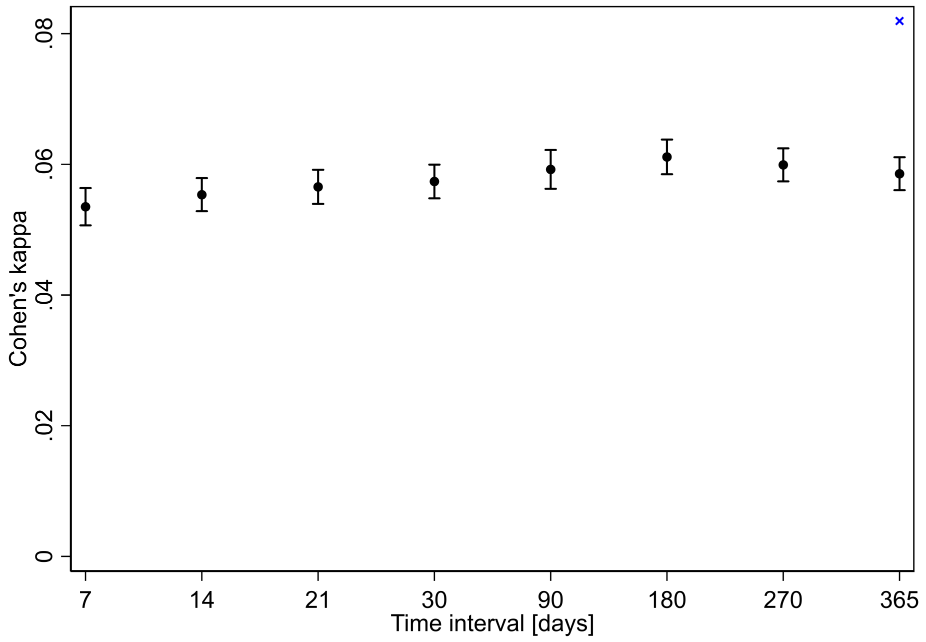

Each black dot represents the mean of the results for crude analyses with the indicated time interval. The analyses used random values of IDW power and search radius. The bars indicate the 95% confidence interval. The blue × represents the analysis with the optimum combination of hyperparameters.

Cohen's kappa as a function of search radius, IDW power and time interval

Time interval: 7 days

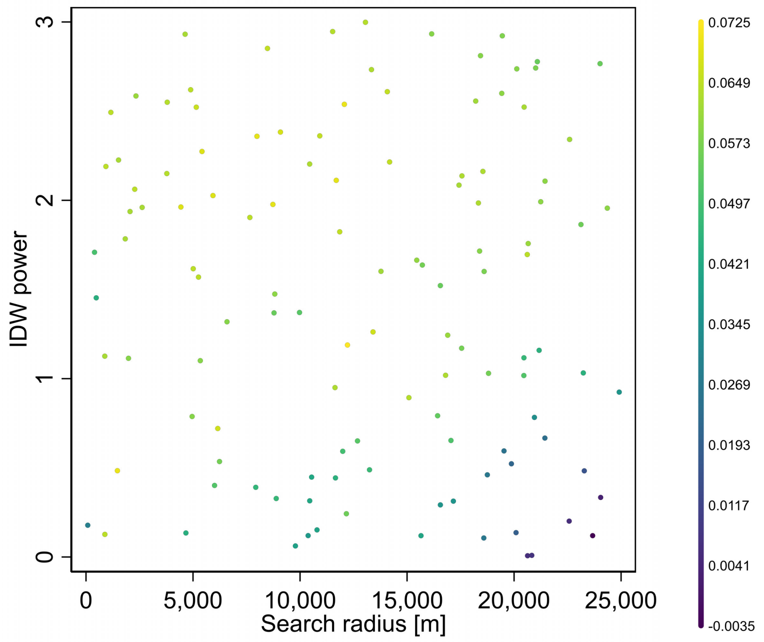

Each dot represents one crude analysis with the specified hyperparameters. Dots are color-coded according to the value of Cohen's kappa.

Time interval: 14 days

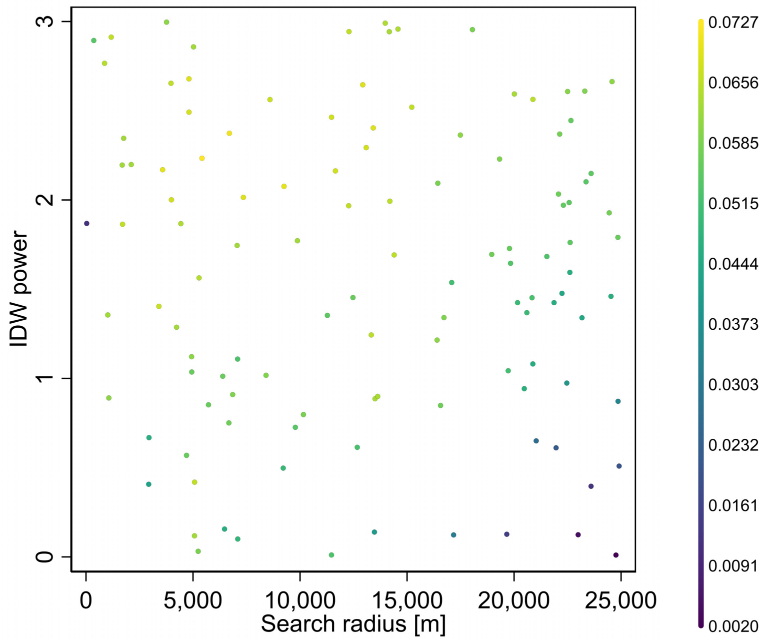

Each dot represents one crude analysis with the specified hyperparameters. Dots are color-coded according to the value of Cohen's kappa.

Time interval: 21 days

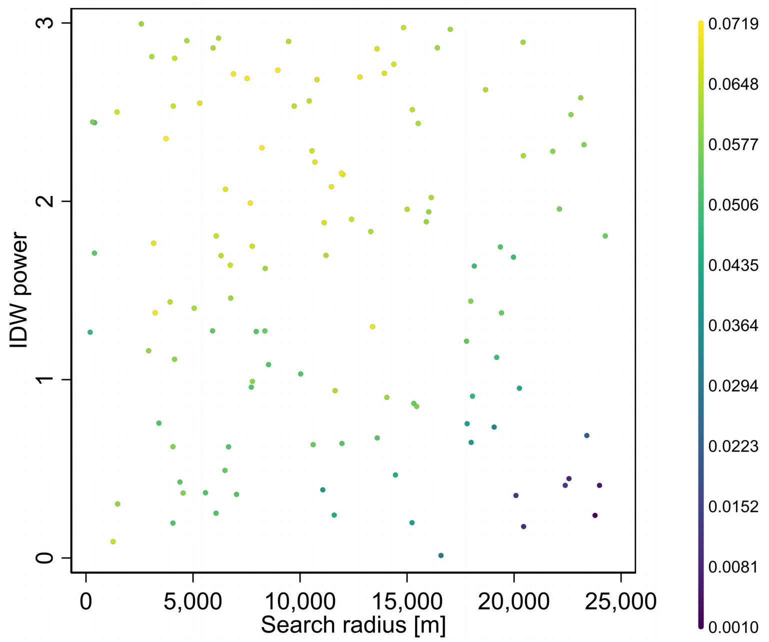

Each dot represents one crude analysis with the specified hyperparameters. Dots are color-coded according to the value of Cohen's kappa.

Time interval: 30 days

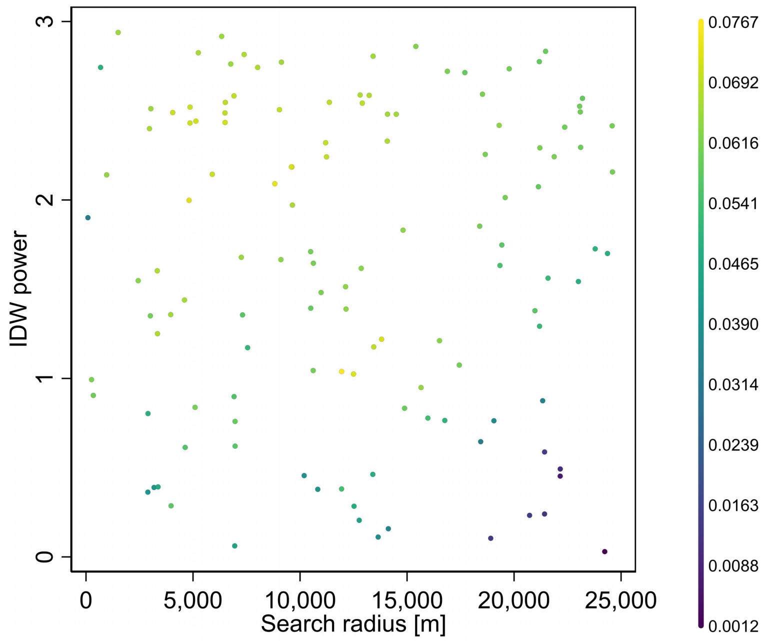

Each dot represents one crude analysis with the specified hyperparameters. Dots are color-coded according to the value of Cohen's kappa.

Time interval: 90 days

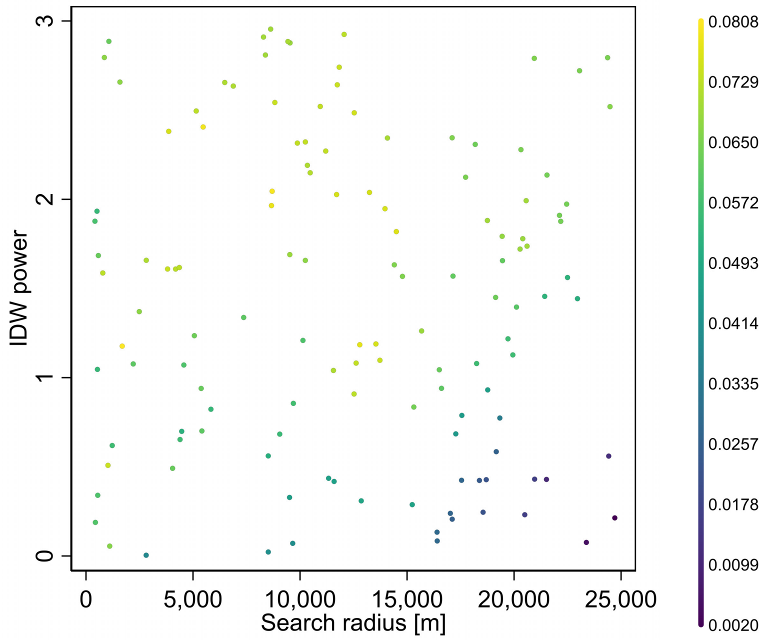

Each dot represents one crude analysis with the specified hyperparameters. Dots are color-coded according to the value of Cohen's kappa.

Time interval: 180 days

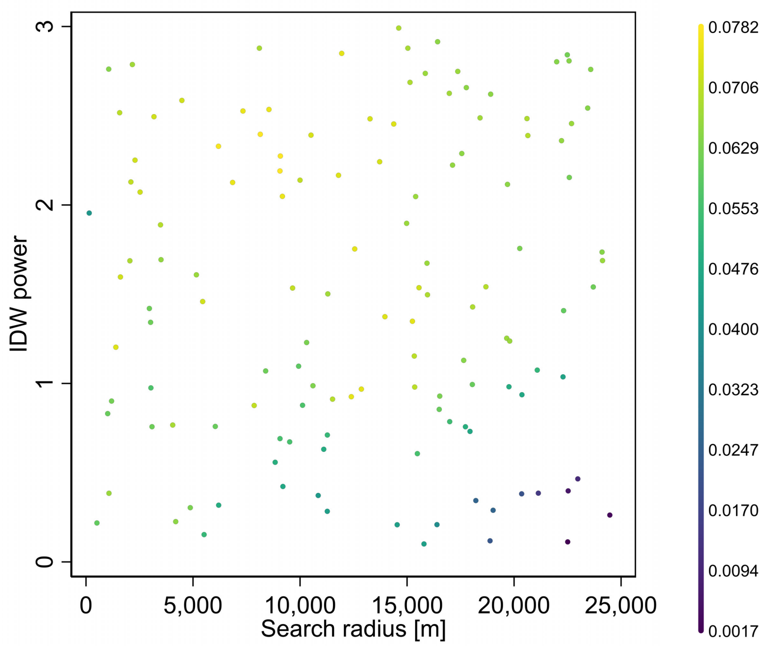

Each dot represents one crude analysis with the specified hyperparameters. Dots are color-coded according to the value of Cohen's kappa.

Time interval: 270 days

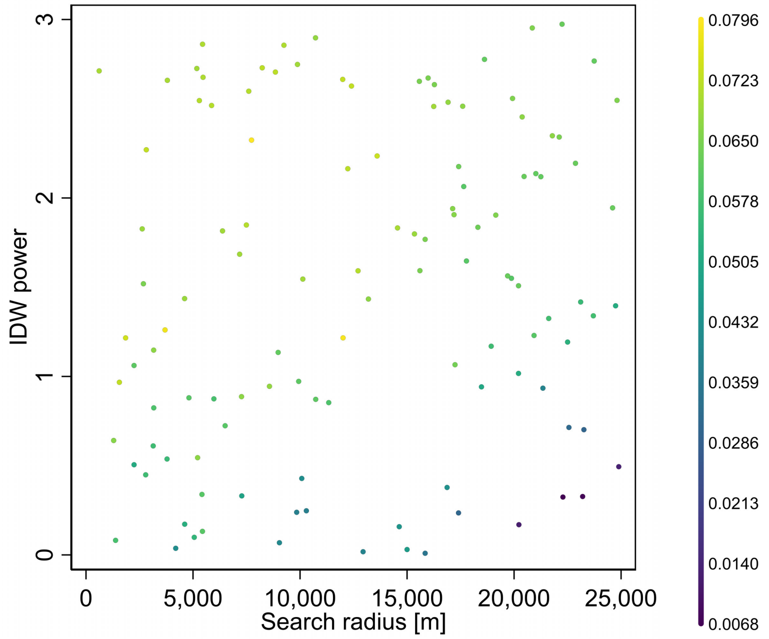

Each dot represents one crude analysis with the specified hyperparameters. Dots are color-coded according to the value of Cohen's kappa.

Time interval: 365 days

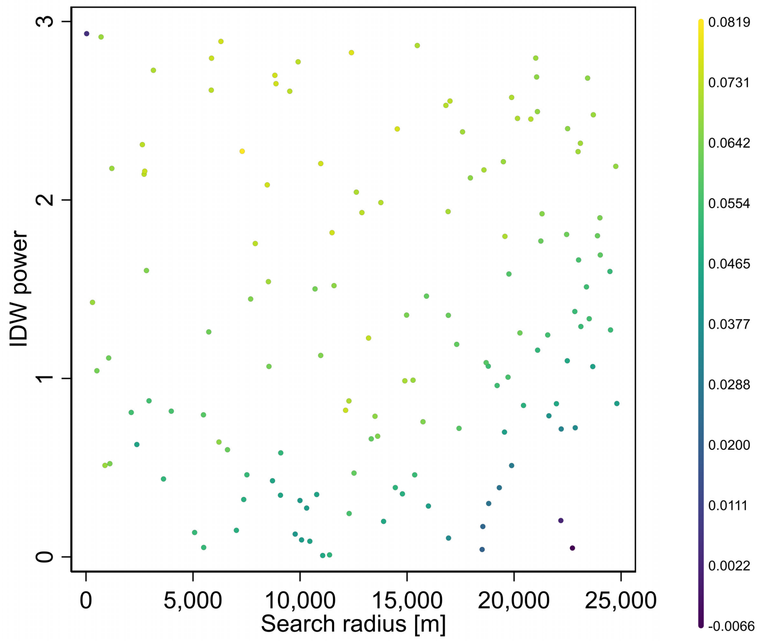

Each dot represents one crude analysis with the specified hyperparameters. Dots are color-coded according to the value of Cohen's kappa.

Time interval: Any

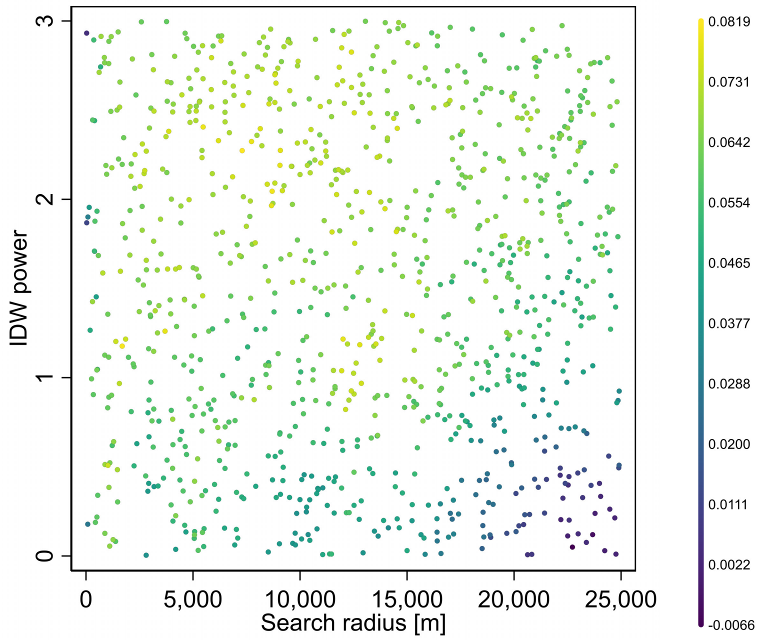

Each dot represents one crude analysis with the specified hyperparameters. Dots are color-coded according to the value of Cohen's kappa.

Analysis number 8

Primary results from this analysis

Crude analysis

Brier score as a function of h_{dw}

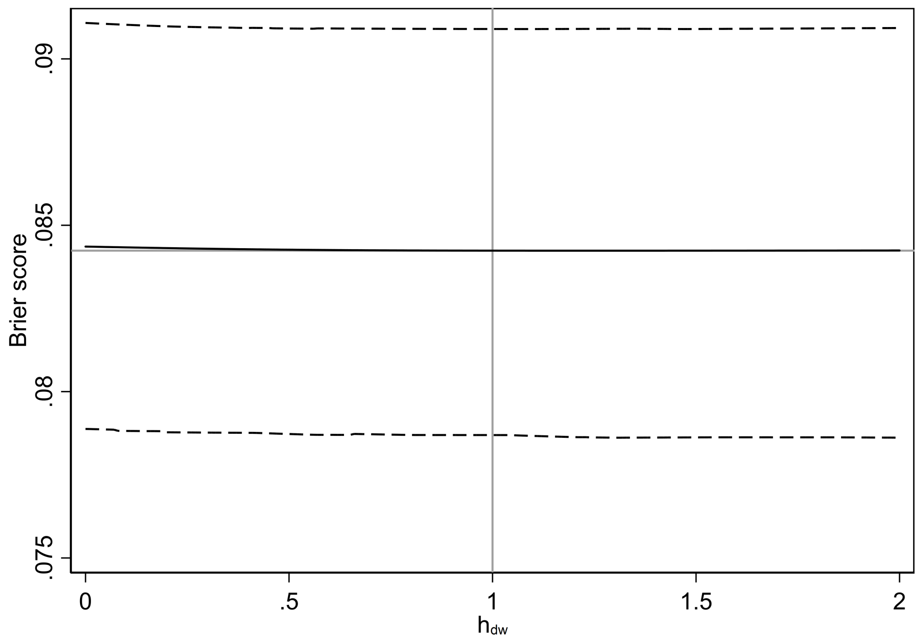

Controls matched to cases by age, gender and municipality of recidence.
Solid line is estimate, dashed lines are 95% confidence interval.

Δ(Brier score) as a function of h_{dw}

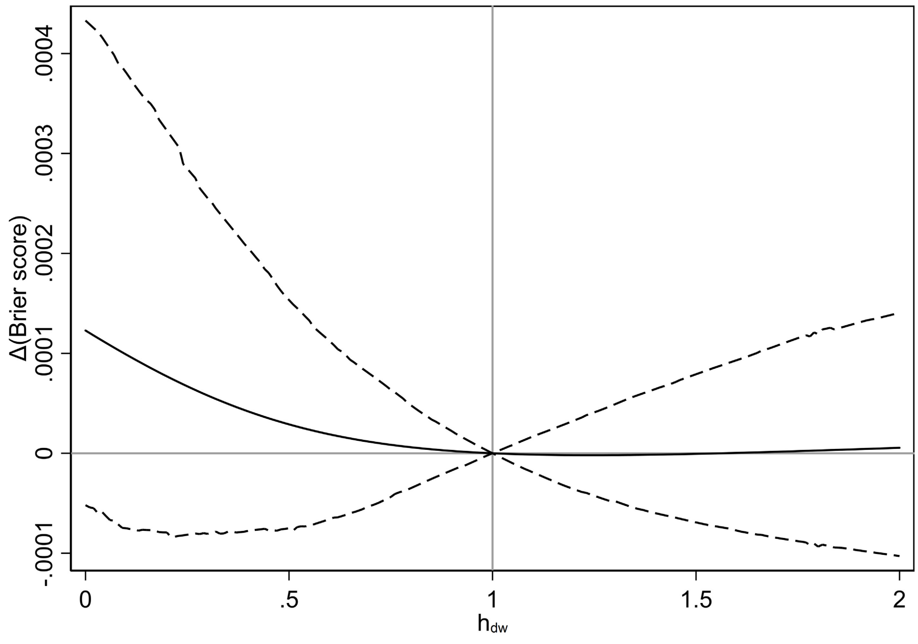

Controls matched to cases by age, gender and municipality of recidence.
Solid line is estimate, dashed lines are 95% confidence interval.

Adjusted analysis

Brier score as a function of h_{dw}

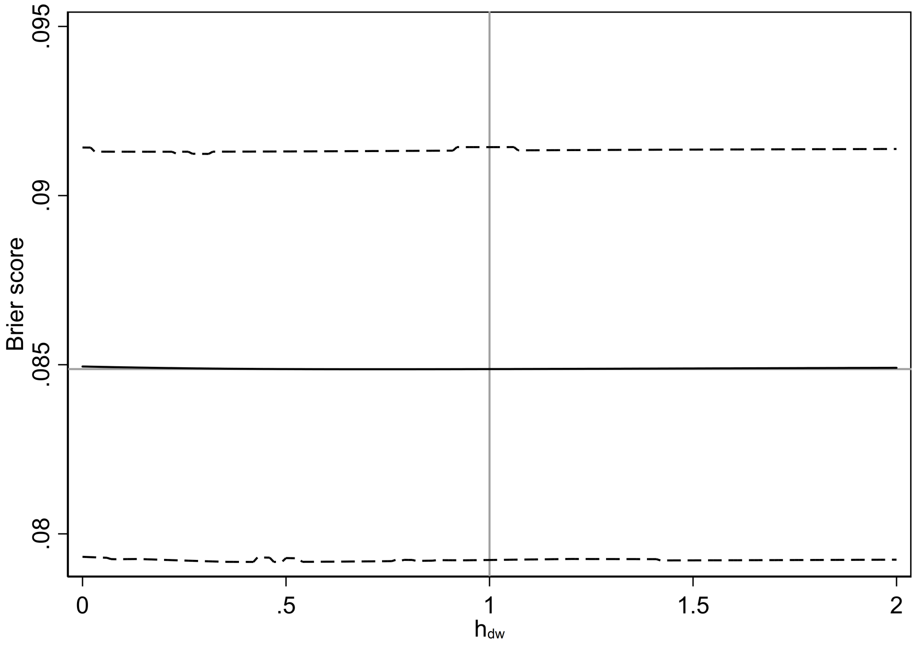

Controls matched to cases by age, gender and municipality of recidence. Analysis adjusted for age, gender, education and income.
Solid line is estimate, dashed lines are 95% confidence interval.

Δ(Brier score) as a function of h_{dw}

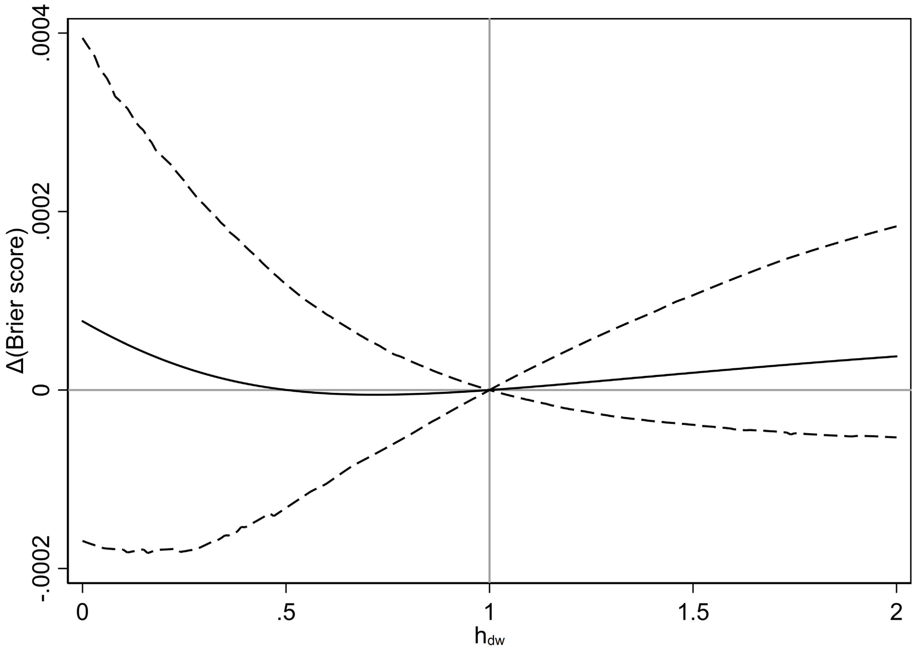

Controls matched to cases by age, gender and municipality of recidence. Analysis adjusted for age, gender, education and income.
Solid line is estimate, dashed lines are 95% confidence interval.

Secondary results for this analysis: Description of hyperparameter optimization

Brier score as a function of IDW power

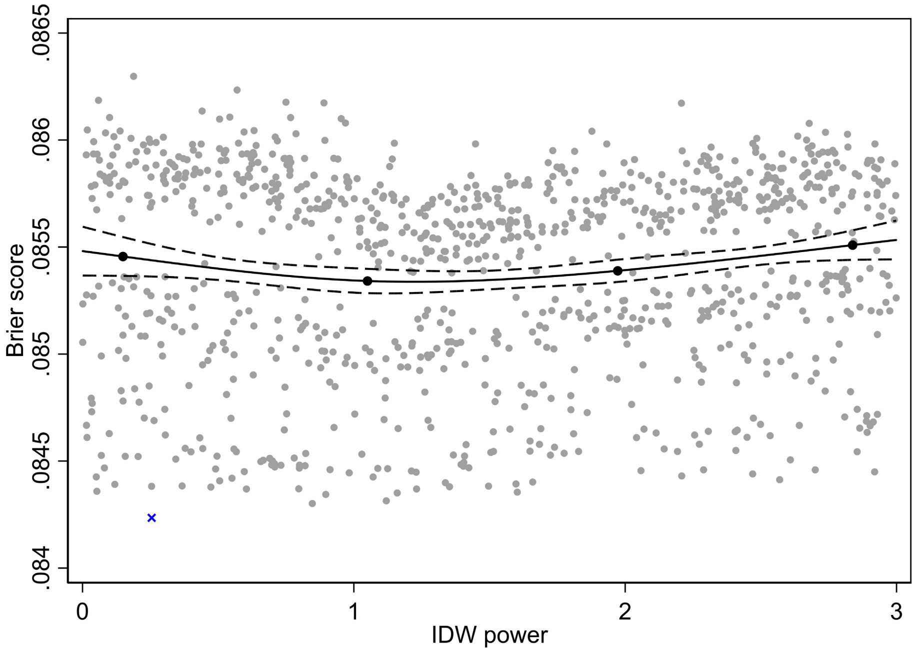

Each gray dot represents one crude analysis. The analyses used random time intervals and search radii. The blue × represents the analysis with the optimum combination of hyperparameters. The solid line represent the trend, modelled with restricted cubic splines with four knots (the location of the knots indicated by black dots). The dashed lines are the 95% confidence interval for the trend.

Brier score as a function of search radius

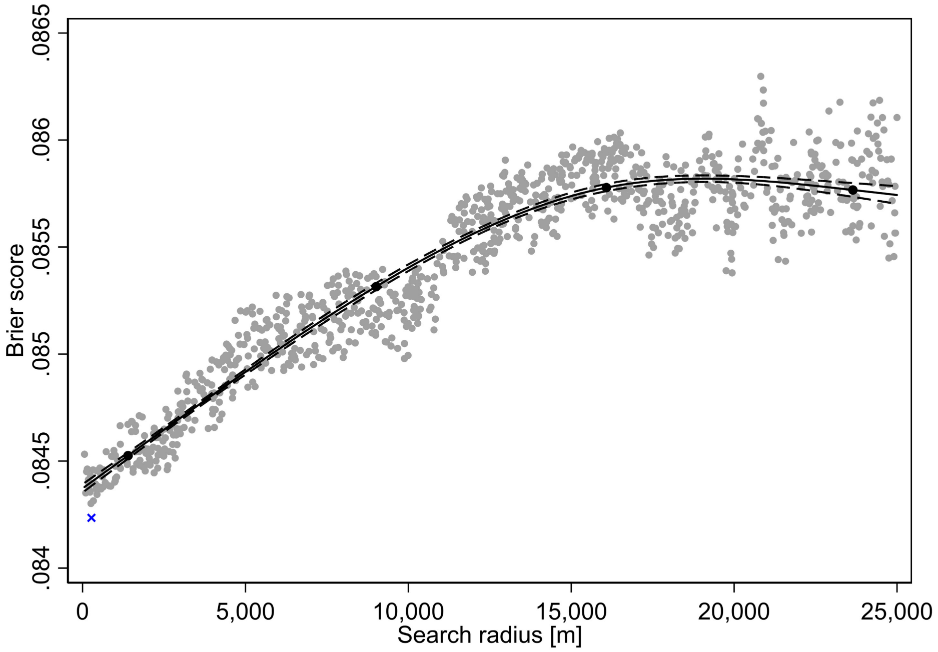

Each gray dot represents one crude analysis. The analyses used random time intervals and values of IDW power. The blue × represents the analysis with the optimum combination of hyperparameters. The solid line represent the trend, modelled with restricted cubic splines with four knots (the location of the knots indicated by black dots). The dashed lines are the 95% confidence interval for the trend.

Brier score as a function of time interval

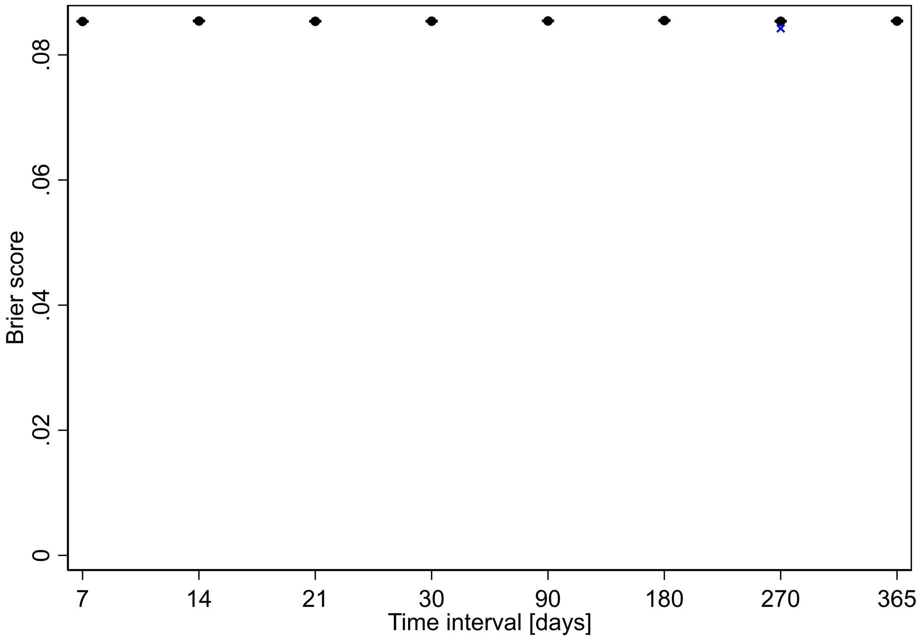

Each black dot represents the mean of the results for crude analyses with the indicated time interval. The analyses used random values of IDW power and search radius. The bars indicate the 95% confidence interval. The blue × represents the analysis with the optimum combination of hyperparameters.

Brier score as a function of search radius, IDW power and time interval

Time interval: 7 days

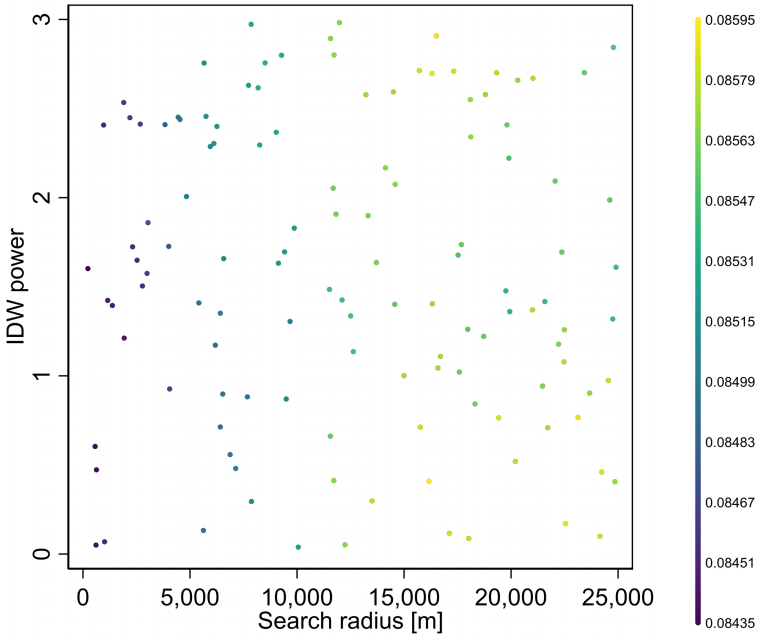

Each dot represents one crude analysis with the specified hyperparameters. Dots are color-coded according to the value of Brier score.

Time interval: 14 days

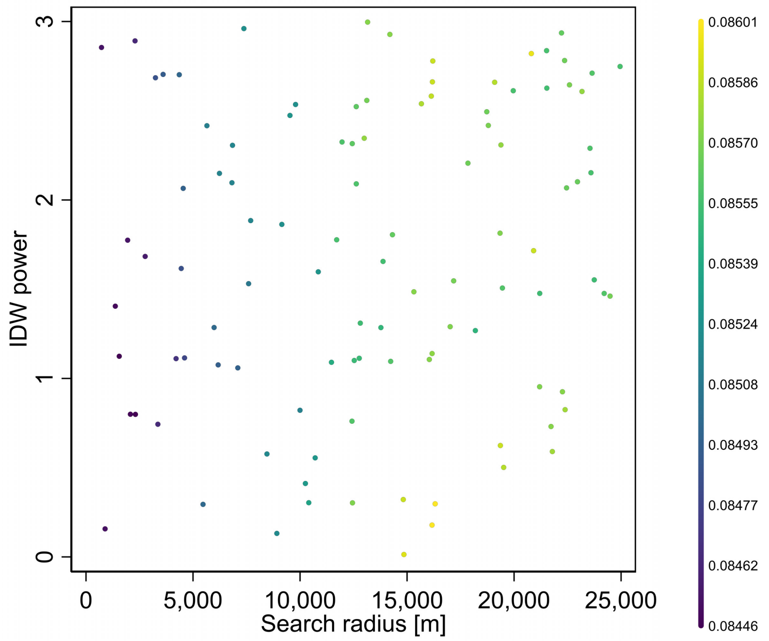

Each dot represents one crude analysis with the specified hyperparameters. Dots are color-coded according to the value of Brier score.

Time interval: 21 days

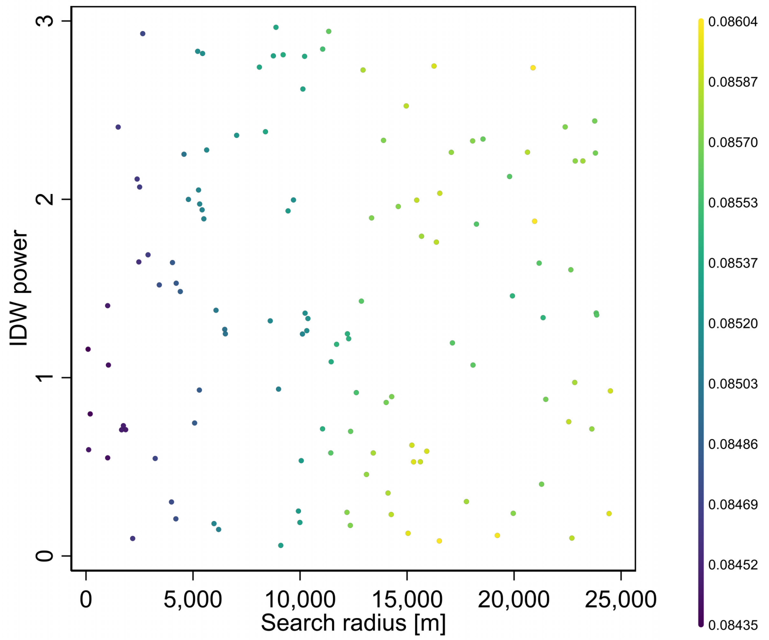

Each dot represents one crude analysis with the specified hyperparameters. Dots are color-coded according to the value of Brier score.

Time interval: 30 days

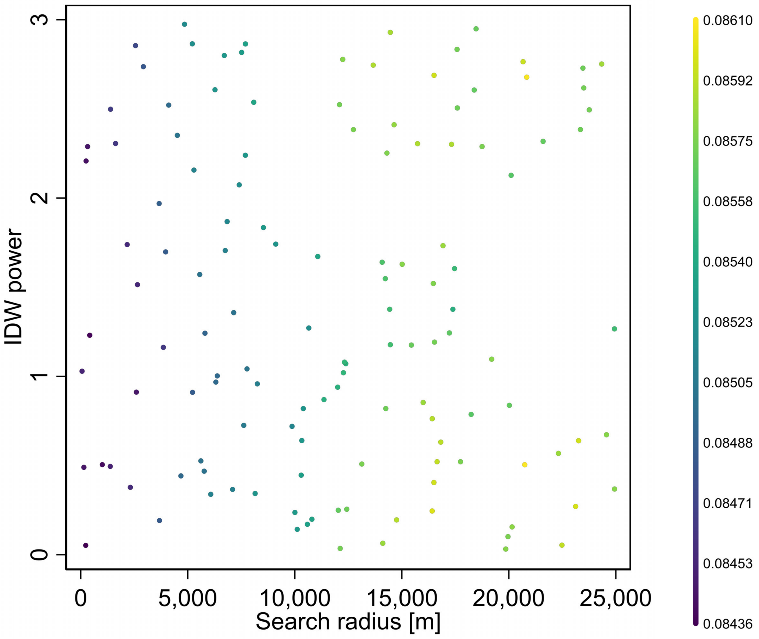

Each dot represents one crude analysis with the specified hyperparameters. Dots are color-coded according to the value of Brier score.

Time interval: 90 days

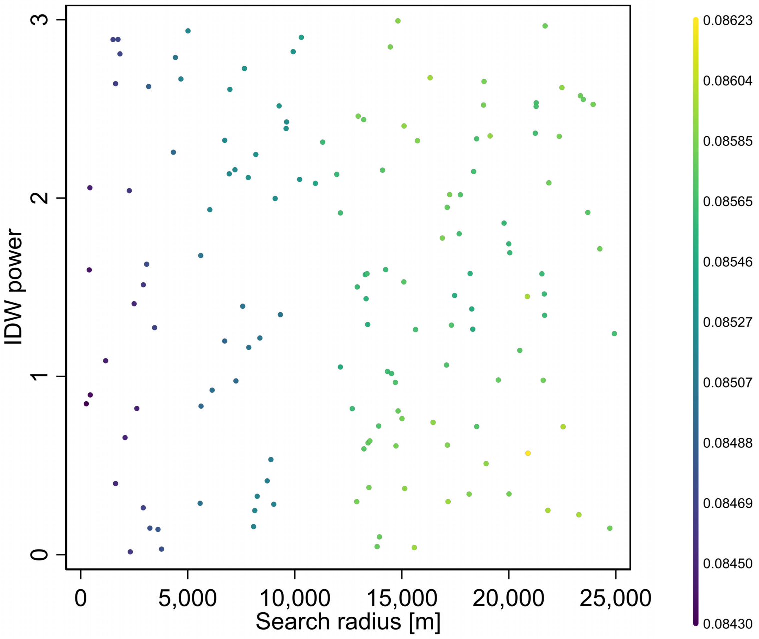

Each dot represents one crude analysis with the specified hyperparameters. Dots are color-coded according to the value of Brier score.

Time interval: 180 days

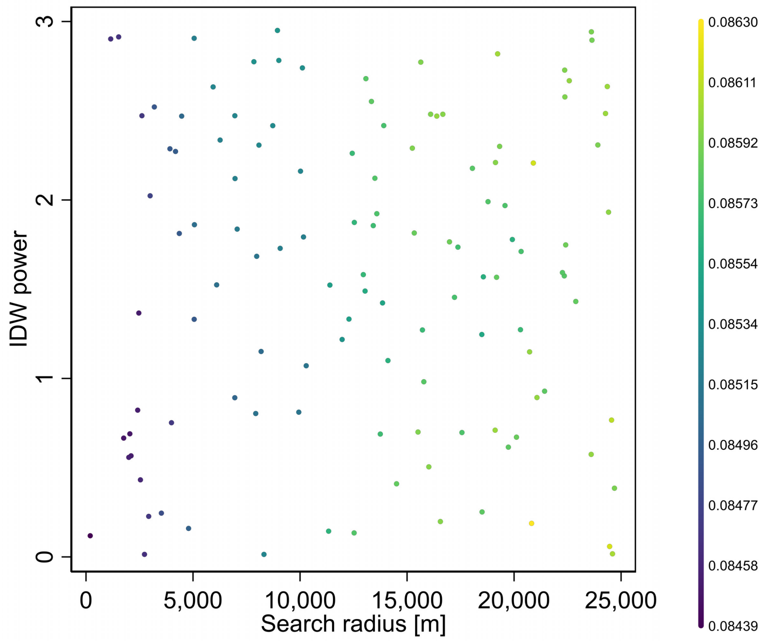

Each dot represents one crude analysis with the specified hyperparameters. Dots are color-coded according to the value of Brier score.

Time interval: 270 days

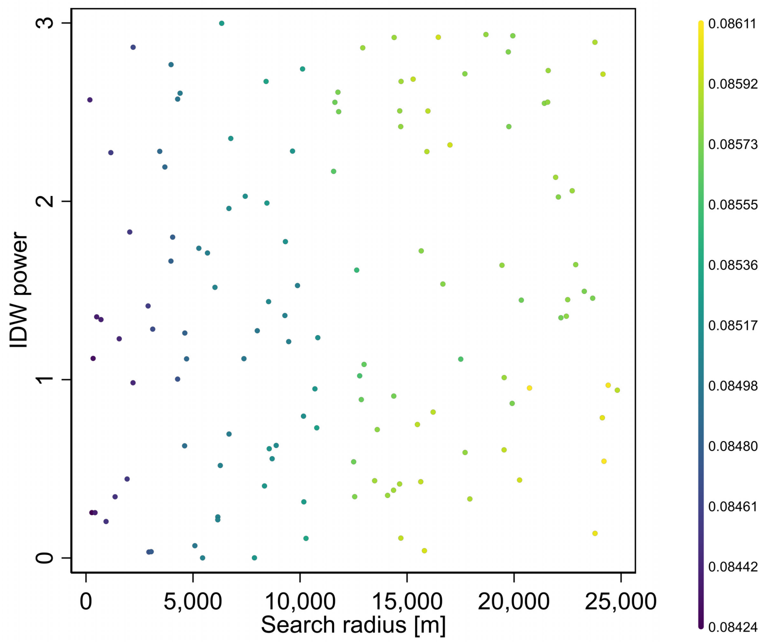

Each dot represents one crude analysis with the specified hyperparameters. Dots are color-coded according to the value of Brier score.

Time interval: 365 days

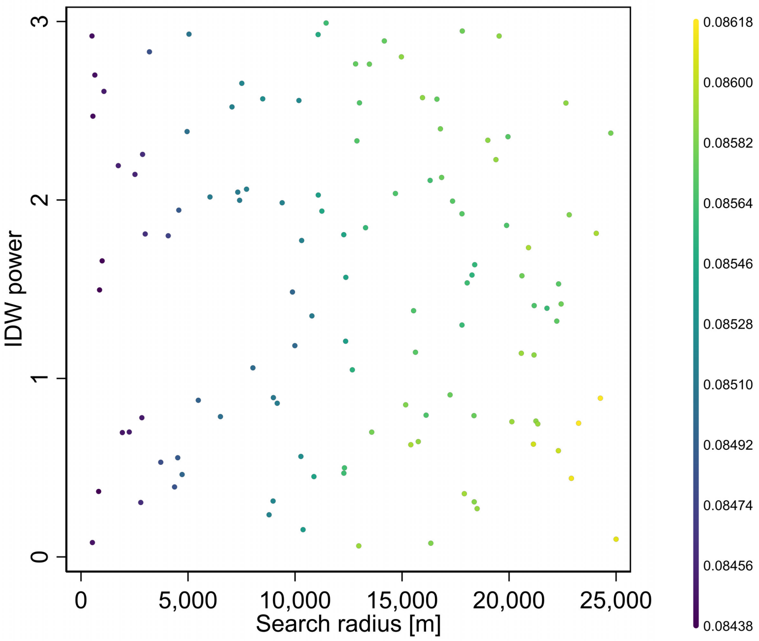

Each dot represents one crude analysis with the specified hyperparameters. Dots are color-coded according to the value of Brier score.

Time interval: Any

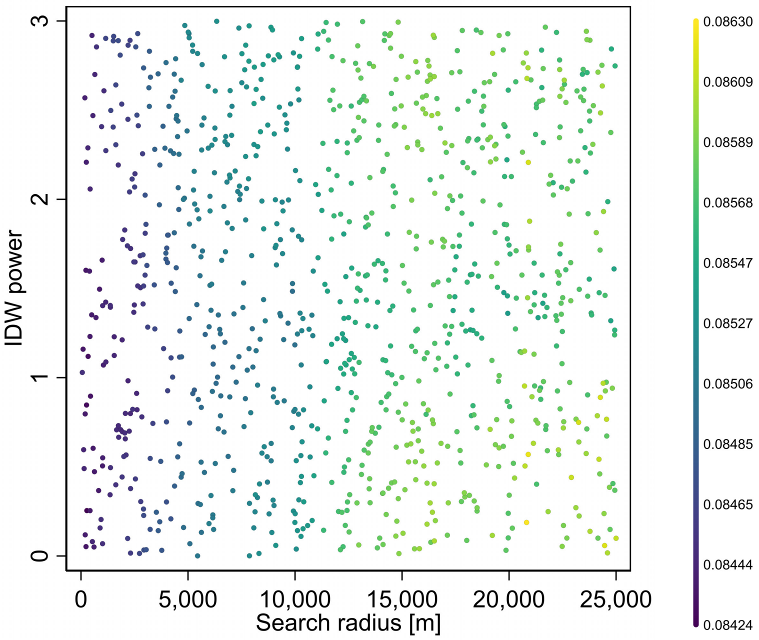

Each dot represents one crude analysis with the specified hyperparameters. Dots are color-coded according to the value of Brier score.
