# Supplementary material for: Influence of wind direction on the relationship between proximity to pig farms and risk of infection with MRSA CC398 among persons without known contact to livestock: a Danish nationwide population-based study
Source: Infection. 2025 Sep 8;53(6):2795–808. doi: 10.1007/s15010-025-02629-2 (PMC12675557; doi:10.1007/s15010-025-02629-2)

Online supplement S4: Graphical presentation of secondary analysis

On the following pages, results are presented for a secondary post-hoc analysis in which the number of pig herds has been summarized for each participant within a ring-shaped zone, followed by a comparison of the mean number of herds in each zone for cases and controls.

Zones are color-coded according to the difference between cases and controls, relative to the mean number for controls. Red is positive (more pig herds for cases), blue is negative (more pig herds for controls). The color bar at the right side of each figure shows the numerical meaning of the colors (numbers are in percent). Zones marked with a dot indicate statistically significant differences between cases and controls. The house and pig symbols indicate the positions of the home address and the pig herds, respectively.

In crude analyses, controls were matched to cases based on age, gender and municipality. In adjusted analyses, controls were matched in the same manner, but the analyses were also adjusted for age, gender, education and income. When calculating the relative difference between cases and controls in the adjusted analysis, the difference between the two groups was divided by the mean value for a control with female gender, age 50 years, income percentile 50, and less than high school education.

Wind summarized over 7 days

Crude analysis

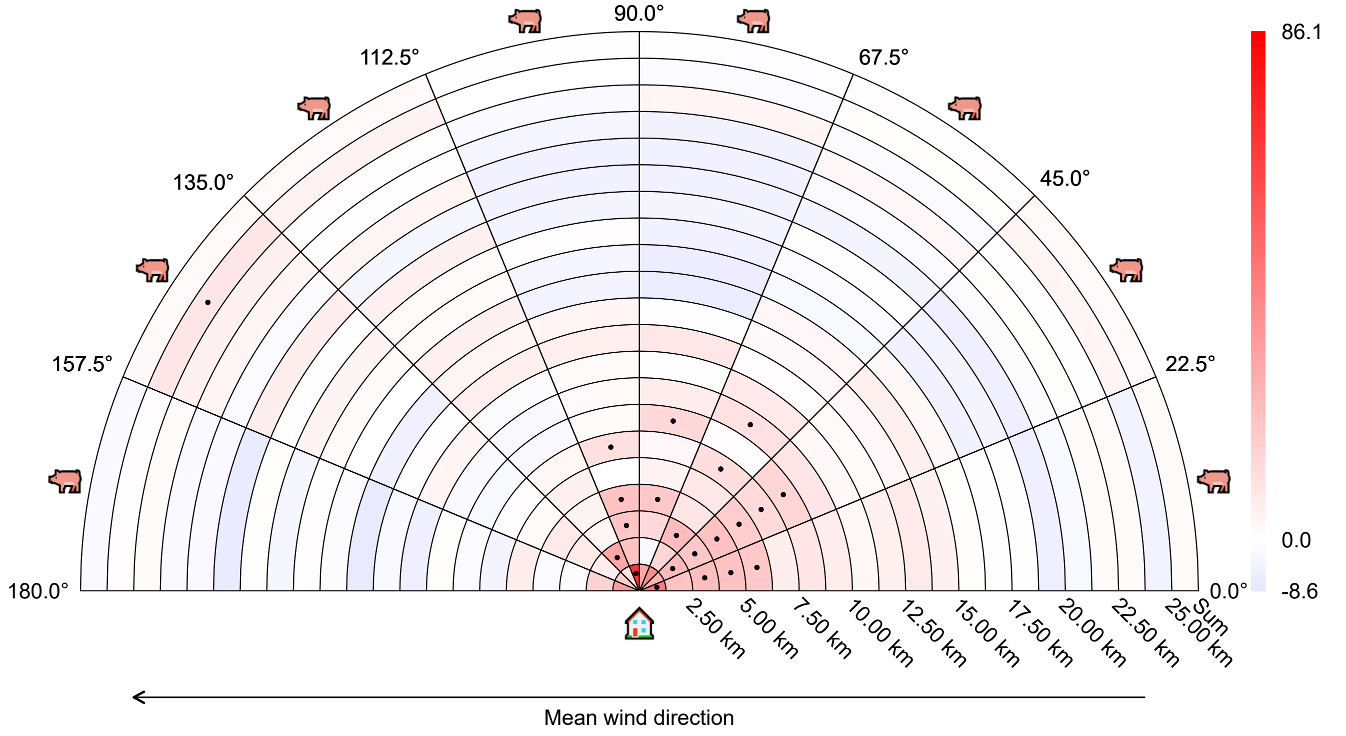

Adjusted analysis

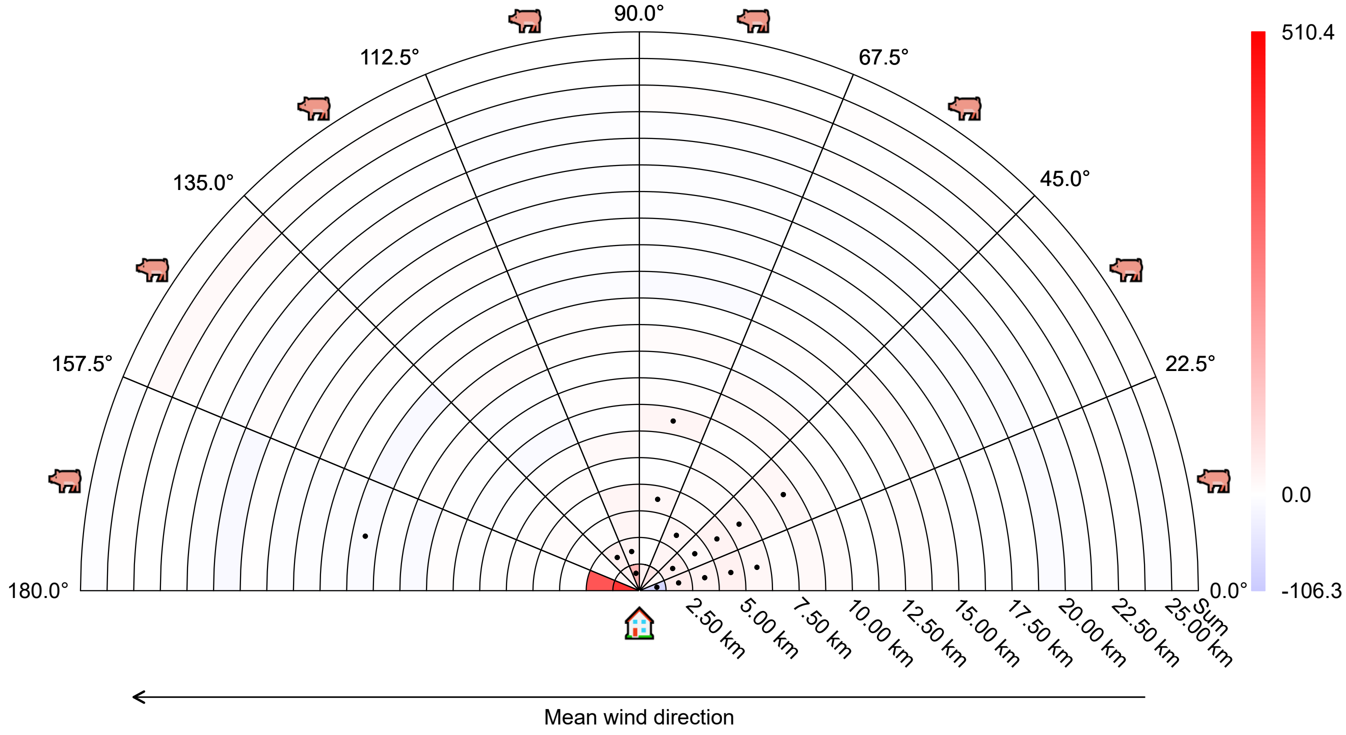

Wind summarized over 14 days

Crude analysis

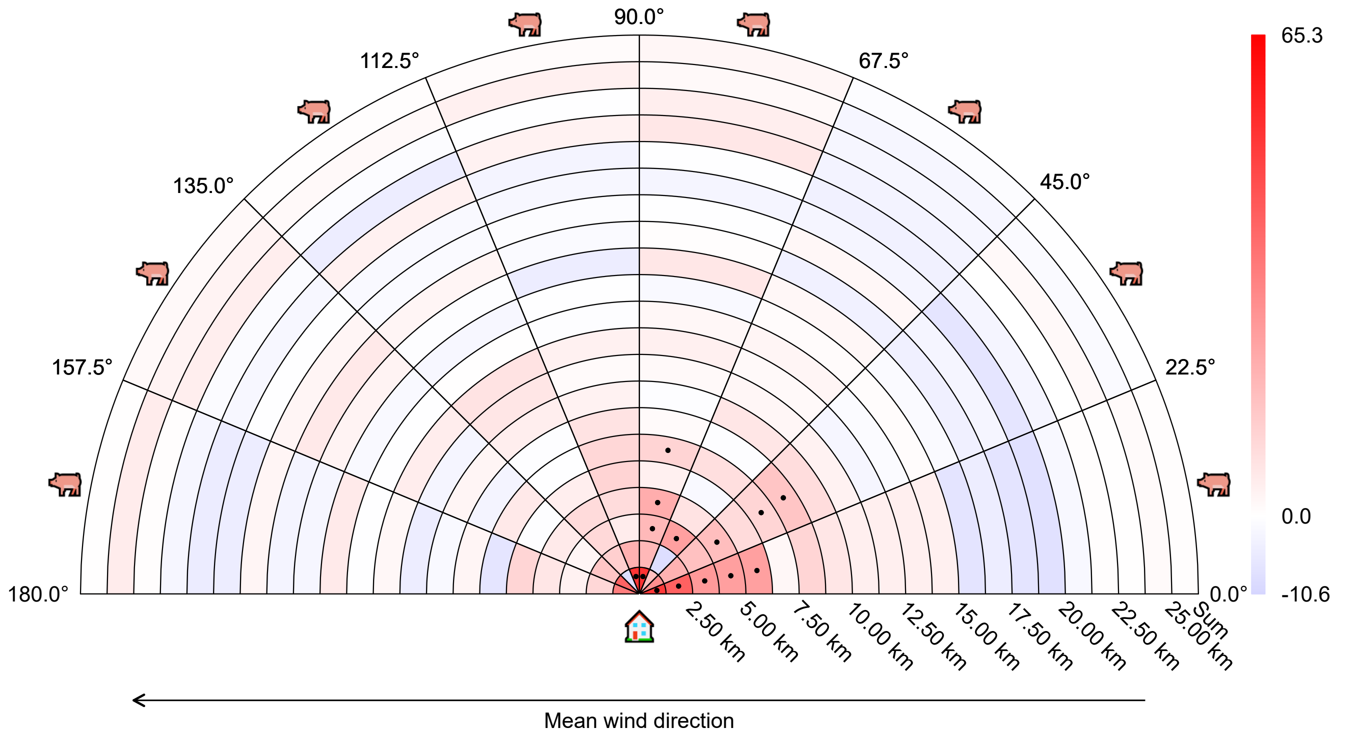

Adjusted analysis

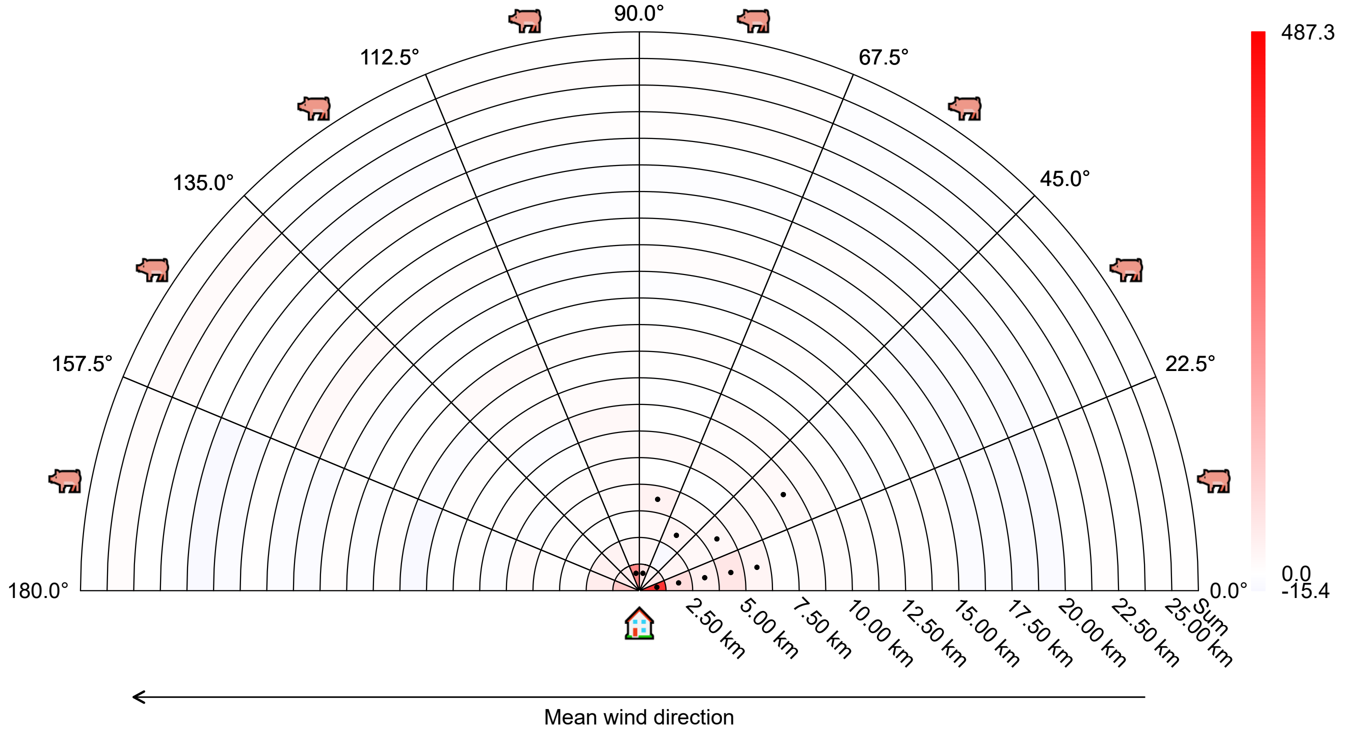

Wind summarized over 21 days

Crude analysis

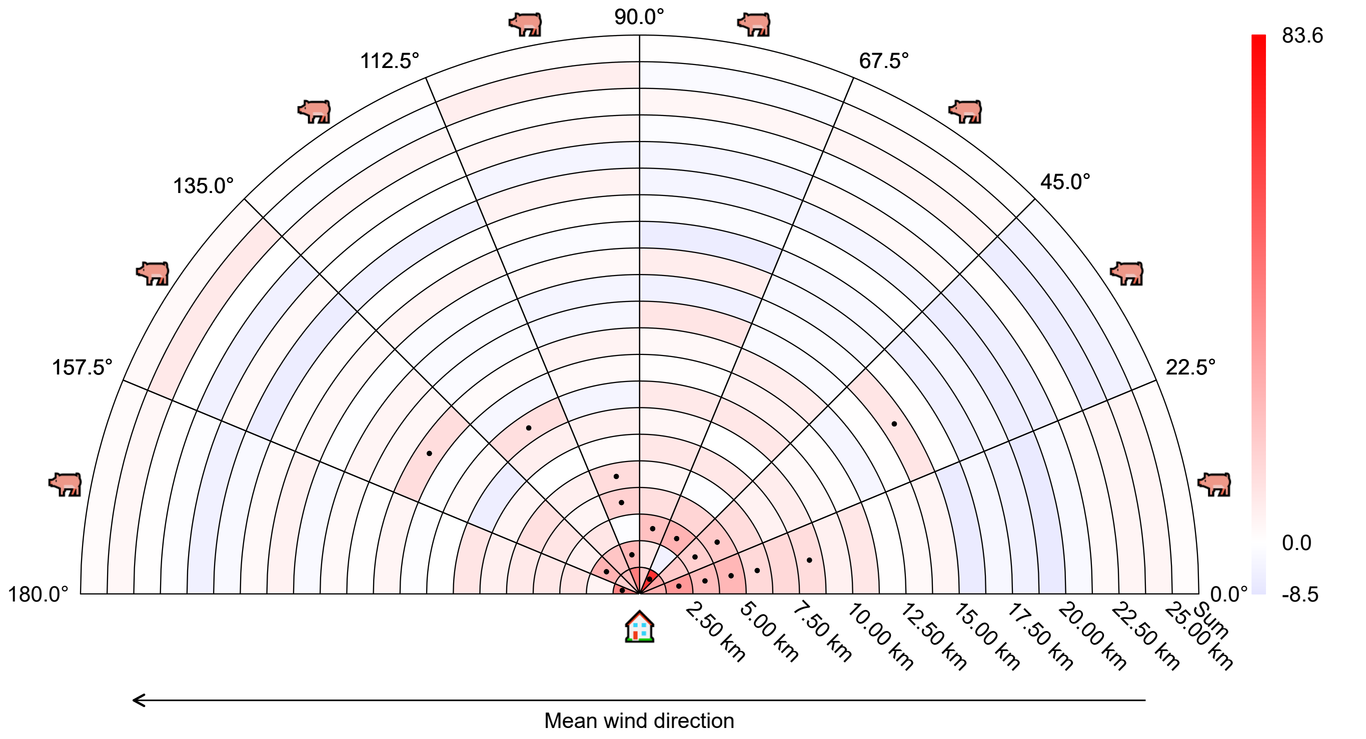

Adjusted analysis

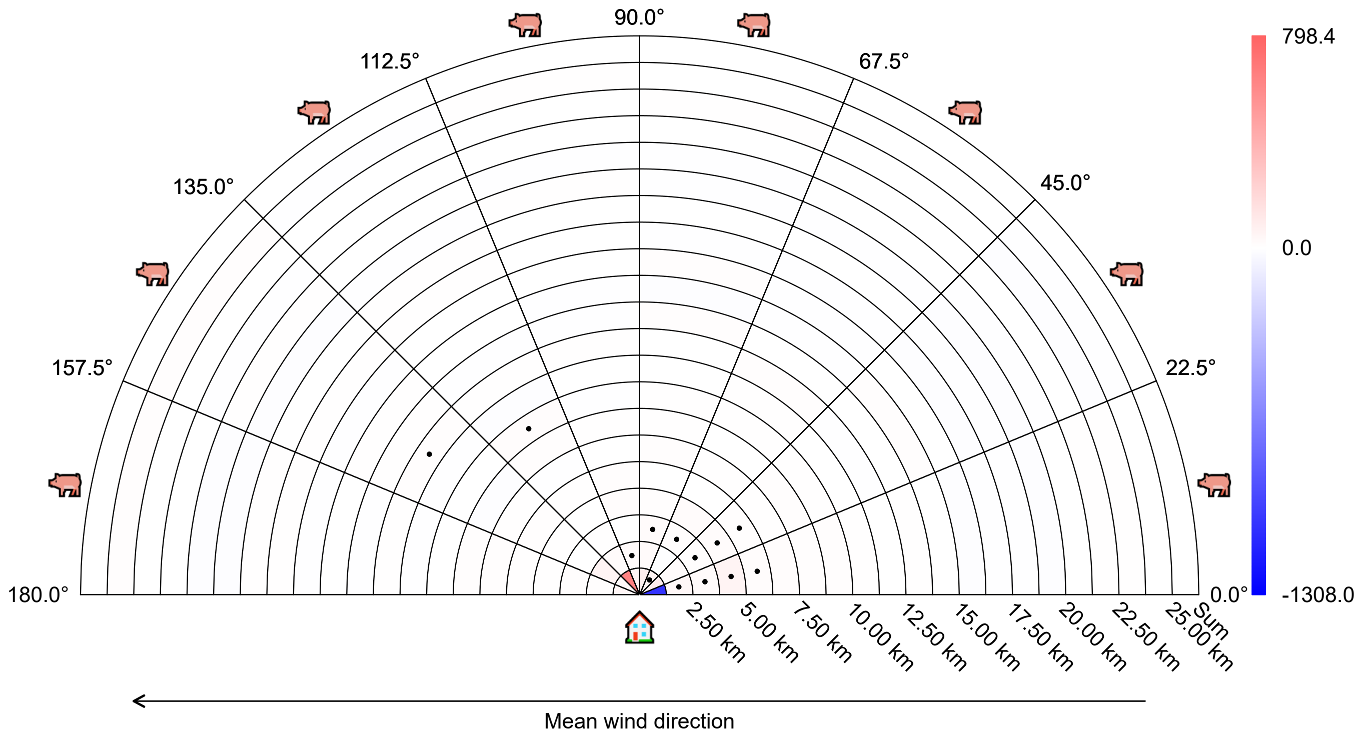

Wind summarized over 30 days

Crude analysis

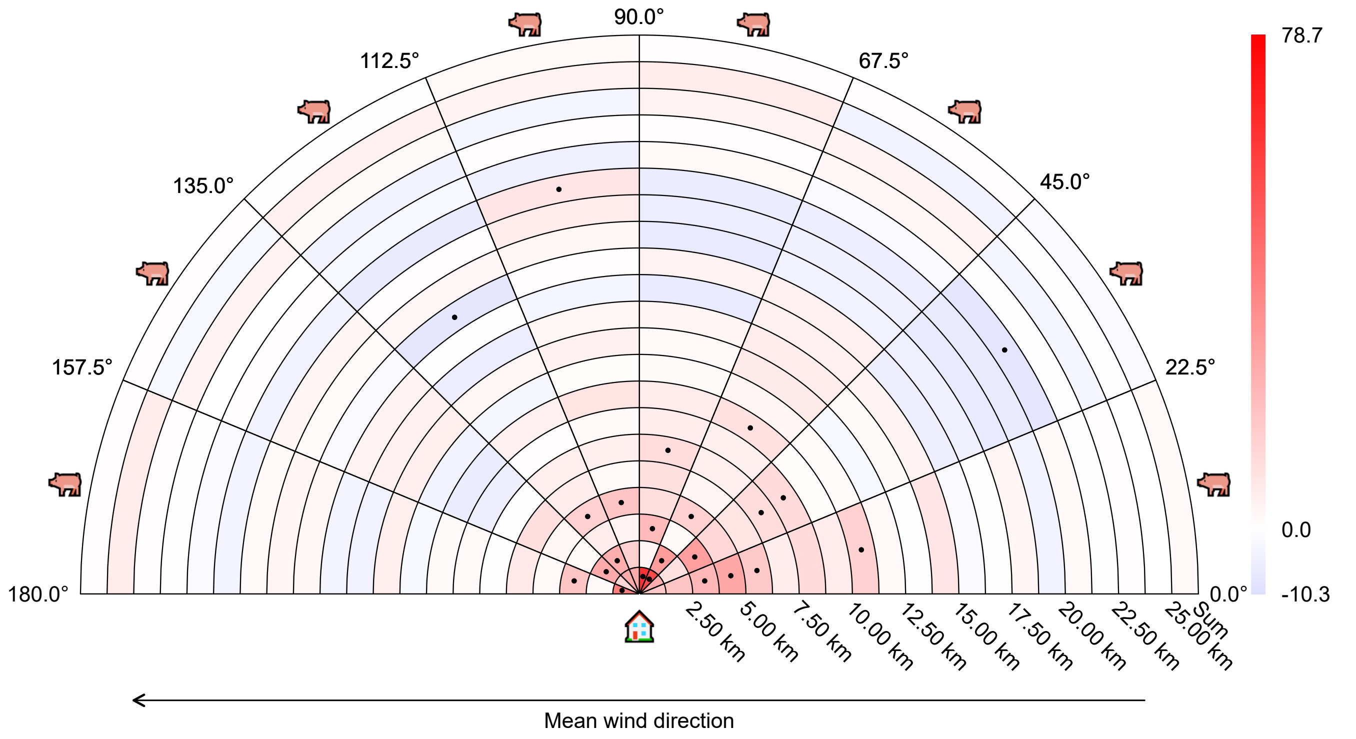

Adjusted analysis

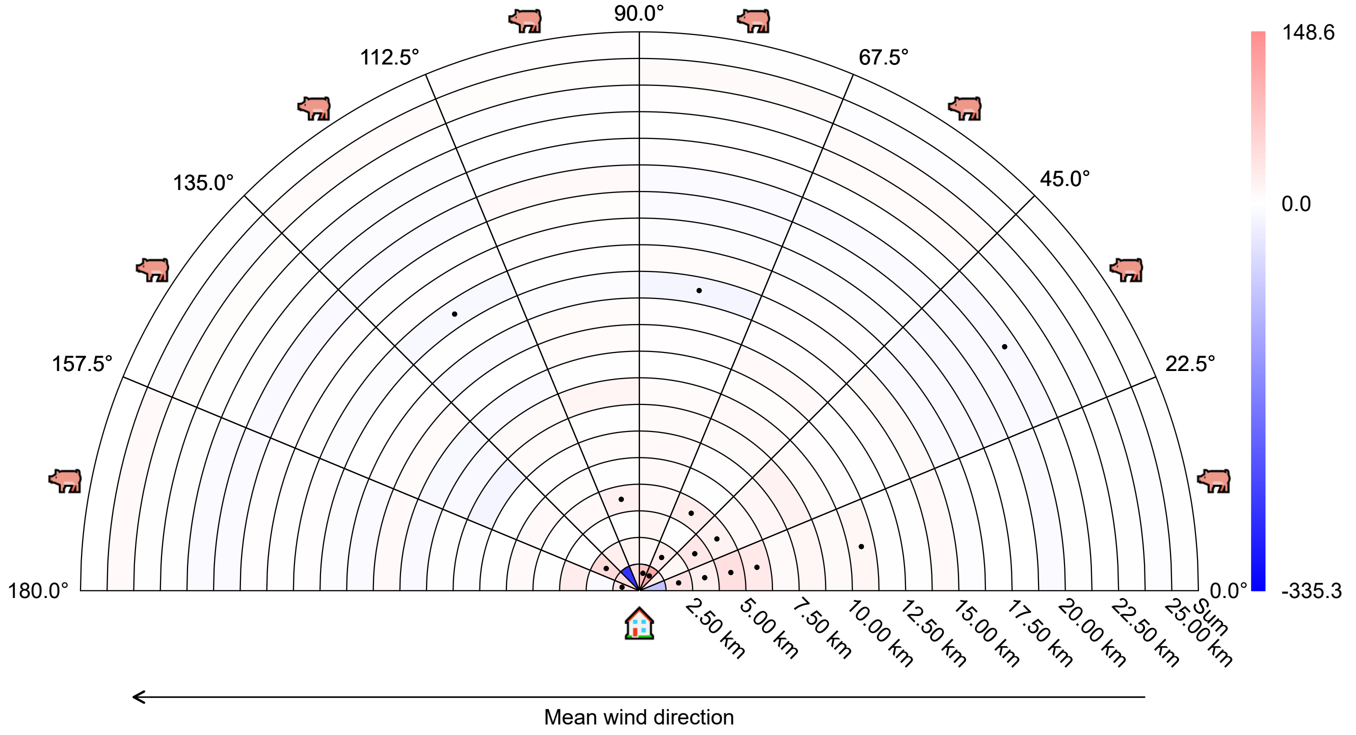

Wind summarized over 90 days

Crude analysis

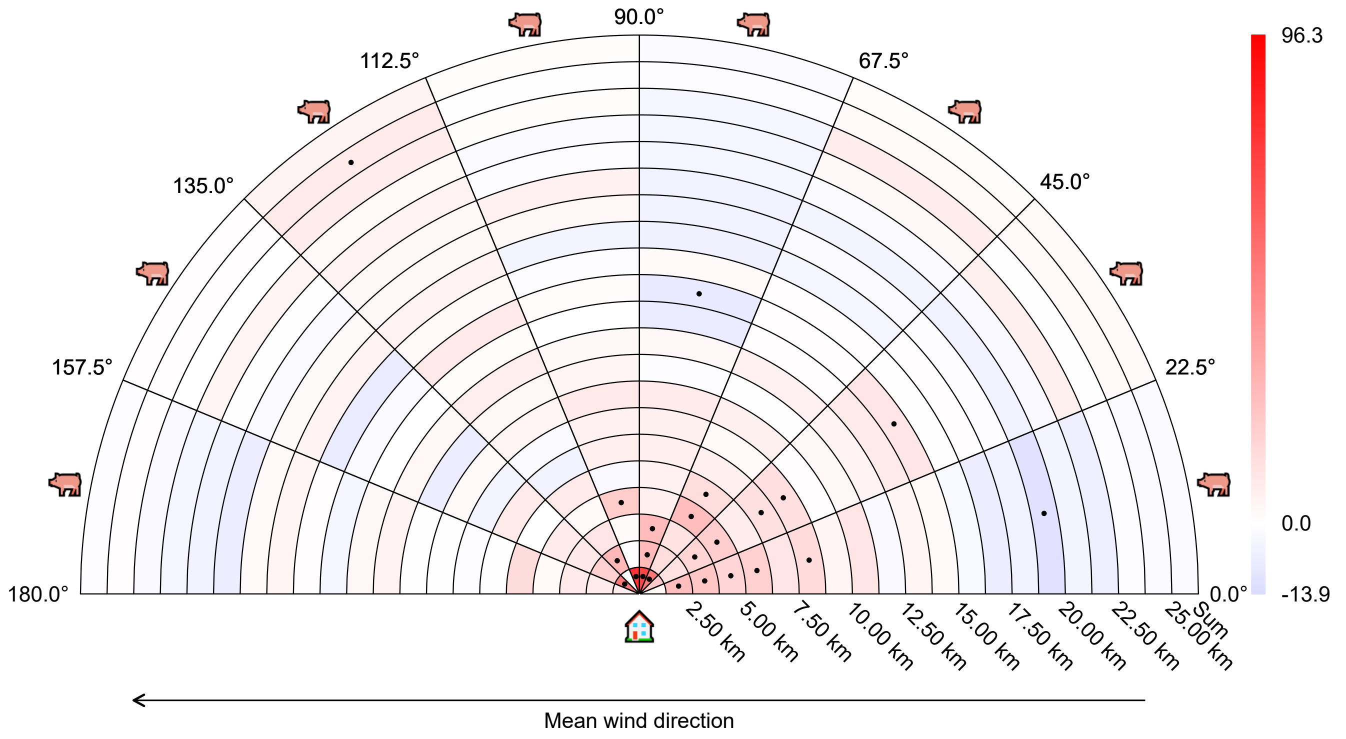

Adjusted analysis

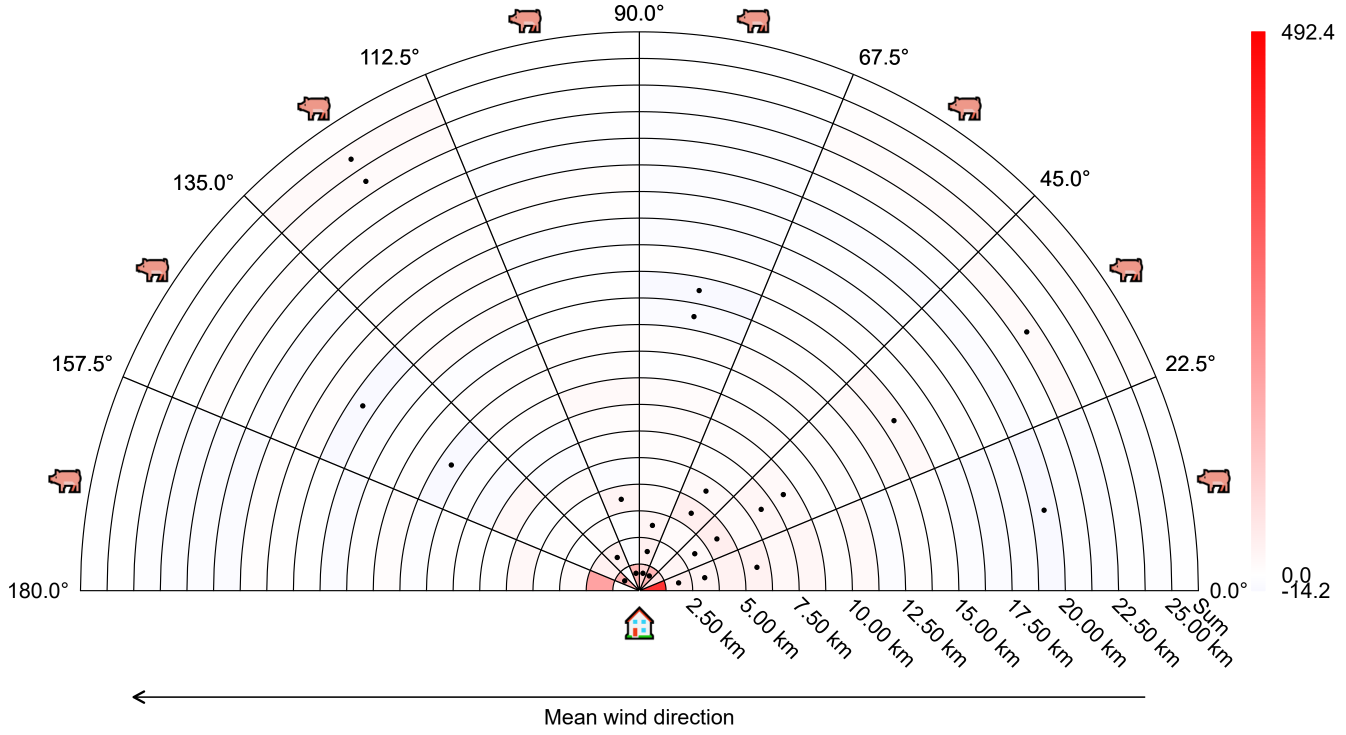

Wind summarized over 180 days

Crude analysis

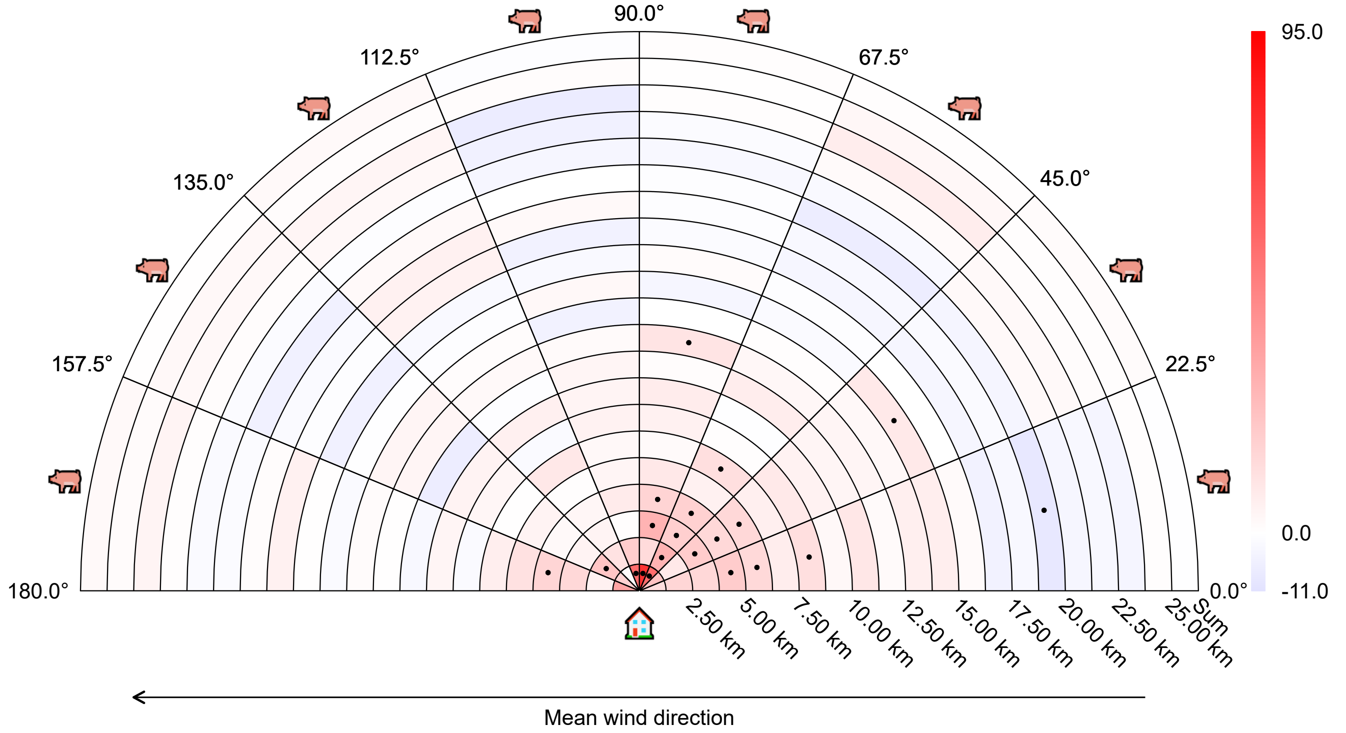

Adjusted analysis

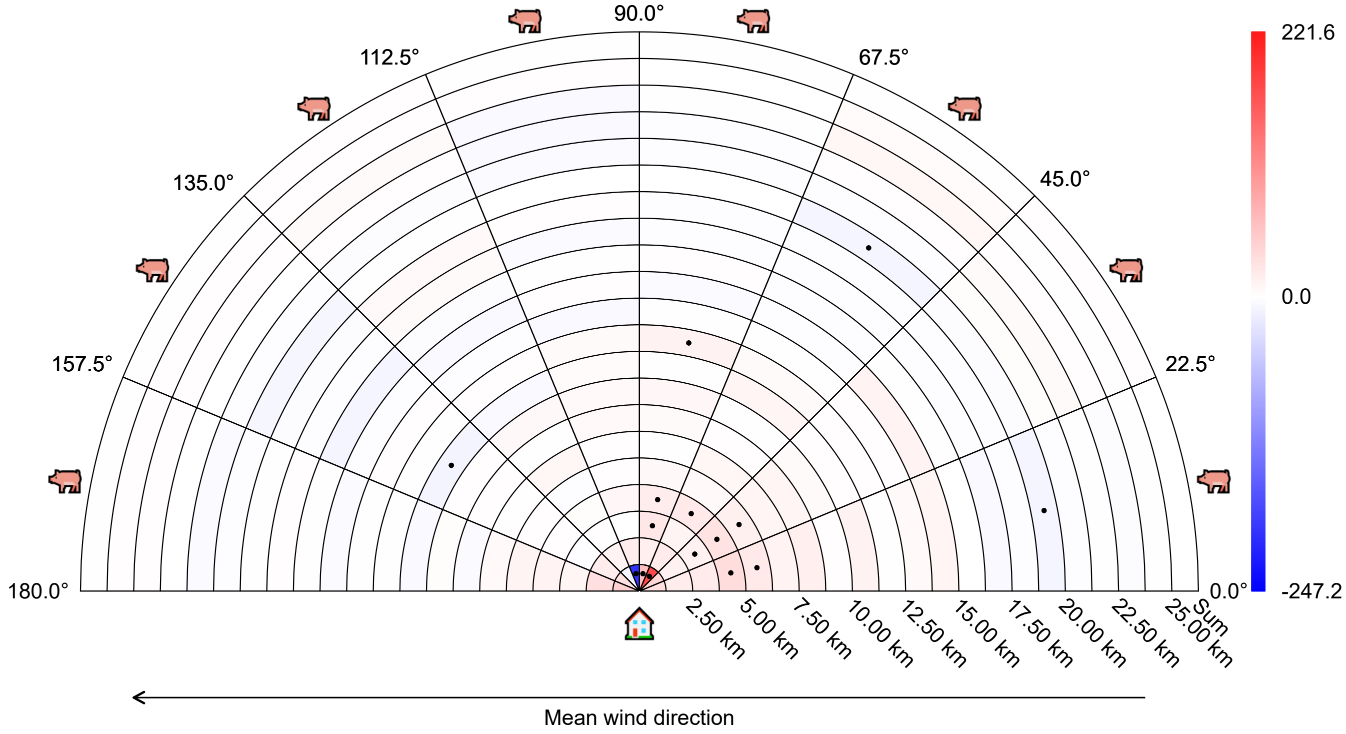

Wind summarized over 270 days

Crude analysis

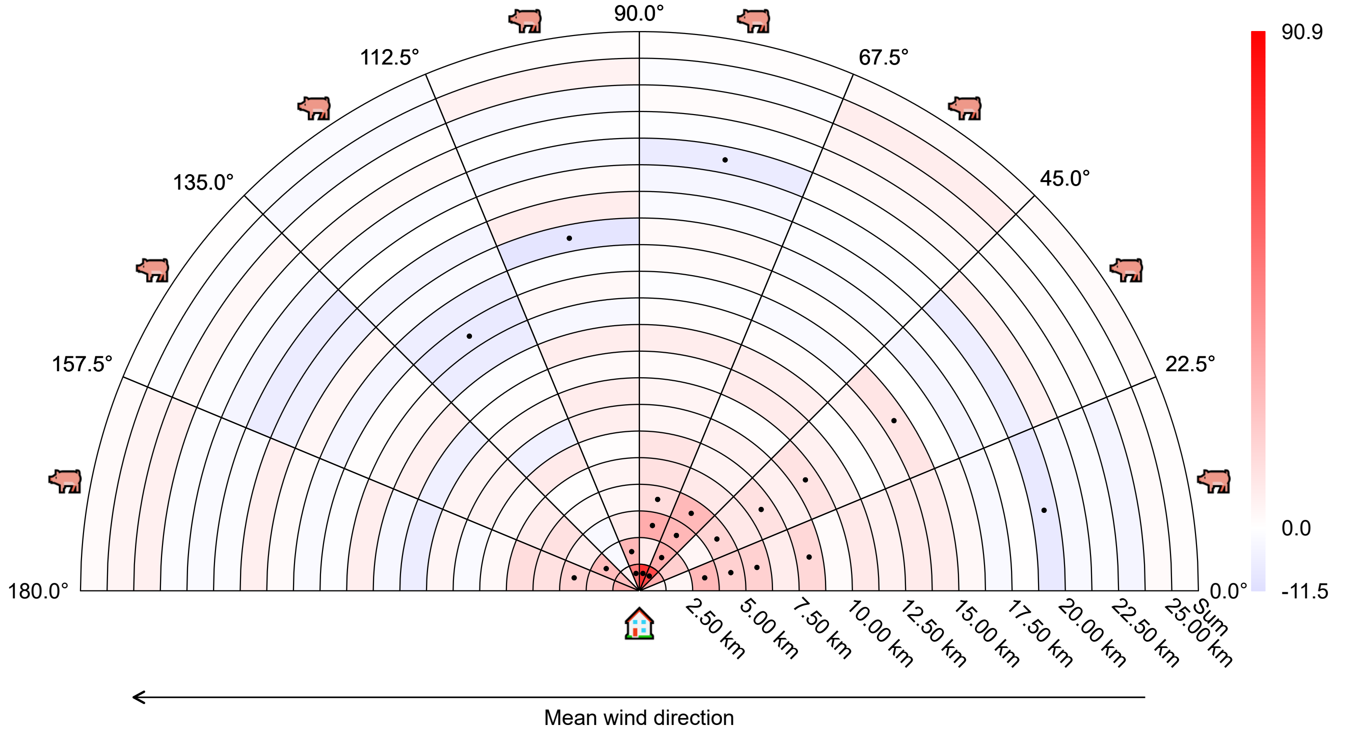

Adjusted analysis

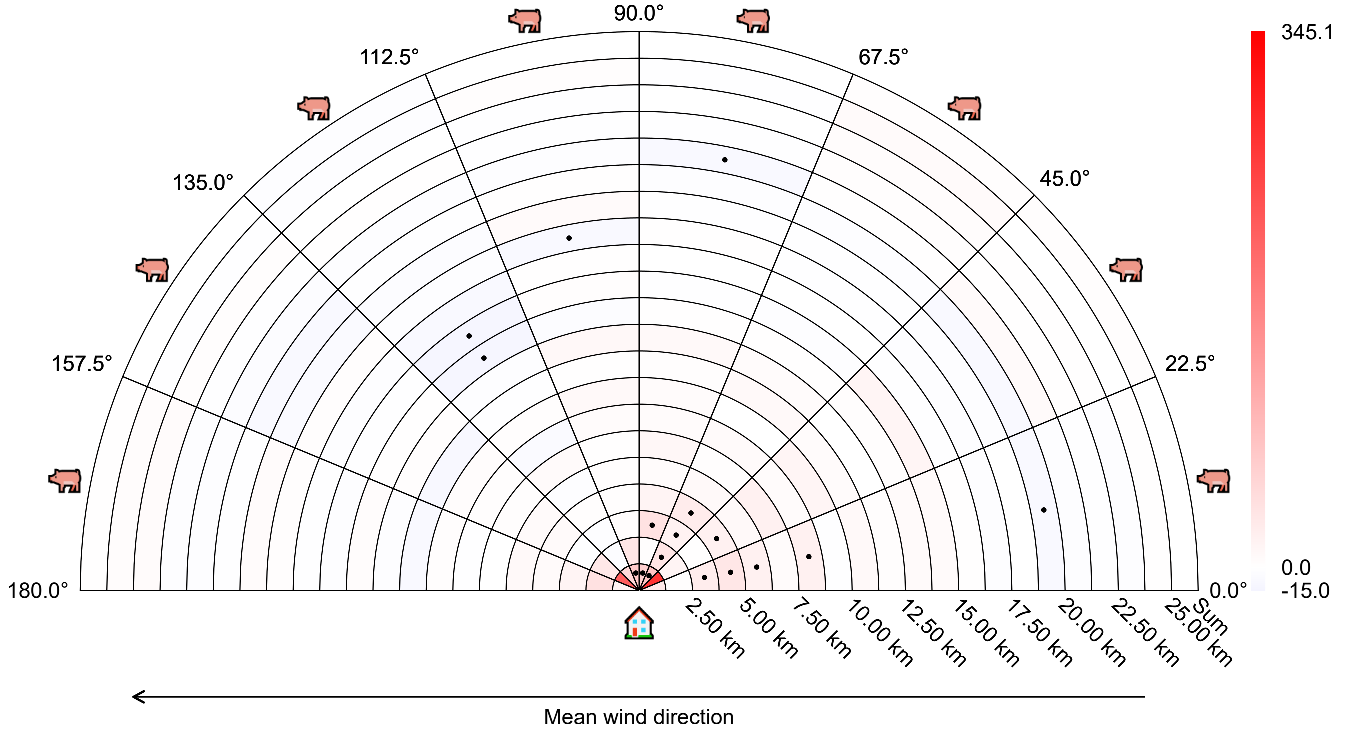

Wind summarized over 365 days

Crude analysis

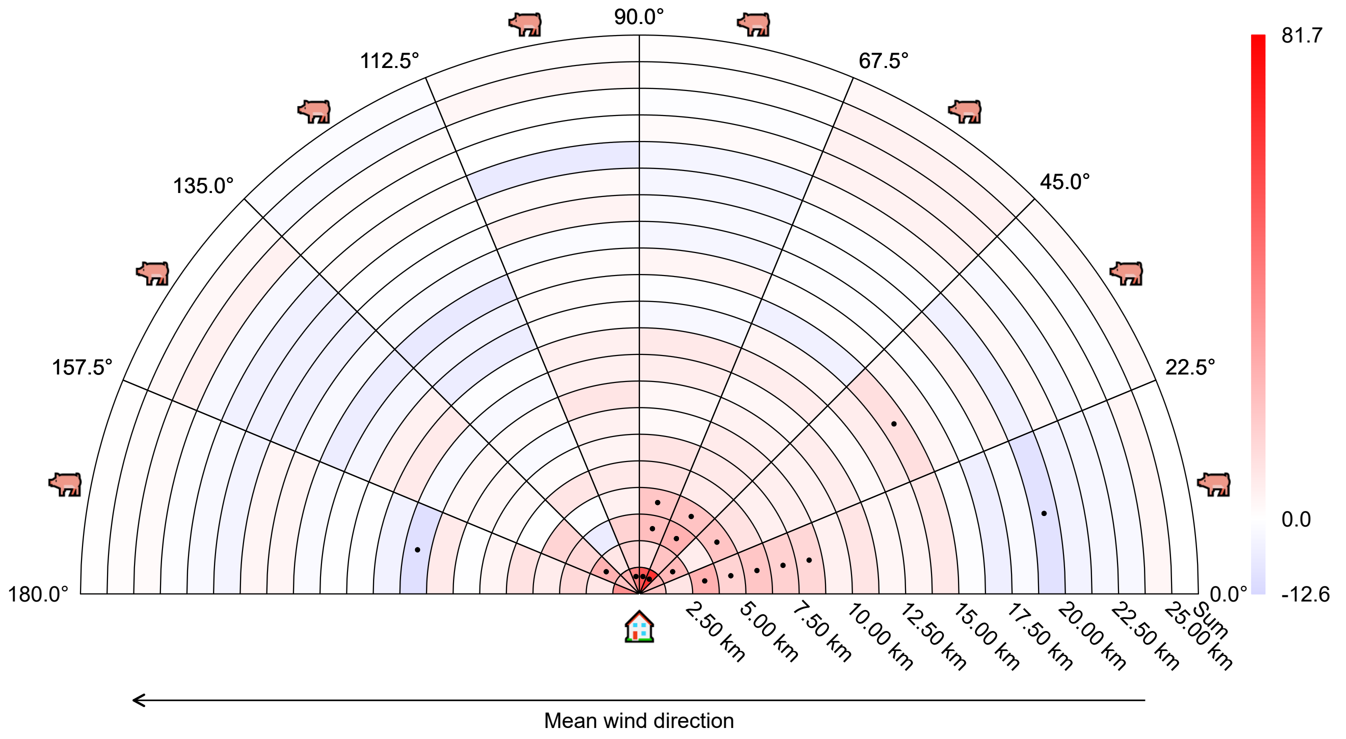

Adjusted analysis

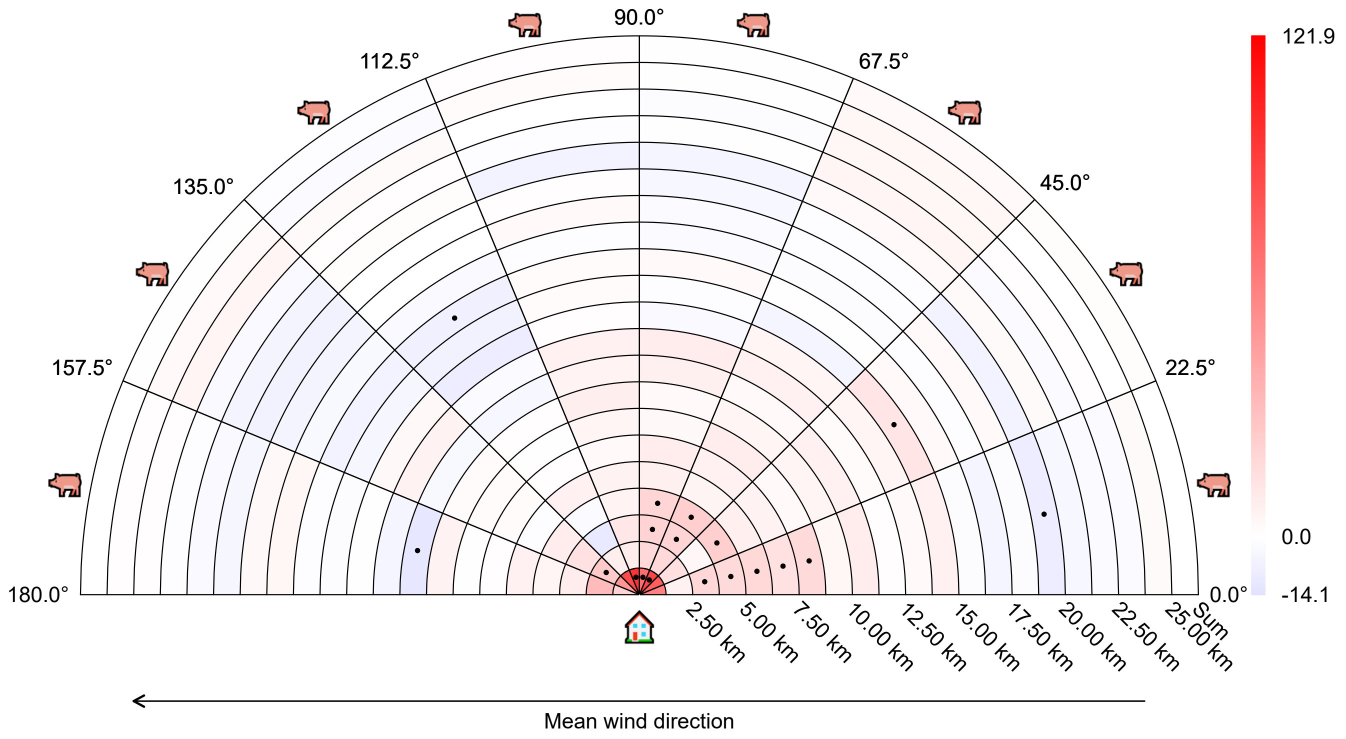

Supplement: Supplementary file 4 — Supplementary Material 4: Results from all post-hoc secondary analyses [file 15010_2025_2629_MOESM4_ESM.pdf]
