# Supplementary material for: Influence of wind direction on the relationship between proximity to pig farms and risk of infection with MRSA CC398 among persons without known contact to livestock: a Danish nationwide population-based study
Source: Infection. 2025 Sep 8;53(6):2795–808. doi: 10.1007/s15010-025-02629-2 (PMC12675557; doi:10.1007/s15010-025-02629-2)

Online supplement S5: Graphical presentation of wind direction in various time intervals

Wind-based calculations in the project were based on the ERA5 weather model (https://doi.org/10.24381/cds.adbb2d47) that has a spatial resolution of 0.25°×0.25°.

To assess whether our analyses could potentially be improved by using wind data with a higher spatial resolution, the following pages show plots of wind direction in each of the grid cells from the ERA5 model covering Denmark proper. Arrows indicate the direction of wind vectors (i.e., the direction of the airflow, which is the opposite of the meteorological wind direction).

We have summarized the wind direction in 9 random time intervals for each of the durations 7, 14, 21, 30, 90, 180 and 270 days, as well as in each of the calendar years from 2015 to 2021.

The outline of Denmark on the map is based on the dataset DAGIREF_SHAPE_UTM32-EUREF89 from Kortforsyningen.

Wind summarized over 7 days

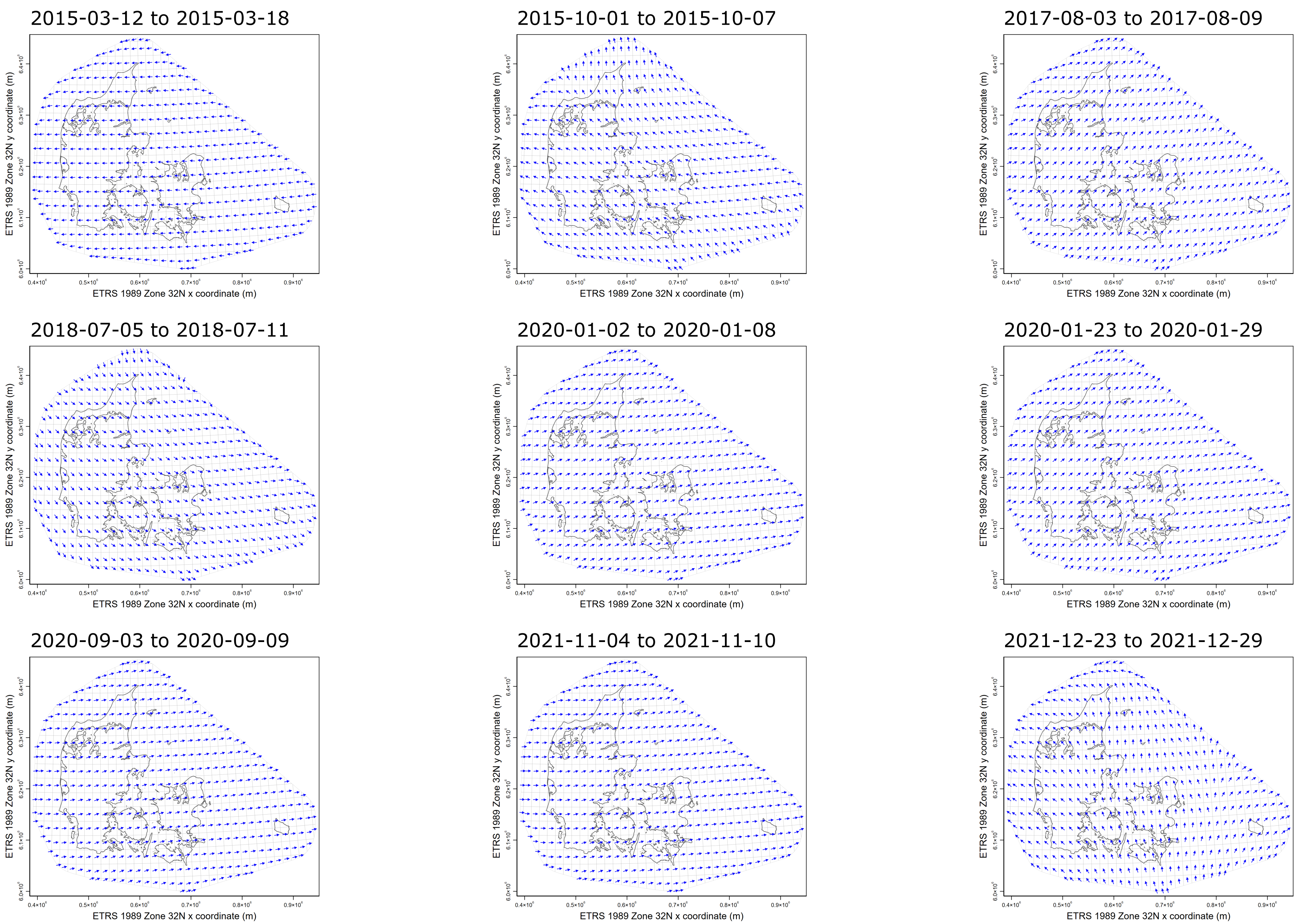

Wind summarized over 14 days

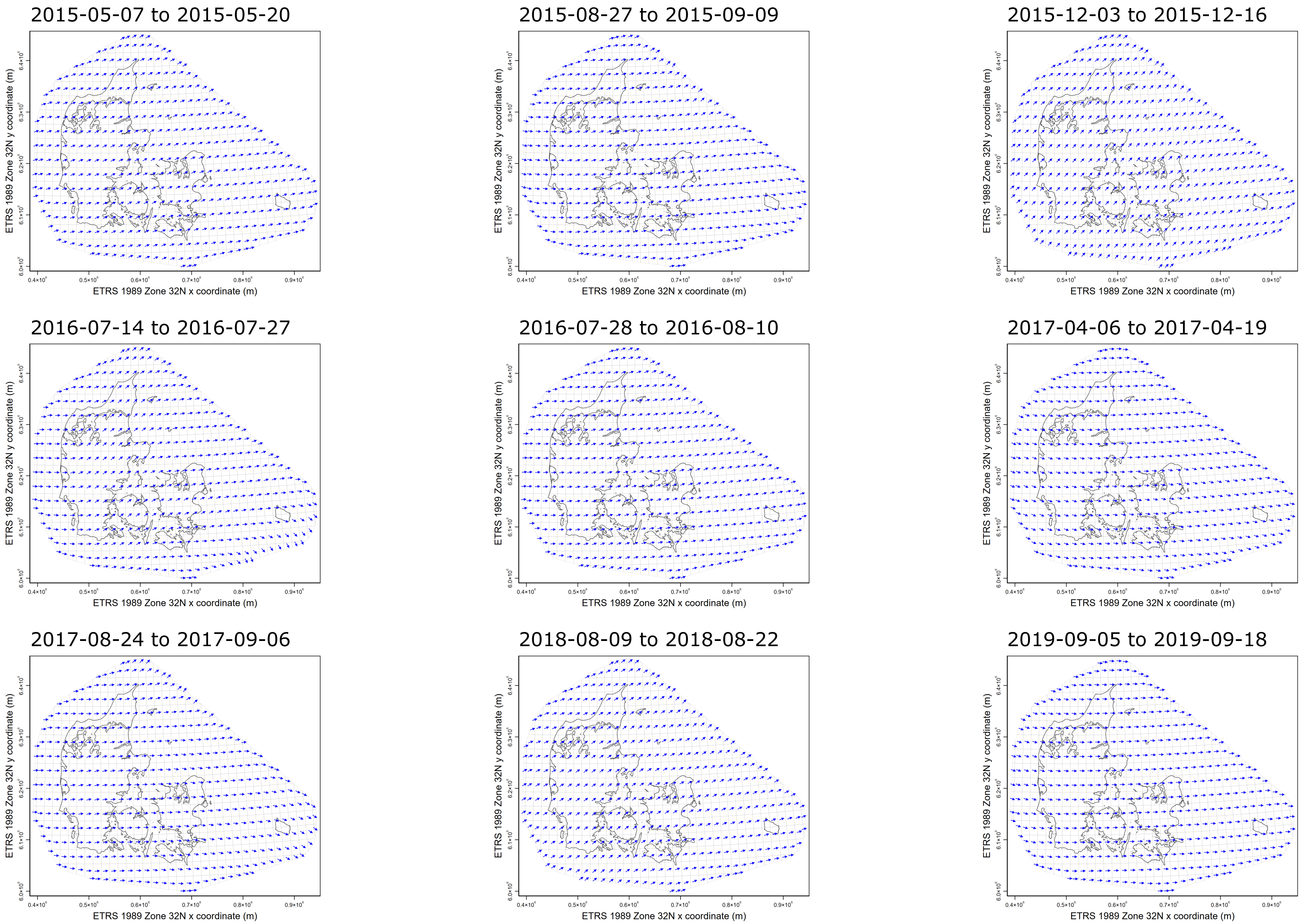

Wind summarized over 21 days

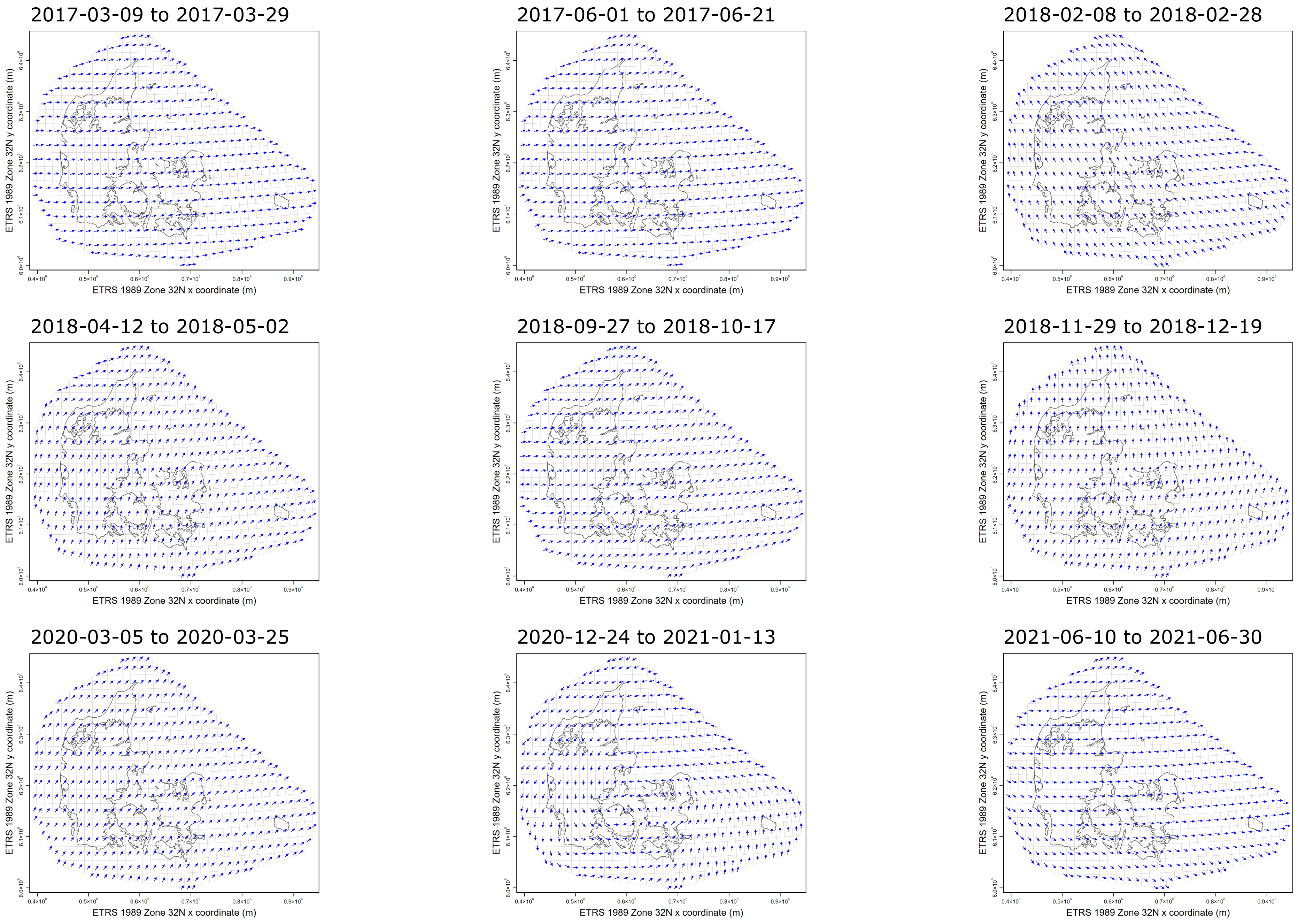

Wind summarized over 30 days

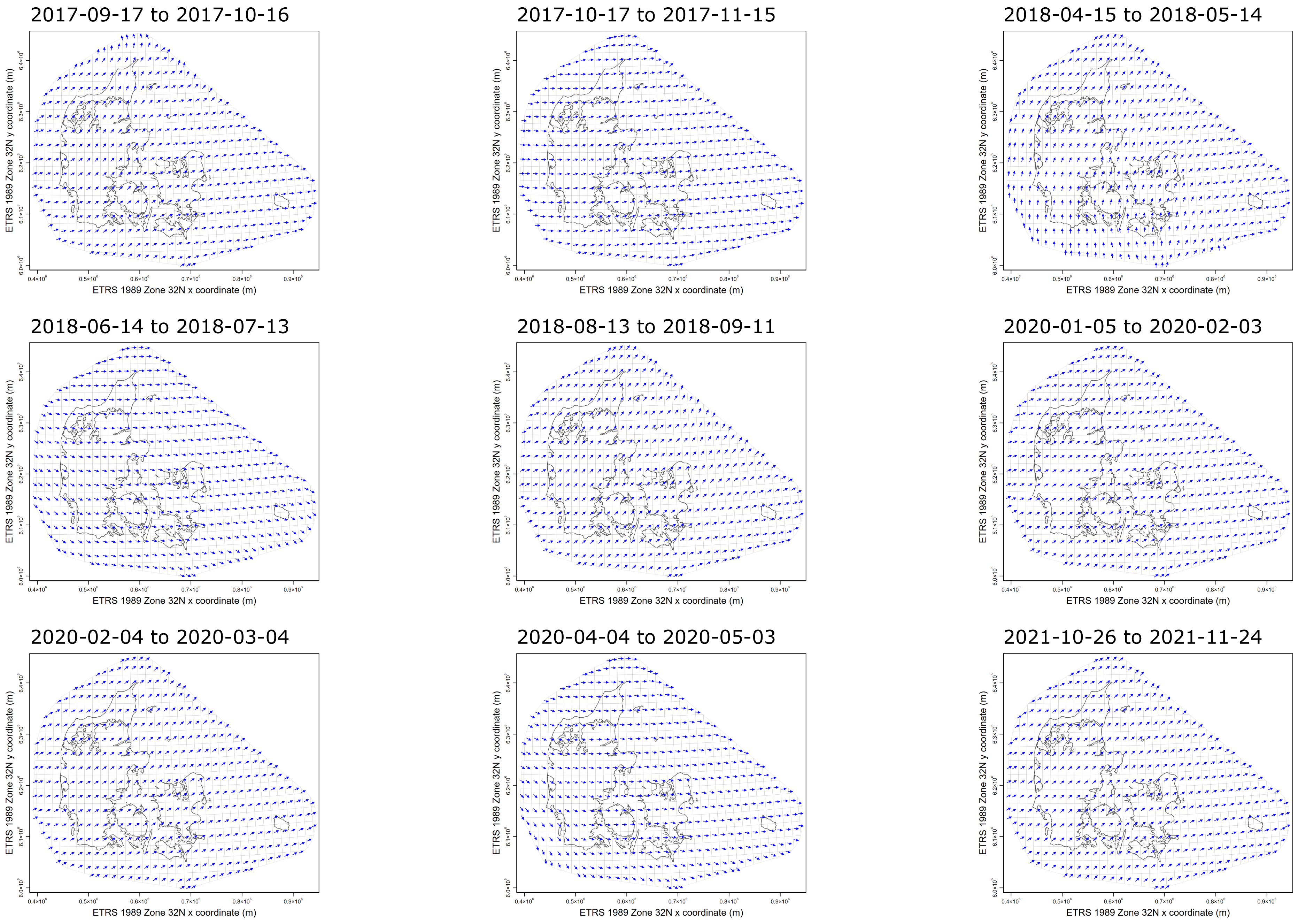

Wind summarized over 90 days

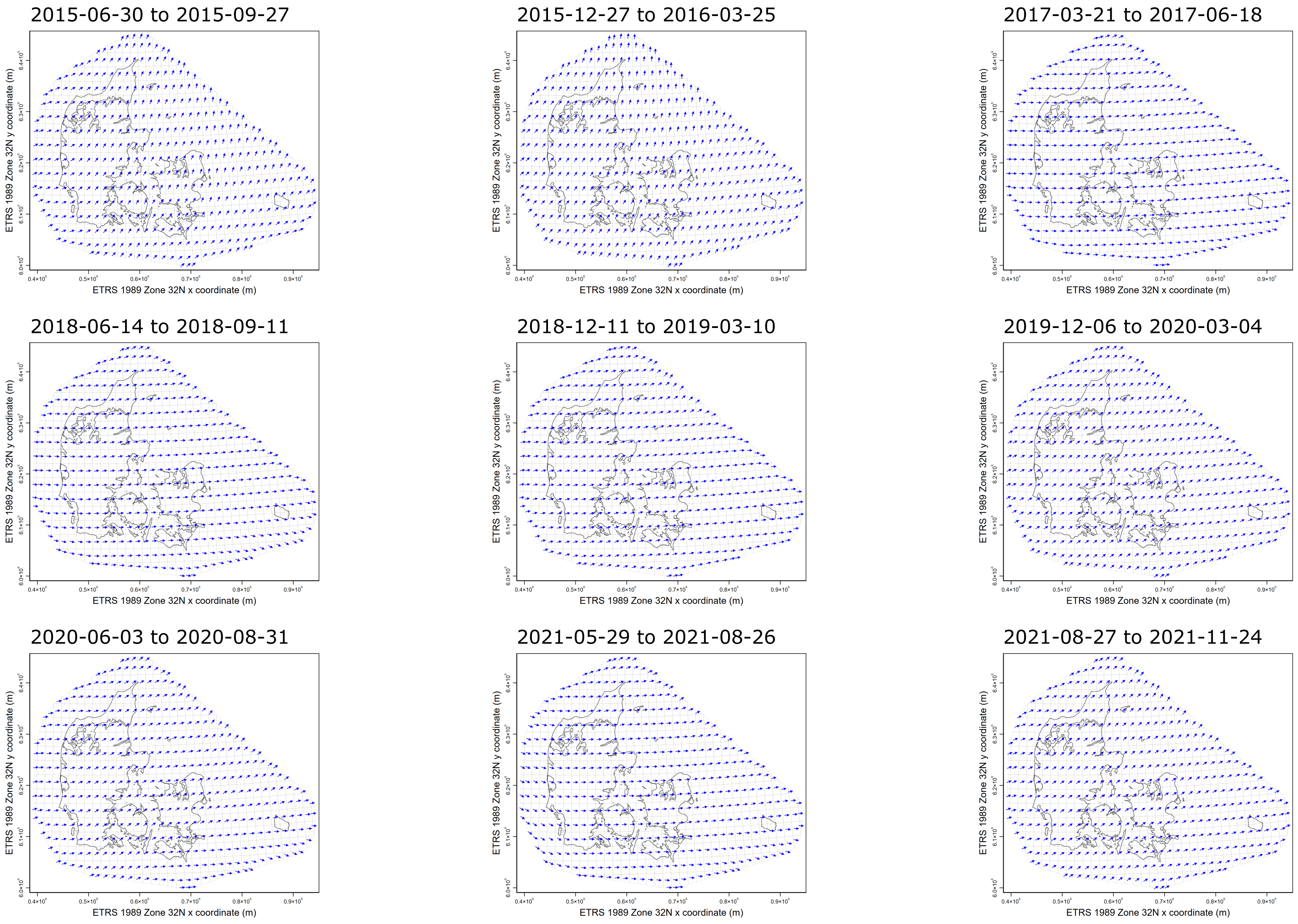

Wind summarized over 180 days

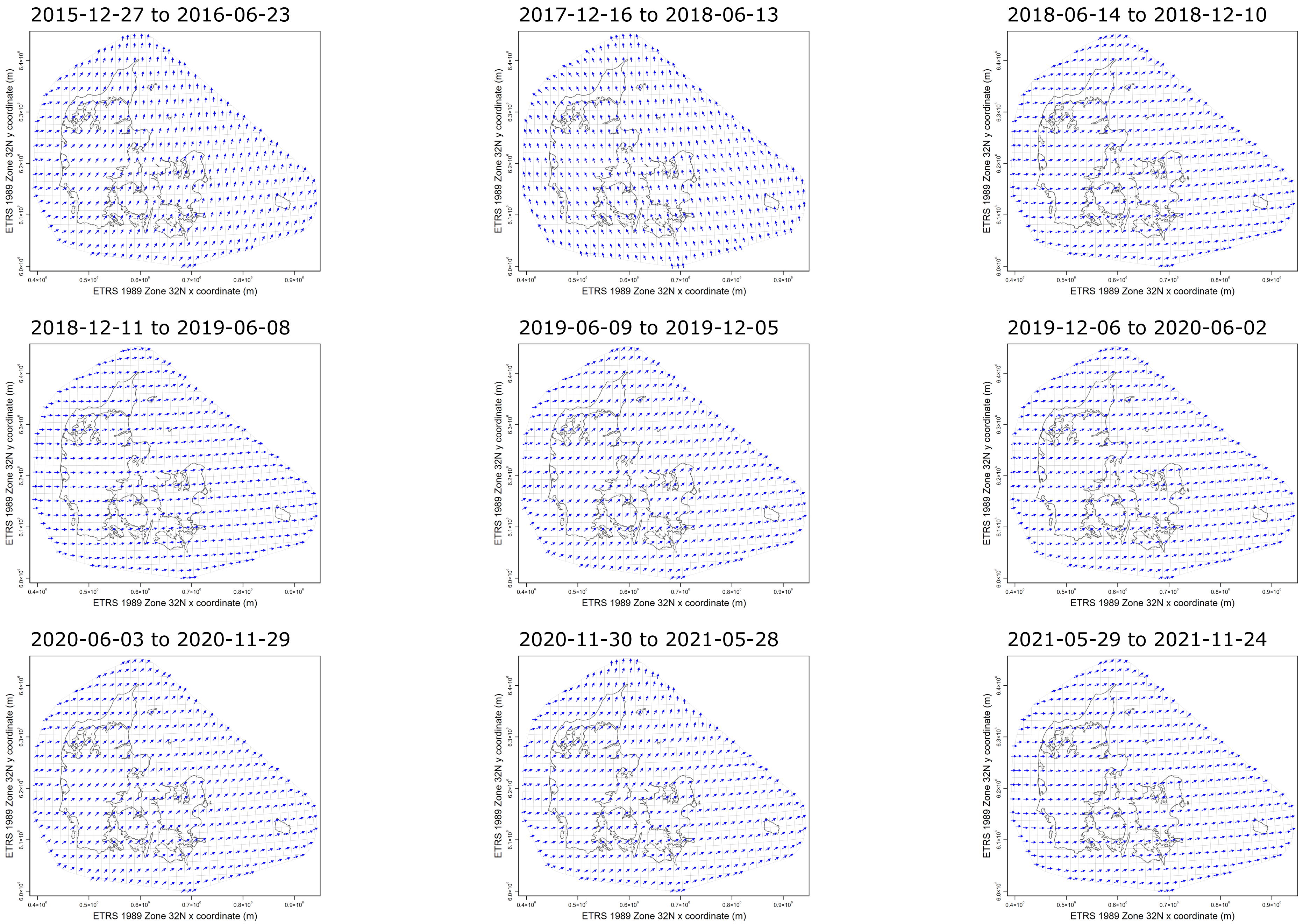

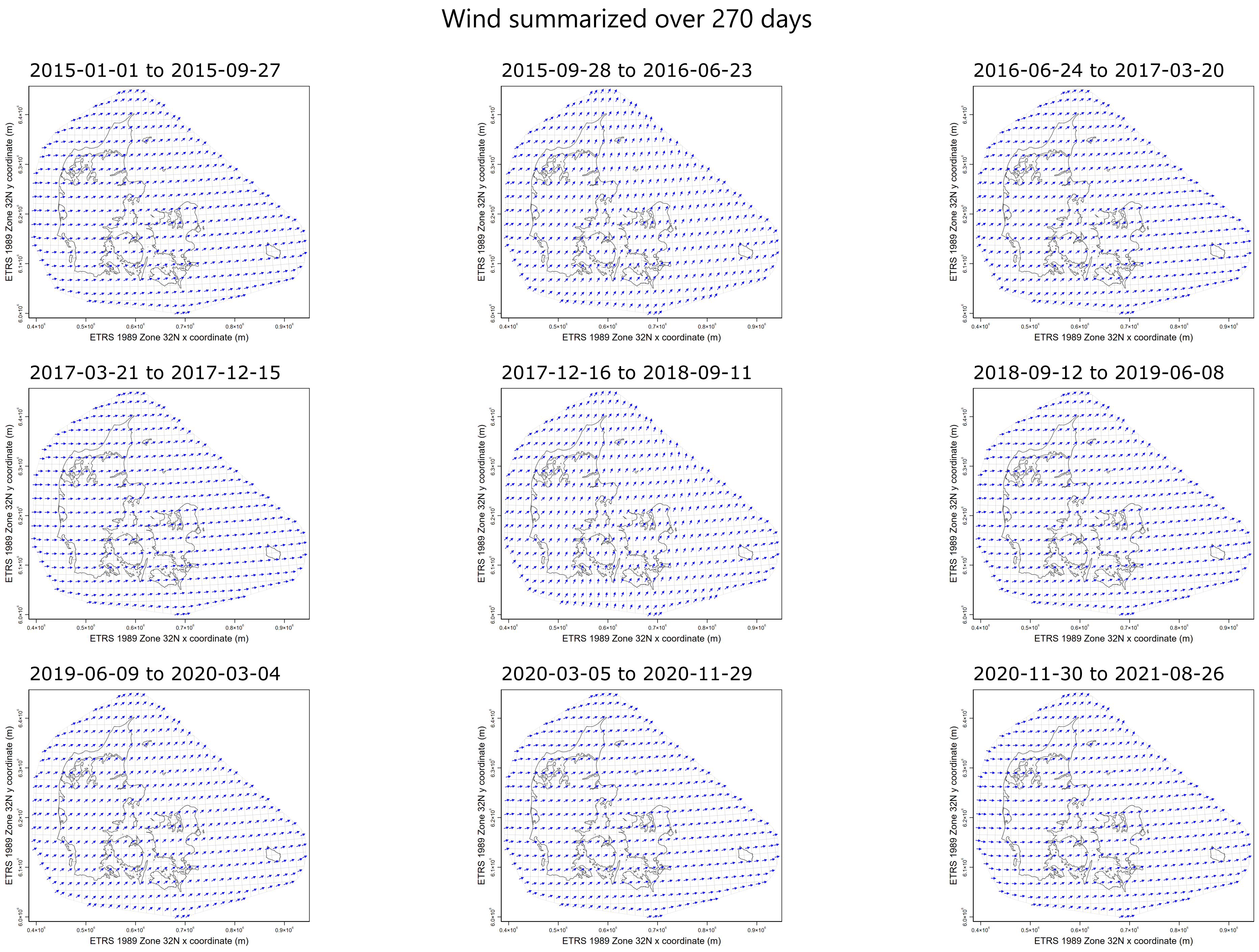
Wind summarized over 270 days
2015-01-01 to 2015-09-27
2015-09-28 to 2016-06-23
2016-06-24 to 2017-03-20
2017-03-21 to 2017-12-15
2017-12-16 to 2018-09-11
2018-09-12 to 2019-06-08
2019-06-09 to 2020-03-04
2020-03-05 to 2020-11-29
2020-11-30 to 2021-08-26
ETRS 1989 Zone 32N x coordinate (m)
ETRS 1989 Zone 32N y coordinate (m)

Wind summarized over 1 calendar year

2015-01-01 to 2015-12-31

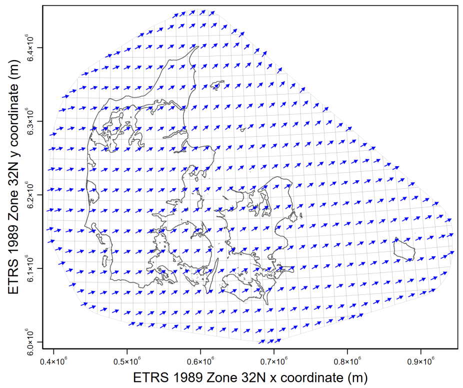

2016-01-01 to 2016-12-31

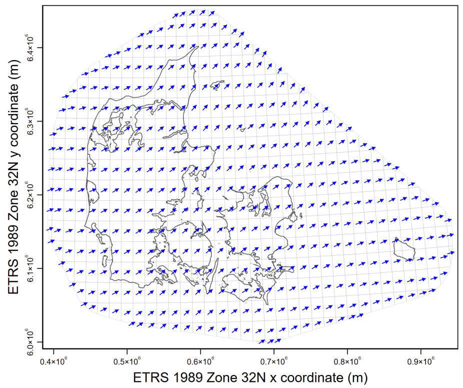

2017-01-01 to 2017-12-31

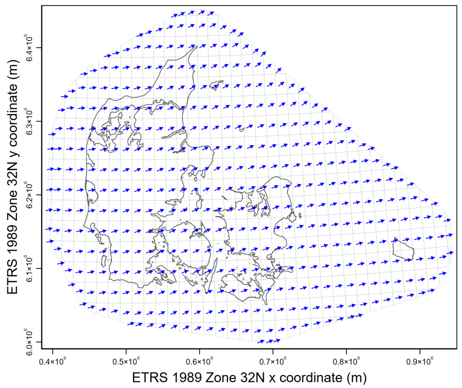

2018-01-01 to 2018-12-31

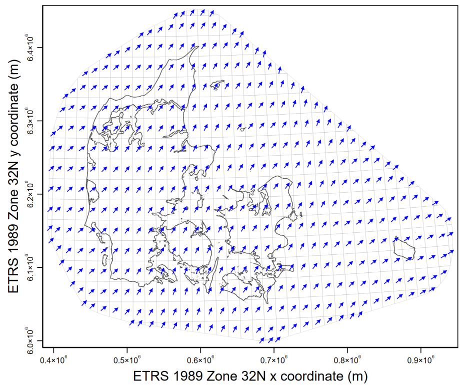

2019-01-01 to 2019-12-31

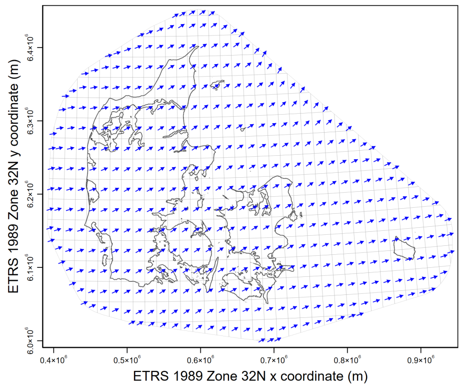

2020-01-01 to 2020-12-31

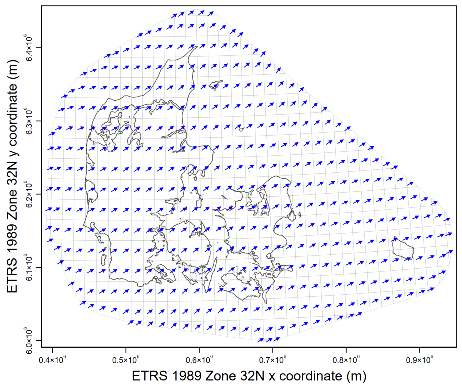

2021-01-01 to 2021-12-31

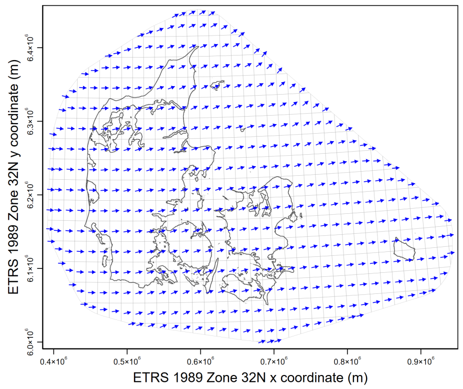

Supplement: Supplementary file 5 — Supplementary Material 5: Graphical presentation of wind direction in Denmark proper in various time intervals [file 15010_2025_2629_MOESM5_ESM.pdf]
